# Emirates Heart Health Project (EHHP): A protocol for a stepped-wedge family-cluster randomized-controlled trial of a health-coach guided diet and exercise intervention to reduce weight and cardiovascular risk in overweight and obese UAE nationals

**DOI:** 10.1371/journal.pone.0282502

**Published:** 2023-04-10

**Authors:** Jeffrey K. King, Mohamud Sheek-Hussein, Nico J. D. Nagelkerke, Alexander Kieu, Saif Al-Shamsi, Javaid Nauman, Nicholas Hoque, Romona D. Govender, Iffat ElBarazi, Kristoffer Crawford

**Affiliations:** 1 Department of Family Medicine, College of Medicine and Health Sciences, United Arab Emirates University, Al Ain, Abu Dhabi, United Arab Emirates; 2 Home Based Primary Care, Division of Geriatrics and Extended Care, Greater Los Angeles, Department of Veterans Affairs, Los Angeles, California, United States of America; 3 Institute of Public Health, College of Medicine and Health Sciences, United Arab Emirates University, Al Ain, Abu Dhabi, United Arab Emirates; 4 Department of Bioengineering, Imperial College London, London, United Kingdom; 5 Kanad Hospital, Al Ain, Abu Dhabi, United Arab Emirates; 6 Department of Internal Medicine, College of Medicine and Health Sciences, United Arab Emirates University, Al Ain, Abu Dhabi, United Arab Emirates; 7 Department of Pediatrics, College of Medicine and Health Sciences, United Arab Emirates University, Al Ain, Abu Dhabi, United Arab Emirates; 8 School of Public Health, Loma Linda University, Loma Linda, California, United States of America; Endocrinology and Metabolism Population Sciences Institute, Tehran University of Medical Sciences, ISLAMIC REPUBLIC OF IRAN

## Abstract

**Introduction:**

Cardiovascular disease (CVD) is the most common cause of death both globally and in the United Arab Emirates. Despite public health measures and health education, the rates of death from CVD remain stable. Barriers previously identified to lifestyle changes include cultural reasons, boredom, and lack of family support. The Emirates Heart Health Project (EHHP) seeks to support healthy lifestyle changes through a family-based intervention using a health coach and fitness tracker.

**Methods and analysis:**

The EHHP is a stepped-wedge cluster-randomized trial with each cluster comprised of members of an extended family. Eligible participants will be ≥ 18 years of age, with BMI ≥ 25, have Emirati citizenship and be able to give informed consent for study participation. The cluster will have 16 weekly teaching sessions in the participants’ family home by a health coach who will review individual weight, diet and exercise (monitored by a wearable fitness tracker). The clusters will have pre-intervention assessments of their weight and CVD risk profile and enter the intervention in randomized order. Each cluster will have a post-intervention assessment of the same measures. The primary outcome is weight reduction from baseline. Secondary outcomes will include change in CVD risk factors such as systolic and diastolic blood pressure, hemoglobin A1c, total cholesterol, LDL cholesterol, HDL cholesterol and triglycerides, waist circumference, and BMI. A mixed linear model will be used for analysis, where the parameters measured at the end of each 16-week episode will be the outcome values. These will be analyzed such that baseline values (measured just prior to the start of an episode) will be fixed covariables. Random effects are the family units. This trial has been registered with the NIH at clinicaltrials.gov (NCT04688684) and is being reported using the SPIRIT (Standard Protocol Items: Recommendations for Interventional Trials) and TIDieR (Template for intervention description and replication) framework.

**Trial registration:**

Clinicaltrials.gov NCT04688684.

## Introduction

Non-communicable diseases overtook infectious diseases as the leading cause of death in the past century, and despite concerted efforts, continue to result in the deaths of millions worldwide each year. Despite methodological challenges, a growing body of literature gives mixed evidence that links these diseases with modern lifestyles, which include an increased intake of calorie-dense nonnutritive foods combined with a lack of physical activity leading to obesity [1, 2] and overweight [3] which increases the risk of developing cardiovascular disease [4, 5] as well as cancer [6]. These trends are mirrored in the country of the United Arab Emirates (UAE), as well as in the specific location of this clinical trial, the emirate of Abu Dhabi [7, 8].

### Existing knowledge

The latest available data from 2017 show that cardiovascular disease (CVD) is the leading cause of death among the population residing in the emirate of Abu Dhabi [9]. Particularly striking is that CVD was listed as the cause of death in over 39% of those over the age of 45. The most recent available data show that in those over 60 years of age, mortality from CVD (n = 617, 62% of 996 total deaths) was more than 2.5 times the combined mortality from the second, third and fourth leading causes of death (n = 241): cancer (n = 121), respiratory diseases (n = 69), and infectious disease (n = 51), respectively. The higher mortality rate due to CVD has been stable over time despite concerted efforts, in contrast to significant progress in reducing mortality due to injuries and genetic disease (Table 1). For example, a government-funded screening program named Weqaya (meaning “prevention”) has been in place since 2008 for citizens of the UAE to annually evaluate CVD risk factors such as waist circumference, body mass index (BMI), blood pressure, lipids, tobacco use, and the presence of diabetes or prediabetes. Smoking cessation clinics, obesity clinics, and chronic disease clinics were also instituted within the government primary health centers to address these risk factors, and patients identified through screening as having CVD risk are linked to these services.

**Table 1:** Causes of death in the emirate of Abu Dhabi, 2001–2017.

	2017	2016	2015	2014	2013	2012	2011	2010	2009	2008	2007	2006	2005	2004	2003	2002	2001
Total	3262	3283	3163	3153	3015	2923	2902	2879	2988	2949	2742	2450	2446	2489	2492	2617	2574
Diseases of the circulatory system	1198	1219	1105	1107	1107	1135	1089	762	707	697	506	378	424	413	624	778	622
Injuries	562	653	674	587	590	532	581	587	689	549	621	503	565	563	574	614	600
Neoplasms	524	500	500	487	468	474	461	461	397	360	370	315	294	298	276	289	252
Congenital malformations & chromosomal abnormalities	78	89	97	117	62	85	72	144	199	120	177	131	156	146	152	199	190
Endocrine, nutritional and metabolic diseases	63	70	83	78	73	63	98	194	210	79	201	130	133	126	103	73	65
Other	837	752	704	777	715	634	601	731	786	1144	867	993	874	943	763	664	845

Source: Department of Health, Abu Dhabi Health Statistics 2017: A healthier Abu Dhabi. November 2018.

Citizens of the UAE, called Emiratis, belong to a collectivistic culture centered on the family and the religion of Islam. The UAE has rapidly incorporated cutting-edge technology in both the health and social sectors. For example, the rate of smartphone use penetration is one of the highest in the world at 91% [10], and telemedicine has become part of routine clinical care [11]. At the same time, traditional values have been preserved, such as the extended family gathering weekly for lunch after Friday prayers, which can include dozens of family members. It also remains common to eat together from a communally prepared meal, which makes it challenging to make drastic changes in the individual diet. Modesty is also highly valued, which in some families, along with extreme heat for several months of the year, has limited options for outdoor physical exercise, especially for women.

These factors may at least partially explain why rates of obesity, overweight, and CVD risk factors have remained stable despite greatly expanded screening and referrals to obesity clinics and dieticians in the primary health centers with no out-of-pocket cost. In fact, studies done in the UAE show that despite widespread knowledge of healthy diet and exercise, multiple barriers persist to lifestyle change. The findings of a paper-based survey showed that a sub-optimal diet was the leading risk factor for deaths due to cardio-metabolic disease (CMD) and estimated that this accounted for 72% of CMD deaths in the UAE, which would account for an excess mortality of 444 lives out of the 617 deaths recorded in 2017. In particular, the low consumption of whole grains was responsible for 22% of CMD deaths in the UAE. Other risk factors included high levels of processed meat, red meat, and sugar sweetened beverages [7].

A survey of patients in the UAE with type 2 diabetes showed only 3% met numerous guidelines’ recommendations for 150 minutes of moderate intensity aerobic activity or 75 minutes of vigorous aerobic activity per week [12]. The reasons given for not exercising included cultural reasons (29.2%), “exercise is boring” (20.3%), and lack of family support (4.1%).

Individual appointments are available through primary health centers to access dieticians and physician-led obesity programs, which include the use of lifestyle interventions as well as pharmacologic interventions such as metformin, orlistat, and liraglutide. These services are available at no out-of-pocket cost to Emiratis who meet certain clinical criteria. Despite wide access to these services which comes at significant cost to the health care system, the overall goal of reduction of harm has not been achieved.

The Emirates Heart Health Project (EHHP) is a culturally sensitive, family-based intervention utilizing a health coach and leveraging smartphone and wearable sensor technology to promote lifestyle changes based on the highly successful and durable Diabetes Prevention Program (DPP) curriculum. Given the high rates of smartphone use and openness to technology, it is hypothesized that the use of a wearable fitness tracker will allow for the collection and sharing of physical activity by participants with a health coach and members of the extended family, which may give added motivation and opportunities for positive reinforcement. Instant messaging between participants and the health coach can also allow for ongoing communication such as reminders and addressing challenges to sustained lifestyle change. At the same time, given the central role of family in the decisions individual participants make, a collective commitment on the part of an extended family to eat a healthier diet and participate in increased physical activity may address some of the documented perceived barriers to positive change, such as perceived cultural reasons, the monotony of exercise, and the lack of family support.

To our knowledge, no study has investigated the effect of a family-based diet and exercise intervention in the UAE. The EHHP is such an intervention that has adapted the DPP, which is based on individual participation and North American cultural values, into a family-based, culturally and religiously sensitive program with intensified access to a health coach. This trial is intended to assess whether a family-based program may result in better uptake and adherence to proven lifestyle-based interventions than the internationally standard, individual-based care that is currently offered as usual care. This may inform policy changes, allocation of resources, and future research addressing the leading cause of mortality in the UAE.

### Objectives

Research hypothesis: The EHHP, a family-based diet and exercise intervention using a family health coach with technological aids, reduces cardiovascular risk factors when compared to the current usual management of obese and overweight patients in the emirate of Abu Dhabi, United Arab Emirates.

#### Study objectives

*Primary objective*. To determine if the EHHP reduces weight and BMI in obese and overweight Emiratis.

*Secondary objectives*. To determine in obese and overweight Emiratis, if the EHHP has significant effect in:

- Reducing systolic and diastolic blood pressure;
- Reducing hemoglobin A1c;
- Reducing total cholesterol, LDL cholesterol and triglycerides;
- Increasing HDL.
- Decreasing waist circumference
- Decreasing BMI

## Methods and analysis

This protocol is being reported using the SPIRIT (Standard Protocol Items: Recommendations for Interventional Trials) and TIDieR (Template for intervention description and replication) framework and checklists (S1 and S2 Appendices).

### Trial design

Due to the family-based nature of the intervention package, the EHHP is designed as a stepped-wedge cluster randomized trial. Each cluster receives the intervention at a randomized time, which causes them to crossover from control to intervention. This type of study design has logistical advantages as well as having utility in evaluating the group-level impact of interventions that have been proven effective in individually randomized trials [13, 14]. There exists an extensive literature on the conduct, benefits, and limitations of stepped-wedge studies, some of which is available as open-source [15]. For an expanded discussion of the specific benefits and limitations in the context of the EHHP, please see the discussion section. The EHHP itself is based on the Diabetes Prevention Program, which has shown both short- and long-term benefit in multiple individual randomized control trials [16, 17].

Patients that present to Kanad Hospital with a BMI that meets inclusion criteria who give consent (S3 and S4 Appendices) for participation will be asked to invite other extended family members to participate. Participants will then provide contact information for family members to schedule appointments at Kanad Hospital for eligibility screening according to inclusion and exclusion criteria. Those meeting criteria will be invited to participate in the study and consent will be obtained. For logistical purposes, a minimum of eight participants per family will be required for enrollment. Once the family is enrolled, baseline measurements will be obtained.

**Fig 1:**
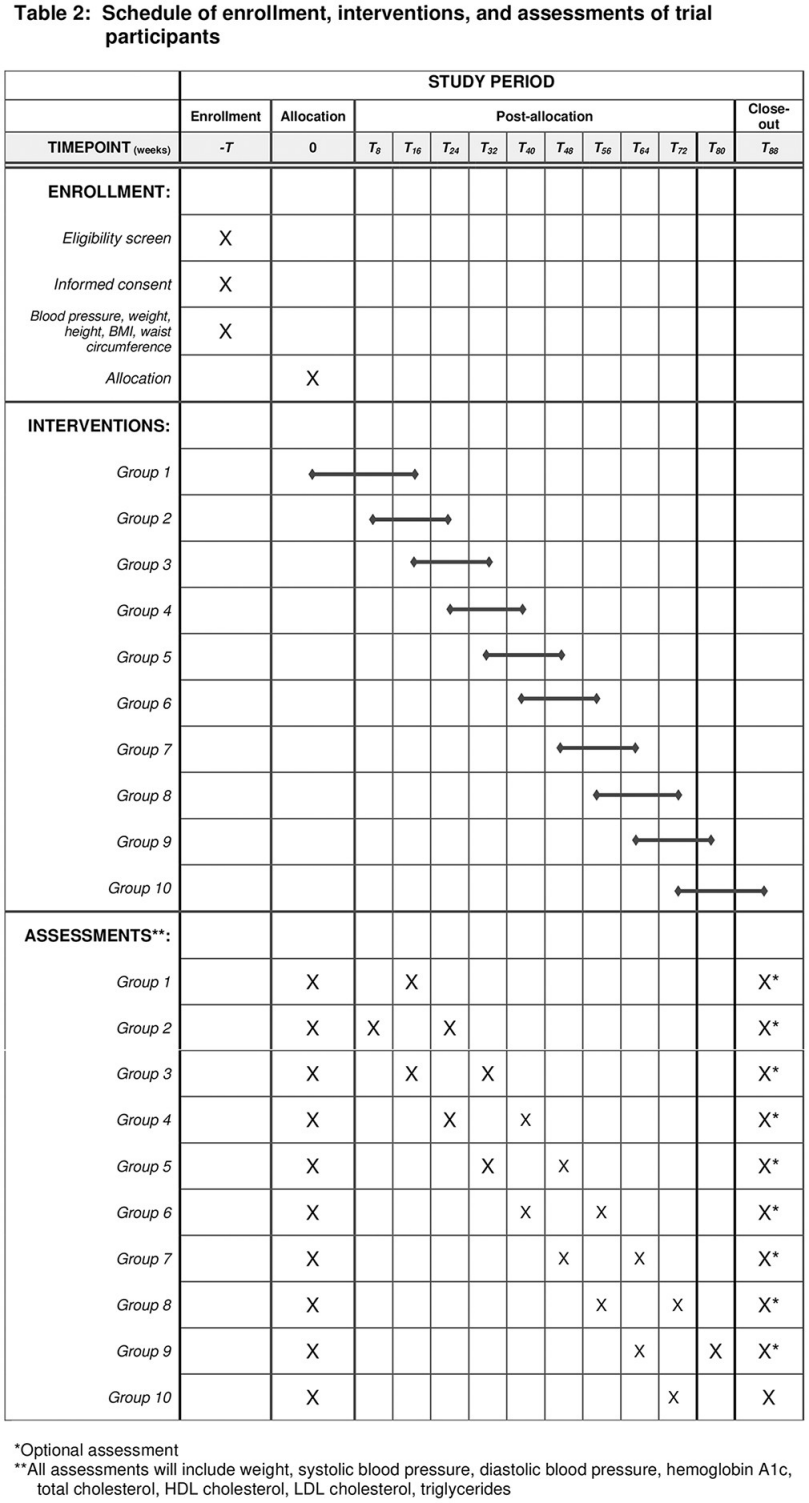
SPIRIT schedule of stepped intervention.

**Fig 2:**
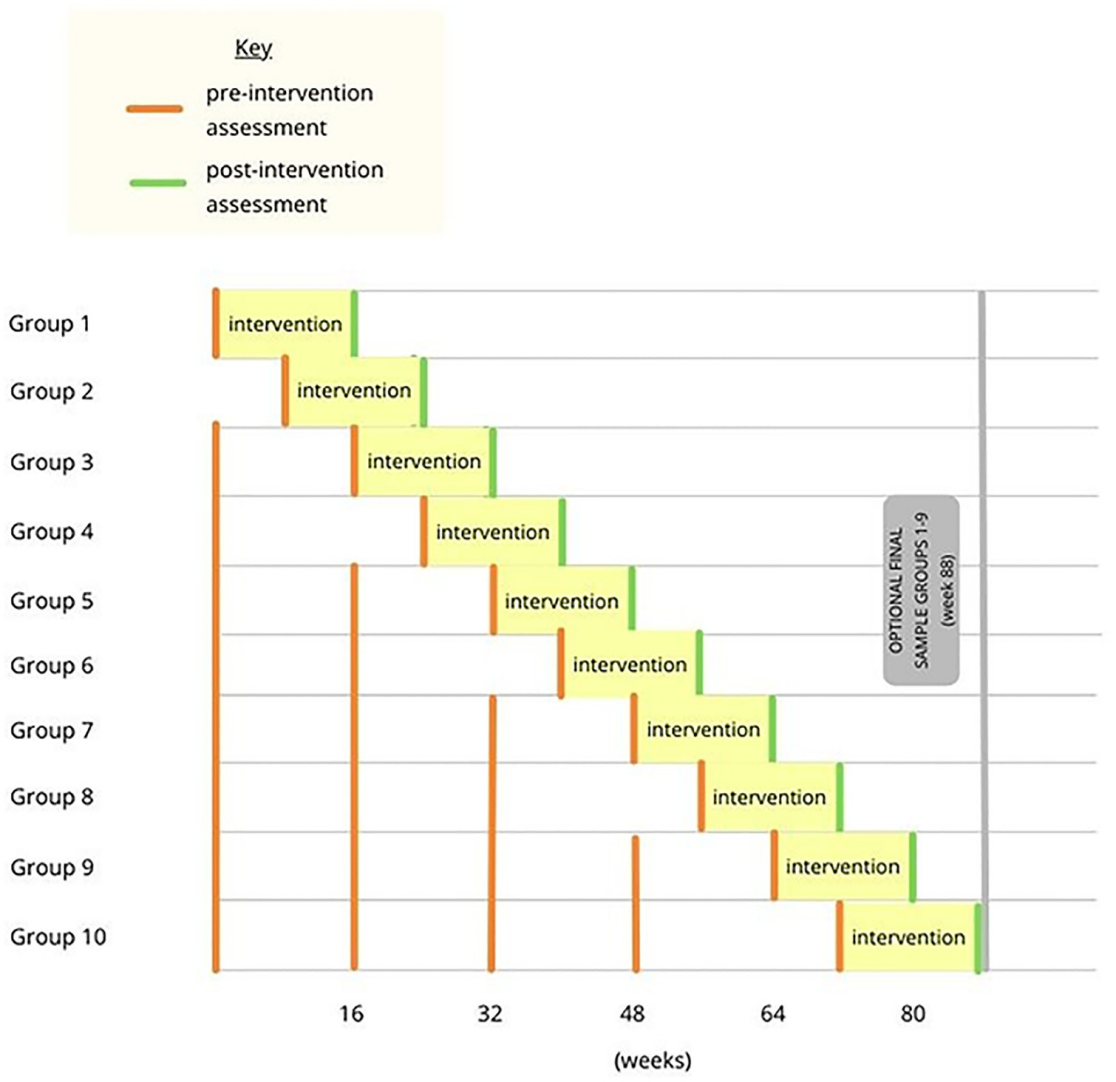
Graphical timeline of stepped intervention.

Once the prespecified number of family clusters are enrolled, they will be randomized by the statistical team (NN, MSH) using random number generation and assigned a number 1 through n, where n is the number of clusters. This number will be communicated to the health-coach by encrypted message and will provide order of intervention [18], with a cluster entering the intervention at each time as a cohort to maximize study power (Figs 1 and 2) [19].

Prior to being exposed to the intervention, the participants will be exposed to usual care which will comprise scheduled individual visits with a dietician, who will recommend individualized diet and exercise modifications. As noted below, individuals undergoing or planning to undergo either bariatric surgery or the use of weight-loss pharmacotherapy are not eligible to participate in this study. The available medications in our location include liraglutide, semaglutide, and orlistat.

Each cluster will thus be exposed to the intervention in a stepped fashion. The intervention, which will be described in further detail below, will take approximately 16 weeks to complete. Pre-intervention measurements will be taken approximately every 16 weeks until the cluster enters the intervention phase. These measurements will be compared with post-intervention measurements at the conclusion of the intervention to assess for a statistically significant difference. The specific type of trial is a closed cohort, complete stepped-wedge cluster randomized trial [13]. This study protocol has been approved by the Kanad Hospital Research Ethics Committee on March 16, 2021 with the assigned authorization #2012.15. Approval of amendments and renewal were given on March 16, 2022 with the same assigned authorization number.

#### Statistical analysis

A mixed (random + fixed effects) linear model will be used where the parameters (e.g., BMI) measured at the end of each 16-week episode will be the outcome (dependent, y variables) values. These will be analyzed such that baseline values (measured just prior to the start of an episode) will be fixed covariables, as well as whether the episode was an "intervention" or "control" episode. Random effects are the family units.

Episodes after the intervention episode will be excluded from the main analysis and used to explore the persistence of intervention effects.

#### Patient and public involvement

The research question and outcomes were chosen to assess clinically significant outcomes in reducing the morbidity and mortality of patients in the UAE. The intervention was designed based on prior surveys and analyses that revealed barriers to optimizing diet and exercise patterns in our specific setting. The results will be made available on completion of the study to participants, and to the public via publication in a medical journal. Patients or the public were not otherwise involved in the design or dissemination plans of our research and will not be involved in the recruitment to or conduct of this study.

#### Study setting

Study recruitment will take place in the outpatient clinics of Kanad Hospital in the emirate of Abu Dhabi, United Arab Emirates. This center is one of five major hospitals serving a region of 766,900 [20]. Citizens of the UAE, called Emiratis, are the focus of this trial and thus one of the inclusion criteria is that participants must be a citizen of the UAE. This is to limit the enormous genetic variability of the majority of the rest of the population, from which nearly all countries of the world are represented. In 2021, 51.3% of outpatient visits at the Kanad outpatient clinics were with Emirati patients, which will assist in enrolling sufficient numbers of study participants from the target population.

#### Eligibility criteria

Participants must provide written, informed consent before enrollment in the study.

*Inclusion criteria*. Patients eligible for the trial must meet all of the following at enrollment:

1. Emirati citizenship;
2. Age ≥ 18 years;
3. Ability to give informed consent for study participation;
4. BMI ≥ 25.

*Exclusion criteria*.

1. Current or planned use of weight-loss pharmacotherapy;
2. Recent (within 6 months) bariatric surgery;
3. Current diagnosis of cancer.

#### Interventions

*EHHP curriculum*. The Diabetes Prevention Program (DPP) is a well- known, durable, and broadly recommended diet and exercise program which results in improvement in known cardiovascular risk factors such as total cholesterol (TC), low density lipoproteins (LDL), triglycerides (TG), body mass index (BMI), blood pressure (BP) and glucose [14]. The DPP diet is flexible and not severely restrictive, and therefore is appropriate for the vast majority of people. The proportion of participants in the original DPP study remains high at the 10 and 15 year follow ups in the follow-on Diabetes Prevention Program Outcomes Study (DPPOS) with sustained improvement in cardiovascular risk profile when compared to placebo [16, 21, 22].

The DPP, on which the EHHP is based, is a flexible, individualized program that allows each participant to follow the program according to their own needs. For example, participants that are completely sedentary at study enrollment are advised to gradually and consistently increase their level of physical activity with the goal of performing 150 minutes a week of moderate aerobic exercise by the end of the intervention, rather than starting at some preset amount of exercise in week 1.

#### Guidance from a health coach

For the intervention group, a health coach will deliver 16 weekly teaching sessions (see S6–S35 Appendices for the English and Arabic language versions of the curriculum) in the participating family’s home. These teaching sessions have been contextualized from the DPP material to suit local culture and customs and translated from English to Arabic. The material for each session is largely scripted for standardization.

The health coach will review each participant’s weight, dietary logbook, and wearable fitness tracker exercise records weekly. Individual diets will initially be recorded using the daily food record sheets developed by the DPP, which have also been translated into Arabic. Study participants are anticipated to have a wide variety of health and technology literacy, and previous surveys have lost considerable amounts of data when standardized forms are required to be filled out by their study population. In the cases where participants are unable to utilize these sheets effectively, they will report caloric intake in whatever format the participant is able, and sent to the health coach for logging and analysis.

The Fitbit Charge 3 fitness tracker was selected due to its affordable cost and long battery life, which will reduce the rate of missing data due to insufficient charge. Makeup sessions will be arranged and given as needed for individuals who miss sessions. Each participant will be encouraged to participate in the family’s effort to adhere to the program, and to ask for help, advice, and support from the health coach during the intervention period.

Since this is a pragmatic trial that aims to have real world applicability, participants with diabetes, hypertension, or other comorbid conditions will be encouraged to continue any individual treatment plans under the ongoing care of their primary physicians and specialists. Participants are advised that medications may need to be adjusted by their preexisting care teams in case weight loss results in significant changes in their blood sugar or blood pressure. As long as the participant agrees to continue in the study, they will remain enrolled; the only criteria for stopping the intervention will be withdrawal of participant consent.

Prior studies performed in the UAE have faced the challenge of retention of participants during the study period. Similar challenges exist for the usual care of obese and overweight patients as loss to follow up with the dietician and obesity clinic team is common. The health coach is the primary measure that is expected to improve adherence and participant retention, as the therapeutic relationship is established. The investigators have selected a health coach who speaks Arabic, has thirty years of in-country experience as a medical interpreter and clinical care coordinator, is familiar with local customs and practices, and has a degree in psychology. It is also hoped that the introduction of the fitness tracker as an electronic method of quantifying physical activity will increase participants’ interest in adhering and continuing with the intervention.

### Outcomes

#### Primary outcome measure

The primary outcome measure is the difference between the pre- and post-intervention body weight. This aligns with the American Diabetes Association guidelines on the management of diabetes, as a 5–7% reduction in weight is associated with a clinically significant improvement in glycemic control and cardiovascular risk factors. Weight is also a recognized goal of the anticipated participants and easily measured.

#### Secondary outcome measures

Secondary measures include the absolute and relative change pre- and post-intervention in:

- Systolic blood pressure
- Diastolic blood pressure
- Hemoglobin A1c
- Total cholesterol
- LDL cholesterol
- HDL cholesterol
- Triglycerides
- Waist circumference
- BMI

Elevated blood pressure is strongly associated with the development of atherosclerotic disease [23, 24], as is the presence of diabetes or impaired glucose tolerance [25–27]. In addition, LDL cholesterol is recognized globally as a major risk predictor for major adverse cardiovascular events (MACE) [28]. Since our resources are limited which further limits the time that we will have for follow up, our secondary outcomes of interest are improvements in known risk factors for cardiovascular disease. These have been well delineated in the literature, and have been incorporated into various risk models [29]. Furthermore, these are the same secondary outcomes that are addressed in the previously mentioned Diabetes Prevention Program (DPP), on which the Emirates Heart Health Project is based. For reduction in A1c, a clinically significant reduction would be 0.5–1.1%, which is the expected benefit of adding an additional medication to treat hyperglycemia. The reduction achieved by lifestyle modification would have the equivalent expected benefit of adding another medication for glycemic control.

For HDL cholesterol, an increase of 10% would be considered beneficial, and for LDL, every 20-point reduction is expected to reduce 10-year cardiovascular risk. The percentage of this reduction depends on many factors such as age, sex, race, blood pressure, cholesterol levels, history of diabetes, and smoking status [30]. A 39 mg/dL reduction in LDL cholesterol was shown to reduce the risk of major cardiovascular events by 20% using statin and non-statin medications. This suggests that lifestyle modifications that achieve similar LDL reductions may also significantly reduce cardiovascular risk [31].

#### Participant timeline

utcome measures will be assessed in eligible family members after enrollment and informed consent. Depending on the randomized order of entry, between one and four pre-intervention assessments will take place while the participant is receiving individual care with the dietician and primary care team. Once the cluster enters the intervention, the sixteen in-home sessions will then be delivered to the family by the health coach with inter-session coaching and follow up. After completion of the final session, the participant will have repeat outcome measurements [Figs 1 and 2 and S36 Appendix].

#### Sample size

In order to calculate an appropriate sample size without reliable information on the intracluster correlation we will calculate our sample size in terms of the number of clusters required. This also takes into account the cluster design of the study. The sample size in terms of individuals will then be the required number of clusters times the average cluster size.

For this purpose, we assume that the natural fluctuation in cluster mean weight change (i.e., the standard deviation in mean weight change among clusters) is 3.5%, i.e., half the target weight reduction of 7%. This assumption is based on (or rather inspired by) variation in BMI between successive waves (approximately 5 years apart: much longer than our follow-up period) observed in the Framingham Study [29] where the standard deviation in individual weight differences was between 6% and 7%.

We will assume that we compare the two groups using an unpaired t-test.

For a two-armed parallel cluster randomized design to achieve a power of 80% and a one-sided significance level of 5%, we would need four clusters in each trial arm, i.e., a total of eight clusters [32] Since the stepped-wedge cluster design that we have selected is more efficient than a parallel design [33], this sample size should be adequate given our assumption. However, to accommodate for unduly optimistic assumptions about the standard deviation in weight changes we will increase our total sample size to 10 clusters.

An interim analysis will be performed once four clusters have completed the intervention and the required sample size will be re-calculated on the basis of observed fluctuations in parameters; if this leads to the conclusion that more than 10 clusters are required to detect a statistical significance in the primary outcome, the team will meet to discuss how and if the sample of clusters can be extended.

#### Recruitment

Participants will be recruited from Kanad Hospital based on the eligibility criteria and willingness to give consent. Screening and recruitment will continue until the required sample size is reached or the grant expires, whichever comes first. Participants will be allowed to keep the activity tracker at the conclusion of the intervention.

#### Allocation

Due to the nature of a family-based intervention, it is not practically possible to compare family units undergoing the intervention directly with family units undergoing the usual care of receiving individual visits with the dietician and obesity clinic team. In addition, variation in the size of the family units makes a matched family-to-family allocation impractical. Therefore, it was decided to pursue a strategy of a cluster randomized stepped-wedge trial.

#### Blinding

Due to the nature of the intervention, it is not possible to blind the participants.

#### Data collection

Weight, blood pressure, waist circumference, hemoglobin A1c and lipid parameters will be measured at enrollment (baseline) and after the study intervention. Each study participant will also have access to a scale within the home for weekly weight measurements. For blood pressure, we will follow the American Heart Association (AHA) guidelines of 2019 on proper measurement of blood pressure in humans [34]. This includes proper selection of cuff size, appropriate body position of the participant, proper measuring technique, and accurate blood pressure reading. For weight and height measurements, we will follow the *Good Practice Guidelines* as described by the National Nurses Nutrition Group and the Centers for Disease Control and Prevention (CDC) guidelines, respectively [35, 36]. All devices undergo routine calibration according to Joint Commission International standards.

In cases of study discontinuation or withdrawal, the data will be analyzed based on an intention-to-treat basis.

#### Data management

The data of interest will be extracted and double entered into a password-protected spreadsheet with a separate password protection for logging in to the laptop itself by the health coach and principal investigator (JK). Each data element will be verified for accuracy and format. This spreadsheet will have participants’ data organized by their study number, not their clinic medical record number. A separate spreadsheet with a third password will contain only participants’ study numbers and clinic medical record numbers; this separation of the clinic medical record numbers from the actual data will de-identify the data from HIPAA-defined personal medical information.

The passwords will be changed on a regular basis. A complete back up of the data will be performed twice a month to an external drive, which will be stored in a separate location under climate-controlled conditions and will be retained indefinitely.

#### Missing data

As noted above, in cases of study discontinuation or withdrawal, the data for the intervention participants will be classified under intention-to-treat.

#### Data monitoring

Data will be reported to the Kanad Hospital Research Ethics Committee (KHREC), which is independent of the study organizers and the United Arab Emirates University. This committee is responsible for establishing the ethical nature of all research involving human subjects within the hospital, monitoring the progress of each approved study, and stopping any study that has concerns regarding ethical matters or participant safety. Due to the anticipated benign nature of the intervention and the stringent oversight supplied by the Research Ethics Committee, a dedicated Data Monitoring Committee is not needed for this study.

#### Harms

Since this is a non-pharmacological study, no significant risk of harm is anticipated. Each study participant will continue under the usual care of their physicians.

An adverse event, for the purposes of the study, will be defined as any potential or actual negative occurrence with respect to the participant’s health. The most likely adverse event would be hypoglycemia in those participants who may be taking certain medications for diabetes. The potential for adverse effects is highlighted in the informed consent form, along with the contact information for the principal investigator (JK).

The health coach has been made aware of common potential adverse events, such as hypoglycemia, and which medications place participants at risk. The health coach will report any adverse event to the principal investigator and encourage the participant to follow up with their physician.

Any severe event or events that cannot be attributed to other causes other than the study intervention will be reported to the Ethics Committee within 24 hours.

#### Auditing

The PI (JK) will meet with the health coach regularly to review the data of each participant. The health coach will coordinate planned clinic visits for repeat measurements of biometrics and labs and notify the PI (JK) who will then request and record the appropriate data.

One of the co-investigators will serve as the monitor (RDG) and will confirm that all associated documents are kept up-to-date in a regulatory binder. This collection will include hard copies of the study protocol and informed consent forms in English and Arabic along with any revised versions, ethics committee approvals of the previously mentioned documents, ethics committee correspondence, and any adverse effect reports. Independent monitoring is not required due to the lack of conflict of interest and the non-pharmacologic nature of the intervention.

#### Ethics

*Research ethics approval*. This protocol has been approved by KHREC with respect to scientific content and compliance with local regulations concerning research in human subjects.

The ethics committee has requested at least annual review of the protocol and data, which will include study enrollment census, biometric and lab data, and any reports of adverse effects. The ethics committee will be notified of any severe adverse effects within 24 hours.

#### Protocol amendments

Any changes to the protocol which may affect the conduct of the study, decrease potential benefits or increase risk of harm to the participants will be communicated to the ethics committee for review and approval. On approval of these changes, these changes will then be communicated to the participants and a revised informed consent signed. If the participant refuses to sign the revised consent form, they will be removed from the trial.

Minor modifications that do not meet the above criteria will be communicated to the ethics committee and patients at the discretion of the investigational team.

#### Consent

Eligible individuals will read and sign the informed consent either in English or Arabic. Any questions or concerns will be addressed by one of the investigators. If there is any concern on the investigators’ part regarding the capacity of that individual to give consent, that person will be excluded from the study. Biologic samples will not be retained for future studies.

#### Confidentiality

The biometric and lab data will be stored as usual in the participants’ electronic medical record, which is access restricted and safeguarded by the information technology team of Kanad Hospital. The data of interest will be extracted and recorded as discussed in the Data Management section above. Participants’ data will not be released outside of the study without individual written permission, except as requested for monitoring purposes by KHREC or as legally required by local or national government authorities.

#### Access to data

Only those investigators who perform clinical duties at the involved hospital will have access to the original biometric and lab data via the participants’ electronic medical record. Once the data has been de-identified, all investigators will have access to de-identified biometric and lab data. The health coach and investigators will have access to the data supplied by each participant on a weekly basis, which includes but is not limited to weight, caloric intake, minutes and type of exercise, and correspondence. This access will be discontinued after the aggregate data has been analyzed and published, other than what is required for ongoing patient care.

#### Ancillary and post-trial care

All Emiratis who are eligible for this study have comprehensive health insurance which will cover, at no cost to the participant, any necessary medical care. No additional compensation is anticipated or required due to the benign nature of the intervention.

Should this study provide evidence of a clinically significant benefit to those who underwent the study intervention, the age and sex matched controls will be contacted at the conclusion of the study and invited to receive the same intervention at the discretion of Kanad Hospital.

### Dissemination policy

#### Trial results

The investigators will review the results, once available. After consensus as to whether the results support the hypothesis, the manuscript will be written. Whether or not a clinically significant result is found, the results will be disseminated via journal publication or trial registry. Funding supporting the study from United Arab Emirates University is an unrestricted grant, so no publication restrictions are anticipated.

#### Authorship

A writing committee will write the manuscript at the direction of the principal investigator. Consensus of all investigators will be required prior to submission of the manuscript for publication or in the trial registry. All authors of any submitted manuscript will meet the authorship criteria as set forth by The International Committee of Medical Journal Editors.

#### Reproducible research

Within 3 years after the collection of the data, a completely de-identified data set will be made available to an appropriate data archive to support ongoing research efforts.

## Discussion

Although previous surveys and reported data have defined the scope of the problem of deaths related to obesity and CVD in the UAE and have suggested possible underlying lifestyle-related etiologies, this would be, to our knowledge, the first prospective interventional trial utilizing the strength of family relationships to affect positive lifestyle change.

There is a lack of prospective studies in our setting, which is traceable, at least in part, to the difficulties in conducting robust studies. Previous studies have fallen victim to higher-than-expected rates of drop out and loss to follow up, funding for studies is difficult to obtain and utilize due to multiple organizational restrictions and limitations, and privacy and ethical concerns inhibit the participation and of, and collaboration between, larger organizations within the health system.

In an ideal scenario, this vital research question would be investigated using a cluster randomized control trial with family clusters matched for the presence of confounding factors such as age, obesity, presence and severity of diabetes, hypertension, and hyperlipidemia. It would be a multicentric trial that applied the intervention simultaneously for all family clusters to avoid the confounding effects of time and contamination. In addition to the previously mentioned restrictions, our specific limitations include insufficient funding to utilize more than one health coach, a grant period of 2–3 years, and limited administrative support.

The EHHP team decided that despite these limitations, the study should proceed with the best possible protocol under these conditions. Therefore, a pragmatic study design utilizing a stepped wedge of clusters was selected to mitigate these restrictions. The stepped nature of the intervention allows statistical analysis (using mixed models) that utilize both differences in weight and other target parameters between time periods before and after intervention, and differences between clusters (comparing clusters under intervention with those not yet having received the intervention). This allows an efficient estimation of the impact of the intervention on target parameters and to test whether this impact is statistically significant. This may inform and benefit follow on studies.

The study focuses on the primary outcome of weight as well as secondary outcomes which consist of simple measures of CVD risk. Despite the importance of additional data, the team lacks the resources to obtain intake of whole grains, ultra-processed foods, and red meat. As the primary outcome in this study is change in weight, which is most directly correlated to change in caloric intake and expenditure, we have prioritized accurate recording of caloric intake accordingly. Unfortunately, our grant does not allow us to bring a dietician onto our team. The software that comes with the fitness tracker allows the participant to enter their dietary intake into their smartphone, but it excludes commonly eaten foods in our setting and is not available in the Arabic language at this time. Due to the anticipated amount of missing data, we have decided to exclude a diet diary in this iteration of the study despite the richness of information that could be collected. The investigators have elected not to run multiple clusters simultaneously, allowing the health coach to be focused on one to two families at a time. Although we are studying family clusters, these clusters represent extended families that may be spread across a geographic area of several hundred square kilometers; the afternoons after Friday prayers are the optimal time to gather and address the extended family as a group. This restricts the time available for group sessions to a few hours a week, and thus limits the number of clusters a single health coach can effectively manage. This opens up the possibility of changes over the course of the study in the clusters that are randomized to later intervention episodes; their willingness to participate may have waned, leading to increased losses, or the families may have chosen other approaches for weight loss, leading to contamination.

An additional limitation that stems from our inability to fund multiple health coaches is that any result becomes difficult to generalize to approaches using multiple health coaches or infer how such coaches should be selected and trained. This makes the external validity of our findings uncertain and would require larger, better resourced studies to investigate.

Smoking is a potent cardiovascular risk factor, and any complete package of interventions aiming at cardiovascular risk reduction must address tobacco use. However, the focus of the DPP and our study are the reduction in weight through diet and physical activity modification. The DPP curriculum, which we have adapted, does not include much emphasis on smoking cessation. Since our secondary outcomes are limited to the metabolic risk factors and do not explicitly address the 10-year cardiovascular risk score for which smoking cessation or continuation would influence the level of risk, smoking will not be a major confounding factor in our outcomes. Additionally, a limitation of our study design that affects our ability to follow and assess cardiovascular outcomes in our participants is the lack of an enduring usual care arm. As stated earlier, the cluster wedge design was selected due to our limited resources and, through statistical analysis, allows us to compare participants’ risk factors after intervention with the group’s pre-intervention risk factors. If our intervention results in the anticipated clinically significant reduction in risk, a larger study could be enrolled similar to the DPP and DPPOS studies. This future study could also assess the portion of risk attributable to tobacco use.

The EHHP team recognizes the desire of the leadership of the UAE to back research that contributes to the health and well-being of the nation. The citizens of the UAE are also highly interconnected and word of new developments travels quickly. A clinical benefit, whether statistically significant or merely perceived by a proportion of the participants, could lead to support for a larger, more comprehensive, and more rigorous study in the future. Despite the study limitations described previously, this would be well worth the time and resources that have been invested in this study.

## Supporting information

S1 Appendix SPIRIT (Standard Protocol Items: Recommendations for Interventional Trials) checklist. (DOC)

S2 Appendix TIDieR (Template for intervention description and replication) framework and checklists. (DOCX)

S3 Appendix Informed consent, English. (DOCX)

S4 Appendix Informed consent, Arabic. (DOCX)

S5 Appendix Kanad hospital ethics application. (PDF)

S6 Appendix EHHP Session 1 English. (DOCX)

S7 Appendix EHHP Session 1 Arabic. (DOCX)

S8 Appendix EHHP Session 2 English. (DOCX)

S9 Appendix EHHP Session 2 Arabic. (DOCX)

S10 Appendix EHHP Session 3 English. (DOCX)

S11 Appendix EHHP Session 3 Arabic. (DOCX)

S12 Appendix EHHP Session 4 English. (DOCX)

S13 Appendix EHHP Session 4 Arabic. (DOCX)

S14 Appendix EHHP Session 5 English. (DOCX)

S15 Appendix EHHP Session 5 Arabic. (DOC)

S16 Appendix EHHP Session 6 English. (DOCX)

S17 Appendix EHHP Session 6 Arabic. (DOCX)

S18 Appendix EHHP Session 7 English. (DOCX)

S19 Appendix EHHP Session 7 Arabic. (DOCX)

S20 Appendix EHHP Session 8 English. (DOCX)

S21 Appendix EHHP Session 8 Arabic. (DOCX)

S22 Appendix EHHP Session 9 Arabic. (DOCX)

S23 Appendix EHHP Session 10 English. (DOCX)

S24 Appendix EHHP Session 10 Arabic. (DOCX)

S25 Appendix EHHP Session 11 English. (DOCX)

S26 Appendix EHHP Session 11 Arabic. (DOCX)

S27 Appendix EHHP Session 12 English. (DOCX)

S28 Appendix EHHP Session 12 Arabic. (DOCX)

S29 Appendix EHHP Session 13 English. (DOCX)

S30 Appendix EHHP Session 13 Arabic. (DOCX)

S31 Appendix EHHP Session 14 English. (DOCX)

S32 Appendix EHHP Session 15 English. (DOCX)

S33 Appendix EHHP Session 15 Arabic. (DOC)

S34 Appendix EHHP Session 16 English. (DOCX)

S35 Appendix EHHP Session 16 Arabic. (DOC)

S36 Appendix EHHP Table 2. (DOCX)
