# Correction: Emirates Heart Health Project (EHHP): A protocol for a stepped-wedge family-cluster randomized-controlled trial of a health-coach guided diet and exercise intervention to reduce weight and cardiovascular risk in overweight and obese UAE nationals

**DOI:** 10.1371/journal.pone.0315685

**Published:** 2024-12-09

**Authors:** The PLOS ONE Staff

There are errors in the author affiliations. The correct affiliations are as follows:

Jeffrey K. King^1,7^, Mohamud Sheek-Hussein^2,8^, Nico J. D. Nagelkerke^2^, Alexander Kieu^1,3^, Saif Al-Shamsi^4^, Javaid Nauman^2^, Nicholas Hoque^3,5,6^, Romona D. Govender^1^, Iffat ElBarazi^2^, Kristoffer Crawford^3^

**1** Department of Family Medicine, College of Medicine and Health Sciences, United Arab Emirates University, Al Ain, Abu Dhabi, United Arab Emirates, **2** Institute of Public Health, College of Medicine and Health Sciences, United Arab Emirates University, Al Ain, Abu Dhabi, United Arab Emirates, **3** Kanad Hospital, Al Ain, Abu Dhabi, United Arab Emirates, **4** Department of Internal Medicine, College of Medicine and Health Sciences, United Arab Emirates University, Al Ain, Abu Dhabi, United Arab Emirates, **5** Department of Pediatrics, College of Medicine and Health Sciences, United Arab Emirates University, Al Ain, Abu Dhabi, United Arab Emirates, **6** Department of Bioengineering, Imperial College London, London, United Kingdom, **7** Home Based Primary Care, Division of Geriatrics and Extended Care, Greater Los Angeles, Department of Veterans Affairs, Los Angeles, California, United States of America, **8** School of Public Health, Loma Linda University, Loma Linda, California, USA.

The publisher apologizes for the error.
