# Supplementary material for: Emirates Heart Health Project (EHHP): A protocol for a stepped-wedge family-cluster randomized-controlled trial of a health-coach guided diet and exercise intervention to reduce weight and cardiovascular risk in overweight and obese UAE nationals
Source: PLoS One. 2023 Apr 10;18(4):e0282502. doi: 10.1371/journal.pone.0282502 (PMC10085020; doi:10.1371/journal.pone.0282502)
Supplement: S3 Appendix — (DOCX) [file pone.0282502.s003.docx]

CONSENT FORM

Centre number:

Study number:

Patient ID number used in the study:

Title of project: The Emirates Heart Health Project Study (EHHP)

Names of researchers:

Dr. Jeffrey King

Dr. Mohamud Sheek Hussein

1 I confirm that I have read and understand the information sheet dated October 8, 2019 (Version 1) for the above study and have had the opportunity to ask questions.

2 I understand that my participants is voluntary and that I am free to withdraw at any time.

3 I understand that if I withdraw from the study it will not adversely affect my healthcare or employment.

4 I understand that my data will be kept confidential and in a safe place.

5 I agree to take part in the above study.

Name of patient Date Signature

Name of person taking consent Date Signature

Name of witness (if subject unable to read or write)

Date Signature

*Patient information sheets*

Study title: The Emirates Heart Health Project (EHHP)

You are being invited to take part in a research study. Before you decide whether to participate, it is important for you to understand why the research is being done and what it will involve. Please take the time to read the following information carefully and discuss it with others if you wish. Ask us if there is anything that is not clear or if you would like more information. Please take time to decide whether or not you wish to take part.

Thank you for reading this.

In the UAE a major health problem is heart disease. Heart disease can be caused by problems with blood pressure, diabetes, and cholesterol. We designed the EHHP program to decrease blood pressure, blood sugar and cholesterol levels in the blood, and this study is to see if the program is effective. This study will take 3 months to complete.

We are looking for Emiratis above the age of 18 years to take part in this study. We need 8 Emirati families in order to complete the study.

It is up to you to decide whether or not to take part in this study. If you do decide to take part you will be given this information sheet to keep, and to sign a consent form. If you decide to take part you are still free to withdraw at any time and without giving a reason. A decision to withdraw at any time, or a decision not to take part, will not affect the care you receive.

If you take part, your blood pressure, weight, height and heart rate will be measured at the beginning. Blood will be drawn to measure your cholesterol and sugar levels. You will fill out a paper that asks about what you eat, and how much physical activity you do each week. The families will take turn going through 16 week-long program. You will need to attend a time with people from your family where you watch a video, have a demonstration of healthy cooking or physical activity that we want you to try, and a discussion time. At the end of the study, we will repeat your blood pressure, weight, and heart rate and blood will be drawn again to measure your cholesterol and sugar levels to see if the EHHP program had any benefit.

If you take medications prescribed by your doctor, these medications should be continued. If the EHHP program has benefit, the doses of these medications may need to be adjusted.

The EHHP program is a combination of a healthy diet and more physical activity. You do not need to restrict the overall number of calories you eat, but the program encourages you to eat more plant based whole foods. It also encourages you to be more physically active, primarily through walking.

If this diet is different from your usual diet, you may experience a short period of time where your digestion may be different, resulting in abdominal gas or changes in your bowel movements, but this should resolve within a week. If you are taking medications for blood pressure or diabetes and you notice weakness, dizziness, sweating or shaking of the hands, you should seek medical attention.

Since this is a study of a natural intervention, there should be no risks in taking part in this study. Many people have been studied on similar programs all over the world without any significant problems. Many of the people that have participated in similar programs with previous studies have had improvements in their weight, blood pressure, cholesterol and sugar levels. We hope that this program will have the same benefits for you, however, this cannot be guaranteed. The information we get from this study may help the UAE to decrease the risk of developing heart disease.

Sometimes during the course of a research project, new information becomes available about the treatment that is being studied. If this happens, your research doctor will tell you about it and discuss with you whether or not you want to continue in the study. If you decide to withdraw, your research doctor will make arrangements for your care to continue. If you decide to continue in the study, you will be asked to sign an updated consent form.

Once the research study stops, you will be notified of your test results. If you have an improvement in your results, you are of course free to continue the diet and physical activity program that you have participated in.

If you have any problems or complaints about the study, please contact Dr. Jeffrey King at [jking@uaeu.ac.ae](mailto:jking@uaeu.ac.ae).

All information which is collected about you during the course of the research will be kept strictly confidential. Any information about you which leaves the university will have your name and address removed so that you cannot be recognized from it.

The results of this study will be submitted to a journal for other health care professionals to read. You will not be identified as having taken part in this study. If you would like a copy of the study, you can request a copy from Dr. Jeffrey at the above email address.

This study is being funded by a grant from the United Arab Emirates University (UAEU) research fund. The research team all work for UAEU and will not receive any additional compensation for running the study. (text removed)

This study has been approved by the Kanad Hospital Human Research Ethics Committee (KH-HREC).

Thank you for considering participating in this study.
