# Supplementary material for: Emirates Heart Health Project (EHHP): A protocol for a stepped-wedge family-cluster randomized-controlled trial of a health-coach guided diet and exercise intervention to reduce weight and cardiovascular risk in overweight and obese UAE nationals
Source: PLoS One. 2023 Apr 10;18(4):e0282502. doi: 10.1371/journal.pone.0282502 (PMC10085020; doi:10.1371/journal.pone.0282502)
Supplement: S4 Appendix — (DOCX) [file pone.0282502.s004.docx]

**نموذج الموافقة**

**رقم المركز:**

**رقم الدراسة:**

**رقم هوية المريض المستخدمة في الدراسة:**

**عنوان المشروع: دراسة تعزيز صحة القلب في الإمارات**

**أسماء الباحثين:**

**الدكتور:- جيفري كينج**

**الدكتور:- محمود الشيخ حسين**

1- أؤكد أنني قد قرأت وفهمت ورقة المعلومات المكتوبة بتاريخ 8 أكتوبر 2019 (الإصدار رقم 1) للدراسة المذكورة أعلاه وأتيحت لي الفرصة لطرح الأسئلة..

2. أدرك أن مشاركتي طوعية وأن لدي حرية الانسحاب في أي وقت.

3. أدرك أنه إذا انسحبت من الدراسة ، فلن يؤثر ذلك سلبًا على رعايتي الصحية أو وظيفتي.

4. أدرك أن بياناتي ستبقى سرية وفي مكان آمن.

أوافق على المشاركة في الدراسة المذكورة أعلاه.

اسم المريض التاريخ التوقيع

اسم الشخص الذي يأخذ الموافقة التاريخ التوقيع

اسم الشاهد (إذا كان الشخص غير قادر على القراءة أو الكتابة)

التاريخ التوقيع

ورقة معلومات المريض

عنوان الدراسة: دراسة تعزيز صحة القلب في الإمارات

أنت مدعو للمشاركة في دراسة بحثية.,ولكن قبل أن تقرر المشاركة ، من المهم أن تفهم سبب إجراء هذه الدراسة وما الذي سيتضمنه. يرجى أخذ الوقت الكافي لقراءة المعلومات التالية بعناية ومناقشتها مع الآخرين إذا كنت ترغب في ذلك.بامكانك أن تطرح علينا الأسئلة إذا كان هناك أي شيء غير واضح أو إذا كنت بحاحة لمعرفة مزيد من المعلومات.يمكنك أخذ بعض الوقت لتقرير ما إذا كنت ترغب في المشاركة أم لا.

شكرا لقراءتك هذا.

من أكبر المشاكل الصحية بدولة الامارات هي مشاكل القلب. يمكن أن يكون سبب أمراض القلب مشاكل في ضغط الدم والسكري والكوليسترول. لقد قمنا بتصميم برنامج دراسة صحة القلب في الامارات لخفض مستوى ضغط الدم ومستويات السكر و الكوليسترول في الدم ، وهذه الدراسة لمعرفة ما إذا كان البرنامج فعالاً. ستستغرق هذه الدراسة 16 أسبوع

نحن نتطلع لمشاركة الإماراتيين فوق سن 18 عامًا ليكونوا جزءا في هذه الدراسة. نحتاج إلى ٨ عائلات إماراتية لاستكمال الدراسة.

لك الحق في أن تقرر ما إذا كنت ستشارك في هذه الدراسة أم لا.في حالة الموافقة ، ستحصل على نموذج من ورقة المعلومات هذه لتحتفظ بها ، القيام بالتوقيع على نموذج الموافقة. إذا قررت المشاركة ،لك الحرية بالانسحاب في أي وقت ودون إبداء اي سبب. علما بانه في حال انسحابك أو عدم مشاركتك لن يؤثر ذلك على الرعاية التي تتلقاها.

في حال اشتراكك سنقوم في البداية بقياس ضغط الدم والوزن والطول ومعدل ضربات القلب.و سيتم أخذ عينة من الدم لقياس مستويات الكوليسترول والسكرفي الدم. سوف تقوم بتعبئة ورقة تحتوي على أسئلة عن الوجبات التي تتناولها ومقدار النشاط البدني الذي تقوم به كل أسبوع. سوف تكون معنا العائلات في برنامج طويل لمدة ١٦ أسبوعا. يجب عليك الحضور مع أفراد من عائلتك لمشاهدة مقطع فيديو يوضح طرق الطهي الصحي أو الأنشطة البدنية التي نود منك تجربتها ونقوم بمناقشة الفيديو معك. في نهاية الدراسة ، سنعيد قياس ضغط الدم والوزن ومعدل ضربات القلب وسيتم سحب الدم مرة أخرى لقياس مستويات الكوليسترول والسكر لمعرفة ما إذا كان برنامج دراسة صحة القلب مفيد.

اذا كنت تتناول أدوية موصوفة لك من قبل طبيبك ، يجب أن تستمر في تناول هذه الأدوية. إذا عاد عليك برنامج صحة القلب بالنفع والفائدة ربما تحتاج لتعديل جرعات الدواء

برنامج صحة القلب هو مزيج من النظام الغذائي الصحي و النشاط البدني. لا تحتاج إلى تقييد أو تقليل إجمالي للسعرات الحرارية التي تتناولها ، لكن البرنامج يشجعك على تناول المزيد من الأطعمةالنباتية. كما يشجعك على زيادة النشاط البدني من خلال المشي بشكل أساسي.

إذا كان هذا النظام الغذائي مختلفًا عن نظامك الغذائي المعتاد ، فقد تتعرض خلال مدة زمنية قصيرة لاختلاف في الهضم ، مما ينتج عنه تشكل غازات في البطن أو تغيرات في حركات الأمعاء لديك ، ولكنها تحل في غضون أسبوع. إذا كنت تتناول أدوية لضغط الدم أو السكري ولاحظت ضعف عام أو دوخة أو تعرق أو رجفة في اليدين ، فعليك طلب العناية الطبية.

نظرًا لأن هذه دراسة للتدخل الطبيعي ، فلا ينبغي أن تكون هناك مخاطر في المشاركة في هذه الدراسة. تمت دراسة العديد من الأشخاص على برامج مماثلة في جميع أنحاء العالم دون أي مشاكل كبيرة. العديد من الأشخاص الذين شاركوا في برامج مماثلة مع دراسات سابقة قد تحسنت أوزانهم و مستويات ضغط الدم والكوليسترول ومستويات السكر. نأمل أن يكون لهذا البرنامج نفس الفوائد بالنسبة لك ، ومع ذلك ، لا يمكن ضمان ذلك. المعلومات التي نحصل عليها من هذه الدراسة قد تساعد دولة الإمارات العربية المتحدة على تقليل خطر الإصابة بأمراض القلب.

في بعض الأحيان أثناء مشروع بحثي ، يمكن أن تتوفر معلومات جديدة حول العلاج الذي تتم دراسته. إذا حدث ذلك ، فسوف يخبرك طبيبك عن ذلك ويناقش معك ما إذا كنت ترغب في الاستمرار في الدراسة أم لا. إذا قررت الانسحاب ، فسيقوم طبيبك بإجراء الترتيبات اللازمة لمواصلة رعايتك. إذا قررت الاستمرار في الدراسة ، فسيُطلب منك التوقيع على نموذج موافقة محدث.

بمجرد توقف الدراسة البحثية ، سيتم إخطارك بنتائج الاختبار. إذا كان لديك تحسن في نتائجك ، فأنت بالطبع لديك الحرية في متابعة برنامج النظام الغذائي والنشاط البدني الذي شاركت فيه.

إذا كانت لديك أي مشاكل أو شكاوى حول الدراسة ، فيرجى التواصل مع الدكتور جيفري كينج على jking@uaeu.ac.ae

سيتم الاحتفاظ بسرية تامة على جميع المعلومات التي يتم جمعها عنك أثناء البحث . أي معلومات عنك خارج الجامعة سيتم حذف اسمك وعنوانك بحيث لا يمكن التعرف عليك.

سيتم تقديم نتائج هذه الدراسة إلى مجلة لقرائتها من قبل محترفي الرعاية الصحية الآخرين. لن يتم تعريفك على أنك شاركت في هذه الدراسة. إذا كنت ترغب في الحصول على نسخة من الدراسة ، يمكنك طلب نسخة من الدكتور جيفري على عنوان البريد الإلكتروني أعلاه.

تمول هذه الدراسة بمنحة من صندوق البحوث بجامعة الإمارات العربية المتحدة. يعمل فريق البحث في جامعة الإمارات العربية المتحدة ولن يحصل على أي تعويض إضافي لإجراء الدراسة.

تمت الموافثة على الدراسة من قبل لجنة أخلاقيات البحث الإنساني في مستشفى كند

شكرا لك على المشاركة في هذه الدراسة
