# Supplementary material for: Emirates Heart Health Project (EHHP): A protocol for a stepped-wedge family-cluster randomized-controlled trial of a health-coach guided diet and exercise intervention to reduce weight and cardiovascular risk in overweight and obese UAE nationals
Source: PLoS One. 2023 Apr 10;18(4):e0282502. doi: 10.1371/journal.pone.0282502 (PMC10085020; doi:10.1371/journal.pone.0282502)
Supplement: S6 Appendix — (DOCX) [file pone.0282502.s006.docx]

**Session 1: Welcome**

**Materials:**

- Handouts
- Weight chart
- Food and Activity tracker
- Scales
- Name tags with Sharpie marker
- Whiteboard with markers and eraser

**Before you begin:**

- Create a meeting schedule. Include your name, phone number and email.
- View the video.
- Review the objectives/goals of the session.
- Review the classroom presentation.

**Objectives/goals:**

By the time session 1 is finished, the participants will:

- Be able to explain in their own words the purpose and benefits of the Emirates Heart Health Program.
- Be able to describe the basic structure of each session.
- Know the weight loss and physical activity goals of the program.
- Know their personal weight loss and physical activity goals.
- Be able to explain how self-monitoring helps with behavior change.

**Overview:**

*Part 1: Introduction (10 minutes)*

Introduce yourself and ask the participants to introduce themselves.

Name

Reason for participating in the program.

*Part 2: The Emirates Heart Health Program (10 minutes)*

View the video together of Dr. Durra speaking about the research study with its goals, protocol and desired outcomes.

*Part 3: What to expect (20 minutes)*

This section covers how sessions are structured and what is expected of the Lifestyle Coach and the participants.

*Part 4: Getting started losing weight (20 minutes)*

View the video together of Dr. Latifa explaining the DASH diet.

Teach the participants how to track their food intake. They will record everything they eat or drink for the next week.

*Part 5: Wrap up and To-do list (5 minutes)*

Summarize what was covered and discuss what the participants should do until the next session.

**Key messages:**

- **The purpose of the EHHP is to reduce the risk of heart disease by reducing body weight, cholesterol, blood pressure and blood sugar.**
- **The amount of weight loss needed to reduce risk may be less than participants hope for.**
- **Goals will be pursued gradually and safely.**
- **The family, Lifestyle Coach and each participant will work together as a team.**
- **Self-monitoring of food intake is important to reach the goal.**

**Classroom presentation**

*Part 1: Introduction (10 minutes)*

Welcome the participants.

Say: You are here as part of the Emirates Heart Health Program to see if these lifestyle changes will help reduce your risk for heart disease. This is a big problem in this country, and the project, if it is successful, could help the people of this country with part of the solution to this big problem. We will work together as a team during the next 16 weeks to become healthier by changing our diet and by being physically active.

During this first meeting, we will start to get to know each other, talk about how the program works, and what our responsibilities will be moving forward.

**Introductions**

**Introduce yourself** as the lifestyle coach.

Name, how long you have been in in the UAE, how you got involved in EHHP.

What your role will be:

1. To help participants learn about healthier eating and physical activity
2. To help participants change their habits to reduce their risk for heart disease.
3. To show participants examples of healthy eating and techniques for physical activity that can be personalized for each individual.
4. To help participants track their food intake and minutes and type of physical activity.
5. To help the group to problem solve together any challenges that make achieving the goal more difficult; and to help the group learn from and support each other.

**Ask participants to introduce themselves.**

Name, the reason they decided to participate.

**Present:** This week we will:

- Go over the purpose and benefits of the program.
- Review what we will do during every session.
- Figure out your individual weight loss goals.
- Discuss why self-monitoring is important.

**Present:** Before we continue, let us go over some details. We will plan to meet at the same place and time every week. If we need to change this, we should discuss and decide as a group how to change this. If you miss a class, we can have a review session another time. Feel free to call or WhatsApp me if you have any questions or need any support. Please let me know the day before if you are going to miss a session.

**Ask:** Does anyone see any problems with the meeting schedule (holidays, travel)?

**Allow participants to respond.**

**Present:** Groups, especially families can be a powerful influence. However, they do the greatest good when certain guidelines are agreed to and followed.

**Ask:** What are some good rules for us to follow as a group over the next 16 meetings? (You may need to share an idea or two, such as talking to one another with respect).

**Write them on the whiteboard.**

**Tell** the group you will write a list of the guidelines they agreed to and will send it by WhatsApp.

Make sure that you cover the ground rules in a positive way and get the participants to agree to them.

*Part 2: The EHHP (10 minutes)*

**Present:** Now let us talk about what this program is about. This is a short video where Dr. Durra Al Baloushi will explain what the program is and why we think it is important.

**Refer** the patients to the Program Meeting Schedule handout.

**Present**: This program has 16 sessions that will be take place during the next 16-20 weeks. It is divided into three sections: Getting started which covers the basics of healthy eating and physical activity, Understanding what shapes our eating and activity behaviors which covers how to take control of our eating and physical activity and Long term change. Change is difficult and takes time. We want to see healthy changes in your life for the rest of your life. This last section will involve:

Problem solving

Avoiding tempting situations

Healthy eating when not at home

Managing stress

Staying motivated.

**Present**: The EHHP has two goals: weight loss (about 7% of your current body weight) and regular physical activity. We would like to see you doing 150 minutes of activity each week by the end of the program. We will talk about physical activity in a later session.

**Present**: We want to achieve these goals gradually and safely. And we will all work together to help each other reach our goals of losing weight by eating healthier and being more active.

**Ask:** How do you think you will benefit from reaching your goals?

**Allow a few participants to respond.**

*Part 3: What to expect (20 minutes)*

**What is required of participants:**

**Present**: We have a lot to do together in the next few months. You will be asked to make some changes in your life, and if you do it, we think you will feel better and be healthier. Many people all over the world have done this, and you can do it too. You will not be alone. Your family will do this with you, and I will be here to help you through the process. But I cannot do this without your commitment.

**Present:** For you to get the most out of this program, I need you to commit to coming to each session. I need you to record what foods and drinks you are taking. I need you to record how much physical activity. I need you to be honest and accurate in doing this.

Ask me questions, tell me your concerns. I can help you.

Don’t give up! Some days will be better than others, but if you give it your best effort, I know that you will succeed.

**What is required of the Lifestyle Coach:**

**Present:** You can count on me and the group to:

Notice what you are doing well and what can be improved.

Answer your questions.

Share solutions and ideas while we learn together.

Be honest.

Support you when you face challenges.

Believe that you can reach your goals.

**Ask:**

Is there anything else I can do for you?

I would now like you to sign the “We will work together” handout.

**Remember the purpose:**

**Ask:** Do you remember why we are here and what we are trying to do?

What are you excited about?

What are you not excited about?

Note: Watch for anxious participants. Some may now feel overwhelmed because they do not believe they can do this. Address concerns. Consider speaking with them privately.

*Part 4: Getting started losing weight (20 minutes)*

**Present:** Now you know what the program is about and how it is going to work. You have heard that:

Each week you will work toward your personal goals of losing weight through healthy eating and being more physically active.

You will receive the information and support you need to safely and gradually reach and maintain your goals. We do not want just a temporary change.

**Ask**: Before I go on, are there any questions about what we have discussed so far?

**Answer any questions.**

**Present:** We are now going to talk about how you can reach your goals.

The most important thing we can do is to eat healthier, and to reduce the number of calories we are eating. And the first step to eating healthier and fewer calories is to figure out what we are eating now. To do this, we are going to record every day everything that we eat and drink. We are going to continue this for the rest of our time together. Keeping track of what we do is the most important part of changing our habits. This will help us see:

What foods we eat.

How much we eat.

When and where we eat.

How our habits change over time.

How we can adjust what we eat to get a healthier, more balanced diet.

**Refer to the handout.**

**Present:**

During this program, I want you to record everything you eat and drink. You will start tomorrow morning. Here are some key ideas:

- Spelling is not important.
- Be honest. Write down every little thing you eat, even if it is very small.
- Be accurate. Write down what you eat as soon as you can so you don’t forget.
- Be complete. Include everything you eat or drink.
- Try your best to estimate the amount. We will work on this as the program goes on, and you will become better at it.
- Without writing things down, it is difficult to figure out an effective eating plan.

**Present**: Now let’s take a minute to practice this.

**Ask:** What did you eat for dinner last night, or earlier today?

Circle any of the foods that are unhealthy.

**Present**: Portion size is important. A small difference in portion size can make a big difference in the amount of sugar, fat and calories.

**Circle the room, and help participants when needed.**

*Part 5: Wrap up and the to-do list (5 minutes)*

**Distribute** the “Food and Activity Trackers” for next week.

**Present:** For the next week, write down everything you eat and drink, every day in your Tracker.

Write down: What you ate, How much you ate, When you ate it.

Circle the foods that you think are highest in calories.

Start to estimate the amount of food you eat.

**Summarize key points:**

 Record personal goals and expected outcomes; “What do I want to see happen because of this?”

 Teach self-monitoring of food intake using the plate method.

 During this program you will have my full support. We will do this together.

 We need you. You need to be committed to yourself and to the others.

**Present:** We have come to the end of this session. Are there any questions or concerns about anything we have discussed today?

**Answer any questions or concerns.**

**Present:** Next week we will take a closer look at which foods are adding fat, sugar and calories to our diet. I look forward to seeing you next week!

**Dismiss the group.**
