# Supplementary material for: Emirates Heart Health Project (EHHP): A protocol for a stepped-wedge family-cluster randomized-controlled trial of a health-coach guided diet and exercise intervention to reduce weight and cardiovascular risk in overweight and obese UAE nationals
Source: PLoS One. 2023 Apr 10;18(4):e0282502. doi: 10.1371/journal.pone.0282502 (PMC10085020; doi:10.1371/journal.pone.0282502)
Supplement: S7 Appendix — (DOCX) [file pone.0282502.s007.docx]

**الجلسة رقم 1: مرحبًا**

**الأهداف:**

عند انتهاء الجلسة الأولى، سوف يتمكن المشارك من:

- أن يكون قادرًا على شرح الغرض والفوائد من برنامج صحة القلب الإماراتي باستخدام طريقته الخاصة في التعبير.
- أن يكون قادرًا على وصف الهيكل القاعدة الأساسي لكل جلسة.
- التعرف على أهداف فقدان الوزن والنشاط البدني للبرنامج.
- التعرف على أهداف فقدان الوزن النشاط البدني الشخصية لكل مشترك .
- أن يكون قادرًا على شرح كيف تساعد المراقبة الذاتية في تغيير السلوك.

**المواد:**

- نشرات توزيعية
- مخطط الوزن
- أداة لتعقب ومراقبة الغذاء والنشاط الرياضي
- ميزان
- بطاقة للاسم مع قلم
- سبورة بيضاء مع اقلام وممحاة

**قبل ان تبدأ:**

- قم بإنشاء جدول اجتماع. قم بتضمين اسمك ورقم هاتفك والبريد الإلكتروني.
- اعرض الفيديو.
- قم بمراجعة أهداف الجلسة.
- قم بمراجعة العرض التقديمي

**نظرة عامة:**

الجزء الاول : المقدمة (10 دقائق)

قدم نفسك واطلب من المشاركين تقديم أنفسهم.

الاسم

سبب المشاركة في البرنامج

الجزء الثاني: برنامج صحة قلب الإمارات (10 دقائق)

شاهد الفيديو مع الدكتورة درة التي سوف تتحدث عن الدراسة البحثية مع أهدافها والنتائج المرجوة منها.

الجزء 3: ماذا تتوقع (20 دقيقة)

يغطي هذا القسم كيفية تنظيم الجلسات وما هو متوقع من مدرب نمط الحياة والمشاركين.

الجزء 4: البدء في فقدان الوزن (20 دقيقة)

شاهد الفيديو مع د. لطيفة تشرح حمية داش لخفض ضغط الدم.

علّم المشاركين كيفية تسجيل الأطعمة المتناولة . سوف يسجلون كل ما يأكلونه أو يشربونه للأسبوع المقبل.

الجزء 5: الخاتمة وقائمة المهام التي يجب تنفيذها (5 دقائق)

لخص ما تمت تغطيته وناقش ما يجب على المشاركين فعله حتى الجلسة التالية.

**الرسائل الرئيسية:**

- **الغرض من دراسة صحة القلب في الامارات هو تقليل خطر الإصابة بأمراض القلب عن طريق خفض وزن الجسم والكوليسترول وضغط الدم والسكر في الدم.**
- **قد تكون كمية فقدان الوزن اللازمة لتقليل المخاطر أقل مما يرجو المشاركون.**
- **وسيتم متابعة الأهداف تدريجيا و بشكل آمن .**
- **ستعمل العائلة ومدرب نمط الحياة وكل مشارك معًا كفريق واحد.**
- **الرصد الذاتي لتناول الطعام مهم للوصول إلى الهدف.**

**عرض الفصل الدراسي**

الجزء 1: مقدمة (10 دقائق)

مرحبا بالمشاركين

قل: أنتم هنا كجزء من برنامج الامارات لصحة القلب لمعرفة ما إذا كانت هذه التغييرات في نمط الحياة ستساعد في تقليل خطر الإصابة بأمراض القلب. هذه مشكلة كبيرة في هذا البلد، والمشروع، إذا كان ناجحًا، يمكن أن يساعد أهل هذا البلد في حل جزء من هذه المشكلة الكبيرة. سنعمل معًا كفريق واحد خلال الأسابيع الستة عشر القادمة لنصبح أكثر صحة من خلال تغيير نظامنا الغذائي ونشاطنا البدني.

خلال هذا الاجتماع الأول، سنبدأ في التعرف على بعضنا البعض، والتحدث عن كيفية عمل البرنامج، وما هي مسؤولياتنا.

**المقدمة**

**عرف عن نفسك** كمدرب نمط الحياة.

الاسم، المدة التي قضيتها في الإمارات العربية المتحدة، وكيف **شاركت في برنامج الامارات لصحة القلب**

ما سيكون دورك:

1. لمساعدة المشاركين على التعلم عن الأكل الصحي والنشاط البدني

2. لمساعدة المشاركين على تغيير عاداتهم للحد من مخاطر الإصابة بأمراض القلب.

3. لعرض على المشاركين أمثلة عن الأكل الصحي وتقنيات النشاط البدني التي يمكن تخصيصها لكل فرد.

4. لمساعدة المشاركين على تتبع كمية الطعام والدقائق ونوع النشاط البدني.

5. مساعدة المجموعة على حل أي تحديات تجعل تحقيق الهدف أكثر صعوبة ؛ ولمساعدة المجموعة على التعلم من بعضهم البعض ودعمهم

**اطلب من المشاركين تقديم أنفسهم.**

الاسم ، وسبب المشاركة في الدراسة

**حاضر/اعرض**: هذا الأسبوع سوف:

- نشرح الغرض من البرنامج وفوائده.
- سنراجع ما سنفعله خلال كل جلسة.
- معرفة أهداف فقدان الوزن الفردية.
- سنناقش أهمية المراقبة الذاتية.

**حاضر/اعرض:** قبل أن نواصل، دعونا نناقش بعض التفاصيل. سنخطط للقاء في نفس المكان والزمان كل أسبوع. إذا كنا بحاجة إلى تغيير هذا، يجب أن نناقش ونقرر كمجموعة كيفية تغيير هذا. إذا فاتتك جلسة، فيمكننا الحصول على جلسة مراجعة مرة أخرى. لا تتردد في الاتصال بي أو التواصل معي عن طريق الواتساب إذا كان لديك أي أسئلة أو بحاجة إلى أي دعم. وارجوا ابلاغي قبل موعد الجلسة بيوم إذا كنت لن تحضر الحصة

**اسأل:** هل أي شخص يرى أي مشاكل مع الجدول الزمني للاجتماعات (العطلات، والسفر)؟

اسمح للمشاركين بالرد.

**حاضر/اعرض:** يمكن للمجموعات، وخاصة العائلات أن يكون لها تأثير قوي. ومع ذلك، فإنهم يبذلون قصارى جهدهم عندما يتم الاتفاق على بعض الإرشادات وإتباعها.

**اسأل:** ما هي بعض القواعد الجيدة التي يجب علينا اتباعها كمجموعة خلال الاجتماعات الـ 16 المقبلة؟ (قد تحتاج إلى مشاركة فكرة أو اثنتين، مثل التحدث مع بعضهما البعض باحترام).

اكتبها على السبورة.

أخبر المجموعة أنك ستكتب قائمة بالمبادئ التوجيهية التي وافقوا عليها وسوف ترسلها عبر واتساب.

تأكد من تغطية القواعد الأساسية بطريقة إيجابية واجعل المشاركين يوافقون عليها.

الجزء 2: برنامج دراسة القلب في الامارات (10 دقائق)

**حاضر/اعرض:** الآن دعونا نتحدث عن البرنامج. هذا فيديو قصير حيث ستوضح الدكتورة درة البلوشي ماهية البرنامج ولماذا نعتقد أنه مهم.

**قم بإحالة** المرضى إلى نشرة جدول اجتماع البرنامج.

**حاضر/اعرض:** يحتوي هذا البرنامج على 16 جلسة ستعقد خلال الأسابيع 16-20 القادمة. وهي مقسمة إلى ثلاثة أقسام: ابدء الذي يغطي أساسيات الأكل الصحي والنشاط البدني ، وفهم العوامل التي تساعد في تشكيل سلوكيات الأكل والنشاط البدني وكيفية السيطرة على الأكل والنشاط البدني والتغيير طويل الأجل. التغيير صعب ويستغرق وقتًا. نريد أن نرى تغييرات صحية في حياتك لبقية حياتك. سيشمل هذا القسم:

حل المشاكل

تجنب المواقف المغرية

الأكل الصحي عند تناول الطعام خارج المنزل

إدارة الإجهاد

البقاء مندفع ومتحفز

**حاضر:** إن برنامج الامارات لصحة القلب له هدفين: فقدان الوزن (حوالي 7 ٪ من وزن الجسم الحالي) والنشاط البدني بانتظام. نود أن نراكم تمارسون نشاطًا مدته 150 دقيقة كل أسبوع بنهاية البرنامج. سنتحدث عن النشاط البدني في جلسة لاحقة.

**حاضر:** نريد تحقيق هذه الأهداف تدريجياً وبشكل آمن. وسنعمل جميعًا معًا لمساعدة بعضنا البعض للوصول إلى أهدافنا المتمثلة في فقدان الوزن عن طريق تناول طعام صحي ومزيد من النشاط البدني.

**اسأل:** كيف تعتقد أنك سوف تستفيد من تحقيق أهدافك؟

**اسمح لعدد قليل من المشاركين بالرد.**

الجزء 3: ماذا تتوقع (20 دقيقة)

**ما هو مطلوب من المشاركين:**

**حاضر/اعرض**: لدينا الكثير لنفعله معًا في الأشهر القليلة المقبلة. سيُطلب منكم إجراء بعض التغييرات في حياتكم ، وإذا قمتم بذلك ، نعتقد أنكم ستشعرون بالتحسن وستكونون أكثر صحة. كثير من الناس في جميع أنحاء العالم قاموا بذلك ، ويمكنكم القيام بذلك أيضًا. أنتم لن تكونوا لوحدكم. ستقوم عائلاتكم بهذا معكم ، وسأكون هنا لمساعدتكم في هذه العملية. لكن لا يمكنني القيام بذلك دون التزامكم.

**حاضر:** لكي تحصل على أقصى استفادة من هذا البرنامج ، أحتاج منك أن تلتزم بحضور كل جلسة. أحتاج منك أن تسجل الأطعمة والمشروبات التي تتناولها. و ان تقوم بتسجيل مقدار النشاط البدني. أريد منك أن تكون صادقا ودقيقا في القيام بذلك.

اسألني أسئلة ، قل لي مخاوفك. لكي أستطيع مساعدتك.

لا تستسلم! ستكون بعض الأيام أفضل من غيرها ، ولكن إذا بذلت قصارى جهدك ، فأنا أعرف أنك ستنجح.

**ما هو المطلوب من مدرب نمط الحياة:**

**حاضر:** يمكنك الاعتماد على و على المجموعة ل:

ملاحظة ما تفعله جيدًا وما الذي يمكن تحسينه.

الاجابة عن أسئلتك.

مشاركة الحلول والأفكار بينما نتعلم معًا.

كن صادقا.

ادعمك عندما تواجه التحديات.

نعتقد أنه يمكنك الوصول إلى أهدافك.

**اسأل:-**

هل هناك أي شيء آخر يمكنني القيام به من أجلك؟

أود منك الآن توقيع نشرة "سنعمل معًا".

**تذكر الغرض:**

**اسأل:** هل تتذكر لماذا نحن هنا وما نحاول القيام به؟

ما الشئ الذي يحمسك؟

ما الأشياء التي انت غير متحمس بخصوصها؟

ملاحظة: انتبه للمشاركين القلقين. قد يشعر البعض الآن بالارتباك لأنهم لا يعتقدون أنه يمكنهم القيام بذلك. معالجة المخاوف. فكر في التحدث معهم على انفراد.

الجزء 4: البدء في فقدان الوزن (20 دقيقة)

**حاضر:** الآن أنت تعرف ما هو البرنامج وكيف سيعمل. لقد سمعت أنك:

ستعمل كل أسبوع على تحقيق أهدافك الشخصية المتمثلة في فقدان الوزن من خلال الأكل الصحي والنشاط البدني.

سوف تتلقى المعلومات والدعم الذي تحتاجه للوصول إلى أهدافك بشكل آمن وتدريجي والحفاظ عليها. نحن لا نريد مجرد تغيير مؤقت.

**اسأل**: قبل أن أتابع ، هل هناك أي أسئلة حول ما ناقشناه حتى الآن؟

أجب عن أي أسئلة

**حاضر:** نحن الآن بصدد الحديث عن كيفية تحقيق أهدافك.

الشيء الأكثر أهمية الذي يمكننا القيام به هو تناول طعام صحي وتقليل عدد السعرات الحرارية التي نتناولها. والخطوة الأولى نحو تناول سعرات حرارية أكثر صحة وقليلة هي معرفة ما نتناوله الآن. للقيام بذلك ، سنقوم بتسجيل كل يوم كل ما نأكله ونشربه. سنواصل هذا طوال فترة البرنامج. تتبع ما نقوم به هو الجزء الأكثر أهمية في تغيير عاداتنا. هذا سوف يساعدنا على رؤية:

ما الأطعمة التي نأكلها.

كم نأكل

متى وأين نأكل.

كيف تتغير عاداتنا بمرور الوقت.

كيف يمكننا ضبط ما نأكله للحصول على نظام غذائي أكثر صحة وأكثر توازنا.

**الرجوع إلى المنشورات**

**حاضر:**

خلال هذا البرنامج ، أريدك أن تسجل كل شيء تأكله وتشربه. سوف تبدأ صباح الغد. فيما يلي بعض الأفكار الرئيسية:

- القواعد الاملائية\ الكتابية ليست مهمة.
- كن صادقا. اكتب كل شيء تأكله ، حتى لو كان صغيرًا جدًا.
- كن دقيقا. اكتب ما تأكله بأسرع ما يمكن حتى لا تنسى.
- كن شاملا. تشمل كل ما تأكله أو تشربه.
- ابذل قصارى جهدك لتقدير المبلغ. سوف نعمل على ذلك مع استمرار البرنامج ، وسوف تصبح أفضل في ذلك.
- بدون تدوين أو كتابة هذه الأمور ، سيكون من الصعب معرفة خطة فعالة لتناول الطعام.

**حاضر:** الآن لنأخذ دقيقة لممارسة هذا.

**اسأل:** ماذا تناولت في وجبة العشاء الليلة الماضية ، أو في وقت سابق من اليوم؟

ضع دائرة حول أي من الأطعمة غير الصحية.

حاضر: حجم الحصة مهم. اختلاف بسيط في حجم الحصة المتناولة يمكن أن يحدث فرقًا كبيرًا في كمية السكر والدهون والسعرات الحرارية.

قم بجولة حول الغرفة وساعد المشاركين عند الحاجة.

الجزء 5: الخاتمة وقائمة المهام (5 دقائق)

**قم بتوزيع** "متعقب/متتبع الطعام والنشاط" للأسبوع المقبل.

**حاضر:** للأسبوع التالي ، اكتب كل ما تأكله وتشربه ، كل يوم في متعقب الطعام.

اكتب: ماذا أكلت ، وكم أكلت و متى أكلته.

ضع دائرة حول الأطعمة التي تعتقد أنها الأعلى في السعرات الحرارية.

ابدأ بتقدير كمية الطعام الذي تتناوله.

**تلخيص النقاط الرئيسية:**

سجل الأهداف الشخصية والنتائج المتوقعة ؛ "ماذا أريد أن يحدث بسبب هذا؟"

تعليم الرصد الذاتي لتناول الطعام باستخدام طريقة اللوحة.

خلال هذا البرنامج ، سيكون لديك دعم كامل. سنفعل هذا معا.

نحن بحاجتك. يجب أن تكون ملتزمًا تجاه نفسك والآخرين.

حاضر: لقد وصلنا إلى نهاية هذه الجلسة. هل هناك أي أسئلة أو مخاوف بشأن أي شيء ناقشناه اليوم؟

**أجب عن أي أسئلة أو مخاوف.**

**حاضر:** في الأسبوع القادم ، سوف نلقي نظرة فاحصة على الأطعمة التي تزيد الدهون والسكر والسعرات الحرارية إلى نظامنا الغذائي. وإنني أتطلع إلى رؤيتكم الأسبوع المقبل!

**اسمح للمجموعة بالمغادرة.**
