# Supplementary material for: Emirates Heart Health Project (EHHP): A protocol for a stepped-wedge family-cluster randomized-controlled trial of a health-coach guided diet and exercise intervention to reduce weight and cardiovascular risk in overweight and obese UAE nationals
Source: PLoS One. 2023 Apr 10;18(4):e0282502. doi: 10.1371/journal.pone.0282502 (PMC10085020; doi:10.1371/journal.pone.0282502)
Supplement: S8 Appendix — (DOCX) [file pone.0282502.s008.docx]

**Session 2: Be a Fat and Calorie Detective**

**Objectives/goals:**

By the end of session 2, the participants will be able to:

- Self-monitor their weight for the following weeks.
- Document their weight at home.
- Explain the relationship between fat and calories.
- Explain the reason for, and how to do, self-monitoring of fat grams and calories.
- Identify their personal fat gram goals.
- Use the “Fat and Calorie Counter” to calculate the calories and fat grams of a given selection of foods.
- Keep a running total of the fat grams they eat each day.

**Materials:**

- Session 2 handouts
- Copies of ground rules from Session 1
- Food and Activity Trackers for Session 2
- Weight charts
- Fat and calorie counter
- Name tags and Sharpie marker
- White board
- Food samples for Part 3 of Session 2
- Measuring cups, spoons, ruler, and food scale.
- Scales

**Before you begin:**

- View the video.
- Review the objectives/goals of the session.
- Review the classroom presentation.

**Overview:**

Session 2 is about self-monitoring our weight, and estimating the amount of fat and calories we eat, which are essential parts of the program. We begin to use the weight tracking instrument. Participants will track their fat and calorie intake and see how reducing these will result in weight loss. Being a “fat and calorie detective” involves recognizing high fat and high calorie foods and reducing their intake.

There are 4 parts:

- Part 1: Weekly progress and review (10 minutes)
  - Each session will start with a short review of the information from the last session and then you will lead a discussion about group members’ successes, challenges and questions since last time.
- Part 2: Tracking your weight (10 minutes)
  - IYou will teach the group how to track their weight at home. Use an example chart to show them how.
- Part 3: Self-monitoring of fat intake (30 minutes)
  - You will teach the group the importance of their monitoring of fat and calorie intake.
  - They will also learn which foods are high in fat and calories.
  - They will be taught how to, and asked to record all the foods they eat.
  - They will also be assigned a fat gram goal and try to stay below it each day.
- Part 4: Wrap up and to-do list (10 minutes)
  - Summarize what was covered and assign tasks for the next week.

**Key messages:**

- **Consistent self-monitoring of weight and food intake are keys to achieving and maintaining long term weight loss.**
- **Participants can learn to estimate quickly and easily the amount of fat and calories in various goods. This will allow them to choose foods that together fall within their fat budget.**
- **Because fats are so high in calories, reducing fat is a relatively easy way of reducing the number of calories we eat.**
- **Reducing fat lowers cholesterol and reduces risk for heart attacks.**

**Classroom presentation:**

*Part 1: Weekly progress and review (10 minutes)*

Give each participant this session’s handouts.

**Review:** ground rules from last session. Ask the group if they want to change or add any rule.

**Present:** Last week, we reviewed the purpose and goals of this program and introduced you to some key ideas that we will focus on in the next few weeks.

We also saw the “Food and Activity Tracker” which will help you track your weight, food and physical activity.

**Ask**: How are you feeling about this week about your goals and what we are here to do?

**Open responses from the group.**

**Present:** As we go on, monitoring your weight and how much you eat will become a more natural part of your life. Remember, we will work together to overcome any challenges you will have along the way.

**Ask:** How did keeping track of what you ate and drank go last week? Were you able to write down everything that you ate and drank?

**Open responses from the group.**

**Note:** Address challenges, but do not allow this to be a complaining session. Keep the tone positive. Ask about successes and achievements.

**Ask:** What did you learn by this process? What did you see in your habits? What difficulties did you have?

**Open responses from the group.**

**Present:** At the end of our session here, I will collect your Food and Activity Trackers and return them to you next time. They will not be graded. The reason is to allow me to give you some individual feedback to help you reach your goals.

**Present:** Sometimes it is difficult to monitor what you eat. But as we have discussed, it is important to know what we are eating in order to make changes. For those of you who had trouble, tell me what happened.

**Open responses.**

**Ask:** Does anyone have suggestions for options for those who had a difficult time?

**Present:** In this program, we will help you overcome difficulties in tracking your progress. I know most of you are trying and making progress toward self-monitoring.

**Present:** This week we will learn to become fat and calorie detectives. We will become more aware of the amount of fat and the number of calories we eat. We will learn how knowing the amount we eat will help us lose weight. We will:

- Discuss how to monitor your weight at home.
- Identify your personal fat gram goals.
- Learn about how fat is related to calories.
- Use the Fat and Calorie Counter to find the number of fat grams and calories in food.
- Learn to keep a running fat and calorie total throughout the day.
- Learn how to read nutrition labels.

*Part 2: Tracking your weight (10 minutes)*

**Present:** Many of you are here to try to lose some weight. Last week we discussed the program’s weight loss goal for each of you: 7%.

We also talked about how we will record your progress toward that goal. Weekly here together, and daily at home and recorded on your “Food and Activity Tracker”.

**Hand out the weight charts.**

**Explain:** I’ve entered your starting weight and goal weight on this chart. On the top is the session week, and we will record your weight on this chart to see you reach your goal by week 24.

**Caution** the participants.

Your weight loss will vary from week to week.

Many people lose weight faster at first, and then average 0.5 – 1 kilogram a week.

We want to see a pattern of weight loss over time, not just focus on 1 or 2 numbers.

We want you not only to lose weight but to keep it off. We will work on how to do this together.

**Refer** to the “Food and Activity Tracker”.

**Present:** Last week you recorded your daily food and drink intake on this paper. This week you will also record your weight each day or at least twice a week.

Weigh yourself every day or every few days at the same time of day, wearing similar clothing.

Always use the same scale, because different scales may show slightly different readings.

*Part 3: Self monitoring fat intake (30 minutes)*

**Present:** This week we will learn how to be fat and calorie detectives. We will learn how much fat and how many calories we ear. And we will learn how knowing what we eat will help us to lose weight.

**Ask:** To start, who knows what fat is?

**Open responses.**

**Ask:** Who knows what calories are?

**Open responses.**

**Present:** Fat is one of many important nutrients that our bodies need. Fats are important to give us energy, protect our organs, keep us warm. Our bodies need fat, but not as much as most people eat. When we eat too much fat, our bodies store what it doesn’t need as extra weight.

A calorie is the way we measure how much energy is in food or drink. When you eat food or drink certain liquids, you take in energy, measured in calories. Calories can come from fat, carbohydrates or protein. A calorie is the same energy whether it comes from fat, carbohydrates or protein.

But… fat is the most concentrated in calories. Each gram of fat contains more than twice as many calories as a gram of carbohydrate or protein. Each gram of fat has 9 calories, where each gram of carbohydrate or protein has 4 calories.

**Ask:** What are examples of foods that are high in fat and calories?

**Open responses.**

**Present:** 1 kilo of fat in the body equals 7,700 calories. That means that if you want to lose one kilo per week, we need to reduce your calorie intake by 7,700 calories in a week, or about 1,100 calories per day. In this program we are aiming for 0.5-1 kilo per week, which is 550 to 1,100 calories less per day.

Reducing fat is a great way to reduce calories, while also producing benefits that reduce risk for type 2 diabetes. For the next few weeks we will concentrate on fat; how to avoid eating too much of it by tracking how much we do eat and then reducing the amount. However we do not have a specific calorie goal.

**Present:** Our goal is to help you learn to make healthy food choices. We want to reduce the amount of fat we eat because:

- Fat contains more than twice the calories as the same amount of carbohydrate or protein.
- Even a small amount of high fat food is high in calories. (Hold up a tablespoon.) A tablespoon of butter has 100 calories.
- Eating low fat foods may make you feel fuller and satisfied for fewer calories. (Air popped popcorn).

Effects of too much fat

**Watch:** The video with Dr. Durra will explain the effects of eating too much fat.

Excess fat is associated with heart disease and diabetes, which are the reasons we are interested in trying this program. Eating a lot of fat can increase your cholesterol. The higher the cholesterol in your blood, the higher your chances are of having a heart attack or stroke. Changing the amount and the type of food you eat is one of the most important things you can do to improve your health.

High fat foods

**Ask:** What foods do you eat that are high in fat?

**Open responses. Ask for volunteers to share.**

**Present:** Now look at some of the high fat foods that you circled in your “Food and Activity Tracker” which shows which foods and drinks you had last week.

Food groups and types of food that tend to be high in fat are: (write on whiteboard, it’s better if the group is participating)

- Meats
- Dairy (whole milk, regular cheese, ice cream, Rainbow)
- Chips
- Butter, margarine
- Cookies, cakes
- Fried foods
- Fast foods

These are the kinds of foods we have to watch out for as we become “fat and calorie detectives”. It can be hard to stop eating them because:

They are easy to get.

We like the taste.

They may be in traditional family or cultural foods.

They are part of celebrations: Ramadan, weddings, parties, etc.

Hidden fat

**Present:** Most of the fat we eat (70%) is hidden inside food.

**Ask:** What do we mean by “hidden”?

**Open responses.**

**Present:** So the fat that is hidden isn’t obvious. Examples include:

Marbling in meats

Baked foods

Sauces, salad dressings

Batter on fried foods.

**Present:** A lot of the fat in these items comes from how they are made. For example there is the same amount of fat in this milkshake as there is in this stick of butter. Because of the ice cream!

Cutting down on fat

**Present:** The best way to learn how much fat and calories are in foods is to keep track of fat and calories that you eat every day.

*Step 1:* Write down everything you eat and drink in your “Food and Activity Tracker.” This is the most important step toward changing your behavior. It will help us see:

- What foods you eat.
- When and where you eat.
- How much you eat.
- How your eating habits change over time.

What is not important? Spelling. As long as we understand what it is, spelling doesn’t matter.

What is important? Honesty (writing down everything you eat), Accuracy (as soon as you can so you don’t forget anything) and Completeness (even small things)/

*Step 2:* Figure out how many calories and fat grams are in every food. Record it in your Tracker. To do this, you need to:

Figure out the amount of food you ate.

Use the tracker to find out the total fat and calories based on how much you ate.

*Step 3:* Add up the totals of all the foods you ate during the day. I will show you how to do this.

Fat gram goal

**Present:** The reason we are keeping track of all of this is that we set a fat gram goal for each of you. Now we will use the Tracker to help us to keep our fat intake under our goal. Eating too little fat is not healthy and cannot be sustained for a long time, but eating too much fat leads to eating too many calories and keeps our weight high and increases the cholesterol in our blood.

Remember that each amount of fat has more than twice the calories as carbohydrates and protein, so we will work on reducing our fat intake first to lose weight by Session 7. If we don’t lose weight by then, we will also look at our other calories.

Now let’s calculate our fat goal.

Let’s look at the “Fat Gram Goal handout”.

**Present:** I have calculated your fat gram goal. Think of this as a budget where you’re trying to eat less than this amount per day. Everyone’s fat gram goal is different, because it is based on your body size and your calorie goal. The amount of fat is related to the total calorie goal (it is about a quarter).

**Present:** Everyone will have some successes and some challenges as we work together to reach our goal. For now, just try to get as close as you can. In the next few weeks we will learn new ways to reduce fat in your food, which will help you reach your weight loss goals.

Fat and calorie counter

**Present:** Let’s practice using our Fat Calorie Counter

{This will need some work. Smartphone app?}

**Roam the room, helping and checking on participants.**

**Discuss** if needed. Encourage the group that you will continue to help them with this now and in the future.

Estimating fat and calories in prepared foods

**Ask:** How do you handle recording fat grams and calories when you cook from recipes?

**Present:** If you cook from recipes you have two options:

1. Calculate how much of each ingredient you ate (how many kilos of lamb, how many cups of oil, how many cups of rice).
2. Find a similar food in the “Fat and Calorie Counter” and use the nutrition information for that food.

**Ask:** What if you eat food from a package?

**Present:** If you eat packaged foods, look on the label on the package. Find the serving size and see how many servings you ate.

Nutrition labels

**Present:** Now let’s learn together how we can read nutrition labels.

Present an example, pointing out serving size, fat grams, total calories.

Tracking fat and calorie totals.

**Present:** Now we know how to get an idea of how much fat and how many calories we are eating. And we know what our daily goal is for fat intake.

**Present:** Now let’s think about how we can record this over time so we can plan for healthy eating to reach our goal. By recording and totaling the fat grams and calories as we go through the day, we can see how much we have left until we will come to our goal intake for the day. This allows us to plan ahead.

Note: if the group is overwhelmed or confused, do not continue. We will try again next week. Instead focus on just recording intakes for this week.

**Present:** This is like keeping track of how much money we have to spend. When you look at what you have spent, you know how much you have left in the bank or your pocket to spend.

Give an example: How much should I eat for lunch? I have a wedding to go to tonight, where there will probably be a lot of fatty foods to eat. I had 10 grams of fat for breakfast, and my fat gram goal is 50 grams. I probably should have a light lunch, maybe 10 grams of fat so I can have fun at the wedding and still stay under my fat goal for the day.

**Demonstrate** how to add up fat grams.

**Ask** for 5 foods and their fat grams. Participants can use their Counter.

Write the foods and fat grams on the white board.

Add the fat grams from the second to the first and put the total to the side. Keep doing this until you sum all the fat grams of the five foods. Put the total at the bottom.

**Present:** I know this is a lot of new things. This week, we just want you to get started and do your best. You can contact me by WhatsApp, or I can help you with it next week.

Remember, changing the way we eat is a gradual process and it will take time.

We will keep teaching you new ways to help you eat healthier.

Right now, just be the best fat and calorie detective you can be. Look for fat and calories everywhere.

Do your best to stay under your goal each day.

We are more interested in your effort than in being perfect eaters.

We are working on self-monitoring, which is essential to being able to change.

**Ask** if there are any questions before going over the to-do list for the week.

*Wrap up and to-do (10 minutes)*

**Refer** participants to the “To-do list”.

**Present:** For next week:

- Weigh yourself at the same time each day (or every few days), and record your weight.
- Write down everything you eat and drink in your “Food and Activity Tracker.” Do this every day and as soon as possible after you eat. Be honest, accurate and complete.
- Measure portions as much as you can. Read labels if you need to.
- Use the “Fat and Calorie Counter” to figure out the amount of fat and colires you ate, and write it down in your “Food and Activity Tracker.”
- Keep a running fat gram total throughout the day. Try to stay under your fat gram goal everyday. Do your best.

**Collect** the “Food and Activity Trackers” from Session 1.

**Distribute** the new “Food and Activity Trackers”.

**Summarize** key points: Thank you for working hard and listening today. Today we:

- Learned how to monitor our weight.
- Which foods are high in fat. Those are foods we should stay away from.
- Figured out our personal fat gram goal, and how to use tools to help us remember that goal and stay under it each day.
- How to read food labels.

**Close:** At the next session, we will practice using tools that will help us keep track of foods more accurately. We will also learn new ways to eat less fat and fewer calories.

**Ask** participants for any questions.

**Dismiss the group.**

*After the session:*

Write notes and recommendations for improvement in each participant’s “Food and Activity Tracker” from session 1. Limit your notes and suggestions to these topics:

- Process of recording
- Running total
- Self weighing
- How close they are to their fat gram goals.
