# Supplementary material for: Emirates Heart Health Project (EHHP): A protocol for a stepped-wedge family-cluster randomized-controlled trial of a health-coach guided diet and exercise intervention to reduce weight and cardiovascular risk in overweight and obese UAE nationals
Source: PLoS One. 2023 Apr 10;18(4):e0282502. doi: 10.1371/journal.pone.0282502 (PMC10085020; doi:10.1371/journal.pone.0282502)
Supplement: S9 Appendix — (DOCX) [file pone.0282502.s009.docx]

**الجلسة رقم 2: كن مستكشفا/متتبعا للدهون والسعرات الحرارية**

**الأهداف:**

بحلول نهاية الجلسة الثانية ، سيكون المشاركون قادرين على:

- الرصد الذاتي لأوزانهم للأسابيع التالية.
- توثيق أوزانهم في المنزل.
- شرح العلاقة بين الدهون والسعرات الحرارية.
- شرح سبب وكيفية القيام بعملية الرصد الذاتي لغرامات الدهون والسعرات الحرارية.
- تحديد هدفهم الشخصي بالنسبة لغرامات الدهون
- استخدم "عداد الدهون والسعرات الحرارية" لحساب السعرات الحرارية وغرامات الدهون لمجموعة مختارة من الأطعمة.
- الاحتفاظ بمجموع غرامات الدهون التي يتناولونها كل يوم.

**المواد:**

- منشورات الجلسة الثانية
- نسخة من القواعد الأساسية من الجلسة الأولى
- متعقب الطعام والنشاط الرياضي للدورة الثانية
- مخططات الوزن
- حاسبة الدهون والسعرات الحرارية
- بطاقة الاسم و قلم
- سبورة البيضاء
- عينات المواد الغذائية للجزء 3 من الجلسة الثانية
- أكواب القياس , ملاعق ،مسطرة ، وميزان الطعام
- مقاييس / موازين

**قبل ان تبدأ:**

- عرض الفيديو.
- مراجعة أهداف الجلسة
- مراجعة عرض الفصل

**نظرة عامة:**

تدور الجلسة الثانية حول المراقبة الذاتية للوزن وتقدير كمية الدهون والسعرات الحرارية التي نتناولها ، والتي تعد جزءًا أساسيًا من البرنامج. سنبدأ في استخدام أداة تتبع الوزن. سيتابع المشاركون تناولهم للدهون والسعرات الحرارية ويرون كيف سيؤدي تقليلها إلى فقدان الوزن. كونك "محققًا للدهون والسعرات الحرارية" يتضمن التعرف على الأطعمة الغنية بالدهون والسعرات الحرارية العالية وتقليل تناولها.

هناك 4 أجزاء:

لجزء 1: التقدم الأسبوعي والمراجعة (10 دقائق)

ستبدأ كل جلسة بمراجعة مختصرة للمعلومات من الجلسة الأخيرة ، وبعد ذلك ستكون هنالك مناقشة حول نجاحات أعضاء المجموعة والتحديات والأسئلة منذ آخر مرة.

الجزء 2: تتبع وزنك (10 دقائق)

سوف تعلم المجموعة كيفية تتبع وزنهم في المنزل. استخدم مخطط الوزن كمثال لتوضيح كيفية ذلك.

الجزء 3: الرصد الذاتي لتناول الدهون (30 دقيقة)

سوف تعلم المجموعة أهمية مراقبة الدهون والسعرات الحرارية.

سوف يتعلمون أيضا الأطعمة التي تحتوي على نسبة عالية من الدهون والسعرات الحرارية.

سيتم تعليمهم طريقة ذلك ، ويطلب منهم تسجيل جميع الأطعمة التي يتناولونها.

سيتم أيضًا تحديد كمية غرامات الدهون التي يحتاجها كل شخص ومحاولة البقاء دونه يوميًا.

الجزء 4: اختتام وقائمة المهام (10 دقائق)

تلخيص ما تم تغطيته وتحديد المهام للأسبوع المقبل.

**الرسائل الرئيسية:**

- تعد المراقبة الذاتية المستمرة للوزن وتناول الطعام من العناصر الرئيسية لتحقيق والحفاظ على فقدان الوزن على المدى الطويل.
- يمكن للمشاركين تعلم تقدير كمية الدهون والسعرات الحرارية في السلع المختلفة بسرعة وسهولة. هذا سيسمح لهم باختيار الأطعمة التي تقع ضمن ميزانية الدهون الخاصة بهم.
- نظرًا لأن الدهون تحتوي على نسبة عالية من السعرات الحرارية ، فإن تقليل الدهون طريقة سهلة نسبيًا لتقليل عدد السعرات الحرارية التي نتناولها.
- خفض الدهون يخفض الكوليسترول ويقلل من خطر الاصابة بالازمات القلبية.

**عرض الفصل الدراسي:**

الجزء 1: التقدم الأسبوعي والمراجعة (10 دقائق)

امنح كل مشارك منشورات هذه الجلسة.

**مراجعة**: القواعد الأساسية من الجلسة الأخيرة. اسأل المجموعة عما إذا كانوا يريدون تغيير أو إضافة أي قاعدة.

**حاضر:** في الأسبوع الماضي ، قمنا بمراجعة غرض وأهداف هذا البرنامج وقدمنا ​​لك بعض الأفكار الرئيسية التي سنركز عليها في الأسابيع القليلة القادمة.

لقد شاهدنا أيضًا "متبع الطعام والنشاط " الذي سيساعدك على تتبع وزنك وطعامك ونشاطك البدني.

**اسأل:** ما هو شعورك حول هذا الأسبوع عن أهدافك وما هو شعورك حول ما يجب علينا القيام به؟

استمع لردود المجموعة.

**حاضر:** مع تقدمنا ​​، ستصبح مراقبة وزنك ومقدار ما تأكله جزءًا طبيعيًا في حياتك. تذكر ، سنعمل معًا للتغلب على أي تحديات ستواجهك طوا ل الطريق.

**اسأل:** كيف تتتبعت ما أكلته وشربته في الأسبوع الماضي؟ هل كنت قادرًا على كتابة كل ما أكلته وشربته؟

استمع لردود المجموعة.

**ملحوظة:** تعامل مع التحديات ، لكن لا تسمح لها أن تكون دورة شكاوى. الحفاظ على لهجة إيجابية. اسأل عن النجاحات والإنجازات.

**اسأل:** ماذا تعلمت من هذه العملية؟ ماذا رأيت في عاداتك؟ ما الصعوبات التي واجهتك؟

**افتح المجال لسماع الرد من المجموعة.**

**حاضر:** في نهاية جلستنا هذه ، سوف أقوم بتجميع متتبعي الطعام والنشاط وإعادتهم إليكم في المرة القادمة. لن يتم تقيمهم. السبب هو السماح لي بتقديم بعض الملاحظات الفردية لمساعدتك في الوصول إلى أهدافك.

**حاضر:** في بعض الأحيان يكون من الصعب مراقبة ما تأكله. ولكن كما ناقشنا ، من المهم معرفة ما نأكله من أجل إجراء تغييرات. لأولئك الذين واجهوا مشاكل ، الرجاء اخباري بما حدث.

**افتح المجال لسماع الرد من المجموعة.**

**اسأل:** هل لدى أي شخص اقتراحات للحصول على خيارات للذين مروا بوقت عصيب؟

**حاضر:** في هذا البرنامج ، سنساعدك في التغلب على الصعوبات في تتبع تقدمك. أعرف أن معظمكم يحاول إحراز تقدم نحو المراقبة الذاتية.

**حاضر:** سوف نتعلم هذا الأسبوع أن نصبح محققين للدهون والسعرات الحرارية. سنصبح أكثر وعيا لكمية الدهون وعدد السعرات الحرارية التي نتناولها. سوف نتعلم كيف أن معرفة الكمية التي نتناولها ستساعدنا على إنقاص الوزن. سنقوم:

- مناقشة كيفية مراقبة الوزن في المنزل.
- تحديد أهداف غرامات الدهون الشخصية.
- التعرف على علاقة الدهون بالسعرات الحرارية.
- استخدام عداد الدهون والسعرات الحرارية للتعرف على كميات الدهون والسعرات الحرارية في الطعام.
- تعلم كيفية الحفاظ على إجمالي الدهون والسعرات الحرارية على مدار اليوم.
- تعلم كيفية قراءة ملصقات التغذية.

الجزء 2: تتبع وزنك (10 دقائق)

**حاضر:** كثير منكم هنا لمحاولة فقدان بعض الوزن. ناقشنا في الأسبوع الماضي هدف خسارة الوزن في البرنامج لكل واحد منكم: 7٪.

تحدثنا أيضًا عن كيفية تسجيل تقدمك نحو هذا الهدف. أسبوعيًا معًا ، يوميًا في المنزل وتسجيله على "متتبع الطعام والنشاط".

وزع الرسوم البيانية للوزن.

**قم بشرح الآتي**: لقد أدخلت وزنك الأساسي ووزن الهدف في هذا المخطط. في الأعلى هو أسبوع الجلسة ، وسنسجل وزنك على هذا المخطط لنرى وصولك إلى هدفك بحلول الأسبوع 24.

**حذر** المشاركين.

سوف يختلف وزنك من أسبوع لآخر.

كثير من الناس يفقدون الوزن بشكل أسرع في البداية ، ثم في المتوسط ​​0.5 - 1 كيلوغرام في الأسبوع.

نريد أن نرى نمطًا من فقدان الوزن مع مرور الوقت ، وليس فقط التركيز على رقم واحد أو اثنين.

نحن نريدك ليس فقط أن تفقد الوزن ولكن الحفاظ عليه أيضا. سوف نعمل على كيفية القيام بذلك معًا.

**ارجع** إلى "متتبع الطعام والنشاط".

**حاضر:** قمت في الأسبوع الماضي بتسجيل كمية الطعام والشراب اليومية على هذه الورقة. هذا الأسبوع سوف تسجل وزنك كل يوم أو مرتين على الأقل في الأسبوع.

تزن نفسك كل يوم أو كل بضعة أيام في نفس الوقت من اليوم ، واحرص على ارتداء ملابس مماثلة.

استخدم دائمًا نفس المقياس ، لأن المقاييس المختلفة قد تظهر قراءات مختلفة قليلاً.

الجزء 3: كمية الدهون في المراقبة الذاتية (30 دقيقة)

**حاضر:** هذا الأسبوع سوف نتعلم كيف أن نتتبع الدهون والسعرات الحرارية. سوف نتعلم حساب مقدار الدهون وعدد السعرات الحرارية التي نتناولها. وسوف نتعلم كيف معرفة ما نأكله سوف يساعدنا على فقدان الوزن.

**اسأل:** للبدء ، من يعرف ماهية الدهون؟

**افتح المجال للرد**

**اسأل:** من يعرف ماهية السعرات الحرارية؟

**افتح المجال للرد**

**حاضر:** الدهون هي واحدة من العديد من العناصر الغذائية الهامة التي تحتاج أجسامنا. الدهون مهمة لمنحنا الطاقة ، وحماية أعضائنا ، وتبقينا دافئين. تحتاج أجسامنا إلى الدهون ، لكن ليس بقدر ما يأكل معظم الناس. عندما نأكل الكثير من الدهون ، فإن أجسامنا تخزن ما لا تحتاجه كوزن إضافي.

السعرات الحرارية هي الطريقة التي نقيس بها كمية الطاقة الموجودة في الطعام أو الشراب. عندما تأكل طعامًا أو تشرب بعض السوائل ، فإنك تستهلك طاقة تقاس بالسعرات الحرارية. السعرات الحرارية يمكن أن تأتي من الدهون والكربوهيدرات أو البروتين. السعرات الحرارية هي نفس الطاقة سواء أكانت ناتجة عن الدهون أو الكربوهيدرات أو البروتين.

ولكن ... الدهون هي الأكثر تركيزا في السعرات الحرارية. يحتوي كل جرام من الدهون على أكثر من ضعف السعرات الحرارية التي يحتوي عليها جرام من الكربوهيدرات أو البروتين. كل غرام من الدهون يحتوي على 9 سعرات حرارية ، حيث يحتوي كل جرام من الكربوهيدرات أو البروتين على 4 سعرات حرارية.

**اسأل:** ما هي أمثلة الأطعمة التي تحتوي على نسبة عالية من الدهون والسعرات الحرارية؟

**افتح المجال للرد .**

**حاضر :** 1 كيلو من الدهون في الجسم يساوي 7،700 سعرة حرارية. هذا يعني أنه إذا كنت تريد أن تفقد كيلو واحد في الأسبوع ، فنحن بحاجة إلى تقليل السعرات الحرارية التي تحصل عليها بواقع 7،700 سعرة حرارية في الأسبوع ، أو حوالي 1100 سعرة حرارية في اليوم. في هذا البرنامج ، نهدف إلى الحصول على 0.5-1 كيلو في الأسبوع ، أي ما يتراوح بين 550 و 1100 سعر حراري في اليوم.

تعد تقليل الدهون طريقة رائعة لتقليل السعرات الحرارية ، بينما ينتج عنها أيضًا فوائد تقلل من خطر الإصابة بالنوع الثاني من مرض السكري. خلال الأسابيع القليلة القادمة سوف نركز على الدهون. كيفية تجنب تناول الكثير منها بمراقبة كمية الطعام التي نتناولها ثم تقليل الكمية. ومع ذلك ، ليس لدينا هدف محدد للسعرات الحرارية.

**حاضر:** هدفنا هو مساعدتك على تعلم اتخاذ الخيارات الغذائية الصحية. نريد تقليل كمية الدهون التي تتناولها لأن:

- الدهون تحتوي على أكثر من ضعف السعرات الحرارية بنفس كمية الكربوهيدرات أو البروتين.
- حتى كمية صغيرة من المواد الغذائية عالية الدهون عالية في السعرات الحرارية. (امسك ملعقة كبيرة). ملعقة كبيرة من الزبدة بها 100 سعرة حرارية.

تناول الأطعمة قليلة الدسم قد يجعلك تشعر بالشبع والرضا لأن السعرات الحراريةتكون أقل. ( الفشار).

آثار تناول كميات كبيرة من الدهون

**شاهد:** الفيديو مع الدكتورة درة سيوضح آثار تناول كميات كبيرة من الدهون.

ترتبط الدهون الزائدة بأمراض القلب والسكري ، وهي الأسباب التي تجعلنا مهتمين بتجربة هذا البرنامج. تناول الكثير من الدهون يمكن أن يزيد من نسبة الكوليسترول في الدم. كلما ارتفع مستوى الكوليسترول في الدم ، زادت فرصتك في الإصابة بنوبة قلبية أو سكتة دماغية. يعد تغيير كمية ونوع الطعام الذي تتناوله أحد أهم الأشياء التي يمكنك القيام بها لتحسين صحتك.

الأطعمة عالية الدهون

**اسأل:** ما الأطعمة التي تتناولها والتي تحتوي على نسبة عالية من الدهون؟

**افتح المجال للرد. اطلب متطوعين للمشاركة.**

**حاضر:** انظر الآن إلى بعض الأطعمة التي تحتوي على نسبة عالية من الدهون والتي قمت بتدوينها في "متتبع الطعام والنشاط" الذي يعرض الأطعمة والمشروبات التي تناولتها في الأسبوع الماضي.

مجموعات الأطعمة وأنواع الأطعمة التي تحتوي على نسبة عالية من الدهون هي: (الكتابة على السبورة ، من الأفضل أن تشارك المجموعة)

- اللحوم
- منتجات الألبان (الحليب كامل الدسم ، الجبن العادي ، الآيس كريم , حليب أبوقوس )
- رقائق البطاطا
- الزبدة والسمن
- البسكويت و الكعك
- الأطعمة المقلية
- الوجبات السريعة

هذه هي أنواع الأطعمة التي يجب أن نراقبها عندما نصبح "محققين للدهون والسعرات الحرارية". قد يكون من الصعب التوقف عن تناوله لأنه:

من السهل الحصول عليها.

نحن نحب التذوق.

قد تكون من الأطعمة الثقافية أو التقليدية.

إنها جزء من المناسبات والاحتفالات: رمضان ، حفلات الزفاف ، الحفلات ألأخرى ، إلخ.

الدهون المخفية

**حاضر:** معظم الدهون التي نأكلها (70٪) مخبأة داخل الطعام.

**اسأل:** ماذا نعني بكلمة "خفية"؟

**افتح المجال لردود المجموعة**

**حاضر:** إذاً الدهون المخفية غير واضحة. الامثله تشمل:

الدهون في اللحوم

الأطعمة المخبوزة

الصلصات ، صلصات السلطة

الخليط في الأطعمة المقلية.

**حاضر:** الكثير من الدهون في هذه العناصر تأتي من كيفية صنعها. على سبيل المثال ، هناك نفس كمية الدهون في اللبن المخفوق كما هو الحال في الزبدة هذه. بسبب الآيس كريم!

خفض الدهون

حاضر: إن أفضل طريقة لمعرفة مقدار الدهون والسعرات الحرارية الموجودة في الأطعمة هي تتبع الدهون والسعرات الحرارية التي تتناولها كل يوم.

الخطوة 1: اكتب كل ما تأكله وتشربه . هذه هي أهم خطوة نحو تغيير سلوكك. سوف يساعدنا على رؤية:

- ما الأطعمة التي تتناولها.
- متى وأين تأكل.
- كم الكمية التي تأكل.
- كيف تتغير عاداتك الغذائية بمرور الوقت.

ما هو غير مهم؟ الكتابة الإملائية. طالما نفهم ما هو المقصود ، لا يهم الإملاء.

ما هو المهم؟ الصدق (اكتب كل ما تأكله) والدقة (بأسرع ما يمكن حتى لا تنسى أي شيء) والكمال (حتى الأشياء الصغيرة)

الخطوة 2: معرفة عدد السعرات الحرارية وغرامات الدهون الموجودة في كل طعام. تسجيله في الدفتر الخاص بك. للقيام بذلك ، تحتاج إلى:

معرفة كمية الطعام الذي تناولته.

استخدم المتعقب لمعرفة إجمالي الدهون والسعرات الحرارية بناءً على مقدار ما تأكله.

الخطوة 3: أضف جميع الأطعمة التي تتناولها خلال اليوم. سوف تظهر لك كيفية القيام بذلك.

هدف حساب الدهون

**حاضر:** السبب في أننا نتابع كل هذا هو أننا وضعنا هدفًا غراميًا من الدهون لكل واحد منكم. الآن سوف نستخدم المتتبع لمساعدتنا في الحفاظ على تناول الدهون لدينا بشكل أقل من هدفنا. إن تناول القليل من الدهون ليس صحيًا ولا يمكن تحمله لفترة طويلة ، لكن تناول الكثير من الدهون يؤدي إلى تناول الكثير من السعرات الحرارية والحفاظ على وزننا مرتفعًا ويزيد من نسبة الكوليسترول في الدم.

تذكر أن كل كمية من الدهون تحتوي على أكثر من ضعف السعرات الحرارية مثل الكربوهيدرات والبروتين ، لذلك سنعمل على تقليل استهلاكنا للدهون أولاً لفقدان الوزن بحلول الجلسة السابعة. السعرات الحرارية الأخرى.

الآن دعونا نحسب هدف غرامات الدهون التي نحتاجها

دعنا نلقي نظرة على "نشرة هدف غرام الدهون".

**حاضر:** لقد حسبت هدفك من الدهون. فكر في هذا كميزانية حيث تحاول أن تأكل أقل من هذا المبلغ في اليوم. هدف كل شخص في غرام الدهون مختلف ، لأنه يعتمد على حجم جسمك وهدف السعرات الحرارية. يرتبط مقدار الدهون بالهدف الكلي للسعرات الحرارية (حوالي ربع).

**حاضر:** سيكون لكل شخص بعض النجاحات وبعض التحديات بينما نعمل معًا لتحقيق هدفنا. في الوقت الحالي ، حاول فقط الاقتراب قدر المستطاع. في الأسابيع القليلة المقبلة ، سنتعلم طرقًا جديدة لتقليل الدهون في طعامك ، مما سيساعدك على بلوغ أهدافك المتعلقة بفقدان الوزن.

الدهون والسعرات الحرارية مضادة

**حاضر:** هيا نمارس استخدام عداد السعرات الحرارية الخاص بنا

{هذا سوف يحتاج إلى بعض العمل. تطبيق الهاتف الذكي؟}

**تجول في الغرفة ، لمساعدة المشاركين.**

**ناقش** إذا لزم الأمر. شجع المجموعة التي ستستمر في مساعدتها في هذا الأمر الآن وفي المستقبل.

تقدير الدهون والسعرات الحرارية في الأطعمة الجاهزة

**اسأل:** كيف يمكنك التعامل مع تسجيل غرامات الدهون والسعرات الحرارية عند طهي الطعام من الوصفات؟

تقديم: إذا كنت تطبخ من الوصفات لديك خياران:

1. احسب مقدار كل عنصر أكلته (كم كيلوغرام من لحم الغنم ، وكم كوب من الزيت ، وكم كوب من الأرز).

2. ابحث عن طعام مماثل في "عداد الدهون والسعرات الحرارية" واستخدم معلومات التغذية لذلك الطعام.

**اسأل:** ماذا لو كنت تأكل الطعام من عبوة؟

حاضر: إذا كنت تأكل الأطعمة المعلبة ، انظر إلى الملصق الموجود على العبوة. ابحث عن حجم الحصة وشاهد عدد الوجبات التي تتناولها.

تسميات التغذية

**حاضر:** الآن دعونا نتعلم معا كيف يمكننا قراءة ملصقات التغذية.

قدم مثالاً ، مع الإشارة إلى حجم الحصة ، غرامات الدهون ، السعرات الحرارية الكلية.

تتبع مجموع الدهون والسعرات الحرارية.

**حاضر:** الآن نحن نعرف كيفية الحصول على فكرة عن مقدار الدهون وعدد السعرات الحرارية التي نتناولها. ونحن نعرف ما هو هدفنا اليومي هو تناول الدهون.

**حاضر:** الآن دعونا نفكر في كيفية تسجيل هذا مع مرور الوقت حتى نتمكن من التخطيط لتناول الطعام الصحي للوصول إلى هدفنا. من خلال تسجيل وجمع غرامات الدهون والسعرات الحرارية مع مرور اليوم ، يمكننا أن نرى مقدار ما تبقى لدينا حتى نصل إلى هدفنا خلال اليوم. هذا يسمح لنا بالتخطيط للمستقبل.

**ملاحظة**: إذا كانت المجموعة مشوشة ، فلا تقم بالمتابعة. سنحاول مرة أخرى الأسبوع المقبل. ركز بدلاً من ذلك على تسجيل مآخذ هذا الأسبوع فقط.

**حاضر:** هذا يشبه تتبع مقدار الأموال التي يتعين علينا إنفاقها. عندما تنظر إلى ما أنفقته ، فأنت تعرف المبلغ المتبقي في البنك أو في جيبك الذي تنفقه.

أعط مثالاً: كم يجب أن اتناول على الغداء؟ لدي حفل زفاف للذهاب الليلة ، حيث من المحتمل أن يكون هناك الكثير من الأطعمة الدهنية لتناول الطعام. كان لدي 10 غرامات من الدهون لتناول الافطار وهدفي من الغرامات هو 50 غراما. ربما يجب أن أتناول غداءً خفيفًا ، ربما 10 غرامات من الدهون حتى أتمكن من قضاء وقت ممتع في حفل الزفاف وما زلت أظل تحت هدف الدهون الخاص بي لهذا اليوم

**اشرح** كيفية إضافة غرامات من الدهون.

اسأل عن 5 أطعمة وكمية الدهون بها . يمكن للمشاركين استخدام العداد الخاص بهم.

اكتب الأطعمة وكمية غرامات الدهون على السبورة البيضاء.

أضف غرامات الدهون من الثانية إلى الأولى ثم ضعيها على الجانب. استمر في القيام بذلك حتى تقوم بتلخيص جميع غرامات الدهون في الأطعمة الخمسة. ضع المجموع في الأسفل.

**حاضر:** أعرف أنها الكثير من الأشياء الجديدة. هذا الأسبوع ، نريد فقط أن تبدأ وتفعل ما بوسعك. يمكنك الاتصال بي عن طريق واتساب ، أو يمكنني مساعدتك في الأسبوع المقبل.

تذكر أن التغيير عملية تدريجية وستستغرق بعض الوقت.

سوف نستمر في تعليمك طرقًا جديدة لمساعدتك على تناول طعام أكثر صحة.

الآن ، فقط كن أفضل متتبع/مستكشف للدهون والسعرات الحرارية. ابحث عن الدهون والسعرات الحرارية في كل مكان.

ابذل قصارى جهدك للبقاء تحت هدفك كل يوم.

نحن مهتمون أكثر بجهدك أكثر من أن تكون مثالي.

نحن نعمل على الرصد الذاتي ، وهو أمر ضروري ليساعدك على التغيير.

**اسأل** ما إذا كان هناك أي أسئلة قبل الانتقال إلى قائمة المهام خلال الأسبوع.

الخاتمة (10 دقائق)

قم بالرجوع الى "قائمة المهام مع المشاركين .

**حاضر:** للأسبوع القادم:

زن نفسك في نفس الوقت كل يوم (أو كل بضعة أيام) ، وسجل وزنك.

اكتب كل ما تأكله وتشربه في "متتبع الطعام والنشاط". افعل ذلك كل يوم وبأسرع وقت ممكن بعد الأكل. يجب أن صادق ودقيق وشامل.

قياس كميات الطعام بقدر ما تستطيع. قراءة البطاقات الغذائية إذا كنت بحاجة إل ذلك .

استخدم "عداد الدهون والسعرات الحرارية" لمعرفة مقدار الدهون والسعرات التي تتناولها ، وقم بتدوينها في "متتبع الطعام والنشاط ".

الحفاظ على مجموع الدهون على مدار اليوم. محاولة البقاء اقل من الهدف كل يوم. ابذل قصارى جهدك.

**قم بجمع** "متعقب الطعام والنشاط" من الجلسة الأولى.

**قم بتوزيع** "متعقب و الطعام والنشاط" الجديد.

**لخص** النقاط الأساسية: شكرًا لك على العمل الجاد والاستماع اليوم. اليوم نحن:

- تعلمنا كيفية مراقبة أوزاننا.
- الأطعمة التي تحتوي على نسبة عالية من الدهون. هذه هي الأطعمة التي يجب علينا الابتعاد عنها.
- تعلمنا حساب هدف الدهون الشخصي الخاص بنا ، وكيفية استخدام الأدوات لمساعدتنا على تذكر هذا الهدف والبقاء تحته كل يوم.
- كيفية قراءة الملصقات الغذائية

**الخاتمة**: في الجلسة التالية ، سنتدرب على استخدام الأدوات التي ستساعدنا على تتبع الأطعمة بشكل أكثر دقة. سوف نتعلم أيضًا طرقًا جديدة لتناول كميات أقل من الدهون والسعرات الحرارية .

اسأل المشاركين اذا كان لديهم أي أسئلة.

**اسمح لللمجموعة بالمغادرة.**

بعد الجلسة:

اكتب ملاحظات وتوصيات للتحسين في برنامج "تعقب الطعام والنشاط" لكل مشارك من الجلسة الأولى . حدد ملاحظاتك واقتراحاتك على هذه الموضوعات:

عملية التسجيل

مجموع تشغيل

وزن النفس

مدى قربهم من أهداف غرامات الدهون .
