# Supplementary material for: Emirates Heart Health Project (EHHP): A protocol for a stepped-wedge family-cluster randomized-controlled trial of a health-coach guided diet and exercise intervention to reduce weight and cardiovascular risk in overweight and obese UAE nationals
Source: PLoS One. 2023 Apr 10;18(4):e0282502. doi: 10.1371/journal.pone.0282502 (PMC10085020; doi:10.1371/journal.pone.0282502)
Supplement: S10 Appendix — (DOCX) [file pone.0282502.s010.docx]

**Session 3: Eating Less Fat and Fewer Calories**

**Objectives/goals:**

By the end of session 3, the participants will be able to:

- Weigh and measure foods.
- Estimate the fat and calorie content of common foods.
- Describe three ways to eat less fat and fewer calories.
- Create a plan to eat less fat for the following week.

**Materials:**

- Participant handouts
- Food and Activity Trackers for Session 3. Label with current session number. Have extra copies available.
- Session 1 Food and Activity Trackers with your comments for each participant
- Fat and Calorie Counter
- Name tags
- White board with marker
- Scale
- Measuring cups, measuring spoons, food scale
- Food or food models
  - Try to select foods that the participants said were part of their diet (pasta, rice, meat, French fries)
  - Weigh the foods in advance, write the weight on tape and put it on the bottom of the food container.
  - 3 teaspoons soft margarine in a plastic tub (12 grams of fat)
  - 4 tablespoons oil on the bottom of a frying pain
  - 4 tablespoons oil in a small bowl
  - 1 ¾ cup macaroni
  - 3 cups butter flavored movie popcorn (29 gm fat)
  - 1 ½ cups whole milk in a bowl (12 grams fat)
  - 3 ounces hamburger after cooked (19 gm fat)
  - 4 ounces cheddar cheese (28 grams fat)
  - Empty packages of chips, nuts, cake
  - Butter: 1 teaspoon = 4 grams fat, 1 stick = 96 grams fat

**Before you begin:**

- View the video.
- Review the objectives/goals of the sessions.
- Review the classroom presentation.
- Make sure you have all materials and handouts you need.

**Overview:**

Session 3 is about eating less fat and brings the new practice of weighing and measuring foods to more accurately determine how much food is being consumed. We also talk about three ways to eat less fat and fewer calories, and use these to create a plan to eat healthier for the following week.

There are 4 parts:

- Part 1: Weekly progress and review (10 minutes)
  1. Review the information from the last session.
  2. Lead a discussion about participants’ successes, challenges and questions since the last session.
- Part 2: Weighing and measuring (20 minutes)
  1. Lead participants through a practice session using:
     - Measuring spoons and cups for solids
     - Measuring cup for liquids
     - Scale for solids.
  2. Participants will guess portion sizes and fat content and then verify using measuring and other tools.
  3. They will be shown actual fat content compared to butter in a dish.
- Part 3: Three ways to eat less fat and fewer calories (20 minutes)
  1. Eating fat less often
  2. Eating fat in smaller amounts
  3. Substituting lower fat or lower calorie foods.
- Part 4: Wrap and to-do list (10 minutes)

**Key messages:**

- **Knowing portion size is the key to accurately calculating fat intake.**
- **Accurately calculating fat intake is key to staying within your fat gram goal.**
- **Staying within your fat gram goal is key to losing weight.**
- **Measuring your food is one way to know your portion size.**
- **You can know how much fat and how many calories are in food.**
- **Eating less fat requires making a decision to eat high-fat and high-calorie foods less often and in smaller amounts and eating low-fat and low-calorie foods instead.**

**Classroom presentation**

*Part 1: Weekly progress and review (10 minutes)*

**Distribute** hand-outs.

**Collect** Session 2 “Food and Activity Trackers” after the session.

**Review** ground rules, if needed.

**Discuss** participants’ successes and difficulties in meeting their goals in the past week.

**Present:** Last week we talked about fat gram goals, reducing the overall calories you eat, and tracking your progress. During this last week our goal was for you to weigh yourself at least every few days and to write down everything you ate and drank. We also talked about becoming familiar with the idea of measuring portions- and whatever you eat- when and where possible.

**Ask:** Did you weigh yourself at home? How are you feeling about your goals and your progress so far?

**Remind:** Two scales may show slightly different weights, but the overall pattern if you use the same scales should be similar. If you are losing weight, the numbers should be changing by the same amount on your home scale and at our meetings.

**Open responses.**

**Ask:**

How did it go using the “Fat and Calorie Counter”?

Were you able to keep a running subtotal of fat grams?

How close did you come to meeting your fat gram goals?

What positive changes did you make this week?

What difficulties did you experience?

**Address** any questions or difficulties.

**Present:** This week we will:

- Show you how to weigh and measure food to determine portion size and fat grams.
- Learn how to estimate the fat content of common foods.
- Discuss 3 ways to eat less fat and fewer calories.
- Create a plan for you to eat less fat.

*Part 2: Weighing and measuring (20 minutes)*

**Ask:** How many of you paid closer attention to portion sizes since we started this program? How has keeping track of *what* you eat changed *how* you eat or *how* you view portion sizes?

**Open responses.**

**Present:** Portion size is important. So in addition to reading labels for packaged foods, we encouraged you to use measuring cups and spoons and a scale. Weighing and measuring foods is an important step to know what we are eating, and this allows us to make healthier choices. Although it may seem like a lot of trouble to measure our food, it won’t take long for you to learn how to measure your food visually and then you won’t have to measure everything so carefully.

But until then, it is better to measure our food because even a small difference in portion size can mean a big difference in fat grams and calories.

Today, we are going to start with some hands-on practice weighing and measuring food.

Measuring food

**Present:** Let’s go over a few tips on how to use the cups, spoons, and scale.

**Refer** participants to the handout “Tips for Weighing and Measuring Food”.

**Present:** Use measuring cups and spoons to measure: Solid foods (cooked rice) and Dry ingredients (cereal, sugar).

Fill the cup or spoon so that it is full. Then level it off with a knife.

**Demonstrate:** how to measure and level off the cups and spoons using actual food.

**Present:** To measure liquids, use a measuring cup. These cups have markings for milliliters on one side. What is important is that you can see through the cup so you can see what you are measuring against the line. Use this to measure milk, soup, juice.

Pour the liquid in the cup, then read the measurement with the surface of the liquid at eye level while the cup is on a flat surface like a table or counter. If you read it from above the surface, it may not be accurate.

**Present:** Use a small food scale to measure items such as meat, cheese, bread.

Remember even a small amount of extra food can over a few days greatly increase the amount of fat and calories you eat.

Weighing meats *after* they are cooked is important because they lose about a quarter of their weight during cooking.

Guessing portion size and fat quantities

**Present:** Many people do not bother weighing and measuring their food because they think they already know how much they eat. Yet most of us are surprised when we actually measure our food; our eyes can trick us.

Let’s test how accurate we are at measuring the fat and calories of several of our favorite food.

**Set out** 3-5 examples of common high fat foods (try to use ones that have been mentioned by the group previously).

**Present:** Here are some common high-fat foods. We are going to guess the portion sizes and the fat content of each.

**Refer** the participants to the “Guess What?” handout.

ON the handout, write the name of each food. Then under “Guess” write how much you think there is. Use the measurements we have just talked about: cups, tablespoons, teaspoons.

Then write how many grams of fat you think each food has.

We are just guessing. No cheating. No looking at the “Fat and Calorie Counter”.

**Give** the participants a few minutes to write their guesses.

When they are done, ask them to **share** their guess of the amount of each food, one at a time. If possible, have a volunteer come up to measure the amounts.

**Reveal** the amounts of fat and calories in each food.

**Ask:** Were you surprised by the actual amounts?

**Open responses.**

Even small mistakes in estimating the amounts can make a big difference in the fat and calories you eat each day.

**Ask:** Does this demonstration make you think twice about a measuring a food item, instead of just estimating?

**Open responses.**

**Present:** Eventually you will get better at judging food amounts, but until then, let’s measure and practice estimating our food portions.

Hidden fat revealed

**Present:** Last week we talked about the fact that most of the fat we eat is hidden (70%).

**Ask:** Do you remember some examples of food with hidden fat.

**Open responses.**

**Offer:** Marbling in meats, baked products, sauces, salad dressings, batter on fried foods.

Let me show you what the fat in these foods would look like if we could put it all together on a teaspoon.

**Show** the plate or bag with the appropriate amount of butter or shortening. Show the class how many teaspoons of fat each food item contains.

**Present:** The amount of fat in food is not always obvious. Being able to identify the types of food that are high in fat is an important step in helping us reduce the amount of fat we’re eating.

*Part 3: Three ways to eat less fat and fewer calories (20 minutes)*

Methods for eating less fat and fewer calories

Now that we have discussed how to recognize high-fat foods, how can we use this information to actually eat less fat and fewer calories?

**Present:** There are 3 ways to eat less fat and fewer calories:

1. Eat high-fat and high-calorie foods less often.
2. Eat smaller amounts of high-fat and high-calorie foods.
3. Eat low-fat and low-calorie foods instead.

**Ask:** Let’s take the first way. How might we eat high-fat and high-calorie foods less often? Has anyone started doing this?

**Open responses.**

**Offer:** Eating French fries only once a week instead of every day.

**Ask:** How about eating smaller amounts of these foods? Does anyone have an example they can share?

**Open Responses.**

**Offer:** What about sharing the same amount of French fries with a friend or family member?

**Open responses.**

**Ask:** What about eating low-fat or low-calorie foods instead of high-fat and high-calorie foods? Has anyone tried this?

**Open responses.**

**Offer:** Nonfat creamer in coffee instead of Rainbow. Low fat frozen yogurt instead of ice cream.

Menu makeover

**Refer** participants to the “Menu Makeover” handout.

**Present:** This paper has examples of small changes that make a big difference in the number of calories and fat grams saved. These are not menus for you to follow, just examples. Remember that low fat and fat free foods can still have lots of calories in them if they have a lot of sugar.

**Ask:** Does anyone see a food item on the high-fat side that they like but could switch to a food on the low-fat side?

Are there ways to eat a less fatty meal even at your favorite fast food place?

**Open responses.**

**Ask:** Does anyone have an example of a change they made where you ate a low-fat food instead of a high-fat food?

**Open responses.**

**Offer** (if needed): Maybe you could have your sandwich without cheese, or have baked chips instead of fried foods.

**Remind:** There are no forbidden foods. You can just eat a smaller amount of a high-fat food. Remember there are 3 ways to eat less fat and fewer calories.

**Present:** Managing your fat gram goal is a lot like managing your money. You don’t want to spend more money than you have. And just like you keep track of how much money you have left, you can keep track of how many fat grams you have until you reach your fat gram goal.

Knowing how many fat grams you ate already helps you plan for later meals. If you know that you will probably eat a higher number of fat grams at a particular meal, you can reduce the number of fat grams at other meals to still meet your fat gram goal.

*Part 4: Wrap up and to-do list (10 minutes)*

**Present:** For next week, try to eat less fat. Remember the 3 ways we talked about today: Eat high-fat food less often. When you do eat it, eat a smaller amount. If you can eat a low-fat food without a lot of sugar instead.

**Refer:** participants to the “To do next week” handout.

Take a minute to write down 5 foods that you eat that are high in fat. They should be foods you eat regularly (not birthday cake, for example).

Circle one of the 5 foods, and choose one of the 3 ways we discussed today to plan how you will reduce your fat intake from that food: Eat it less often, eat it in smaller amounts, substitute something lower in fat and calories.

Think about and write down any challenges you think you might face in doing this.

Closing

**Summarize the key points. Today we learned how to:**

- Weigh and measure foods to get an accurate portion size. Weighing and measuring foods makes it easier to figure out the fat grams and calories in each food that we eat.
- Estimate the fat and calorie content in common foods.
- Choose one of three ways to reduce how much fat and how many calories we eat.
- Make a plan to eat less fat.

**Close:** As you begin your third week, do your best to keep tracking what you eat, and to pay attention to the amount of fat and calories in your foods. Remember, some fat is hidden.

**Ask**: Are there any questions?

**Answer questions.**

**Dismiss the group.**

**After the session:**

- In each participant’s “Food and Activity Tracker” from Session 2, write notes on successes (positive reinforcement) and recommend strategies for improvement. Limit your recommendations to the recording of foods, keeping a running total and how close they are coming to fat gram goals.
