# Supplementary material for: Emirates Heart Health Project (EHHP): A protocol for a stepped-wedge family-cluster randomized-controlled trial of a health-coach guided diet and exercise intervention to reduce weight and cardiovascular risk in overweight and obese UAE nationals
Source: PLoS One. 2023 Apr 10;18(4):e0282502. doi: 10.1371/journal.pone.0282502 (PMC10085020; doi:10.1371/journal.pone.0282502)
Supplement: S11 Appendix — (DOCX) [file pone.0282502.s011.docx]

**الجلسة 3:** تناول كميات أقل من الدهون والسعرات الحرارية

**الأهداف:**

بحلول نهاية الجلسة 3 ، سيكون المشاركون قادرين على:

- وزن وقياس الأطعمة.
- تقدير كمية الدهون والسعرات الحرارية في الطعام
- شرح ثلاث طرق لتناول كميات قليلة من الدهون والسعرات الحرارية.
- وضع خطة لتناول كميات قليلة من الدهون والسعرات الحرارية للأسبوع المقبل.

**المواد:**

- نشرة توزيعية المشاركين
- متتبع الطعام والنشاط للجلسة 3. مسمية برقم الجلسة الحالي. خذ بعض النسخ الاضافية
- متتبع الطعام والنشاط للجلسة الأولى مع ملاحظاتك لكل مشارك
- عداد الدهون والسعرات الحرارية
- بطاقة الأسماء
- لوح أبيض (سبورة) مع قلم
- ميزان
- أكواب قياس ، ملاعق ،ميزان الطعام
- نماذج الطعام
- حاول اختيار الأطعمة التي قال المشاركون إنها جزء من نظامهم الغذائي (المعكرونة والأرز واللحوم والبطاطس المقلية)
- قم بوزن الأطعمة مقدمًا ، وسجل الوزن على شريط وقم بوضعه في وسط حاوية الطعام.
- 3 ملاعق صغيرة من السمن الناعم في كأس بلاستيك (12 غرام من الدهون)
- 4 ملاعق كبيرة زيت في وسط وعاء طهي الطعام
- 4 ملاعق كبيرة من الزيت في وعاء صغير
- 1 ¾ كوب مكرونة
- 3 أكواب فشار بنكهة الزبدة (29 جم من الدهون)
- 1 ½ كوب لبن كامل الدسم في وعاء (12 غراما من الدهون)
- 3 أونصات همبرغر مطهو (19 جم من الدهون)
- 4 أونصات جبنة شيدر (28 جرام سمين)
- علب فارغة من رقائق الشيبس ، المكسرات ،الكعك\الكيك
- زبدة: 1 ملعقة صغيرة = 4 غرامات من الدهون ، وصباع واحد = 96 غراما من الدهون

**قبل ان تبدأ:**

- اعرض الفيديو.
- قم بمراجعة أهداف الجلسات.
- قم بمراجعة عرض الفصل
- تأكد من أن لديك جميع المواد والنشرات التي تحتاجها.

**نظرة عامة:**

الجلسة رقم 3 عن تناول كميات أقل من الدهون واكتساب عادة جديدة وهي وزن الأطعمة وقياسها لتحديد كمية الطعام الذي يتم استهلاكه أو تناوله بدقة أكبر. نتحدث أيضا عن ثلاث طرق لتناول كميات أقل من الدهون والسعرات الحرارية ، وسوف يتم استخدامها لوضع خطة لتناول طعام أكثر صحة للأسبوع القادم.

هناك 4 أجزاء:

الجزء 1: التقدم والمراجعة الأسبوعية (10 دقائق)

1. مراجعة المعلومات من الجلسة الأخيرة.

2. إجراء مناقشة حول نجاحات المشاركين وتحدياتهم وأسئلتهم منذ الجلسة الأخيرة.

الجزء 2: الوزن والقياس (20 دقيقة)

1. قيادة المشاركين عن طريق جلسة تدريبية باستخدام:

- أكواب وملاعق القياس للمواد الصلبة
- كوب قياس للسوائل
- ميزان للمواد الصلبة.

2. سيقوم المشاركون بتخمين أحجام الطعام ومحتوى الدهون ، ثم التحقق باستخدام أدوات القياس وغيرها.

3. سيتم عرض محتوى الدهون الفعلي لهم و مقارنته بالزبدة في الطبق.

الجزء 3: ثلاث طرق لتناول كميات أقل من الدهون والسعرات الحرارية (20 دقيقة)

1. تناول الدهون بعدد مرات أقل

2. تناول الدهون بكميات أقل

3. استبدال الأطعمة بقليلة الدسم أو قليلة السعرات الحرارية.

الجزء 4: الخاتمة وقائمة المهام (10 دقائق)

**الرسائل الرئيسية:**

- معرفة الحجم هو المفتاح لحساب كمية الدهون بدقة.
- الحساب الدقيق للدهون هو المفتاح للبقاء ضمن كمية الدهون المناسبة لك.
- البقاء ضمن كمية الدهون المناسبة لك هو المفتاح لفقدان الوزن.
- قياس طعامك هو وسيلة واحدة لمعرفة حجم الطعام الخاص بك.
- يمكنك معرفة مقدار الدهون وعدد السعرات الحرارية الموجودة في الطعام.

يتطلب تناول كميات أقل من الدهون اتخاذ قرار بتناول الأطعمة التي تحتوي على نسبة عالية من الدهون وعالية السعرات الحرارية بعدد مرات أقل وبكميات أقل واستبدالها بالأطعمة التي تحتوي على نسبة منخفضة من الدهون وا السعرات الحرارية.

**عرض الفصل الدراسي**

الجزء 1: التقدم والمراجعة الأسبوعية (10 دقائق)

**توزيع** المنشورات

**جمع** "متتبعي الطعام والنشاط الجلسة 2" بعد الجلسة.

**مراجعة** القواعد الأساسية ، إذا لزم الأمر.

**ناقش** نجاحات المشاركين والصعوبات التي واجهتهم في تحقيق أهدافهم في الأسبوع الماضي.

**حاضر**: تحدثنا في الأسبوع الماضي عن أهداف كمية الدهون ، و السعرات الحرارية الإجمالية التي نتناولها. في خلال الأسبوع الماضي ، كان هدفكم هو وزن انفسكم كل بضعة أيام وأن تدونوا كل ما تناولتم وشربتم. تحدثنا أيضًا عن التعرف على فكرة قياس الاطعمة .

**اسأل**: هل قمتم بقياس اوزانكم في المنزل؟ ما هو شعوركم تجاه أهدافكم وتقدمكم حتى الآن؟

**تذكير**: قد يظهر الميزانين أوزان مختلفة قليلاً ، ولكن يجب أن يكون النمط العام إذا كنت تستخدم نفس الميزان متشابهاً. إذا فقدت الوزن ، فيجب أن تتغير الأرقام بنفس المقدار على مقياس منزلك وفي اجتماعاتنا.

**افتح المجال للرد.**

**اسأل:**

كيف كان استخدام "عداد الدهون والسعرات الحرارية"؟

هل كنت قادرًا على الاحتفاظ بمجموع الدهون الكلي؟

ما مدى قربك من تحقيق أهداف بالنسبة لكمية الدهون؟

ما هي التغييرات الإيجابية التي قمت بها خلال هذا الأسبوع؟

ما الصعوبات التي واجهتك؟

**معالجة** أي أسئلة أو صعوبات.

**حاضر:** هذا الأسبوع سوف:

- نبين لك كيفية وزن وقياس الطعام لتحديد الحجم وكمية الدهون.
- نتعلم كيفية تقدير محتوى الدهون في الأطعمة الشائعة.
- نناقش 3 طرق لتناول كميات أقل من الدهون والسعرات الحرارية.
- نضع لك خطة لتناول كميات أقل من الدهون.

الجزء 2: الوزن والقياس (20 دقيقة)

**اسأل:** كم منكم قد أولي اهتمامًا أكبر بأحجام منذ أن بدأنا هذا البرنامج؟ كيف أدى تتبع ما تأكله إلى تغيير طريقة تناولكم للطعام؟

**افتح المجال للرد**

**حاضر:** حجم الجزء مهم. لذلك بالإضافة إلى قراءة الملصقات للأطعمة المعلبة ، نشجعك على استخدام أكواب وملاعق القياس وميزان الطعام. يعد وزن الأطعمة وقياسها خطوة مهمة لمعرفة ما نتناوله ، وهذا يسمح لنا باتخاذ خيارات صحية. على الرغم من أنه قد يبدو من صعبا ، إلا أن الأمر لن يستغرق وقتًا طويلاً لتتعلم كيفية القياس بصريًابعد ذلك لن تضطر إلى قياس كل شيء بعناية فائقة.

ولكن حتى ذلك الحين ، من الأفضل قياس طعامنا لأنه حتى اختلاف بسيط في حجم الجزء يمكن أن يعني فرقًا كبيرًا في غرامات الدهون والسعرات الحرارية.

اليوم ، سنبدأ ببعض التمارين العملية لقياس الطعام.

قياس الطعام

**حاضر:** دعنا نراجع بعض النصائح حول كيفية استخدام الـأكواب والملاعق والميزان.

قم بإحالة المشاركين إلى منشور"نصائح لوزن الطعام وقياسه".

**حاضر**: استخدم أكواب وملاعق القياس لقياس: الأطعمة الصلبة (الأرز المطبوخ) والمكونات الجافة (الحبوب والسكر).

املأ الكوب أو الملعقة بحيث تكون ممتلئة. ثم ساوي الكوب بالسكين.

**وضح:** كيفيةالقياس باستخدام الطعام الفعلي.

**حاضر:** لقياس السوائل ، استخدم كوب القياس. هذه الأكواب لها علامات للملليتر على جانب واحد. المهم هو أنه يمكنك رؤية الكأس حتى تتمكن من رؤية ما تقيسه مقابل الخط. استخدم هذا لقياس الحليب والشوربة والعصير.

صب السائل في الكوب ، ثم اقرأ القياس من سطح السائل على مستوى العين بينما يكون الكوب على سطح مستو مثل طاولة. إذا قرأته من فوق السطح ، فقد لا تكون القراءة دقيقة.

حاضر: استخدم مقياس طعام صغير لقياس العناصر مثل اللحوم والجبن والخبز.

تذكر أنه حتى كمية صغيرة من الطعام الإضافي يمكن أن تزيد بشكل كبير كمية الدهون والسعرات الحرارية التي تتناولها على مدى بضعة أيام.

يعد وزن اللحوم بعد طهيها أمرًا مهمًا لأنها تفقد حوالي ربع وزنها أثناء الطهي.

تخمين حجم الجزء وكميات الدهون

**حاضر:** كثير من الناس لا يهتمون بقياس أطعمتهم لأنهم يعتقدون أنهم يعرفون بالفعل مقدار ما يأكلون. ومع ذلك ، فإن معظمنا يفاجأ عندما نقيس طعامنا بالفعل ؛ عيوننا يمكن أن تخدعنا.

دعونا نختبر مدى دقتنا في قياس الدهون والسعرات الحرارية للعديد من الأطعمة المفضلة لدينا.

حضر 3-5 أمثلة على الأطعمة عالية الدهون الشائعة (حاول استخدام تلك التي ذكرتها المجموعة سابقًا).

حاضر: إليكم بعض الأطعمة الشائعة عالية الدهون. سنخمن أحجام الأجزاء والمحتوى الدهني لكل منها.

اطلب من المشاركين الرجوع إلى منشور"خمن ماذا؟"

على المنشور ، اكتب اسم كل طعام. ثم تحت كلمة "خمن" اكتب'كم تعتقد المقدار؟”. استخدم القياسات التي تحدثنا عنها قبل قليل: الأكواب وملاعق الطعام.

ثم اكتب عدد جرامات الدهون التي تعتقد أن كل طعام يحتوي عليها.

هذا مجرد تخمين. دون غش. لا تبحث في "عداد السعرات الحرارية والدهون".

امنح المشاركين بضع دقائق لكتابة تخميناتهم.

عند الانتهاء من ذلك ، اطلب منهم مشاركة تخمينهم بشأن كمية كل طعام . إذا كان ذلك ممكنًا ، اطلب من أحد المتطوعين لقياس الكميات.

**اكشف** عن كميات الدهون والسعرات الحرارية في كل ألأطعمة.

**اسأل:** هل تفاجئتم بالكميات الفعلية؟

**افتح المجال للرد.**

حتى الأخطاء الصغيرة في تقدير الكميات يمكن أن تحدث فرقًا كبيرًا في الدهون والسعرات الحرارية التي تتناولها يوميًا.

**اسأل:** هل هذا التوضيح يجعلك تفكر مرتين في قياس عنصر غذائي ، بدلاً من مجرد تقدير؟

**افتح المجال للرد.**

**اسأل:** هل التوضيح يجعلكم تفكرون مرتين في قياس الأطعمة بدلاً من مجرد تقدير الكميات؟

**افتح المجال للرد.**

**حاضر:** سوف تصبحون أفضل

**حاضر:** في النهاية ستتحسن في تقدير كميات الطعام ، ولكن حتى ذلك الحين ، دعونا نقيس ونمارس تقدير أجزاء الطعام الخاصة بنا وممارستها بشكل عملي.

كشف الدهون الخفية

**حاضر:** في الاسبوع الماضي تحدثنا عن حقيقة أن معظم الدهون التي نأكلها مخفية (70٪)

**اسأل:** هل تتذكروا بعض الأمثلة للأطعمة التي تحتوي على الدهون المخفية

**افتح المجال للرد**

**اعرض:**  اللحوم الدهنية ، المنتجات المخبوزة ، الصلصات ، صلصات السلطة ، خليط الأطعمة المقلية.

اسمحوا لي أن أريكم كيف ستبدو الدهون في هذه الأطعمة إذا وضعناها معًا في ملعقة صغيرة.

**احضر** طبق به كمية مناسبة من الزبدة أو السمنة. أري الفصل عدد ملاعق الدهون التي تحتوي عليها كل مادة غذائية.

**حاضر**: كمية الدهون في الطعام ليست واضحة دائما. تعد القدرة على تحديد أنواع الأغذية الغنية بالدهون خطوة مهمة في مساعدتنا على تقليل كمية الدهون التي نتناولها.

الجزء 3: ثلاث طرق لتناول كميات أقل من الدهون والسعرات الحرارية (20 دقيقة)

الآن وبعد أن ناقشنا كيفية التعرف على الأطعمة الغنية بالدهون ، كيف يمكننا استخدام هذه المعلومات لتناول كميات أقل من الدهون والسعرات الحرارية ؟

**حاضر:** هناك 3 طرق لتناول كميات أقل من الدهون والسعرات الحرارية الأقل:

1. تناول الأطعمة الغنية بالدهون والسعرات الحرارية بعدد مرات أقل.

2. تناول كميات أقل من الأطعمة الغنية بالدهون والعالية بالسعرات الحرارية.

3. استبدال الأطعمة الغنية بالدهون والسعرات الحرارية بأطعمة قليلة الدسم وقليلة السعرات الحرارية .

**اسأل**: لنأخذ الطريقة الأولى. كيف يمكن أن نتناول الأطعمة الغنية بالدهون والسعرات الحرارية بعدد مرات أقل؟ وهل بدأ أي شخص منكم بالقيام بذلك؟

**افتح المجال للرد**

قدم مثال: تناول البطاطس المقلية مرة واحدة فقط في الأسبوع بدلاً من كل يوم.

اسأل: ماذا عن تناول كميات أقل من هذه الأطعمة؟ هل لدى أي شخص مثال يمكنه مشاركته؟

افتح المجال للرد

اسال : ماذا عن مشاركة نفس القدر من البطاطس المقلية مع صديق أو أحد أفراد الأسرة؟

افتح المجال للرد

اسأل: ماذا عن تناول الأطعمة قليلة الدسم أو منخفضة السعرات الحرارية بدلاً من الأطعمة عالية الدهون وعالية السعرات الحرارية؟ هل هناك اي احد جرب هذا؟

افتح المجال للرد

اعرض مثال: كريمات خالية من الدهون في القهوة بدلاً من الحليب المبخر(الرينبو). زبادي مجمد قليل الدسم بدلاً من الآيس كريم.

تغييرات قائمة الطعام

اطلب من المشاركين الرجوع إلى منشور "تغييرات في القائمة"

**حاضر:** تحتوي هذه الورقة على أمثلة للتغييرات الصغيرة التي تحدث فرقًا كبيرًا في عدد السعرات الحرارية وكمية الدهون . هذه ليست قوائم يجب عليك اتباعها ،انها مجرد أمثلة فقط. تذكر أن الأطعمة قليلة الدهون و الخالية من الدهون يمكن أن تحتوي على الكثير من السعرات الحرارية إذا كانت تحتوي على الكثير من السكر.

**اسأل:** هل يرى أي شخص طعاما من أطعمته المفضلة في الجانب الذي يحتوي على نسبة عالية من الدهون و يمكن أن يستبدله بأطعمة من جانب قليلة الدهون؟هل هناك طرق لتناول وجبة قليلة الدسم في مكانك المفضل للوجبات السريعة؟

افتح المجال للرد

اسأل: هل لدى أي شخص مثال على التغيير الذي أجراه حين تناول طعامًا قليل الدسم بدلاً من تناول طعام يحتوي على نسبة عالية من الدهون؟

افتح المجال للرد

**قل** (إذا لزم الأمر): ربما يمكن أن تتناول شاندويتش بدون جبن ، أو رقائق بطاطس مخبوزة بدلاً من المقلية.

**تذكير:** لا توجد أطعمة ممنوعة. يمكنك فقط تناول كمية أقل من الأطعمة الغنية بالدهون. تذكر أن هناك 3 طرق لتناول كميات أقل من الدهون والسعرات الحرارية.

**حاضر:** إدارة هدف كمية الدهون الخاص بك يشبه إلى حد كبير إدارة أموالك. لا تريد إنفاق أموال أكثر مما لديك. ومثلما تتعقب كمية الأموال المتبقية ، يمكنك تتبع عدد الدهون التي لديك حتى تصل إلى هدفك من الدهون.

معرفة عدد غرامات الدهون التي تناولتها يساعدك على التخطيط لوجباتك التي تليها. إذا كنت تعلم أنك على الأرجح ستأكل عددًا أكبر من الدهون في وجبة معينة ، فيمكنك تقليل كمية غرامات الدهون في الوجبات الأخرى لتستمر في تحقيق هدفك.

الجزء 4: الخاتمة وقائمة المهام (10 دقائق)

**حاضر:** للأسبوع المقبل ، حاول أن تتناول كميات أقل من الدهون. تذكر الطرق الثلاث التي تحدثنا عنها اليوم: تناول الأطعمة الغنية بالدهون بشكل أقل. و تأكل كمية أصغر.يمكنك تناول طعام قليل الدسم وقليل السكر.

**اطلب** من المشاركين الرجوع الى منشور "المهام التي يجب القيام بها في الأسبوع المقبل".

خذ دقيقة لتدوين 5 أطعمة تأكلها غنية بالدهون. يجب أن تكون الأطعمة التي تتناولها بانتظام (على سبيل المثال ليس كيكة عيد ميلاد ).

ضع دائرة حول واحد من الأطعمة ال5، واختر إحدى الطرق الثلاث التي ناقشناها اليوم للتخطيط لكيفية تقليل كمية الدهون التي تتناولها فيالطعام الذي اخترته: تناولها بعدد مرات أقل ، تناولها بكميات أقل ، واستبدلها بقليلة الدهون والسعرات الحرارية.

فكر في أي تحديات تعتقد أنك قد تواجهها أثناء القيام بذلك.

الخاتمة

**لخص النقاط الرئيسية. اليوم تعلمنا كيفية:**

- وزن وقياس الأطعمة للحصول على حجم جزء دقيق. إن وزن الأطعمة وقياسها يجعل من السهل معرفة غرامات الدهون والسعرات الحرارية في كل طعام نتناوله.
- تقدير محتوى الدهون والسعرات الحرارية في الأطعمة الشائعة.
- اختر واحدة من ثلاث طرق لتقليل كمية الدهون وعدد السعرات الحرارية التي نتناولها.
- ضع خطة لتناول كميات أقل من الدهون.

**اختم :** عندما تبدأ أسبوعك الثالث ، ابذل قصارى جهدك لمتابعة ما تأكله ، ولاحظ مقدار الدهون والسعرات الحرارية في الأطعمة الخاصة بك. تذكر ،أن بعض الدهون مخفية.

اسأل: هل هناك أي أسئلة؟

**أجب عن الأسئلة.**

**اسمح للمجموعة بالمغادرة.**

**بعد الجلسة:**

راجع متتبع الطعام والنشاط لكل مشارك من الجلسة 2 ، اكتب ملاحظات عن النجاحات (التعزيز الإيجابي) واقترح التوصيات للتحسن. احصر توصياتك في تسجيل الأطعمة ومدى القرب من تحقيق الأهداف
