# Supplementary material for: Emirates Heart Health Project (EHHP): A protocol for a stepped-wedge family-cluster randomized-controlled trial of a health-coach guided diet and exercise intervention to reduce weight and cardiovascular risk in overweight and obese UAE nationals
Source: PLoS One. 2023 Apr 10;18(4):e0282502. doi: 10.1371/journal.pone.0282502 (PMC10085020; doi:10.1371/journal.pone.0282502)
Supplement: S12 Appendix — (DOCX) [file pone.0282502.s012.docx]

**Session 4: Healthy eating**

**Objectives/goals:**

By the end of session 4, the participants will be able to-

- Explain the health benefits of eating less fat and fewer calories.
- Describe the MyPlate food guide and its recommendations, including how to reduce fat and calories in our diet.
- Compare and contrast the MyPlate guidelines with participants’ eating habits.
- List ways to replace high-fat and high-calorie foods with low-fat and low-calorie foods.
- Explain the importance of eating plenty of whole grains, vegetables, and fruits, while staying within fat gram goals.
- Explain the importance of eating foods from all groups of MyPlate and of eating a variety of foods from within each group.
- Explain why a balanced diet is beneficial to health.
- Explain why eating the same foods over and over is not the best strategy for long term success.

**Materials:**

- Participant handouts for Session 4
  - Ways to eat healthy
  - MyPlate
  - MyPlate: Food choices
  - MyPlate: Low-fat and Low-calorie choices
  - Rate your plate
  - Eating as MyPlate suggests
  - My food substitutions
  - To-Do for next week
- Food and Activity Trackers for Session 4
- Name tags
- Whiteboard and marker
- Measuring cups, spoons, ruler, food scale
- Food for demonstration

**Before you begin:**

- View the video.
- Review the objectives/goals of the sessions.
- Review the classroom presentation.
- Make sure you have all materials and handouts you need.

**Overview:**

Session 4 introduces the MyPlate food guide and why following the MyPlate guide is a way of eating healthy. This session will be interactive: participants will compare what they eat with the foods recommended by MyPlate. They will also share ideas about how to bring their eating habits more in line with the MyPlate guidelines.

Session 4 has four parts:

- Part 1: Weekly progress and review (10 minutes)
  1. Review the information from the last session.
  2. Lead a discussion about participants’ successes, challenges and questions since the last session.
- Part 2: Healthy ways to eat (10 minutes)
  1. Healthy eating involves not only **what** people eat, but also **how** they eat.
  2. Discuss ways to make healthier food choices.
- Part 3: MyPlate (30 minutes)
  1. Introduce MyPlate and discuss the major food groups.
     - How much of each group should be eaten daily.
     - How to select low-fat alternatives in each group.
  2. Show actual food items in appropriate serving sizes.
     - Discuss which high-fat and high-calorie foods fall into each category so they can recognize which foods they should limit.
  3. Participants will practice comparing what they eat (based on their recording in the “Food and Activity Trackers”) with the recommended MyPlate foods. You will ask them to compare the Tracker and the MyPlate model several times in the coming week.
- Part 4: Wrap up and To-Do list (10 minutes)

**Key messages:**

- **Healthy eating is determined both by *what* we eat and *how* we eat.**
- **MyPlate recommends how much people should eat, depending on their sex, age, and level of physical activity.**
- **Replace high-fat or high-calorie foods with healthier foods that include grains, vegetables, and fruits.**
- **Avoid the common tendency to eat the same foods over and over as a means of simplifying keeping track of what we eat. It can lead to trouble when we become bored with these foods and also we do not learn to calculate fat grams and portion sizes so that we can deal with unfamiliar foods when we need to.**

**Classroom presentation**

*Part 1: Weekly progress and review (10 minutes)*

**Distribute** hand-outs and Session 2 Trackers with your notes and recommendations.

**Collect** Session 3 “Food and Activity Trackers” after the session.

**Ask** whether the ground rules are working and whether any changes need to be made.

**Discuss** the group’s successes and difficulties in meeting their goals in the last week.

**Present:** Last week we learned about the importance of measuring our food, and we practiced using measuring spoons, cups and a scale. You guessed portion sizes and the fat and calorie content of common foods and compared them with the measurements. We also discussed different ways of reducing the amount of fat and calories we eat.

**Ask:** What were the three ways we talked about last week to eat less fat and fewer calories?

**Open responses.**

**Offer** (if needed): Eat high-fat and high-calorie foods 1) less often, 2) in smaller amounts or 3) substitute low-fat and low-calorie foods.

**Present:** At the end of the last session I asked you to name 5 high-fat or high-calorie foods that you like to eat. Your homework was to decide how to use the three ways we talked about to reduce your fat and calorie intake from at least one of these foods.

**Ask:** What did you come up with? Did you try any of the 3 ways? What worked, and what did not?

**Open responses.**

**Ask:** How did everyone do measuring food?

**Open responses.**

**Ask:** How are you feeling this week about your goals and what we are here to do?

**Open responses.**

**Present:** This week we will:

1. Discuss why healthy eating is not only *what* we eat, but *how* we eat it.
2. Take a look at something called MyPlate and what it recommends.
3. Compare the MyPlate recommendations and what you are eating now.
4. Find ways to replace high-fat and high-calorie foods with low-fat and low-calorie foods.
5. Discuss the importance of eating plenty of whole grains, vegetables and fruits, while staying within our fat gram goal.

*Part 2: Healthy ways to eat (10 minutes)*

**Present:** In the past few weeks, we have talked about eating less fat and fewer calories. Eating less fat and fewer calories is essential to losing weight. It is also a part of healthy eating in general. Today we are going to look at some other parts of healthy eating using a model called MyPlate and then compare our eating pattern to it. We will then look for ways to improve our eating habits.

The way we eat

**Present:** Healthy eating is determined by *what* we eat and *how* we eat.

**Ask:** In what ways do you think *how* we eat is related to healthy eating?

**Open responses.**

**Refer** to the “Ways to eat healthy” hand out.

**Present:** Eating healthy is not always easy. This handout has some ideas we can use to help us eat healthy.

**Briefly discuss.**

**Ask:** Does anyone have any ideas you would like to share that are not on the paper?

**Open responses.**

**Present:** For more ideas, we can use tools like MyPlate to help us choose healthier foods in portions that are right for you.

The type of food we eat

**Present:** Another part of healthy eating is the type of food we eat. The MyPlate food guide will help you think about what types of food you should eat and in what amounts. The MyPlate model illustrates the five food groups using a plate.

**Refer”** “MyPlate handout”.

**Ask:** How many of you have seen this before?

**Open responses.**

**Ask:** Has anyone used this before to decide what kinds of food to eat? (If so, ask what they learned from it.)

**Open responses.**

*Part 3: MyPlate (30 minutes)*

**Present:** MyPlate is a general guide to healthy eating. It is based on the latest research about nutrition and health.

The plate shows us the relative portion size of each food group we should be eating at our meals. For example, we should be eating more vegetables than rice or meat.

Each person has a slightly different version of MyPlate because it is different for sex, age and level of physical activity. The amounts of each food are given in ounces (for grains and proteins) or cups (for fruits, vegetables and dairy).

**Present:** Let’s take a look at what food groups are included in MyPlate.

**Refer** to the “MyPlate” handout.

**Present:** The food groups are:

- Grains: all foods made from wheat, rice, cornmeal, barley. Examples are bread, pasta, bread.
- Fruits and vegetables: this includes all- fresh, frozen, canned, dried, juiced.
- Dairy: milk, yogurt, cheese.
- Protein foods: meat, poultry, fish, eggs, nuts, beans, seeds.

**Present:** MyPlate is designed to show several healthy eating habits that we should follow:

*Balance the calories in our meals*

- Enjoy our food, but eat less.
- Avoid oversized portions.

*Foods to increase*

- Fruits and vegetables should be half the food on the plate.
- Grains: at least half of your grains should be whole grains.
- Fat-free or low-fat (1%) milk

*Foods to decrease*

- Foods with a lot of salt.
- Drink water instead of sugary drinks like juice or soda.

MyPlate recommends how much people eat, depending on their age, sex, and level of physical activity. This is just a starting point. What your body needs to reach your goal may be different.

(To save time, have examples of various foods measured and set out on the table before this demonstration. Cover them until it is time for the discussion.)

Demonstrate**:** Uncover the food. Show each food group and tell how much is there.


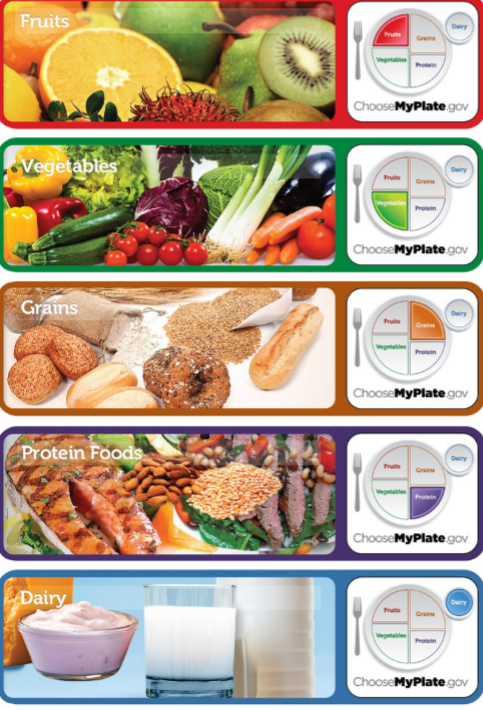


Grains: 4-6 ounces

Vegetables 1.5 – 2.5 cups

Fruit: 1-2 cups

Milk: 2-3 cups

Protein foods 3-6 ounces.

**Present:** MyPlate recommends the number of servings per day of each food group. The number depends on your sex, age, and level of physical activity.

Grains

**Present:** Grains are divided into smaller groups: whole grains and refined grains.

Whole grains contain the entire kernel: whole wheat flouWhr, cracked wheat, brown rice.

Refined grains are milled which removes some parts: white flour, white bread, white rice.

MyPlate recommends that at least half of our grains come from whole grains.

**Ask:** What low-fat foods fit into the grains group?

**Open responses and write them on the white board.**

**Present:** A lot of the fat is added to the grains during cooking or when we serve it. Examples: sauces or cheese.

**Offer** these examples of low-fat grains: one slice of whole wheat bread, one piece of brown bread.

**Ask:** Can anyone think of any high-fat or high-calorie foods in the grains group?

**Open responses.**

**Offer** (if needed): doughnuts, chips, muffins, sugar coated cereal.

**Ask:** Why do you think that chips are high in fat? They are just potatoes, right?

**Open responses.**

**Present:** Chips are usually cooked in fat, usually unhealthy fat. We need to limit foods cooked in fat.

Vegetables

**Ask:** What about vegetables? How can you prepare and serve vegetables without adding too much fat?

**Open responses.**

**Offer** (if needed): green salad with low fat dressing. Steamed, roasted or grilled vegetables. Vegetables with low fat dipping sauce.

**Ask:** And what kinds of vegetables should be limited?

**Open responses.**

**Offer** (if needed): fried vegetables, vegetables in butter, cream or cheese.

**Present:** Usually vegetables are not high in fat but people often add them during cooking or when we serve them.

Fruit

**Ask:** What about fruit? What are low-fat and low-calorie ways to eat fruit?

**Open responses.**

**Offer** (if needed): whole fresh fruits are best. Canned fruit without syrup is also good.

**Ask:** What kinds of fruit should we limit?

**Open responses.**

**Offer** (if needed): Fruits in pastry, juices, fruit sweetened with sugar or syrup.

Dairy

**Ask:** How about the dairy group? What are low-fat and low-calorie options here?

**Open responses.**

**Offer** (if needed): skim or 1% milk, low fat yogurt, low fat cheese.

**Ask:** What kind of milk products are high in fat?

**Offer** (if needed): whole milk, regular cheese.

Proteins

**Ask:** What about low-fat and low-calorie proteins?

**Open responses.**

**Present:** Many meats are high in fat. When we eat red meat, we should choose leaner cuts, trim visible fat and use low-fat cooking methods.

Nuts are included in this category, but the fat in nuts is mostly healthy. Just be careful with the amount.

This group also includes beans, which are not high in fat unless you cook them or serve them with added fat.

**Offer** these examples of lean meats: extra lean ground beef, skinless chicken, fish, eggs.

**Ask:** What are some high-fat and high-calorie proteins we should limit?

How have you been eating?

**Present:** Each food group should be included in what you eat in order to get all the nutrients your body needs.

Now let’s compare how you have been eating with the MyPlate recommendations. Remember, we are trying to make safe and gradual changes. It is okay if we are not eating exactly according to the recommendations, but we are trying to move toward a healthier diet.

**Refer** to participants’ “Food and Activity Tracker” for Session 3 and the “Rate your plate” handout.

**Ask participants to select one day from their “Tracker”.** Then they should check one box for every ½ ounce or ½ cup of each thing that they ate from each group of foods in MyPlate. Tell them not to worry about being exact, this exercise is to get a general idea of how our current diet compares to the MyPlate recommendations.

If this is too difficult, you can ask for a volunteer to share what he or she ate for breakfast, and then have the group work together to “Rate your plate”.

Then have a second volunteer for dinner last night. Etc.

**Ask:** Did the recommended number of servings for each food group in MyPlate match the number you actually ate?

**Open responses.**

**Ask:** For which food group or groups did you exceed the recommended number of servings?

**Open responses.**

**Ask:** For which food group or groups did you have fewer than the recommended number of servings?

**Open responses.**

**Ask:** How could you eat better overall?

**Refer** to the handout “Eating as MyPlate suggests”.

**Ask:** What kinds of food could you eat as a family that would help you as a family to eat better?

**Open responses.**

**Present:** Last week we talked about 3 ways to eat less fat and fewer calories. One of them was to eat low-fat and low-calorie foods instead of high-fat and high-calorie foods. MyPlate and the practice of doing that work together to help you reach your goal.

**Ask:** Can anyone suggest other ways to substitute low-fat and low-calorie food for high-fat and high-calorie foods?

**Open responses.**

**Ask:** How can you reduce fat when you cook at home?

**Offer:** If you have a favorite food you cook at home, we can see how you make it and suggest some healthier substitutions we can try to reduce the amount of fat and calories in it.

*Part 4: Wrap up and to-do list*

To do for next week

**Ask** whether participants have any questions.

**Present:** MyPlate is only one model of healthy eating. The main message here is to eat a variety of foods from all of the MyPlate food groups.

(Do not be too strict about following the recommendations. Many people will feel overwhelmed or frustrated if they feel they have to follow them exactly.)

**Present:** For next week, I want you to:

- Continue to monitor and record your weight and what you eat each day.
- Practice what we learned today. Compare what you eat with the recommendations of MyPlate.

**Summarize** key points:

- **We reviewed some of the benefits of eating less fat and fewer calories.**
- **We introduced the model of MyPlate for healthy eating.**
- **We compared what you normally eat with the MyPlate recommendations.**
- **We listed ways to replace high-fat and high-calorie foods with low-fat and low-calorie foods for each of the food groups.**

**Close:** Now, as you begin your fifth week of the program, do the best you can to make what you eat match the recommendations of MyPlate. You may find that by increasing the amount of vegetables you eat, you will feel more full and satisfied after meals.

Next week we will discuss the changes that you were able to make based on what you have learned so far. We will also start to talk about our physical activity goals.

**Collect** “Food and Activity Trackers” from Session 3.

**Ask** if there are any questions or concerns.

**Address** questions and concerns.

**After the session:**

**Make notes in each participant’s Food and Activity Tracker. Praise successes. Write recommendations if needed. Make a special effort to praise the use of any of the three ways of eating less fat and fewer calories (**less often, smaller amounts, substitution). **Praise them when they stay under or at their fat gram goal.**
