# Supplementary material for: Emirates Heart Health Project (EHHP): A protocol for a stepped-wedge family-cluster randomized-controlled trial of a health-coach guided diet and exercise intervention to reduce weight and cardiovascular risk in overweight and obese UAE nationals
Source: PLoS One. 2023 Apr 10;18(4):e0282502. doi: 10.1371/journal.pone.0282502 (PMC10085020; doi:10.1371/journal.pone.0282502)
Supplement: S13 Appendix — (DOCX) [file pone.0282502.s013.docx]

**الجلسة رقم 4: الأكل الصحي**

**الأهداف:**

في نهاية الجلسة الرابعة ، سيتمكن المشاركون من:

- شرح الفوائد الصحية لتناول كميات أقل من الدهون والسعرات الحرارية.
- وصف دليل طعام "طبقي "وتوصياته ، بما في ذلك كيفية تقليل الدهون والسعرات الحرارية في نظامنا الغذائي.
- مقارنة إرشادات "طبقي " مع عادات الأكل لدى المشاركين.
- ذكر طرق استبدال الأطعمة الغنية بالدهون والسعرات الحرارية العالية بالأطعمة قليلة الدسم و منخفضة السعرات الحرارية .
- شرح أهمية تناول كميات كبيرة من الحبوب الكاملة والخضروات والفواكه مع الحفاظ على أهداف الدهون.
- شرح أهمية تناول الأطعمة من جميع مجموعات" MyPlate طبقي" وتناول مجموعة متنوعة من الأطعمة من داخل كل مجموعة.
- شرح لماذا " أن النظام الغذائي المتوازن مفيد للصحة”.
- شرح لماذا "لا يعتبر تناول الأطعمة نفسها مرارًا وتكرارًا أفضل استراتيجية للنجاح على المدى الطويل”.

**المواد:**

- نشرات المشاركين للدورة 4
  - طرق لتناول طعام صحي
  - طبقي
  - طبقي: خيارات الطعام
  - طبقي: الخيارات قليلة الدسم ومنخفضة السعرات الحرارية
  - تقييم الطبق الخاص بك
  - الأكل كما يقترح دليل الطعام "طبقي"
  - بدائل لطعامي
  - قائمة المهام للأسبوع القادم
- متتبع الطعام والنشاط للجلسة 4
- بطاقات الأسماء
- السبورة و قلم
- أكواب القياس ، الملاعق ، المسطرة ، ميزان الطعام
- طعام للشرح والتوضيح

**قبل ان تبدأ:**

- عرض الفيديو.
- راجع أهداف الجلسات.
- مراجعة عرض الفص التقديمي
- تأكد من أن لديك جميع المواد والنشرات التي تحتاجها.

**نظرة عامة:**

تقدم الجلسة الرابعة دليل طعام طبقي الصحي ولماذا يعد اتباع دليل طبقي الصحي وسيلة لتناول الطعام الصحي. ستكون هذه الجلسة تفاعلية: سيقوم المشاركون بمقارنة ما يتناولونه بالأطعمة التي أوصى بها طبقي الصحي. سيقومون أيضًا بمشاركة الأفكار حول كيفية جعل عاداتهم الغذائية أكثر توافقا مع إرشادات طبقي الصحي.

تتكون الجلسة الرابعة من أربعة أجزاء:

الجزء 1: التقدم الأسبوعي والمراجعة (10 دقائق) .

1. مراجعة المعلومات من الجلسة الأخيرة.
2. مناقشة حول نجاحات المشاركين وتحدياتهم وأسئلتهم منذ الجلسة الأخيرة.

الجزء 2: طرق صحية لتناول الطعام (10 دقائق)

1. الأكل الصحي لا يشمل فقط ما يأكله الناس ، ولكن أيضًا كيف يأكلون.

2. ناقش طرق اتخاذ خيارات غذائية صحية.

الجزء 3: طبقي الصحي (30 دقيقة)

1. أعرض طبقي وناقش المجموعات الغذائية الرئيسية.

- كم تحتاج من كل مجموعة يوميا.
- كيفية اختيار بدائل قليلة الدسم في كل مجموعة.

2. عرض العناصر الغذائية الفعلية في أحجام الوجبات المناسبة.

ناقش الأطعمة التي تحتوي على نسبة عالية من الدهون والسعرات الحرارية في كل فئة حتى يتمكنوا من التعرف على الأطعمة التي يجب التقليل منها

3. سوف يتدرب المشاركون على مقارنة ما يتناولونه (بناءً على تسجيلهم في "متتبعي الأغذية والنشاطات") مع الأطعمة الموصى بها من طبقي الصحي. ستطلب منهم مقارنة المتتبع وطبقي الصحي عدة مرات في الأسبوع القادم.

الجزء 4: اختتام وقائمة المهام (10 دقائق)

**الرسائل الرئيسية:**

- **الأكل الصحي يتحدد حسب ما نأكل وكيف نأكل.**
- **توصي “ طبقي MyPlate " بالكمية التي يجب أن يتناولها الأشخاص ، بناءً على نوع الجنس والعمر ومستوى النشاط البدني.**
- **استبدل الأطعمة الغنية بالدهون أو عالية السعرات الحرارية بالأطعمة الصحية التي تشمل الحبوب والخضروات والفواكه.**
- **تجنب النزعة الشائعة لتناول نفس الأطعمة مرارًا وتكرارًا كوسيلة لتبسيط تتبع ما نأكله. يمكن أن يؤدي ذلك إلى حدوث مشكلات عندما نشعر بالملل من هذه الأطعمة وأيضًا لا نتعلم حساب غرامات الدهون وأحجام الأجزاء حتى نتمكن من التعامل مع الأطعمة غير المألوفة عندما نحتاج إلى ذلك.**

**عرض الفصل الدراسي**

الجزء 1: التقدم والمراجعة الأسبوعية (10 دقائق)

قم **بتوزيع** المنشورات ومتتبع الجلسة 2 مع ملاحظاتك وتوصياتك.

**اجمع** "متتبعي الطعام والنشاط"للجلسة الثالثة بعد هذه الجلسة.

**اسأل** ما إذا كانت هناك حاجة إلى إجراء تغييرات في القواعد الأساسية.

**ناقش** نجاحات المجموعة وصعوباتها في تحقيق أهدافها في الأسبوع الماضي.

**حاضر:** في الأسبوع الماضي تعلمنا أهمية قياس طعامنا ، ومارسنا باستخدام ملاعق القياس و أكوابالقياس والميزان. لقد خمنا أحجام الأجزاء ومحتوى الدهون والسعرات الحرارية في الأطعمة الشائعة وقارناها بالقياسات. ناقشنا أيضًا طرقًا مختلفة لتقليل كمية الدهون والسعرات الحرارية التي نتناولها.

**اسأل:** ما هي الطرق الثلاث التي تحدثنا عنها الأسبوع الماضي لتناول كميات أقل من الدهون والسعرات الحرارية؟

**افتح المجال للرد**

اعرض (إذا لزم الأمر): تناول الأطعمة التي تحتوي على نسبة عالية من الدهون وعالية السعرات الحرارية 1) بعدد مرات أقل ، 2) بكميات أصغر أو 3) الأطعمة البديلة قليلة الدسم ومنخفضة السعرات الحرارية.

**حاضر:** في نهاية الجلسة الأخيرة ، طلبت منكم تسمية 5 أطعمة عالية الدهون أو عالية السعرات الحرارية وتود أن تتناولها. كان واجبك هو أن تقرر كيفية استخدام الطرق الثلاث التي تحدثنا عنها لتقليل الدهون والسعرات الحرارية المستهلكة من واحدة على الأقل من هذه الأطعمة.

**اسأل:** ماذا الذي توصلتم اليه؟ هل جربتم أي طريقة من الطرق الثلاث؟ ما الذي نجح وما الذي لم ينجح؟

**افتح المجال للرد**

**اسأل**: كيف قمتم بقياس الطعام؟

**افتح المجال للرد**

**اسأل:** ما هو شعورك هذا الأسبوع حول أهدافك وعن البرنامج؟

**افتح المجال للرد**

**حاضر:** هذا الأسبوع سوف:

1. نناقش لماذا الأكل الصحي ليس فقط ما نأكله ، ولكن كيف نأكله.

2. نلقي نظرة على دليل يسمى طبقي وما ينصح به.

3. نقارن توصيات طبقي وما نتناوله الآن.

4. نبحث عن طرق لاستبدال الأطعمة التي تحتوي على نسبة عالية من الدهون والسعرات الحرارية مع الأطعمة قليلة الدسم وقليلة السعرات الحرارية .

5. نناقش أهمية تناول الكثير من الحبوب الكاملة والخضروات والفواكه ، مع الحفاظ على هدفنا في كمية الدهون.

الجزء 2: طرق صحية لتناول الطعام (10 دقائق)

**حاضر:** في الأسابيع القليلة الماضية ، تحدثنا عن تناول كميات أقل من الدهون والسعرات الحرارية . إن تناول كميات أقل من الدهون وتقليل السعرات الحرارية أمر ضروري لفقدان الوزن. كما أنه جزء من الأكل الصحي بشكل عام. سننظر اليوم في بعض الأجزاء الأخرى من الأكل الصحي باستخدام نموذج يسمى " طبقي" ثم نقارن نمط الأكل به. سنبحث بعد ذلك عن طرق لتحسين عاداتنا الغذائية.

طريقة تناول الطعام

**حاضر**: الأكل الصحي يتحدد حسب نوع الأكل وكيفيته.

**اسأل:**بأي طريقة تعتقد أن كيفية تناول الطعام مرتبط بالأكل الصحي؟

**افتح المجال للرد**

**ارجع** إلى منشور "طرق الأكل الصحي".

**حاضر: ا**لأكل الصحي ليس بالأمر السهل. يحتوي هذا المنشور على بعض الأفكار التي يمكننا استخدامها لمساعدتنا على تناول طعام صحي.

مناقشة لفترة وجيزة.

**اسأل**: هل لدى أي شخص أفكار يرغب في مشاركتها ؟

**افتح المجال للرد**

**حاضر**: لمزيد من الأفكار ، يمكننا استخدام أدوات مثل طبقي لمساعدتنا في اختيار الأطعمة الصحية بكميات مناسبة لك.

نوع الطعام الذي نأكله

حاضر: جزء آخر من الأكل الصحي هو نوع الطعام الذي نتناوله. سيساعدك دليل الطعام في "طبقي" على التفكير في أنواع الأطعمة التي يجب أن تتناولها وبأي كميات. يوضح نموذج"طبقي" المجموعات الغذائية الخمس باستخدام الطبق.

راجع "نشرة "طبقي الصحي".

**اسأل:** كم منكم شاهد هذا من قبل؟

**افتح المجال للرد**

**اسأل:** هل استخدم أحد هذا من قبل لتحديد أنواع الطعام التي يجب تناولها؟ (إذا كان الأمر كذلك ، اسأل ماذا تعلمتم منه).

**افتح المجال للرد**

الجزء 3: "طبقي الصحي" (30 دقيقة)

الحاضر: "طبقي" هو دليل عام للأكل الصحي. يعتمد على أحدث الأبحاث حول التغذية والصحة.

توضح لنا اللوحة الحجم النسبي لحجم كل مجموعة غذائية يجب أن نتناولها في وجباتنا. على سبيل المثال ، يجب أن نتناول المزيد من الخضروات بكمية أكثر من الأرز أو اللحم.

كل شخص لديه نسخة مختلفة قليلا من "طبقي الصحي" لأنه يختلف باختلاف الجنس والعمر ومستوى النشاط البدني. يتم إعطاء كميات كل طعام بالأوقية (للحبوب والبروتينات) أو الأكواب (للفواكه والخضروات ومنتجات الألبان).

**حاضر:** دعنا نلقي نظرة على المجموعات الغذائية المدرجة في "طبقي “.

ارجع إلى منشور"'طبقي ".

**حاضر:** المجموعات الغذائية هي:

- الحبوب: جميع الأطعمة المصنوعة من القمح والأرز ودقيق الذرة والشعير. ومن الأمثلة على ذلك الخبز والمعكرونة والخبز.
- الفواكه والخضروات: ويشمل ذلك الطازج والمجمد والمعلب والمجفف والعصائر.
- منتجات الألبان: الحليب والزبادي والجبن.
- الأطعمة البروتينية: اللحوم والدواجن والأسماك والبيض والمكسرات والبقوليات والبذور

**حاضر:** تم تصميم نموذج "طبقي" لإظهار العديد من عادات الأكل الصحية التي يجب علينا اتباعها:

*موازنة السعرات الحرارية في وجباتنا*

- الاستمتاع بتناول الطعام ، ولكن بكميات أقل.
- تجنب الأحجام الكبيرة.

الأطعمة التي يمكن زيادتها

- يجب أن تكون الفواكه والخضروات في نصف الطبق.
- الحبوب: يجب أن يكون نصف الحبوب على الأقل عبارة عن حبوب كاملة.
- حليب خالي الدسم أو قليل الدسم (1٪)

الأطعمةالتي يجب التقليل منها

- الأطعمة التي تحتوي على الكثير من الملح.
- شرب الماء بدلاً من المشروبات المحلاة مثل العصائر أو المشروبات الغازية ا.

يوصي طبقي كمية الطعام للاشخاص حسب العمر والجنس ومستوى النشاط البدني. هذه مجرد نقطةبداية. ما يحتاجه جسمك للوصول إلى هدفك قد يكون مختلفًا.

(لتوفير الوقت ، حضر أمثلة على الأطعمة المختلفة المقاسة ورتبها على الطاولة قبل ان تبدأ بهذا العرض التوضيحي. قم بتغطيتها حتى يحين وقت المناقشة).

شرح: اكشف الطعام. أظهر كل مجموعة من المواد الغذائية وقل كم هناك؟.


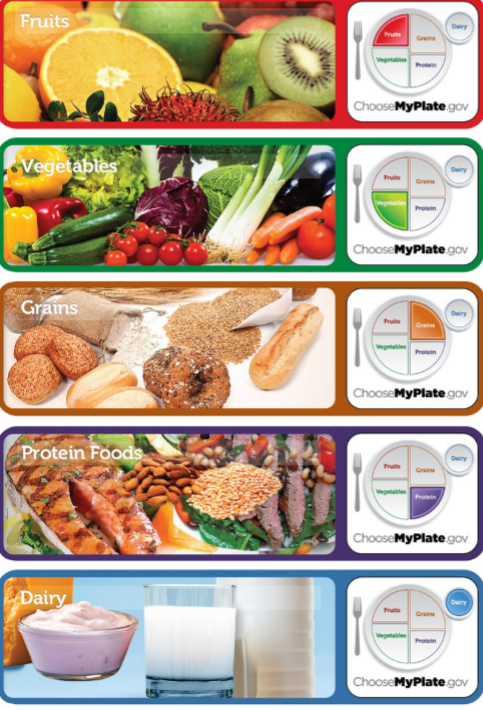


الحبوب: 4-6 أوقية

الخضروات 1.5 - 2.5 كوب

الفاكهة: 1-2 أكواب

الحليب: 2-3 أكواب

الأطعمة البروتينية 3-6 أوقية.

**حاضر:** يوصي نمودج طبقي بعدد الحصص لكل مجموعة غذائية في اليوم . يعتمد العدد على جنسك وعمرك ومستوى نشاطك البدني.

بقوليات

**حاضر:** الحبوب مقسمة إلى مجموعتين: الحبوب الكاملة والحبوب المكررة.

تحتوي الحبوب الكاملة على النواة بأكملها: القمح الكامل ، البرغل ، الأرز البني.

يتم طحن الحبوب المكررة لازالة بعض الأجزاء: الطحين الأبيض والخبز الأبيض والأرز الأبيض.

توصي MyPlate بأن نصف الحبوب على الأقل يجب أن تكون من الحبوب الكاملة.

**اسأل:** ما الأطعمة قليلة الدسم التي تدخل في مجموعة الحبوب؟

**استمع للمشاركات ودونها في السبورة البيضاء.**

**حاضر:** يضاف الكثير من الدهون إلى الحبوب أثناء الطهي أو عند تقديمها. أمثلة: الصلصات أو الجبن.

**قدم** هذه الأمثلة من الحبوب قليلة الدسم: شريحة واحدة من خبز القمح الكامل ، وقطعة واحدة من الخبز البني.

**اسأل:** هل يمكن لأي شخص أن يفكر في أي أطعمة عالية الدهون أو عالية السعرات الحرارية في مجموعة الحبوب؟

**افتح المجال للرد**

**اعرض** (إذا لزم الأمر): دونات ، الرقائق ، الكيك ، الحبوب االمغطاه بالسكر.

**اسأل:** لماذا تعتقد أن رقائق البطاطا غنية بالدهون؟ هي مجرد البطاطس ، أليس كذلك؟

**افتح المجال للرد**

**حاضر:** عادة ما يتم طهي الرقائق في الدهون ، وعادة ما تكون الدهون غير الصحية. نحن بحاجة إلى الحد من الأطعمة المطبوخة في الدهن.

خضروات

**اسأل**: ماذا عن الخضروات؟ كيف يمكنك تحضير الخضار وتقديمها دون إضافة الكثير من الدهون؟

**افتح المجال للرد**

**اعرض** (إذا لزم الأمر): سلطة خضراء مع صلصة قليلة الدسم. الخضار على البخار ، المحمص أو المشوي. خضار مع صلصة تغميس قليلة الدسم.

**اسأل**: وما أنواع الخضروات التي يجب التقليل منها؟

**افتح المجال للرد**

**اعرض** (إذا لزم الأمر): الخضروات المقلية ، والخضروات بالزبدة أو بالكريمة أو الجبن.

**حاضر**: عادة لا تكون الخضروات غنية بالدهون ولكن الناس يضيفونها غالبًا أثناء الطهي أو عندما تقدم لهم.

فاكهة

**اسأل:** ماذا عن الفاكهة؟ ما هي الطرق قليلة الدسم ومنخفضة السعرات الحرارية التي يمكن تناول الفاكهة بها؟

**افتح المجال للرد**

**اعرض** (إذا لزم الأمر): الفواكه الطازجة الكاملة هي الأفضل. الفواكه المعلبة دون شراب هي أيضا جيدة.

**اسأل:** ما هي أنواع الفاكهة التي يجب أن نقلل منها؟

**افتح المجال للرد**

**اعرض** (إذا لزم الأمر):الفواكه اذا كانت في المعجنات والعصائر والفواكه المحلاة بالسكر أو الشراب.

الألبان

**اسأل:** ماذا عن مجموعة الألبان؟ ما هي الخيارات قليلة الدسم والمنخفضة السعرات الحرارية في هذه المجموعة؟

**افتح المجال للرد**

**اعرض** (إذا لزم الأمر): حليب خالي الدسم أو 1٪ ، زبادي قليل الدسم ، جبن قليل الدسم.

**اسأل**: ما نوع منتجات الألبان الغنية بالدهون؟

**اعرض** (إذا لزم الأمر): الحليب كامل الدسم والجبن العادي.

البروتينات

**اسأل:** ماذا عن البروتينات قليلة الدسم و المنخفضة السعرات الحرارية؟

**افتح المجال للرد**

**حاضر:** العديد من اللحوم غنية بالدهون. عندما نأكل اللحم الأحمر ، يجب أن نختار كميات أصغر حجماً ، ونقلل الدهون المرئية

ونستخدم طرق الطهي الصحية "قليلة الدسم”.

تعد المكسرات من ضمن هذه الفئة ، ولكن الدهون في المكسرات صحية في الغالب. فقط كن حذرا مع الكمية.

تحتوي هذه المجموعة أيضًا على حبوب لا تحتوي على نسبة عالية من الدهون ما لم تقم بطهيها أو تقديمها بالدهون المضافة.

**قدم** هذه الأمثلة من اللحوم الخالية من الدهن: لحم بقري مفروم قليل الدسم ، دجاج بدون جلد ، سمك ، بيض.

**اسأل**: ما هي بعض البروتينات عالية الدهون والسعرات الحرارية التي يجب علينا الحد منها؟

كيف كنت تتناول الطعام؟

**حاضر:** يجب تضمين كل مجموعة غذائية في ما نتناوله من أجل الحصول على جميع العناصر الغذائية التي يحتاجها الجسم.

الآن دعونا نقارن طريقة تناولكم للطعام مع توصيات طبقي. تذكروا أننا نحاول إجراء تغييرات آمنة وتدريجية. لا بأس إذا كنا لا نأكل بالضبط وفقا للتوصيات ، لكننا نحاول التحرك نحو اتباع نظام غذائي أكثر صحة.

ارجع إلى "متتبع الطعام والنشاط" الخاص بالمشاركين للجلسة 3 ونشرة "قيم صحنك".

اطلب من المشاركين اختيار يوم واحد من "المتتبع". بعد ذلك ، يجب عليهم تحديد خانة واحدة مقابل كل ½ أونصة أو ½ كوب من كل شيء يأكلونه من كل مجموعة من الأطعمة في “طبقي”. أخبرهم أن لا يقلقوا بشأن كونهم دقيقين ، فهذا التمرين هو الحصول على فكرة عامة عن كيفية مقارنة نظامنا الغذائي الحالي بتوصيات “ طبقي”.

إذا كان هذا أمرًا صعبًا للغاية ، فيمكنك أن تطلب من أحد المتطوعين مشاركة ما تناوله هو أو هي في وجبة الإفطار ، ثم جعل المجموعة تعمل معًا من أجل "تقييم صحنك".

ثم متطوعًا ثانيًا لمشاركة ما تناوله في وجبة العشاء الليلة الماضية. إلخ.

**اسأل:** هل العدد الموصى به من الوجبات لكل مجموعة طعام في " طبقي" يطابق العدد الذي أكلته بالفعل؟

**افتح المجال للرد**

**اسأل**:في أي مجموعة أو مجموعات غذائية تجاوزت عدد الوجبات الموصى بها؟

**افتح المجال للرد**

**اسأل**: في أي مجموعة أو أي من المجموعات الغذائية كان لديك أقل من العدد الموصى به من الوجبات؟

**افتح المجال للرد**

**اسأل**: بشكل عام كيف يمكن أن تأكل بشكل أفضل؟

**ارجع** إلى المنشور "الأكل كما يوصي “طبقي".

**اسأل:** ما هي أنواع الطعام التي يمكن أن تتناولها كعائلة من شأنها أن تساعدك كعائلة على تناول الطعام بشكل أفضل؟

**افتح المجال للرد**

**حاضر:** في الأسبوع الماضي تحدثنا عن 3 طرق لتناول كميات أقل من الدهون والسعرات الحرارية . كان أحدهذه الطرق هو تناول الأطعمة قليلة الدسم والمنخفضة السعرات الحرارية بدلاً من الأطعمة ذات الدسم العالي والعالية السعرات الحرارية. يعمل طبقي وممارسة ذلك لمساعدتك في الوصول إلى هدفك.

**اسأل**: هل يمكن لأي شخص اقتراح طرق أخرى لاستبدال الأطعمة الغنية بالدهون والسعرات الحرارية العالية بالأطعمة قليلة الدسم و المنخفضة السعرات الحرارية ؟

**افتح المجال للرد**

**اسأل:** كيف يمكنك تقليل الدهون عند الطهي في المنزل؟

**اعرض:** إذا كان لديك طعام مفضل تقوم بطبخه في المنزل ، فيمكننا أن نرى كيف يمكنك صنعه واقتراح بعض البدائل الصحية التي يمكننا أن نحاول تقليل كمية الدهون والسعرات الحرارية فيها.

الجزء 4: الخاتمة وقائمة المهام

قائمة المهام للأسبوع المقبل

**اسأل**  إذا كان لدى المشاركون أي أسئلة.

**حاضر:** طبقي هو نموذج واحد فقط من الأكل الصحي. الرسالة الرئيسية هنا هي تناول مجموعة متنوعة من الأطعمة من جميع مجموعات الغذائية.

(لا تكن صارمًا جدًا بشأن اتباع التوصيات. سيشعر الكثير من الناس بالإرهاق أو الإحباط إذا شعروا أن عليهم اتباعها تمامًا.)

**حاضر:** للأسبوع المقبل ، أريدكم أن:

تستمروا في مراقبة وتسجيل أوزانكم والطعام كل يوم.

ممارسة ما تعلمناه اليوم. قارن ما تأكله بتوصياتط طبقي”.

**تلخيص** النقاط الرئيسية:

استعرضنا بعض فوائد تناول كميات أقل من الدهون والسعرات الحرارية.

قدمنا ​​نموذج " طبقي" للأكل الصحي.

قارنا ما تأكله عادة مع توصيات “طبقي”.

أدرجنا طرقًا لاستبدال الأطعمة التي تحتوي على نسبة عالية من الدهون وعالية السعرات الحرارية بأطعمة قليلة الدهون ومنخفضة السعرات الحرارية لكل مجموعة من المجموعات الغذائية.

خاتمة: الآن ، عندما تبدأ أسبوعك الخامس من البرنامج ، ابذل قصارى جهدك لجعل ما تأكله يتوافق مع توصيات“طبقي”. قد تجد أنه من خلال زيادة كمية الخضروات التي تتناولها ، ستشعر بالمزيد من الشبع بعد الوجبات.

سنناقش في الأسبوع القادم التغييرات التي تمكنتم من إجرائها بناءً على ما تعلمنا حتى الآن. سنبدأ أيضًا في التحدث عن أهداف نشاطنا البدني.

اجمع "متتبع الطعام والنشاط" من الجلسة 3.

اسأل إذا كان هناك أي أسئلة أو مخاوف.

معالجة الأسئلة والمخاوف.

بعد الجلسة:

قم بتدوين ملاحظات في برنامج متتبع الطعام والنشاط الخاص بكل مشارك. مدح النجاحات. اكتب التوصيات إذا لزم الأمر. بذل جهدا خاصا للاشادة باستخدام أي من الطرق الثلاث لتناول كميات أقل من الدهون والسعرات الحرارية (بشكل أقل ، كميات أصغر ، واستبدال). مدحهم عندما يبقون تحت أو في هدف كمية الدهن الخاصة بهم.
