# Supplementary material for: Emirates Heart Health Project (EHHP): A protocol for a stepped-wedge family-cluster randomized-controlled trial of a health-coach guided diet and exercise intervention to reduce weight and cardiovascular risk in overweight and obese UAE nationals
Source: PLoS One. 2023 Apr 10;18(4):e0282502. doi: 10.1371/journal.pone.0282502 (PMC10085020; doi:10.1371/journal.pone.0282502)
Supplement: S14 Appendix — (DOCX) [file pone.0282502.s014.docx]

**Session #5: Move those muscles**

**Learning objectives**

After session #5, participants will be able to:

- Describe their current level of physical activity.
- Name ways that they are already physically active.
- Explain the importance of a physical activity goal.
- Establish a physical activity goal.
- Develop personal plans for physical activity for the next week.

**Materials:**

- Participant handouts:
  - Overview
  - Physical activity goal
  - How active are you?
  - Benefits of physical activity
  - Getting started!
  - Wearing the right shoes
  - To do next week
- Food and Activity Tracker for session %
- Name tags
- White board and markers

**Overview**

Session 5 introduces the physical activity goal. This will be the first of several sessions on physical activity, so today’s content is an introduction. The goal is for each participant to feel comfortable and confident in choosing a physical activity they can do and maintain, gradually building up to 150 minutes per week.

This session assumes that most participants are not very physically active now. You can adapt the material for those who are already active.

There are three parts:

- Part 1: Weekly progress and review (10 minutes)
- Part 2: Physical activity goal (25 minutes)
  1. Discuss the many health benefits associated with being physically active.
  2. Discuss the need to gradually build up to the goal of 150 minutes a week. They may want to start with 60 minutes for the next week.
  3. Encourage participants to choose an activity that they like and that they can continue to do for the long term, even in the summer. Brisk walking is an ideal choice because walking can be done at different speeds and can be done indoors and outdoors.
- Part 3: Getting started with physical activity (15 minutes)
  1. Discuss safety.
  2. Discuss choosing a physical activity.
  3. Discuss proper footwear.
- Part 4: Wrap up and to-do list

**Key messages:**

- **The benefits of physical activity are numerous.**
- **For participants who are beginning a physical activity regimen, safety is the most important concern.**
- **Participants should build up to their physical activity goal slowly.**

**Classroom presentation**

*Part 1: Weekly progress and review (10 minutes)*

**Distribute:** Handouts for session 5 and Food and Activity Trackers for session 5.

Food and Activity Trackers from session 3 with notes and recommendations.

**Collect:** Session 4 Food and Activity Trackers.

**Discuss** participants’ successes and challenges in meeting their weight loss goals during the past week.

**Present:** Last week, we reviewed each food group in MyPlate and discussed how our current food choices compare with the MyPlate recommendations.

**Ask:** Can anyone remember examples of lean, healthy meats? How about healthy and not-so-healthy ways to eat fruits and vegetables?

**Open responses.**

**Present:** At the end of the last session I asked you to keep track of your weight and of what you eat and drink. I also asked you to compare what you eat and drink with the recommendations of the MyPlate model using the “Rate your plate” handout, just like we practiced in the last session.

**Ask:** How many of you were able to complete the “Rate your plate” handout? Would someone volunteer to share what they recorded.

**Discuss** using these questions how well the participants did:

- Did you make any changes during the week to match the MyPlate recommendations? If so, what did you do?
- What problems did you have? How were you able to solve them?
- Did you eat more mindfully?
- Did you try to eat only at regular meal times?

**Open responses.**

**Present:** This week we will

Discuss why physical activity is important, and why we must set goals for ourselves.

Look at our current level of physical activity.

Explore ways that we can be physically active.

Choose a personal physical activity goal.

Make a personal plan for the next week to reach our goal.

*Part 2: Physical activity goal (25 minutes)*

**Present:** So far in this program we have focused on losing weight mainly through healthy eating. We are keeping track of what we eat and limiting our fat and calorie intake. We will continue to do this for the rest of the program.

Today we are going to talk about adding another goal: a physical activity goal. We will talk about what we are already doing for physical activity and we will then talk about ways we might gradually increase our physical activity.

You can do it!

**Present:** Before we go any further, I want to assure you that you can succeed with this program.

It does not matter what your current activity level is. We will start wherever you are, and we will slowly and steadily increase your physical activity from there.

Just as we have been tracking our food, we will now begin to keep track of our physical activity. Keeping track of physical activity is just as important as keeping track of the food that we eat. Tracking anything helps to understand our behavior, what needs to change and helps us to reach our goal.

Your physical activity goal

**Ask:** Does anyone remember from week 1 what the program’s physical activity goal is?

**Open responses. If no one responds correctly: 150 minutes of physical activity per week.**

**Ask:** Why do we have this goal?

**Open responses.**

**Present:** Increasing physical activity is an excellent way to burn calories which helps us achieve and maintain our goal weight.

**Refer** to the “Physical activity goal” handout.

**Present:** The physical activity goal of this program is 150 minutes per week. This is the same as two and a half hours. The good news is that how you reach this goal is flexible. You could do 30 minutes all at one time each day for 5 days, or you can break down the 30 minutes into 2 15 minute sessions or even 3 10 minute sessions.

**Ask:** Does anyone know how many calories this will burn in one week?

**Open responses.**

**Present:** Approximately 700.

This may seem like a lot to you right now, but you can do it. What will help you do this is:

- Working up to this goal slowly. It may take you 4 weeks to get there. That’s OK.
- Pick activities you like. We want you to enjoy this, and we want it to be something convenient for you to do.
- We are asking for something like brisk walking, not anything extreme.
- This 150 minute goal is spread over a whole week.

Your current physical activity level

**Present:** Let’s start by looking at how active you are now.

**Refer** participants to the “How active are you?” handout.

**Ask** participants to take a few minutes to write some information about their current and past physical activity on the handout.

**Present:** Great, this exercise gives us an idea of where we are now with our level of physical activity, and it also suggests how we can find ways to be physically active starting today.

Also, I want to know about physical activities that you have done in the past, but no longer do. I want to know what they were, and why you stopped.

Finally, I want to know your likes and dislikes. What do you like about being active? What do you dislike about being active? What do you like and dislike about being inactive?

**Ask** for volunteers to share about their current level of activity, past experiences and why they stopped, and likes/dislikes for being active and inactive.

Benefits of physical activity

**Ask:** Why do you think that being physically active is so important?

**Open responses.**

**Refer** to the “Benefits of physical activity” handout.

**Present:**

- **It will help you feel and look better by**
  1. Improving your overall health.
  2. Helping you lose weight and keep it off.
  3. Improving your mood and how you feel about yourself.
  4. Working against depression and anxiety.
  5. Giving you more energy.
  6. Making it easier for you to do activities such as playing with children, going to the farm.
  7. Reducing stress.
  8. Helping you sleep better.
- **It will improve your physical fitness by**
  1. Improving muscle strength
  2. Reducing body fat.
  3. Making your joints more flexible.
  4. Reducing back pain.
- **It will improve your health by**
  1. Lowering your risk for heart problems and some kinds of cancer.
  2. Raising your good cholesterol.
  3. Lowering your blood pressure.
  4. Lowering your blood sugar.
  5. Lowering your cholesterol.

*Part 3: Getting started with physical activity (15 minutes)*

**Present:** We are going to make a physical activity plan for next week. We want you to be active every day, and to choose activities that you like.

**Refer** to the “Getting Started!” handout.

**Present:** This handout will help you get started with physical activity. It has some ideas to make this easier. Take a moment to look at it, and then when you have time later, fill it out.

Brisk walking is an easy way to become and stay physically active. The benefit is that it can be done almost anywhere, and all you need is a good pair of shoes.

**Ask:** What does “brisk” mean?

**Open responses.**

**Present:** When we say “brisk walking” we mean walking fast enough that you can’t easily sing a song, but that you CAN have a conversation.

If you haven’t been active, you will need to start slowly. We will build up to our goal over time. Next session we will talk more about how to do this.

**Ask:** What other activities might you like to do? Maybe activities that you enjoy but can only do part of the year?

**Write on the white board the activities.**

**Discuss:** Research here in Al Ain shows that some of the reasons people gave for not doing exercise were family obligations, cultural reasons and that it was boring. **Are there any choices here you could do together as members of a family?**

**Open responses.**

Choosing the right shoes

**Present:** A few minutes ago I mentioned that all you need for walking is a pair of good shoes. Let’s go over briefly what that means.

**Refer** participants to “Wearing the right shoes” handout.

**Present:** Our primary goal in starting exercise is to protect you from injury and keep you comfortable. Having a pair of good shoes is important for this. They need to fit well and prevent you from slipping.

**Present:** If you already have comfortable shoes that fit well, prevent slipping and support your feet, you do not need to buy new shoes. If you are looking at new shoes, this handout may be helpful. Let’s look at it for a moment together.

Going to the store:

Wear the kind of socks you will wear while walking. This helps you to make sure the shoes fit.

If possible, go to the store right after you have been walking. Sometimes your feet may be slightly larger when you have been active.

The shoes you choose should be comfortable right away. They should not need “breaking in”.

One thumb’s width should fit between your longest toe and the end of the shoe.

The heel should not pinch or slip while you walk.

Tell the salesperson that you want to use these shoes for brisk walking for exercise.

*Wrap up and to-do list (10 minutes)*

**Present:** Let’s make a plan for the next week. Remember, we are working towards eventually doing 150 minutes of exercise a week. But we are going to start slowly.

I ask you to set a goal of ***60 minutes*** for the next week.

I don’t want you to do this all in one day! I want you to do 10-20 minutes a day 3-6 days in the next week.

**Ask:** Do you think you can do this?

**Open responses.**

**Refer** to the “To do next week” hand out.

**Present:** Let’s look at this handout together. On the top there are the days of the week. On the side, I want you to write which activity you will do, and for how long.

Remember, I want you to choose activities that you like. It would be even better if you could do it with someone in your family or a friend.

This is the plan. Now when you do the activity, there is a place in your “Food and Activity Tracker” where you can write down what you did and for how many minutes. Just like when you are recording what you eat, it is better to write this down right after you do it. Only record the time you actually spent doing the physical activity. If you stop to rest or talk, don’t include that time.

Keep tracking what you eat like you have done before.

**Closing:**

**Summarize key points: Today:**

- **We discussed the many benefits to being physically active.**
- **You described your current level of activity.**
- **You chose a physical activity that works for you. I set a goal for you for the next week: 60 total minutes of activity.**
- **You planned for this week what activity you will do, on which day this coming week, and for how long.**

**Close:** I’m excited. We are making a big step forward toward improving your health this week. I know you will begin to feel and see some great benefits once you start. The important thing in the next few weeks is to begin slowly and increase gradually.

Next week we will build on what we did today. We will talk about finding time to be active, and how being active throughout the day can make a big difference to your health.

**Ask** if there are any questions or concerns.

**Address any questions or concerns.**

**After the session:**

Review each person’s Food and Activity Tracker from Session 4. Make notes and recommend changes.
