# Supplementary material for: Emirates Heart Health Project (EHHP): A protocol for a stepped-wedge family-cluster randomized-controlled trial of a health-coach guided diet and exercise intervention to reduce weight and cardiovascular risk in overweight and obese UAE nationals
Source: PLoS One. 2023 Apr 10;18(4):e0282502. doi: 10.1371/journal.pone.0282502 (PMC10085020; doi:10.1371/journal.pone.0282502)
Supplement: S15 Appendix — (DOC) [file pone.0282502.s015.doc]

**الجلسة رقم 5: حرك هذه العضلات**

**أهداف التعلم**

بعد الجلسة الخامسة ، سيتمكن المشاركون من:

- وصف مستوى نشاطهم البدني الحالي.
- تسمية الطرق التي هم فيها نشطين فعليا.
- شرح أهمية النشاط البدني.
- وضع هدف النشاط البدني.
- وضع خطط شخصية للنشاط البدني للأسبوع المقبل.

**المواد:**

- نشرات المشاركين:
- نظرة عامة
- هدف النشاط البدني
- كيف نشط أنت؟
- فوائد النشاط البدني
- ابدء!
- ارتداء الأحذية المناسبة
- قائمة المهام للأسبوع المقبل
- متتبع الطعام والنشاط
- بطاقات الأسماء
- لوحة بيضاء وقلم

**نظرة عامة**

الجلسة رقم 5 تقدم هدف النشاط البدني. ستكون هذه هي الأولى من عدة جلسات حول النشاط البدني ، لذلك محتوى اليوم هو مقدمة. الهدف هو أن يشعر كل مشارك بالراحة والثقة في اختيار النشاط البدني الذي يمكنه القيام به والحفاظ عليه ،و الزيادة بشكل تدريجي الى أن يصل إلى 150 دقيقة في الأسبوع.

تفترض هذه الجلسة أن معظم المشاركين ليسوا نشيطين بدنياً لغاية الآن. يمكنك تحضير بعض المواد لأولئك الذين يمارسون النشاط الرياضي.

هناك ثلاثة أجزاء:

الجزء 1: التقدم والمراجعة الأسبوعية (10 دقائق)

الجزء 2: هدف النشاط البدني (25 دقيقة)

1. مناقشة العديد من الفوائد الصحية المرتبطة بالنشاط البدني.

2. مناقشة الحاجة إلى الزيادة التدريجية إلى هدف 150 دقيقة في الأسبوع. قد يرغبون في البدء بـ 60 دقيقة للأسبوع المقبل.

3. شجع المشاركين على اختيار النشاط الذي يفضلونه والذي بإمكانهم الاستمرار في القيام به على المدى الطويل ، حتى في فصل الصيف. يعد المشي السريع خيارًا مثاليًا لأن المشي يمكن القيام به بسرعات مختلفة ويمكن القيام به في الداخل والخارج.

الجزء 3: البدء في ممارسة النشاط البدني (15 دقيقة)

1. ناقش السلامة.

2. مناقشة اختيار النشاط البدني.

3. مناقشة الأحذية المناسبة.

الجزء 4: الخاتمة وقائمة المهام

**الرسائل الرئيسية:**

- **فوائد النشاط البدني عديدة.**
- **بالنسبة للمشاركين الذين سوف يبدأون نظامًا للنشاط البدني ، فإن السلامة هي الأهم.**
- **يجب على المشاركين بناء هدف مدة نشاطهم البدني ببطء.**

**عرض الفصل الدراسي**

الجزء 1: التقدم والمراجعة الأسبوعية (10 دقائق)

**وزع :** النشرات للجلسة 5 ومتتبع الطعام والنشاط للجلسة 5.

متتبعو الطعام والنشاط من الجلسة 3 مع الملاحظات والتوصيات.

**اجمع**: متتبعي الطعام والنشاط للجلسة 4 .

**ناقش** نجاحات المشاركين وتحدياتهم في تحقيق أهداف إنقاص الوزن خلال الأسبوع الماضي.

**حاضر:** في الأسبوع الماضي ، استعرضنا كل مجموعة طعام في" طبقي" وناقشنا كيف نقارن خياراتنا الغذائية الحالية بتوصيات البرنامج.

**اسأل:** هل يمكن لأي شخص أن يتذكر أمثلة اللحوم الخالية من الدهن والصحية؟ ماذا عن الطرق الصحية وغير الصحية لتناول الفواكه والخضروات؟

**افتح المجال للرد.**

**حاضر:** في نهاية الجلسة الأخيرة ، طلبت منكم تتبع أوزانكم والطعام والشراب. كما طلبت منكم مقارنة ما تأكلونه وتشربونه مع توصيات نموذج "طبقي" باستخدام نشرة "قيم طبقك " ، تمامًا كما مارسناها في الجلسة الأخيرة.

**اسأل**: كم منكم تمكّن من إكمال نشرات "قيم صفحتك"؟ هل سيتطوع شخص ما لمشاركة ما سجله

**ناقش** باستخدام هذه الأسئلة مدى نجاح المشاركين:

- هل قمت بإجراء أي تغييرات خلال الأسبوع لتتناسب مع توصيات "طبقي" إذا كان الأمر كذلك، ماذا فعلت؟
- ما هي المشاكل التي واجهتك؟ كيف تمكنت من حلها؟
- هل أكلت أكثر بانتباه؟
- هل حاولت أن تأكل فقط في أوقات الوجبات العادية؟

**افتح المجال للرد.**

**حاضر**: هذا الأسبوع سوف

نناقش أهمية النشاط البدني ، ولماذا يجب أن نضع أهدافًا لأنفسنا.

ننظر إلى مستوى نشاطنا البدني الحالي.

نستكشف الطرق التي يمكن من خلالها ممارسة النشاط البدني.

نختار أهداف النشاط البدني الشخصية.

نضع خطة شخصية للأسبوع القادم للوصول إلى هدفنا.

الجزء 2: هدف النشاط البدني (25 دقيقة)

**حاضر:** حتى الآن في هذا البرنامج ركزنا على فقدان الوزن بشكل رئيسي من خلال الأكل الصحي. نحن نتتبع ما نأكله ونحد من تناول الدهون والسعرات الحرارية. سوف نستمر في القيام بذلك لبقية البرنامج.

اليوم سوف نتحدث عن إضافة هدف آخر: هدف النشاط البدني. سنتحدث عن ما نقوم به بالفعل لممارسة النشاط البدني وسنتحدث بعد ذلك عن الطرق لزيادة نشاطنا البدني تدريجياً.

يمكنك أن تفعل ذلك!

**حاضر**: قبل أن نذهب إلى أبعد من ذلك ، أريد أن أؤكد لكم أنه يمكنكم النجاح في هذا البرنامج.

لا يهم ما هو مستوى نشاطك الحالي. سنبدأ أينما كنت ، وسنزيد نشاطك البدني ببطء وثبات من هناك.

مثلما كنا نتتبع طعامنا ، سنبدأ الآن في تتبع نشاطنا البدني. إن تتبع النشاط البدني لا يقل أهمية عن تتبع الطعام الذي نتناوله. تتبعنا لأي شيء يساعدنا على فهم سلوكنا ، وفهم ما يحتاج إلى التغيير ويساعدنا على الوصول إلى هدفنا.

هدف النشاط البدني الخاص بك

**اسأل:** هل يتذكر أحد ما هو هدف النشاط البدني للبرنامج منذ الأسبوع الأول؟

**افتح المجال للرد**. **إذا لم يجب أحد بشكل صحيح: 150 دقيقة من النشاط البدني في الأسبوع.**

**اسأل:** لماذا لدينا هذا الهدف؟

**افتح المجال للرد**

**حاضر**: زيادة النشاط البدني هو وسيلة ممتازة لحرق السعرات الحرارية التي تساعدنا على الوصول و الحفاظ على الوزن المثالي .

**ارجع** الى نشرة "هدف النشاط البدني".

**حاضر**: هدف النشاط البدني لهذا البرنامج هو 150 دقيقة في الأسبوع. وهو يعد ساعتين ونصف. والخبر السار هو أن طريقة تحقيق هذا الهدف مرنة. يمكنك أن تفعل 30 دقيقة في وقت واحد كل يوم لمدة 5 أيام ، أو يمكنك تقسيم 30 دقيقة في جلستين كل واحدة 15 دقيقة أو حتى على 3 مرات كل مره 10 دقائق .

**اسأل:** هل يعرف أحد عدد السعرات الحرارية التي سيحرقها هذا في أسبوع واحد؟

**افتح المجال للرد**

**حاضر:** حوالي 700.

قد يبدو هذا كثيرًا بالنسبة لك في الوقت الحالي ، ولكن يمكنك القيام بذلك. ما سيساعدك على القيام بذلك هو:

- العمل حتى تصل لهذا الهدف ببطء. قد يستغرق الأمر 4 أسابيع للوصول إليه.
- اختيار الأنشطة التي تريدها. نريدك أن تستمتع بهذا ، ونريدها أن تكون مناسبة لك.
- نحن نطلب منكم أنشطة مثل المشي السريع ، وليس أنشطة شديدة الحدة.
- 150 دقيقة تكون على مدى أسبوع كامل.

مستوى نشاطك البدني الحالي

**حاضر:** لنبدأ بالنظر إلى مدى نشاطك الآن.

قم بإحالة المشاركين إلى منشور "ما مدى نشاطك؟"

اطلب من المشاركين أن يستغرقوا بضع الوقت لكتابة بعض المعلومات حول نشاطهم البدني الحالي والسابق على المنشور.

**حاضر:** رائع ، هذا التمرين يعطينا فكرة عن ما نحن عليه الآن، كما أنه يوحي أيضًا بكيفية إيجاد طرق لممارسة النشاط البدني بداية من اليوم.

كذلك ، أريد أن أعرف عن الأنشطة البدنية التي قمت بها في الماضي ، ولكن لم تعد تفعلها. أريد أن أعرف ماذا كانوا ولماذا توقفت.

أخيرًا ، أريد أن أعرف ما يعجبك وما لا يعجبك. ما الذي يعجبك في النشاط؟ ما الذي لا يعجبك في النشاط؟ ما الذي يعجبك وما لا يعجبك في أن تكون غير نشط؟

**اطلب** من المتطوعين المشاركة حول مستوى نشاطهم الحالي وتجاربهم السابقة ولماذا توقفوا ، وما يحب / وما لا يحب عن كونه نشطًا وغير نشط.

فوائد النشاط البدني

**اسأل:** لماذا تعتقد أن النشاط البدني مهم للغاية؟

**افتح المجال للرد**

راجع "فوائد النشاط البدني".

**حاضر:**

**سوف يساعدك على أن تبدوا و تشعر بشكل أفضل عن طريق**

1. تحسين صحتك العامة.
2. مساعدتك على فقدان الوزن والحفاظ عليه.
3. تحسين حالتك المزاجية شعورك حيال نفسك.
4. العمل ضد الاكتئاب والقلق.
5. يعطيك المزيد من الطاقة.
6. يجعل من السهل عليك القيام بأنشطة مثل اللعب مع الأطفال ، والذهاب إلى المزرعة.
7. الحد من التوتر.
8. مساعدتك على النوم بشكل أفضل.

**وسوف يحسن لياقتك البدنية عن طريق**

1. تحسين قوة العضلات
2. تقليل الدهون في الجسم.
3. جعل المفاصل أكثر مرونة.
4. الحد من آلام الظهر.

**وسوف يحسن صحتك عن طريق**

1. خفض خطر الإصابة بأمراض القلب وبعض أنواع السرطان.
2. رفع مستوى الكولسترول الجيد.
3. خفض ضغط الدم.
4. خفض نسبة السكر في الدم.
5. خفض الكولسترول في الدم

الجزء 3: البدء في ممارسة النشاط البدني (15 دقيقة)

**حاضر:** سنقوم بوضع خطة نشاط بدني للأسبوع المقبل. نريد منك أن تكون نشطًا كل يوم ، وأن تختار الأنشطة التي تريدها.

**ارجع** الى نشرة "البدء في العمل!"

**حاضر:** سيساعدك هذا المنشور في البدء في ممارسة النشاط البدني. يوجد به بعض الأفكار لجعل البداية أسهل. سوف نتوقف لحظة لمراجعته ، ثم عندما يكون لديك وقت لاحق ، املأه.

المشي السريع هو وسيلة سهلة لنصبح ونبقى نشطين بدنيا. الفائدة هي أنه يمكن القيام به في أي مكان تقريبًا ، وكل ما تحتاج إليه هو حذاء جيد.

**اسأل:** ماذا تعني كلمة "سريع"؟

**افتح المجال للرد**

**حاضر:** عندما نقول "المشي السريع" نعني المشي بسرعة كافية بحيث لا يمكنك بسهولة غناء أغنية ، ولكن يمكنك إجراء محادثة.

إذا لم تكن نشط ، فستحتاج إلى البدء ببطء. سنصل الى هدفنا مع مرور الوقت. الجلسة التالية سنتحدث أكثر عن كيفية القيام بذلك.

**اسأل:** ما هي الأنشطة الأخرى التي قد ترغب في القيام بها؟ ربما الأنشطة التي تستمتع ولكن يمكن أن تقوم بها في جزء معين من السنة؟

**اكتب على السبورة البيضاء الأنشطة.**

ناقش: أظهرت الأبحاث هنا في العين أن بعض الأسباب التي أعطاها الناس لعدم ممارسة التمارين كانت التزامات عائلية ، وأسباب ثقافية ولأنها مملة. هل هناك أي خيارات يمكنك القيام بها معًا كأفراد في عائلة؟

**افتح المجال للرد**

اختيار الأحذية المناسبة

**حاضر:** قبل بضع دقائق ذكرت أن كل ما تحتاجه للمشي هو زوج من الأحذية الجيدة. دعنا نناقش ماذا يعني ذلك.

قم **بإحالة** المشاركين إلى نشرة "ارتداء الأحذية المناسبة".

**حاضر:** هدفنا الأساسي في بدء التمرين هو حمايتك من الإصابة والحفاظ على راحتك. وجود زوج من الأحذية الجيدة أمر مهم لهذا الغرض. يجب أن يكون مناسب و يمنعك من الانزلاق.

**حاضر:** إذا كان لديك بالفعل أحذية مريحة تناسبك بشكل جيد ، وتمنع الانزلاق وتدعم قدميك ، فلن تحتاج إلى شراء أحذية جديدة. إذا كنت تبحث عن حذاء جديد ، فقد يكون هذا المنشور مفيدًا. لننظر إليه للحظة معًا.

الذهاب إلى المتجر:

ارتدي نوع الجوارب التي سترتديها أثناء المشي. هذا يساعدك على التأكد من أن الأحذية مناسبة.

إذا أمكن ، انتقل إلى المتجر مباشرة بعد المشي. في بعض الأحيان قد تكون قدميك أكبر قليلاً عندما تكون نشطًا.

يجب أن تكون الأحذية التي تختارها مريحة على الفور.

يجب أن يتناسب عرض الإبهام بين أطول إصبع قدم ونهاية الحذاء.

يجب ألا ينزلق الكعب أثناء المشي.

أخبر مندوب المبيعات أنك تريد استخدام هذه الأحذية للمشي السريع للتمرين.

الخاتمة وقائمة المهام (10 دقائق)

**حاضر:** دعونا نضع خطة للأسبوع القادم. تذكروا أننا نعمل من أجل أن نصل لممارسة 150 دقيقة في الأسبوع. لكننا سنبدأ ببطء.

أطلب منكم تحديد هدف 60 دقيقة للأسبوع المقبل.

لا أريدكم أن تفعلوا كل هذا في يوم واحد! أريدكم أن تفعلوا 10-20 دقيقة في اليوم 3-6 أيام في الأسبوع المقبل.

**اسأل**: هل تعتقدون أنه بامكانكم القيام بذلك؟

**افتح المجال للرد**

ارجع إلى منشور "قائمة المهام للأسبوع المقبل".

**حاضر:** دعونا ننظر إلى هذا المنشور معًا. في الأعلى يوجد أيام الأسبوع. على الجانب ، أريدك أن تكتب النشاط الذي ستقوم به ومدته.

تذكر ، أريدك أن تختار الأنشطة التي تريدها. سيكون من الأفضل لو أمكنك القيام بذلك مع شخص ما في عائلتك أو صديق.

هذه هي الخطة. الآن عند القيام بالنشاط ، يوجد مكان في "متتبع الطعام والنشاط" حيث يمكنك كتابة ما قمت به وعدد الدقائق. تمامًا مثلما تقوم بتسجيل ما تأكله ، من الأفضل أن تكتب مدة النشاط مباشرة بعد القيام به. سجل فقط الوقت الذي قضيته بالفعل في ممارسة النشاط البدني. إذا توقفت للراحة أو التحدث ، فلا تشمل ذلك الوقت.

استمر في تتبع ما تأكله كما فعلت من قبل.

**النهاية :**

**تلخيص النقاط الرئيسية: اليوم:**

- **ناقشنا العديد من فوائد النشاط البدني.**
- **لقد وصفت مستوى نشاطك الحالي.**
- **لقد اخترت نشاطًا بدنيًا يناسبك. لقد حددت لك هدفًا للأسبوع القادم: 60 دقيقة من النشاط.**
- **لقد خططت لهذا الأسبوع ما هو النشاط الذي ستقوم به ، وفي أي يوم من هذا الأسبوع القادم ، وكم الدة.**

**اختتم:** أنا متحمس. نحن نخطو خطوة كبيرة للأمام نحو تحسين صحتك هذا الأسبوع. أعلم أنك ستبدأ في الشعور ببعض الفوائد الرائعة بمجرد أن تبدأ. الشيء المهم في الأسابيع القليلة المقبلة هو البدء ببطء والزيادة التدريجية.

الأسبوع القادم سوف نبني على ما فعلناه اليوم. سنتحدث عن إيجاد وقت لنكون نشيطين ، وكيف نشاطك طوال اليوم يمكن أن يحدث فرقًا كبيرًا في صحتك.

**اسأل** إذا كان هناك أي أسئلة أو مخاوف.

معالجة أي أسئلة أو مخاوف.

بعد الجلسة:

راجع "متتبع الطعام والنشاط" الخاص بكل شخص من الجلسة 4. قم بتدوين الملاحظات والتوصيات بالتغييرات.
