# Supplementary material for: Emirates Heart Health Project (EHHP): A protocol for a stepped-wedge family-cluster randomized-controlled trial of a health-coach guided diet and exercise intervention to reduce weight and cardiovascular risk in overweight and obese UAE nationals
Source: PLoS One. 2023 Apr 10;18(4):e0282502. doi: 10.1371/journal.pone.0282502 (PMC10085020; doi:10.1371/journal.pone.0282502)
Supplement: S16 Appendix — (DOCX) [file pone.0282502.s016.docx]

**Session 6: Being active- a way of life**

**Learning objectives**

By the end of this session, participants will be able to:

- Track their daily physical activity.
- Describe two ways of finding time to be active.
- Define “lifestyle activity”.
- Describe how to prevent injury.
- Develop an activity plan for the coming week.

**Materials**

- **Participant handouts**
  - How am I doing? Physical activity.
  - You can find the time!
  - Lifestyle activity
  - Keep it safe
  - Safe and easy stretches
  - Treating an injury
  - When to stop exercising
  - To do for next week
- Food and activity trackers for session 6
- Name tags
- Whiteboard and markers

**Overview**

Session 6 is the second session on physical activity. During the last session, Session 5, we set physical activity goals of 60 minutes. Using their successes and challenges, discuss this week what makes increasing physical activity difficult for participants, and how to overcome those challenges. Lifestyle activity (choosing to be active throughout the day) will be discussed as a part of the daily physical activity.

Session 6 is divided into four parts:

*Part 1: Weekly progress and review (10 minutes)*

*Part 2: Overcoming barriers (20 minutes):* This will be a group discussion on generating ideas for finding solutions to challenges, especially finding time for physical activity.

*Part 3: Lifestyle activity (20 minutes)*: Having a physically active life is not just about 20-30 minutes a day of exercise. It also involves making active choices such as parking the car further away or using the stairs instead of the elevator.

*Part 4: Wrap up and to-do list (10 minutes)*

**Key messages**

- **You must make the choice to include physical activity in your day.**
- **Train yourself to look for blocks of time when you can be physically active.**
- **Using small blocks of time to be physically active makes reaching your physical activity goal easier.**
- **When you start a physical activity program, you must pay attention to your physical condition, prepare your body properly for increased activity, and be aware of whether your pace is too slow or too fast.**
- **Keeping safe is vital. Know when you need to stop.**

*Part 1: Weekly progress and review (10 minutes)*

**Distribute** Session 6 handouts, Session 6 Food and Activity Trackers, Session 4 “Food and Activity Trackers” with notes.

**Collect** Session 5 Food and Activity Trackers.

**Review** ground rules, if needed.

**Present:** Last week, we began discussing physical activity and set a goal for 60 minutes for this week.

**Ask:** How much physical activity did you do last week?

**Open responses.**

**Ask for volunteers** to share the type and amount of physical activity they did and any related challenges.

**Ask:** Did anyone run into any problems? Weather related? Time?

**Briefly discuss.**

**Present:** This week we will:

- Begin recording your daily physical activity.
- Discuss how lack of time can be a barrier to physical activity.
- Look at different ways to find time to be active and how to incorporate that into our daily routine.
- Look at ways to prevent injury.
- Develop an activity plan for the coming week.

**Present:** Starting with this session, we will record your physical activity and use it to see your progress over time.

**Open Fitbit and smartphone app session.**

*Part 2: Overcoming barriers (20 minutes)*

Barriers to being active

**Present:** Now that we have started setting weekly physical activity goals, it’s important to address any challenges we might be having in following our activity plans. In our weekly review, we started to talk about challenges we had the past week.

**Ask:** Does anyone have any ideas about how to deal with the problems we had last week? Any techniques or solutions that worked well for you?

**Use whiteboard to take notes.**

Note to coach: The most common problems (and solutions) will be:

Time: we will discuss this in the next section.

Children/childcare: ask a family member to help, share/take turns. Use stroller to include child in walking times.

Hot weather: walk indoors. Walk late in the evening.

Finding time to be active

**Present:** For many of us, the biggest problem we have in trying to be active is lack of time. Let’s talk about some possible ideas to overcome that problem.

First, plan to be active. Schedule it into your day. You can:

Set aside a certain block of time every day for planned activity. For example, walk after dinner every night.

Use your calendar on your phone to remind you.

Using your routine can help you.

Your goal for this week is to be active for a total of 90 minutes this week.

**Ask:** When can you set aside 20-30 minutes to do an activity you like? If that’s not possible, when can you schedule 10 minutes twice a day?

Are you a morning person? Would you enjoy walking during lunch? How about after dinner?

**Open responses.**

Using small blocks of time

**Present:** Some of us are so busy we cannot free up 20-30 minutes all in one block of time. Or our schedules may be unpredictable, meaning all of a sudden we have to stop what we are doing to take care of something else.

**Ask:** Does anyone have any ideas on ways to fit in 20-30 minutes of activity a day?

**Open responses.**

**Refer** to the “You can find the time!” handout.

**Present:** One idea is to find several smaller blocks of time. Instead of 30 minutes at one time, maybe you could do 10 minutes in the morning and 20 minutes in the evening. By the end of the day you’ve done 30 minutes, and if you do that 5 days a week, you will have done the recommended amount of exercise.

Seize the moment!

**Present:** Of course, not everyone can schedule these blocks of time, so you could practice watching for opportunities.

If you arrive early to an appointment, walk around the building for a few minutes, or take the stairs.

**Ask:** Can you think of any times during the day when you might have 10 or 15 free minutes?

**Open responses.**

**Present:** Being active does not have to mean trips to the gym or long sessions on the treadmill. You can be active in many places. It is important that you find the time- in the amounts most comfortable to you- to be active throughout the day. You will be surprised at how easy it can be once you start to look for those times.

*Part 3: Lifestyle Activity (20 minutes)*

**Present:** So far, we have talked mostly about the kind of physical activity you will be recording in your “Food and Activity Trackers”, whether you do it all at once or in parts throughout the day.

**Refer** to the “Lifestyle activity” handout.

Another important kind of activity is called “lifestyle activity.” This is making active choices instead of inactive choices. This handout shows some examples of active and inactive choices. An example is to park your car a little farther away and walking instead of as close as possible.

These choices may take only an extra minute or two, but over time these minutes will add up and make a difference. They are generally too brief to count and record, but they will make a significant difference in your overall activity level.

**Ask:** Can anyone think of other ways to be active rather than inactive?

**Open responses.**

**Present:** Many of our parents, and especially our grandparents, did not have a choice about being active during the day. They were active because:

- They had to walk places.
- They had to do laundry and dishes by hand.
- They had to obtain and prepare food.
- They had to take care of animals and farms.

In contrast, most of us have so many conveniences that our lives will be inactive unless we **choose** to be active.

**Ask:** What are some active choices you could make during the day? What are some inactive choices you could limit?

**Write the responses on the whiteboard.**

**After they are finished, write:**

Walking down the hall to talk to your family instead of calling or WhatsApp.

Stand while using the phone instead of sitting.

**Present:** Many people say they have no time in their day to take a walk, but they are on their mobile or watch TV for several hours every evening. Try turning some of that time into walking time.

At first, you may think of walking as work, but when you get used to it, you will discover that walking is a great way to relax, and that you may feel much more rested and refreshed than if you had spent that time on your electronics.

Getting started

**Refer** to the “Keep it safe” handout.

**Present:** As described in this handout, there are a few easy practices that can help you stay safe while being active.

- Build up to brisk activity slowly.
- Start each session slowly to warm up.
- End each session slowly to cool down.
- Drink plenty of water before, during and after activity.
- Wear comfortable socks and keep your feet dry.

When to stop exercising

**Present:** Although being physically active is usually safe, sometimes you should stop exercising. I cannot give you medical advice, so if you have questions about whether you should continue your physical activity plan, please see your doctor for advice.

If you have chest pain or discomfort, severe nausea, shortness of breath, severe sweating, or a lightheaded feeling, you should stop exercising.

If these feelings do not go away soon after stopping your exercise, you should to to a hospital emergency department.

*Part 4: Wrap up and to-do list (10 minutes)*

**Ask** whether anyone has any questions about the material from this session.

**Refer** to the “To do next week” handout.

**Present:** Now let’s make our activity plan for next week. The goal is to do a little more than last week, for a weekly total of 90 minutes of activity. On the handout, write the activities you plan to do each day of the week with the number of minutes for each activity.

Note: If the participant did more or less than 60 minutes last week, use your judgment about their activity goal for the week. Try to add between 15-30 minutes.

**Present:** As usual, I am going to ask you to track your weight, what you eat and the number of minutes of physical activity in your “Food and Activity Tracker”.

Closing

**Summarize these key points.**

- **Make the *choice* to include physical activity in your day.**
- **Be active for small blocks of time throughout the day, if that makes being active easier for you.**
- **Be creative: exchange 10 minutes of time on your electronics for a walk around the yard.**
- **Start slowly, warm up and cool down, and wear comfortable shoes and clothes.**
- **Be safe, and recognize when you need to stop.**

**Close:** If you make a conscious choice to add physical activity to your day, you may be surprised at how easy it is to do so. Remember to be safe. Build up slowly and go at a pace that works for you.

Our next session is called “Tip the calorie balance.” The calorie balance is about the number of calories in against the number of calories out. We will talk about how healthy eating and physical activity can work together to really tip that scale toward weight loss.

**Ask** the participants if there are any questions or concerns.

**Address any questions or concerns.**

**After the session:**

Make notes on each person’s “Food and Activity Tracker” from session 5.
