# Supplementary material for: Emirates Heart Health Project (EHHP): A protocol for a stepped-wedge family-cluster randomized-controlled trial of a health-coach guided diet and exercise intervention to reduce weight and cardiovascular risk in overweight and obese UAE nationals
Source: PLoS One. 2023 Apr 10;18(4):e0282502. doi: 10.1371/journal.pone.0282502 (PMC10085020; doi:10.1371/journal.pone.0282502)
Supplement: S17 Appendix — (DOCX) [file pone.0282502.s017.docx]

**الجلسة 6: أن تكن نشيطًا - نمط للحياة**

**الأهداف**

بحلول نهاية هذه الجلسة ، سيكون المشاركون قادرين على:

- تتبع نشاطهم البدني اليومي.
- وصف طريقتين لإيجاد الوقت ليكونوا نشطًين
- تحديد "نشاط نمط الحياة".
- وصف كيفية الوقاية من الإصابة.
- وضع خطة النشاط للأسبوع المقبل.

**المواد**

نشرات المشاركين

كيف افعل النشاط البدني.

!يمكنك العثور على الوقت

نشاط نمط الحياة

احتفظ بها آمنة

تمتد آمنة وسهلة

علاج الاصابة

متى تتوقف عن ممارسة الرياضة

القيام به للأسبوع المقبل

متعقبو الطعام والنشاط للجلسة 6

علامات الأسماء

السبورة والعلامات

نظرة عامة

الجلسة 6 هي الجلسة الثانية عن النشاط البدني. خلال الجلسة الأخيرة ، الجلسة 5 ، وضعنا أهداف النشاط البدني لمدة 60 دقيقة. باستخدام نجاحاتهم وتحدياتهم ، ناقش هذا الأسبوع ما الذي يجعل زيادة النشاط البدني صعبة للمشاركين ، وكيفية التغلب على هذه التحديات. ستتم مناقشة نشاط نمط الحياة (اختيار أن يكون نشطًا طوال اليوم) كجزء من النشاط البدني اليومي.

تنقسم الجلسة 6 إلى أربعة أجزاء:

الجزء 1: التقدم والمراجعة الأسبوعية (10 دقائق)

الجزء 2: التغلب على العوائق (20 دقيقة): ستكون هذه مناقشة جماعية حول توليد الأفكار لإيجاد حلول للتحديات ، لا سيما إيجاد وقت للنشاط البدني.

الجزء 3: نشاط نمط الحياة (20 دقيقة): إن ممارسة حياة جسدية نشطة ليس فقط حوالي 20-30 دقيقة في اليوم من التمارين. كما يتضمن أيضًا اتخاذ خيارات نشطة مثل إيقاف السيارة بعيدًا أو استخدام السلالم بدلاً من المصعد.

الجزء 4: اختتام وقائمة المهام (10 دقائق)

الرسائل الرئيسية

يجب عليك اختيار تضمين النشاط البدني في يومك.

تدريب نفسك للبحث عن كتل من الوقت عندما تكون نشطة بدنيا.

إن استخدام كتل صغيرة من الوقت للنشاط البدني يجعل الوصول إلى هدف نشاطك البدني أسهل.

عندما تبدأ برنامجًا للنشاط البدني ، يجب الانتباه إلى حالتك البدنية ، وإعداد جسمك بشكل صحيح لزيادة النشاط ، وتكون على دراية بما إذا كانت وتيرتك بطيئة أو سريعة للغاية.

الحفاظ على السلامة أمر حيوي. تعرف عندما تحتاج إلى التوقف.

الجزء 1: التقدم والمراجعة الأسبوعية (10 دقائق)

قم بتوزيع النشرات الخاصة بالجلسة 6 ، ومتبعي الطعام والنشاط في الجلسة 6 ، الجلسة 4 "متتبعي الغذاء والنشاط" مع الملاحظات.

جمع الجلسة 5 الغذاء والنشاط بتتبع.

مراجعة القواعد الأساسية ، إذا لزم الأمر.

الحاضر: في الأسبوع الماضي ، بدأنا مناقشة النشاط البدني ووضع هدف لمدة 60 دقيقة لهذا الأسبوع.

اسأل: ما مقدار النشاط البدني الذي قمت به الأسبوع الماضي؟

ردود مفتوحة.

اطلب من المتطوعين مشاركة نوع ومقدار النشاط البدني الذي قاموا به وأي تحديات ذات صلة.

اسأل: هل واجه أي شخص أي مشاكل؟ الطقس ذات الصلة؟ زمن؟

**مناقشة لفترة وجيزة.**

**الحاضر: هذا الأسبوع سوف:**

**ابدأ في تسجيل نشاطك البدني اليومي.**

**ناقش كيف يمكن أن يكون ضيق الوقت عائقًا أمام النشاط البدني.**

**انظر إلى طرق مختلفة للعثور على الوقت لتكون نشطًا وكيفية دمج ذلك في روتيننا اليومي.**

**انظر إلى طرق الوقاية من الإصابة.**

**وضع خطة نشاط للأسبوع المقبل.**

**حاضر: بدءًا من هذه الجلسة ، سنسجل نشاطك البدني ونستخدمه لرؤية تقدمك بمرور الوقت.**

**فتح جلسة تطبيق Fitbit والهاتف الذكي.**

الجزء 2: التغلب على الحواجز (20 دقيقة)

الحواجز التي تحول دون أن تكون نشطة

الحاضر: الآن وقد بدأنا في تحديد أهداف النشاط البدني الأسبوعية ، من المهم مواجهة أي تحديات قد نواجهها في متابعة خطط أنشطتنا. في مراجعتنا الأسبوعية ، بدأنا نتحدث عن التحديات التي واجهناها في الأسبوع الماضي.

اسأل: هل لدى أي شخص أي أفكار حول كيفية التعامل مع المشكلات التي واجهناها الأسبوع الماضي؟ أي التقنيات أو الحلول التي عملت بشكل جيد بالنسبة لك؟

استخدم السبورة لتدوين الملاحظات.

ملاحظة للمدرب: المشاكل الأكثر شيوعًا (والحلول) ستكون:

الوقت: سنناقش هذا في القسم التالي.

الأطفال / رعاية الطفل: اطلب من أحد أفراد الأسرة المساعدة ، وتبادل / يتناوب. استخدم عربة الأطفال لتشمل الطفل في أوقات المشي.

الطقس الحار: المشي في الداخل. المشي في وقت متأخر من المساء.

العثور على الوقت لتكون نشطة

الحاضر: بالنسبة للكثيرين منا ، فإن أكبر مشكلة لدينا في محاولة أن تكون نشطا هي ضيق الوقت. دعونا نتحدث عن بعض الأفكار الممكنة للتغلب على هذه المشكلة.

أولا ، خطة لتكون نشطة. جدولة ذلك في يومك. تستطيع:

خصص جزءًا معينًا من الوقت كل يوم للنشاط المخطط. على سبيل المثال ، المشي بعد العشاء كل ليلة.

استخدم التقويم الخاص بك على هاتفك لتذكيرك.

باستخدام الروتين الخاص بك يمكن أن تساعدك.

هدفك لهذا الأسبوع هو أن تكون نشطًا لمدة 90 دقيقة هذا الأسبوع.

اسأل: متى يمكنك تخصيص 20-30 دقيقة للقيام بنشاط تريد؟ إذا لم يكن ذلك ممكنًا ، فمتى يمكنك جدولة 10 دقائق مرتين في اليوم؟

هل أنت من النوع الذي يستيقظ باكرا؟ هل تستمتع بالمشي أثناء الغداء؟ ماذا عن بعد العشاء؟

ردود مفتوحة.

باستخدام كتل صغيرة من الوقت

حاضر: البعض منا مشغول للغاية ولا يمكننا تحرير ما بين 20 إلى 30 دقيقة في كتلة واحدة من الوقت. أو قد لا يمكن التنبؤ بجداولنا ، وهذا يعني فجأة أننا يجب أن نتوقف عن ما نقوم به لرعاية شيء آخر.

اسأل: هل لدى أي شخص أي أفكار حول طرق احتواء النشاط خلال 20-30 دقيقة من النشاط يوميًا؟

ردود مفتوحة.

راجع "يمكنك العثور على الوقت!"

الحاضر: فكرة واحدة هي العثور على عدة كتل أصغر من الوقت. بدلاً من 30 دقيقة في وقت واحد ، ربما يمكنك عمل 10 دقائق في الصباح و 20 دقيقة في المساء. بحلول نهاية اليوم ،

فعلت 30 دقيقة ، وإذا كنت تفعل ذلك 5 أيام في الأسبوع ، كنت قد فعلت المبلغ الموصى به من التمارين.

تمسك باللحظة!

حاضر: بالطبع ، لا يمكن لأي شخص تحديد هذه الكتل الزمنية ، لذلك يمكنك التدرب على البحث عن الفرص.

إذا وصلت مبكرا إلى موعد ، تجول في المبنى لبضع دقائق ، أو خذ الدرج.

اسأل: هل يمكنك التفكير في أي أوقات خلال اليوم عندما يكون لديك 10 أو 15 دقيقة مجانية؟

ردود مفتوحة.

حاضر: ممارسة النشاط لا يعني بالضرورة القيام برحلات إلى الجيم أو الجلسات الطويلة على جهاز الجري. يمكنك أن تكون نشطة في العديد من الأماكن. من المهم أن تجد الوقت - بالكميات الأكثر راحة لك - لتكون نشطًا طوال اليوم. سوف تفاجأ بمدى السهولة التي يمكنك بها بمجرد أن تبدأ في البحث عن تلك الأوقات.

الجزء 3: نشاط نمط الحياة (20 دقيقة)

الحاضر: حتى الآن ، تحدثنا في الغالب عن نوع النشاط البدني الذي ستقوم بتسجيله في "متتبعي الطعام والنشاط" ، سواء كنت تفعل كل ذلك مرة واحدة أو في أجزاء طوال اليوم.

راجع نشرة "نشاط لايف ستايل".

هناك نوع مهم آخر من الأنشطة يسمى "نشاط نمط الحياة". هذا هو اتخاذ خيارات نشطة بدلاً من الخيارات غير النشطة. يعرض هذا البيان بعض الأمثلة على الخيارات النشطة وغير النشطة. مثال على ذلك هو ركن سيارتك بعيدًا قليلاً والمشي بدلاً من أقرب وقت ممكن.

قد تستغرق هذه الاختيارات دقيقة أو دقيقتين إضافيتين ، ولكن بمرور الوقت ، ستزيد هذه الدقائق وتحدث فرقًا. عادةً ما تكون قصيرة جدًا بحيث لا يمكن حسابها وتسجيلها ، ولكنها ستحدث اختلافًا كبيرًا في مستوى نشاطك الكلي.

اسأل: هل يمكن لأي شخص أن يفكر في طرق أخرى لتكون نشطًا وليس نشطًا؟

ردود مفتوحة.

حاضر: العديد من آبائنا ، ولا سيما أجدادنا ، لم يكن لديهم خيار حول النشاط خلال اليوم. كانوا نشطين بسبب:

كان عليهم المشي الأماكن.

كان عليهم القيام بالغسيل والأطباق باليد.

كان عليهم الحصول على الطعام وإعداده.

كان عليهم رعاية الحيوانات والمزارع.

على النقيض من ذلك ، لدى معظمنا الكثير من وسائل الراحة بحيث تكون حياتنا غير نشطة ما لم نختار أن نكون نشطين.

اسأل: ما هي بعض الخيارات النشطة التي يمكنك القيام بها خلال اليوم؟ ما هي بعض الخيارات غير النشطة التي يمكنك الحد منها؟

اكتب الردود على السبورة.

بعد الانتهاء ، اكتب:

المشي في القاعة للتحدث مع عائلتك بدلاً من الاتصال أو WhatsApp.

قف أثناء استخدام الهاتف بدلاً من الجلوس.

الحاضر: يقول الكثير من الناس أنه ليس لديهم وقت في يومهم للتمشية ، لكنهم على هواتفهم المحمولة أو يشاهدون التلفزيون لعدة ساعات كل مساء. حاول تحويل بعض الوقت إلى وقت المشي.

في البداية ، قد تفكر في المشي كعمل ، ولكن عندما تعتاد على ذلك ، سوف تكتشف أن المشي وسيلة رائعة للاسترخاء ، وأنك قد تشعر بالراحة والانتعاش أكثر مما لو كنت قد قضيت هذا الوقت في حياتك الإلكترونيات.

ابدء

ارجع إلى النشرة "احتفظ بها آمنة".

الحاضر: كما هو موضح في هذا البيان ، هناك بعض الممارسات السهلة التي يمكن أن تساعدك على البقاء آمنًا أثناء نشاطك.

بناء ما يصل إلى النشاط السريع ببطء.

ابدأ كل جلسة ببطء في عملية الاحماء.

إنهاء كل جلسة ببطء لتهدئة.

شرب الكثير من الماء قبل وأثناء وبعد النشاط.

ارتداء الجوارب المريحة والحفاظ على قدميك جافة.

متى تتوقف عن ممارسة الرياضة

حاضر: على الرغم من أن النشاط البدني عادة ما يكون آمنًا ، إلا أنه يجب عليك في بعض الأحيان التوقف عن ممارسة الرياضة. لا يمكنني تقديم المشورة الطبية لك ، لذلك إذا كانت لديك أسئلة حول ما إذا كان يجب عليك متابعة خطة نشاطك البدني ، فيرجى مراجعة طبيبك للحصول على المشورة.

إذا كنت تعاني من ألم في الصدر أو عدم راحة ، أو غثيان شديد ، أو ضيق في التنفس ، أو تعرق شديد ، أو شعور بدوار ، فيجب عليك التوقف عن ممارسة الرياضة.

إذا لم تختف هذه المشاعر بعد فترة قصيرة من إيقاف التمرين ، فيجب عليك الذهاب إلى قسم الطوارئ في المستشفى.

الجزء 4: اختتام وقائمة المهام (10 دقائق)

اسأل ما إذا كان لدى أي شخص أي أسئلة حول المواد من هذه الجلسة.

ارﺟﻊ إﻟﻰ اﻟﻨﺸﺮة "اﻟﻘﻴﺎم ﺑﺎﻷداء ﻓﻲ اﻷﺳﺒﻮع اﻟﻤﻘﺒﻞ".

تقديم: الآن ، فلنضع خطة نشاطنا للأسبوع المقبل. الهدف هو القيام بما هو أكثر بقليل من الأسبوع الماضي ، ليصبح المجموع الأسبوعي 90 دقيقة من النشاط. في البيان ، اكتب الأنشطة التي تخطط للقيام بها كل يوم من أيام الأسبوع بعدد الدقائق لكل نشاط.

ملاحظة: إذا كان المشارك قد فعل أكثر من 60 دقيقة أو أقل من الأسبوع الماضي ، فاستخدم حكمك بشأن هدف نشاطه للأسبوع. حاول أن تضيف ما بين 15 إلى 30 دقيقة.

الحاضر: كالعادة ، سأطلب منك تتبع وزنك وما تأكله وعدد دقائق النشاط البدني في "متتبع الطعام والنشاط".

إغلاق

تلخيص هذه النقاط الرئيسية.

قم باختيار تضمين النشاط البدني في يومك.

كن نشيطًا لكتل ​​صغيرة من الوقت على مدار اليوم ، إذا كان ذلك يجعلك أكثر نشاطًا لك.

كن مبدعًا: استبدل 10 دقائق من الوقت على إلكترونياتك للتجول في الفناء.

ابدأ ببطء واحم نفسك وتهدئة ، وارتداء أحذية وملابس مريحة.

كن آمناً ، وتعرّف عندما تحتاج إلى التوقف.
