# Supplementary material for: Emirates Heart Health Project (EHHP): A protocol for a stepped-wedge family-cluster randomized-controlled trial of a health-coach guided diet and exercise intervention to reduce weight and cardiovascular risk in overweight and obese UAE nationals
Source: PLoS One. 2023 Apr 10;18(4):e0282502. doi: 10.1371/journal.pone.0282502 (PMC10085020; doi:10.1371/journal.pone.0282502)
Supplement: S18 Appendix — (DOCX) [file pone.0282502.s018.docx]

**Session 7: Tip the calorie balance**

**Learning objectives**

At the close of this session, the participants will be able to:

- Define “calorie balance”.
- Explain how healthy eating and being active are related in terms of calorie balance.
- Describe how calorie balance influences weight loss.
- Describe his or her progress as it relates to calorie balance.
- Develop an activity plan for the coming week.

**Materials**

- Participant handouts for Session 7
  - Overview
  - Understanding the calorie balance
  - Balancing food and activity
  - Tipping the balance
  - Review of your calorie balance
  - A few copies of Session 2’s “Tracking fat and calories”
  - To do next week
- Food and Activity Trackers for Session 7
- Name tags, if needed
- Whiteboard and markers

**Overview**

Session 7 pulls together the previous six sessions which were about healthy eating and physical activity. During this session we show how these two topics are related and how they work together to produce weight loss.

There are 4 parts:

Part 1: Weekly progress and review (10 minutes)

Part 2: What is “calorie balance”? (10 minutes)

The “calorie balance” is the relationship between calories in (eating) and calories out (activity). In this section we review which food groups are relatively higher in calories, and how we use those calories.

Part 3: The “calorie balance” and weight loss (30 minutes)

This section takes the idea of calorie balance and discusses how we can reach the point where we have more calories out than in, which tips the balance towards weight loss.

Part 4: Wrap up and to-do list (10 minutes)

**Key messages**

- **To lose weight, it is not always enough only to reduce fat and calories or only to do physical activity. Both are needed.**
- **The food we eat and the physical activity we do work together to determine how much we weigh. To lose weight, eat fewer calories and be more active, changing both sides of the balance (in and out) at the same time.**
- **Tipping the calorie balance requires you to keep track of calories every day and stay as close as possible to your fat gram goal.**

*Part 1: Weekly progress and review (10 minutes)*

**Distribute** Session 7 handouts, Session 7 Food and Activity Trackers and Session 5 Food and Activity Trackers with your notes.

**Collect** Session 6 Food and Activity Trackers.

**Discuss** participants’ successes and difficulties in meeting the weight loss goals.

**Present:** Last week, we discussed physical activity and set goals for this week.

**Ask:** Would anyone like to share with the group their successes or their difficulties this past week?

**Open responses.**

**Ask:**

- Did you have any trouble keeping track of your eating and activity last week?
- Were you able to stay within your fat gram budget?
- Did you reach your goal for physical activity?
- Were you able to make the active lifestyle choices you planned?

**Open responses.**

**Present:** Starting a physical activity routine can be challenging. But if you stick with it, it will only get easier. Those of you who found time to be physically active made a choice to be active. Keep up the good work!

**Present:** This week we will talk about the role of calories and how to get to a “calorie balance”. We will also talk about the relationship between that calorie balance and weight loss.

*Part 2: What is “calorie balance”? (10 minutes)*

**Present:** During the last six sessions, we spent a lot of time talking about healthy eating and physical activity. We know that both of these habits are important for a healthy lifestyle and for helping to prevent heart disease and other diseases.

Today, we’re going to talk about another reason why healthy eating and physical activity are important to us: they are directly related to weight loss.

The “calorie balance” is the difference between the calories you eat and drink, and the calories you use up by physical activity and burn at rest. If we take in the same number of calories as we use, our weight stays the same. The calories-in and the calories-out are in balance.

However, if we take in more calories than we use, our body stores that extra energy as fat, and we gain weight. The opposite is also true. If we take in fewer calories than we use, our weight will go down.

**Present:** When you eat food or drink something other than water, you take in energy in the form of calories. Calories come from fat, carbohydrates (sugar and starch) and protein.

**Refer** to the “Understanding the calorie balance” handout.

**Present:** As you can see in the handout, there can be big differences in how many calories are in different kinds of foods.

Remember that fat has the highest concentration of calories. Each gram of fat has 9 calories, which is more than twice the amount in a gram of carbohydrate or a gram of protein.

Other ingredients and foods do not have many calories. For example, green, leafy vegetables have few calories because they are mostly water and fiber.

One reason we have been working to eating less fat is because it has more calories than other foods. Fat is where most calories in meat come from.

**Present:** We need to eat some calories. Our body needs them to survive. It gives us the energy to do everything we need to do, including breathing.

We talked about the calories we eat and drink, now we will talk about the calories we use in physical activity. The number of calories we use depends on several things:

The level of activity (light, moderate, intense)

The amount of time that you are active.

How much you weight.

**Present:** Here’s one way to think about calorie balance: it would take more energy for you to walk a kilometer with two heavy bags than if you weren’t carrying anything.

One idea you can use is that 1.5 kilometers of brisk walking (which takes most people 20 minutes) will use about 100 calories.

*Part 3: The calorie balance and weight loss (30 minutes)*

**Present:** So what does all this mean? It means that your weight over time is determined by the balance between food and drink (calories in) and activity (calories out).

**Refer** to the “Balancing Food and Activity” handout.

**Present:** This handout shows how the balance can affect you in different ways.

1. Weight stays the same. Calories in (food) equals calories out (activity).
2. Weight increases: Calories in (food) is more than calories out. Either the calories-in increased, or the calories out decreased, or both.
3. Weight decreases: Calories in (food) is less than calories out. Either the calories-in decreased, or the calories out increased, or both.

We reach a new balance at a new weight. When you lose weight and keep it off, or new balance requires us to maintain new eating habits and a new activity level.

**Present:** What is important to remember is that:

- Food and activity work together to determine how much we weigh. To lose weight, we need to tip the balance by increasing the number of calories-out (activity) or decreasing the number of calories-in (food and drink), or both.
- To lose weight, it is better to do both. Eat fewer calories AND be more active. This will not only help you to lose weight, but it will also improve your health.

By tipping the balance toward fewer calories-in and more calories-out, you can lose all the weight you want to lose.

We will help you to make the changes a part of your lifestyle, so that you will keep the weight off.

Tipping the balance

**Present:** Now you know what the “calorie balance” is. But how does it work in terms of numbers? How many fewer calories-in and how much physical activity is needed to tip the balance toward losing weight?

**Refer** to the “Tipping the balance” handout.

Note: A good example for the following discussion is a kilogram of actual fat.

**Present:** The number of calories you need to eat, or the amount of activity you need to do, is different from person to person. But two facts help us to think about the answer to the questions.

One kilogram of fat stores about 7,700 calories.

The best way to lose body fat is slow and steady weight loss (about half to one kilogram a week).

**Use the whiteboard.**

**Present:** As the handout shows, to lose half a kilogram in one week, you need to tip your calorie balance by 3,850 calories in 7 days, which is 550 calories a day. You have to use 550 calories more than you take in.

To lose one kilogram in a week, tip the calorie balance by 7,700 calories in 7 days, which is 1,100 calories a day. You have to use 1,100 calories more than you take in.

**Present:** Remember, the best way to tip the calorie balance to lose weight is to decrease food intake AND increase physical activity.

It is generally recommended that people who are not as close to their weight loss goal aim to lose 1 kilogram a week and that people who are closer to their weight loss goal aim to lose half a kilogram a week.

No one should eat less than 1,200 calories per day. This can be harmful to your health.

Quick losses of large amounts of weight can mean that water or muscle is being lost rather than fat, and that is also harmful to your health.

Review of your progress

**Present:** Now let’s take a minute to look at some positive changes in balance you have made so far.

**Refer** to “Review of your calorie balance” handout.

**Ask:** First, what changes have you made to be more active?

**Participants should write their answers in the space on the handout. They can share their responses, if they feel comfortable.**

Note: Remember to discuss both planned activity and lifestyle activity (walking up stairs, parking farther from their destination).

Praise all decreases in calories and increases in activity.

Encourage participants to keep up all positive changes.

**Ask:** We focused on eating less fat because fat is the most concentrated source of calories. What changes have you made to eat less fat (and fewer calories)?

**Open responses.**

**Ask** for volunteers to share their responses. Briefly discuss the responses.

**Present:** Now, let’s take a closer look at how these changes have helped you tip the calorie balance to lose weight.

**Refer** to the starting weight, and look at the weight now. Find the expected weight by this time* this section needs some work. Smartphone app?

**Present:** If you have:

1. Stayed the same weight, or gained weight: we will work together to try something else to tip the balance.
2. Lost some weight, but less than our goal: You have made progress. We will try something else to tip the balance further.
3. Lost as much weight as expected (or more): Great! You have tipped the balance. Keep going.

Note: If you find that many participants did not lose as much weight as expected, present the suggestion below. Use your judgment about how to address each person’s specific needs.

**Present:** If your weight loss is not as much as you expected, let’s think back to the steps we have talked about so far. We need to track your food and activity. Are you forgetting to write down any food or drink that you’re consuming? This might help us understand where we can tip the balance farther. If you’re tracking your food and activity, let’s see where we can reduce calories-in and increase calories-out.

If you need to refresh your memory about tracking the food and drink you eat, I have some copies of the handout “Tracking Fat and Calories”.

*Part 4: Wrap up and to-do list (10 minutes)*

To do next week

**Ask** if there are any questions or concerns about what has been covered this week.

**Present:** Now let’s make an activity plan for next week.

The goal is to do a little more physical activity than last week, for a weekly total of 120 minutes of activity. By doing more activity, you will use more calories- which will help you tip the balance toward weight loss.

For next week:

- As before, try setting aside one block of time each day to be active.
- If you cannot do that, look for smaller blocks of at least 10 minutes during the day. To get to 120 minutes a week, we need 12 blocks of 10 minutes in 7 days.
- Fill out the “To do next week” handout with your activity plan for next week.

Closing

**Summarize key points:**

- **The calorie balance is the difference between the calories you take in through eating and drinking and the calories you use through physical activity, lifestyle activity and your body’s natural energy needs.**
- **Food and activity work together to determine how much you weigh. To lose weight, eat fewer calories and be more active. By doing both, you are improving both sides of the balance at once which increases the amount of weight you will lose.**
- **One and a half kilometers of brisk walking (which takes most people 15-20 minutes) uses about 100 calories.**

**Close:** Our next session is called “Take charge of what’s around you”. We will be talking about people, events, and situations in our lives that stand in the way of healthy eating and physical activity. And we will discuss how to remove those barriers.

**Ask** if there are any questions or concerns.

**After the session:**

Write notes on successes and recommended changes on each participant’s “Food and Activity Tracker” from Session 6.
