# Supplementary material for: Emirates Heart Health Project (EHHP): A protocol for a stepped-wedge family-cluster randomized-controlled trial of a health-coach guided diet and exercise intervention to reduce weight and cardiovascular risk in overweight and obese UAE nationals
Source: PLoS One. 2023 Apr 10;18(4):e0282502. doi: 10.1371/journal.pone.0282502 (PMC10085020; doi:10.1371/journal.pone.0282502)
Supplement: S19 Appendix — (DOCX) [file pone.0282502.s019.docx]

**الجلسة 7 :توازن السعرات الحرارية**

**أهداف التعلم**

في ختام هذه الجلسة سيتمكن المشاركون من:

- شرح "توازن السعرات الحرارية".
- شرح كيفية ارتباط الطعام الصحي والنشاط البدني من حيث توازن السعرات الحرارية.
- وصف كيفية تأثير توازن السعرات الحرارية على فقدان الوزن.
- وصف تقدم المشارك أو المشاركةو علاقة تقدمهم بتوازن السعرات الحرارية.
- وضع خطة نشاط للأسبوع القادم.

**المواد**

- نشرات المشاركين للجلسة 7
- نظرة عامة
- فهم توازن السعرات الحرارية
- موازنة الغذاء والنشاط
- قلب الميزان
- مراجعة توازنك من السعرات الحرارية
- بضع نسخ من الجلسة الثانية "تتبع الدهون والسعرات الحرارية"
- قائمة مهام الأسبوع المقبل
- متتبعي الطعام والنشاط للجلسة 7
- بطاقات الأسماء ، إذا لزم الأمر
- السبورة والأقلام

**نظرة عامة**

تجمع الجلسة 7 بين الجلسات الست السابقة التي كانت تتناول الطعام الصحي والنشاط البدني. خلال هذه الجلسة ، نوضح كيف يرتبط هذان الموضوعان وكيف يعملان معًا لفقدان الوزن.

هناك 4 أجزاء:

الجزء الأول: التقدم والمراجعة الأسبوعية (10 دقائق)

الجزء 2: ما هو "توازن السعرات الحرارية"؟ (10 دقائق)

"توازن السعرات الحرارية" هو العلاقة بين السعرات الحرارية في (الأكل) والسعرات الحرارية الخارجة (النشاط). في هذا القسم نستعرض أي مجموعات غذائية أعلى نسبيًا في السعرات الحرارية وكيف نستخدم تلك السعرات الحرارية.

الجزء 3: "توازن السعرات الحرارية" وفقدان الوزن (30 دقيقة)

يتناول هذا القسم فكرة توازن السعرات الحرارية ويناقش كيف يمكننا الوصول إلى النقطة التي نحصل فيها على سعرات حرارية مستهلكة أكثر من داخلة ، مما يحقق التوازن نحو فقدان الوزن.

الجزء الرابع: ختام وقائمة المهام (10 دقائق)

**الرسائل الرئيسية**

- لفقدان الوزن ، لا يكفي دائمًا تقليل الدهون والسعرات الحرارية أو القيام بنشاط بدني فقط. كلاهما مطلوب.
- الطعام الذي نتناوله والنشاط البدني الذي نقوم به يعملان معًا لتحديد أوزاننا. لفقدان الوزن ، تناول سعرات حرارية أقل وكن أكثر نشاطًا ، مع تغيير جانبي التوازن (داخل وخارج) في نفس الوقت.
- يتطلب قلب ميزان السعرات الحرارية تتبع السعرات الحرارية كل يوم والبقاء على مقربة قدر الإمكان من هدف غرام الدهون.

الجزء الأول: التقدم والمراجعة الأسبوعية (10 دقائق)

**وزع** نشرات الجلسة 7 والجلسة 7 متتبعات الطعام والنشاط والجلسة 5 متتبعات الطعام والنشاط مع ملاحظاتك.

**اجمع** متتبعات الطعام والنشاط للجلسة 6 .

**ناقش** نجاحات المشاركين والصعوبات في تحقيق أهداف فقدان الوزن.

**حاضر:** ناقشنا الأسبوع الماضي النشاط البدني وحددنا أهداف هذا الأسبوع.

**اسأل:** هل يرغب أحد في مشاركة النجاحات أو الصعوبات التي واجهته الأسبوع الماضي مع المجموعة؟

**افتح المجال للرد.**

**اسأل:**

- هل واجهت أي مشكلة في تتبع الأكل والنشاط الأسبوع الماضي؟
- هل كنت قادرًا على البقاء ضمن كمية غرام الدهون الخاصة بك؟
- هل وصلت إلى هدفك في لنشاط البدني؟
- هل تمكنت من اتخاذ خيارات أسلوب الحياة النشطة التي خططت لها؟

**افتح المجال للرد.**

**حاضر:** قد يكون بدء روتين النشاط البدني أمرًا صعبًا. ولكن إذا التزمت به ، فسيصبح أسهل. أولئك الذين وجدوا وقتًا للنشاط البدني اختاروا أن يكونوا نشطين. ثابر على العمل الجيد!

**حاضر:** سنتحدث هذا الأسبوع عن دور السعرات الحرارية وكيفية الوصول إلى "توازن السعرات الحرارية". سنتحدث أيضًا عن العلاقة بين توازن السعرات الحرارية وفقدان الوزن.

**الجزء 2: ما هو "توازن السعرات الحرارية"؟ (10 دقائق)**

**حاضر:** خلال الجلسات الست الماضية ، أمضينا الكثير من الوقت في الحديث عن الأكل الصحي والنشاط البدني. نحن نعلم أن كل من هذه العادات مهمة لنمط حياة صحي وللمساعدة في الوقاية من أمراض القلب والأمراض الأخرى.

اليوم ، سنتحدث عن سبب آخر لأهمية الأكل الصحي والنشاط البدني بالنسبة إلينا: إنهما مرتبطان بشكل مباشر بفقدان الوزن.

"توازن السعرات الحرارية" هو الفرق بين السعرات الحرارية التي تتناولها وتشربها ، والسعرات الحرارية التي تستهلكها من خلال النشاط البدني وتحرقها أثناء الراحة. إذا أخذنا نفس عدد السعرات الحرارية التي نستخدمها ، فإن وزننا يبقى كما هو. السعرات الحرارية الداخلة والسعرات الحرارية الخارجة متوازنة.

ومع ذلك ، إذا تناولنا سعرات حرارية أكثر مما نستخدم، فإن أجسامنا تخزن تلك الطاقة الإضافية كدهون ، ونكتسب الوزن. والعكس صحيح أيضا. إذا تناولنا سعرات حرارية أقل مما نستخدمه ، فسوف ينخفض ​​وزننا.

**حاضر:** عندما تأكل طعامًا أو تشرب شيئًا غير الماء ، فأنت تستهلك طاقة على شكل سعرات حرارية. تأتي السعرات الحرارية من الدهون والكربوهيدرات (السكر والنشا) والبروتين.

**ارجع** إلى نشرة "فهم رصيد السعرات الحرارية".

**حاضر:** كما ترى في النشرة ، يمكن أن تكون هناك اختلافات كبيرة في عدد السعرات الحرارية الموجودة في أنواع مختلفة من الأطعمة.

تذكر أن الدهون تحتوي على أعلى تركيز للسعرات الحرارية. يحتوي كل جرام من الدهون على 9 سعرات حرارية ، وهو أكثر من ضعف الكمية في جرام من الكربوهيدرات أو جرام من البروتين.

المكونات والأطعمة الأخرى لا تحتوي على الكثير من السعرات الحرارية. على سبيل المثال ، تحتوي الخضار الورقية على سعرات حرارية قليلة لأنها في الغالب تتكون من الماء والألياف.

أحد أسباب عملنا على تناول كميات أقل من الدهون هو أنها تحتوي على سعرات حرارية أكثر من الأطعمة الأخرى. الدهون هي مصدر معظم السعرات الحرارية في اللحوم.

**حاضر**: نحتاج إلى تناول بعض السعرات الحرارية. جسمنا يحتاجهم للبقاء. يمنحنا الطاقة للقيام بكل ما نحتاجه ، بما في ذلك التنفس.

تحدثنا عن السعرات الحرارية التي نأكلها ونشربها ، والآن سنتحدث عن السعرات الحرارية التي نستخدمها في النشاط البدني. يعتمد عدد السعرات الحرارية التي نستخدمها على عدة أشياء:

- مستوى النشاط (خفيف ، معتدل ، مكثف)
- مقدار الوقت الذي تستغرقه في النشاط.
- الوزن.

**حاضر:** إليك طريقة واحدة للتفكير في توازن السعرات الحرارية:ستحتاج لطاقة أكبر للسير لمسافة كيلومتر بحقيبتين ثقيلتين مما لو لم تكن تحمل أي شيء.

إحدى الأفكار التي يمكنك استخدامها هي أن 1.5 كيلومتر من المشي السريع (الذي يستغرق معظم الأشخاص 20 دقيقة) سيستخدم حوالي 100 سعر حراري.

الجزء 3: توازن السعرات الحرارية وفقدان الوزن 30 دقيقة

**حاضر:** فماذا يعني كل هذا؟ هذا يعني أن وزنك بمرور الوقت يتم تحديده من خلال التوازن بين الطعام والشراب (السعرات الحرارية في) والنشاط (السعرات الحرارية الخارجة).

**راجع** نشرة موازنة الغذاء والنشاط.

**اشرح**: توضح هذه النشرة كيف يمكن أن يؤثر التوازن عليك بطرق مختلفة.

- للحفاظ على الوزن كما هو. السعرات الحرارية في (الغذاء) تساوي السعرات الحرارية في (النشاط).
- زيادة الوزن: السعرات الحرارية في (الطعام) أكثر من السعرات الحرارية في النشاط إما زيادة السعرات الحرارية الداخلة ، أو انخفاض السعرات الحرارية الخارجة ، أو كليهما.
- نقص الوزن: السعرات الحرارية في (الطعام) أقل من السعرات الحرارية في(النشاط). إما انخفاض السعرات الحرارية الداخلة ، أو زيادة السعرات الحرارية الخارجة ، أو كليهما.

نصل إلى توازن جديد بوزن جديد. عندما تفقد الوزن وتحافظ عليه ، أو يتطلب التوازن الجديد الحفاظ على عادات غذائية جديدة ومستوى نشاط جديد.

**حاضر**: المهم أن نتذكر ما يلي:

- يعمل الطعام والنشاط معًا لتحديد وزننا. لفقدان الوزن ، نحتاج إلى قلب التوازن عن طريق زيادة عدد السعرات الحرارية الخارجة (النشاط) أو تقليل عدد السعرات الحرارية (الطعام والشراب) ، أو كليهما.
- لفقدان الوزن ، من الأفضل القيام بالأمرين معًا. تناول سعرات حرارية أقل وكن أكثر نشاطًا. لن يساعدك ذلك على إنقاص الوزن فحسب ، بل سيحسن صحتك أيضًا.

عن طريق تحويل التوازن نحو سعرات حرارية أقل داخلة والمزيد من السعرات الحرارية الخارجة ، يمكنك أن تفقد كل الوزن الذي تريد أن تخسره.

سنساعدك على جعل التغييرات جزءًا من نمط حياتك ، حتى تتمكن من الحفاظ على الوزن.

**قلب الميزان**

**حاضر:** أنت تعرف الآن ما هو "توازن السعرات الحرارية". ولكن كيف يعمل من حيث الأعداد؟ كم عدد السعرات الحرارية الداخلة وكم من النشاط البدني المطلوب لقلب التوازن نحو فقدان الوزن؟

**راجع** نشرة "قلب التوازن".

**ملاحظة:** مثال جيد للمناقشة التالية هو كيلوغرام من الدهون الفعلية.

**حاضر:** يختلف عدد السعرات الحرارية التي تحتاج إلى تناولها ، أو مقدار النشاط الذي تحتاج إلى القيام به ، من شخص لآخر. لكن هناك حقيقتان تساعداننا على التفكير في إجابة الأسئلة.

- كيلو واحد من الدهون يخزن حوالي 7700 سعرة حرارية.
- أفضل طريقة لفقدان دهون الجسم هي فقدان الوزن البطيء والثابت (حوالي نصف كيلوجرام في الأسبوع).

**استخدم السبورة.**

**حاضر**: كما تظهر النشرة ، لفقدان نصف كيلوغرام في أسبوع واحد ، تحتاج إلى قلب رصيدك من السعرات الحرارية بمقدار 3850 سعرة حرارية في 7 أيام ، وهو 550 سعرة حرارية في اليوم. عليك استخدام 550 سعرة حرارية أكثر مما تتناوله.

لفقد كيلوغرام واحد في الأسبوع ، قم بقلب رصيد السعرات الحرارية بمقدار 7،700 سعر حراري في 7 أيام ، وهو 1100 سعر حراري في اليوم. يجب عليك استخدام 1100 سعر حراري أكثر مما تأخذ.

**حاضر**: تذكر أن أفضل طريقة لتقليل توازن السعرات الحرارية لفقدان الوزن هي تقليل تناول الطعام وزيادة النشاط البدني.

يُنصح عمومًا بأن الأشخاص الذين ليسوا قريبين من هدف فقدان الوزن يهدفون إلى فقدان كيلوغرام واحد في الأسبوع وأن الأشخاص الأقرب إلى هدف فقدان الوزن يهدفون إلى فقدان نصف كيلوغرام في الأسبوع.

لا يجب أن يأكل أحد أقل من 1200 سعر حراري في اليوم. يمكن أن يكون ذلك ضارًا بصحتك.

يمكن أن تعني الخسائر السريعة لكميات كبيرة من الوزن فقدان الماء أو العضلات بدلاً من الدهون ، وهذا أيضًا ضار بصحتك.

مراجعة تقدمك

**حاضر:** الآن لنأخذ دقيقة لنلقي نظرة على بعض التغييرات الإيجابية في التوازن التي أجريتها حتى الآن.

**ارجع** إلى نشرة "مراجعة رصيد السعرات الحرارية".

**اسأل:** أولاً ، ما هي التغييرات التي أجريتها لتكون أكثر نشاطًا؟

**يجب على المشاركين كتابة إجاباتهم في الفراغ على النشرة. يمكنهم مشاركة ردودهم ، إذا شعروا بالراحة.**

**ملاحظة:** تذكر أن تناقش كلاً من النشاط المخطط ونشاط نمط الحياة (صعود الدرج ومواقف السيارات البعيدة عن وجهتها).

امتدح جميع الانخفاضات في السعرات الحرارية والزيادات في النشاط.

شجع المشاركين على الاستمرار على جميع التغييرات الإيجابية.

**اسأل:** ركزنا على تناول كميات أقل من الدهون لأن الدهون هي أكثر مصدر للسعرات الحرارية تركيزًا. ما التغييرات التي أجريتها على تناول كمية أقل من الدهون (وقليل من السعرات الحرارية)؟

**افتح المجال للرد**

**اطلب** متطوعين لمشاركة ردودهم. ناقش الردود باختصار.

**حاضر**: الآن ، دعنا نلقي نظرة عن قرب على الكيفية التي ساعدت بها هذه التغييرات على قلب توازن السعرات الحرارية لفقدان الوزن.

راجع وزنك في البداية ، وانظر إلى الوزن الآن. ابحث عن الوزن المتوقع بحلول هذا الوقت * يحتاج هذا القسم إلى بعض العمل. تطبيق الهاتف الذكي؟

**حاضر:** إذا كان لديك:

1. حافظت على نفس الوزن ، أو اكتسبت وزنًا: سنعمل معًا لتجربة شيء آخر لإثبات التوازن.

2. فقدت بعض الوزن ، ولكن أقل من هدفنا: لقد أحرزت تقدما. سنحاول شيئًا آخر لزيادة التوازن.

3. خسرت الكثير من الوزن كما هو متوقع (أو أكثر): عظيم! لقد قلبت التوازن. واصل التقدم.

**ملاحظة:** إذا وجدت أن العديد من المشاركين لم يفقدوا الكثير من الوزن كما هو متوقع ، فقدم الاقتراح أدناه. استخدم حكمك حول كيفية تلبية الاحتياجات الخاصة لكل شخص.

**حاضر:** إذا كان فقدان الوزن ليس بالقدر الذي توقعته ، فلنعد إلى الخطوات التي تحدثنا عنها حتى الآن. نحن بحاجة لتتبع طعامك ونشاطك. هل نسيت كتابة أي طعام أو شراب تستهلكه؟ قد يساعدنا ذلك على فهم أين يمكننا أن نضع التوازن . إذا كنت تتبع طعامك ونشاطك ، فلنرى أين يمكننا تقليل السعرات الحرارية وزيادة السعرات الحرارية.

إذا كنت بحاجة إلى تحديث ذاكرتك بشأن تتبع الطعام والشراب الذي تتناوله ، فلدي بعض النسخ من نشرة "تتبع الدهون والسعرات الحرارية".

**الجزء الرابع: ختام وقائمة المهام (10 دقائق)**

قائمة مهام الأسبوع المقبل

**اسأل** عما إذا كانت هناك أي أسئلة أو مخاوف بشأن ما تم تغطيته هذا الأسبوع.

**حاضر:** الآن دعونا نضع خطة نشاط للأسبوع القادم.

الهدف هو القيام بنشاط بدني أكثر بقليل من الأسبوع الماضي ، بمجموع أسبوعي 120 دقيقة من النشاط. من خلال القيام بمزيد من الأنشطة ، ستستخدم المزيد من السعرات الحرارية - مما سيساعدك على قلب التوازن نحو فقدان الوزن.

للأسبوع القادم:

- حاول تخصيص جزء واحد من الوقت كل يوم لتكون نشطًا.
- إذا لم تتمكن من فعل ذلك ،حاول أن تقوم بالنشاط على فترات لمدة 10 دقائق على الأقل خلال اليوم. للوصول إلى 120 دقيقة في الأسبوع ، نحتاج إلى 12 فترة من 10 دقائق في 7 أيام.
- املأ نشرة "المهام الأسبوع المقبل" بخطة نشاطك للأسبوع المقبل.

**الخاتمة**

**تلخيص النقاط الرئيسية:**

- **توازن السعرات الحرارية هو الفرق بين السعرات الحرارية التي تتناولها من خلال الأكل والشرب والسعرات الحرارية التي تستخدمها من خلال النشاط البدني ونشاط نمط الحياة واحتياجات جسمك من الطاقة الطبيعية.**
- **يعمل الطعام والنشاط معًا لتحديد مقدار وزنك. لفقدان الوزن ، تناول سعرات حرارية أقل وكن أكثر نشاطًا. من خلال القيام بكلاهما ، فأنت تقوم بتحسين جانبي التوازن في وقت واحد مما يزيد من مقدار الوزن الذي ستفقده.**
- **يستخدم كيلومتر ونصف من المشي السريع (الذي يستغرق معظم الأشخاص من 15 إلى 20 دقيقة) حوالي 100 سعره حرارية.**

**اختتام:** الجلسة التالية تسمى " تولى مسؤولية ما حولك". سوف نتحدث عن الناس والأحداث والمواقف في حياتنا التي تقف في طريق الأكل الصحي والنشاط البدني. وسنناقش كيفية إزالة تلك الحواجز.

**اسأل** عما إذا كانت هناك أي أسئلة أو استفسارات.

**بعد الجلسة:**

اكتب ملاحظات عن النجاحات والتغييرات الموصى بها على "متتبع الغذاء والنشاط" لكل مشارك من الجلسة 6.
