# Supplementary material for: Emirates Heart Health Project (EHHP): A protocol for a stepped-wedge family-cluster randomized-controlled trial of a health-coach guided diet and exercise intervention to reduce weight and cardiovascular risk in overweight and obese UAE nationals
Source: PLoS One. 2023 Apr 10;18(4):e0282502. doi: 10.1371/journal.pone.0282502 (PMC10085020; doi:10.1371/journal.pone.0282502)
Supplement: S20 Appendix — (DOCX) [file pone.0282502.s020.docx]

**Session 8: Take charge of what’s around you**

**Learning objectives**

By the end of this session, the participants will know how to:

- Recognize positive and negative food and activity cues.
- Change negative food and activity cues to positive cues.
- Add positive cues for activity and eliminate cues for inactivity.
- Develop a plan for removing one negative food cue for the coming week.

**Materials**

- Handouts
  - Session 8 overview
  - What makes us eat?
  - Changing food cues and habits
  - Shopping tips
  - Activity cues
  - To-do for next week
- Food and Activity Trackers for Session 8
- Name tags
- Whiteboard with markers

**Session 8 overview**

Session 8 focuses on how our surroundings and environment can affect behavior. The concepts of “food cues” and “activity cues” are introduced in order to prompt a discussion about factors in our environment that can lead us, either positively or negatively, to make choices about what and how much we eat and how active we are. The main message is that when respond the same way to a food or activity cue over and over again, we develop a habit.

This session will require participants to visualize or imagine their homes, workplaces and other places to become aware of their negative cues and how to either eliminate them or replace them with positive cues.

Session 8 is divided into 4 parts:

*Part 1: Weekly progress and review (10 minutes)*

*Part 2: Food cues (25 minutes)*

Negative food cues are events, situation, or people that lead us to eat when we are not hungry. Participants will look for their personal negative food cues and work together as a group to come up with ways to eliminate them or to come up with alternative, healthier cues.

*Part 3: Activity cues (15 minutes)*

Just as negative food cues lead us to overeat, negative activity cues lead us to be inactive. For some people, after dinner, they go out of habit and watch TV. Participants will look for their own personal negative activity cues and work together to come up with positive alternatives.

*Part 4: Wrap up and to-do list (10 minutes)*

**Key messages**

- **It is important to become aware of the many factors that influence our behavior related to eating and activity, some in positive ways and some in negative ways.**
- **The good news is that we can make food and activity cues work for us and help us, instead or working against us.**
- **Not all food and activity cues are bad. If cues get in the way of our efforts to eat less fat and fewer calories or be more active, then they are a problem. However, we can start new, healthier habits by adding positive cues and eliminating negative cues.**
- **It is important to build positive food and activity cues into our daily routine.**

*Part 1: Weekly progress and review (10 minutes)*

**Distribute:** Session 8 handouts and Session 6 Food and Activity Trackers with your notes.

**Collect** Session 7 Food and Activity Trackers.

**Discuss** participants’ successes and challenges in the past week.

**Present:** Last week, we talked about balancing the calories you take in through eating and drinking with the calories you use during physical activity. Food and physical activity work together to control your weight. To lose weight, eat fewer calories and be more active.

**Ask:** How many of you reached your goal for physical activity last week?

**Open responses.**

**Ask:** Were you able to make active lifestyle choices you planned? Why or why not?

**Open responses.**

**Ask:** If you decided to lower your calorie goal or fat gram goal, were you able to do so?

**Open responses.**

**Address** any issues or confusion about what participants were asked to do during the past week.

**Present:** This week we will:

- Step back and take a look at what causes us to want to eat and avoid physical activity. These triggers are called “cues”.
- Look at ways to change problem food and activity cues.
- Find ways to add positive cues for activity and eliminate cues for inactivity.

*Part 2: Food cues (25 minutes)*

Defining cues

**Present:** At this point in the program, we want to help you understand some basic ideas about behavior. We are going to talk about factors that have a powerful effect on how much we eat, what we eat, and how much we move.

Scientists who study these factors call them “Cues.” Cues can be positive or negative. Cues are a problem when they cause us to make unhealthy choices like overeating or being physically inactive.

We will talk about food cues: factors that affect how much and what we eat, and activity cues: factors that affect how active we are.

We are not only going to talk about how cues affects us, but also, and more importantly, how each of us can take control of those cues so that they help us instead of preventing us reach our goals.

Cues that make us eat

**Present:** Let’s start with food cues.

**Ask:** What cues make you want to eat?

**Open responses.**

**Refer** participants to the “What makes us eat?” handout.

**Offer** these suggestions:

- *Hunger:* of course, one reason we eat is because we’re hungry.
- *Thinking or feeling:* you might eat because you feel lonely, bored, happy or sad.
- *Other people:* you might eat something because someone offers it to you or everyone else is eating it. Example: visiting other people, weddings.
- *Sight or smell of food:* one of the most powerful cues. Example: you see ice cream on Snapchat and immediately want some.
- *Certain activities* such as watching TV or going to the farm may make you think about eating.

**Ask:** Is anyone willing to offer an example of a personal food cue?

**Open responses.**

**Present:** For example, why do we eat when we go to the mall?

**Open responses.**

**Present:** Do you think it is because we are actually hungry? Most of the time, it is because it is a habit. When you respond to a food cue in the same way over and over again, you build a habit. Once it is a habit, we respond to the food cue in a way that becomes more and more automatic.

When eating at the mall becomes a habit, it is hard to go to the mall and not eat something.

**Ask:** Does anyone have an example for eating habits that you have formed and now are hard to break?

**Open responses.**

Common problem cues in the home.

**Present:** Food cues are not always bad. For example, hunger is a positive cue. If we did not have hunger, our ancestors might have died from not eating. Yet many food cues are negative because now in modern times we need to eat less fat and fewer calories than our ancestors did.

**Ask:** How do you think you can change problem food cues and habits?

**Open responses.**

**Refer** participants to the “Changing food cues and habits” handout.

**Present:** You can try these ideas:

- One of the best ways is to stay away from the food cue or keep it out of sight.
  - Stay away from the food court at the mall.
- Or you can build a new, healthier habit.
  - You can decide on a healthier restaurant or food choice before you go to the mall.

**Present:** Remember it takes time to break an old habit or build a new one. Change does not happen in one day.

It is important to become aware of the many events and situations that have a strong influence on our eating and activity behavior in both positive and negative ways. Think for a minute about the many food cues that are all around us. Often we are not aware of how powerful they are.

**Ask:** What are some examples of food cues around us that are more powerful than we might think?

**Open responses.**

**Offer** these suggestions:

- Food trucks
- Talabat and Jeebly
- Baqala
- Supermarkets put new products on the shelves that are the easiest to see and reach. They know that the easier it is to see and pick up the product, the more likely we are to buy it.

Breaking your problem cues

**Present:** Let’s talk about some of *your* problem cues and discuss some ways you can change them.

We will start with where you live. Imaging that we have just come into your house. Let’s cisualize what is in the first room you enter, and then we will talk about what we see. Which room are you in? Is there any food in the room? Do you see anything else that might make you think about eating, such as a comfortable chair, or a memory of a party?

Move from room to room. Are there other rooms that have cues that make you want to eat?

**Ask:** Would someone share a problem cue during their trip around the house? What change could you make to stay away from that cue or to build a new and healthier habit?

**Open responses.**

**Note:** Write each problem cue on the whiteboard and have the group come up with solutions.

If participants do not respond, have them review their “Food and activity Tracker” and ask if that helps them think of any problem cues.

**Offer** these suggestions if they were not already suggested.

- TV/computer/mobile
  - Make it a rule never to eat while on a device; this can lead to mindless eating and eating even when we are full.
  - Keep an exercise bicycle near the TV.
  - Allow yourself only gum while you are on a device.
- Candy/sweets
  - Do not bring them into the house.
  - If you do bring them into your home, keep them out of sight.
  - Only buy the amount that you will eat at that time.
  - Buy smaller sizes of candies.
- High-fat and high-calorie foods in kitchen
  - Stop buying these foods altogether.
  - Store them out of sight, in an unattractive container like a brown bag or unmarked box.
  - Make them hard to reach.
  - Keep low-fat, low-calorie food easy to reach, in sight and ready to eat. (Example, cut up vegetables in the refrigerator.)
- Foods you cook or eat as leftovers
  - Make it a rule or habit not to eat while cooking.
  - When cooking, taste food only once for the flavor. Then rinse your mouth with water.
  - Ask someone else to taste the food.
- Dinner table
  - Use small plates or bowls.
  - Serve small portions.
  - Eat slowly and chew thoroughly.

Common cues at work

**Present:** You have looked at your home and found some problem food cues.

**Ask:** For those of you who work, what about where you work? Is there anything on your way to work, at work, or on your way home from work that could be a problem food cue?

**Open responses.**

**Offer** these suggestions if they have not already been suggested:

- Fast food restaurant, food truck
  - Take a different route to work.
  - Make it a rule not to eat in the car.
  - Prepare a healthy lunch or breakfast that is ready to go before you leave the house and when you return.
- High-fat and high-calorie foods in public areas (take out, candy on a co-worker’s desk)
  - Avoid those areas
  - Attempt to keep those foods out of sight.
  - Bring low-fat and low-calorie snacks to share with co-workers instead.
    - Apples, raw carrots, pretzels, low-fat popcorn.
- Vending machines
  - Stay away from vending machines.
  - Bring low-fat and low-calorie snacks from home.
  - If you must buy, choose a lower-fat, lower-calorie choice.

**Present:** The important things to remember are:

- Keep high-fat and high-calorie foods out of your house and workplace, or keep them out of sight.
- Keep low-fat and low-calorie foods easy to reach, in sight, and ready to eat.
  - Fresh fruit, raw vegetables, washed and cut, low fat popcorn, water.
- Limit your eating to one place.
  - Don’t eat in front of the TV.
- When you eat, limit other activities.
  - Eat your meal without being on a device, driving.
  - Focus on enjoying the meal.

Common problem shopping cues

**Present:** Finally, let’s take a look at where you usually shop for food.

**Ask:** Who is responsible in the family to shop for food?

**Present:** Visualize walking around the store or souk as you normally do.

**Ask:** What do you see that is a problem for you or your family?

**Open responses.**

**Refer** participants to the “Shopping tips” handout.

**Present:** You do not have as much control over what foods are in the grocery store as you do in your home, but there are some things you can do.

Here are some suggestions:

- Make a shopping list ahead of time. Make it a rule not to but anything that is not on the list.
- Do not go shopping when you are hungry. Have a low-fat or low-calorie snack first.
- Avoid sections of the store that are tempting to you, if possible. Avoid the bakery or ice cream/candy sections, for example.

**Ask:** Are there any other problem food cues that you would like to discuss?

**Open responses.**

**Present:** Now, let’s look at the kinds of cues that can cause us to be inactive.

*Part 3: Activity cues (15 minutes)*

**Present:** Many cues in our environment can lead to being inactive.

For example, after dinner you may automatically sit down in front of the television. That is because you paired finishing dinner and then watching television many times in the past. Remember, though, that you do have a choice. You can change this pattern and choose to take a walk after dinner instead of watching television or watching videos on your mobile.

If you have been inactive for a while, you probably have many cues that trigger inactivity and few cues that trigger activity.

To become active regularly, you must add positive activity cues to your life. Over time, the cues will lead to new habits and more activity. It will become easier over time as your habits become more and more automatic.

**Ask:** What are some positive activity cues that you could acquire?

Let’s begin by visualizing where you live again.

**Ask:** What could you add to the living room that would prompt you to be more active? What about the bedroom? In the kitchen?

**Refer** participants to the “Activity cues” handout.

**Offer** these examples of positive cues:

- In the living room or the bedroom:
  - Keep exercise shoes and equipment in sight (not in the closet).
  - Hang an activity calendar and note when you’ve been active that day.
  - Keep a stationary bike or exercise mat in front of the television.
  - Hang a photo of an outdoor scene or of people being active.
  - Put a note on the television reminding you that a half hour of time watching television could be used for exercise instead.
- In the kitchen:
  - Post notes to be active.
- Use your mobile phone to schedule time to be active.

Removing inactivity cues

**Ask:** What are some positive cues that you could add to your workplace?

**Open responses.**

- Instead of asking your assistant to get something for you, go yourself.
- Put a note on your office door or computer to take a walk.
- Set an alarm on your phone or computer to remind you to move around every hour.

**Present:** There are cues to help you be active that are not dependent on your surroundings.

You can set up a regular date to be active with a family member. Committing to others can help you be active even when you don’t feel motivated or have energy. You can remind each other that “Hey, it’s time to walk!”

**Ask:** Can you think of any other activity cues that we have not discussed?

**Open responses.**

**In summary,** it takes time to break old habits and build new, healthier ones. But it can be done, and you can do it! The most important steps are to find your problem cues and eliminate them, or even better, replace them with new and healthier ones.

You can make food and activity cues work for you, not against you.

*Part 4: Wrap up and to-do list (10 minutes)*

**Ask** if there are any questions about what was covered during this session.

**Present:** Here is what I want you to do for next week: Let’s make a plan for each one of us to remove one negative food cue from our lives.

**Refer** participants to the “To do next week” handout.

Think of one problem good cue that you will get rid of before next week. Think about any problems that you can see with getting rid of it, and how you will overcome them.

Write down the problem cue and what you will do to remove it.

Bonus: think of one positive cue for being more physically active that you can add. Remember the calorie balance. If we can improve the calories-in and the calories-out, we will see better results. Write down your positive cue for being more active, and again, think about what problems you might have in adding this, and how you will solve them.

For next week:

Continue to find ways to be physically active. Most of you have slowly built up to the 150 minutes a week for physical activity.

Remember, the time can be divided into smaller segments such as 20 or even 10 minutes at a time.

Keep track of your weight, eating and activity. Do your best to reach your goals!

**Summarize the key points:**

- **We looked at the kinds of food and activity cues that cause us to behave in unhealthy ways.**
- **We talked about how to change those cues, by avoiding those that stand in the way of our goals and replacing them with positive cues.**
- **We worked on a plan for how to begin removing negative food and activity cues.**

**Close: You have made it through the first half of this program! Keep up the good work!**

Our next session is called “Problem solving.” We will be talking about five steps we can use to solve problems that we face.

**Ask** participants whether they have any questions before closing the session.

**After the session:**

**Review** and write notes on successes and recommendations for the Food and Activity Trackers from Session 7.
