# Supplementary material for: Emirates Heart Health Project (EHHP): A protocol for a stepped-wedge family-cluster randomized-controlled trial of a health-coach guided diet and exercise intervention to reduce weight and cardiovascular risk in overweight and obese UAE nationals
Source: PLoS One. 2023 Apr 10;18(4):e0282502. doi: 10.1371/journal.pone.0282502 (PMC10085020; doi:10.1371/journal.pone.0282502)
Supplement: S21 Appendix — (DOCX) [file pone.0282502.s021.docx]

الجلسة 8: تولى مسؤولية ما حولك

**أهداف التعلم**

في نهاية هذه الجلسة ، سيعرف المشاركون كيفية:

- التعرف على إشارات الطعام والنشاط الإيجابية والسلبية.
- القيام بتغيير إشارات الطعام والنشاط السلبية إلى إشارات إيجابية.
- اضافة إشارات إيجابية للنشاط والتخلص من الإشارات لعدم النشاط.
- وضع خطة لإزالة تلميح سلبي واحد للطعام للأسبوع القادم.

**المواد**

- المنشورات
- نظرة عامة على الجلسة 8
- ما الذي يجعلنا نأكل؟
- تغيير العادات الغذائية
- نصائح التسوق
- إشارات النشاط
- ماذا سنفعل في الأسبوع المقبل
- متتبعي الطعام والنشاط للجلسة 8
- علامات الأسماء
- سبورة بيضاء وأقلام

**نظرة عامة على الجلسة 8**

تركز الجلسة 8 على كيفية تأثير محيطنا وبيئتنا على السلوك. يتم تقديم مفاهيم "إشارات الطعام" و "إشارات النشاط" من أجل إثارة نقاش حول العوامل في بيئتنا التي يمكن أن تقودنا ، سواء إيجابًا أو سلبًا ، إلى اتخاذ خيارات حول ما نأكل وكم نأكل ومدى نشاطنا . الرسالة الرئيسية هي أنه عندما تستجيب بنفس الطريقة لطعام أو نشاط مرارًا وتكرارًا ، فإننا نطور عادة.

تتطلب هذه الجلسة من المشاركين تصور أو تخيل منازلهم وأماكن عملهم والأماكن الأخرى ليكونوا على دراية بإشاراتهم السلبية وكيفية التخلص منها أو استبدالها بإشارات إيجابية.

تنقسم الجلسة 8 إلى 4 أجزاء:

الجزء الأول: التقدم والمراجعة الأسبوعية (10 دقائق)

الجزء الثاني: إشارات الطعام (25 دقيقة)

إشارات الطعام السلبية هي أحداث أو مواقف أو أشخاص يدفعوننا لتناول الطعام عندما لا نكون جائعين. سيبحث المشاركون عن إشاراتهم الغذائية السلبية الشخصية وسيعملون معًا كمجموعة للتوصل إلى طرق للقضاء عليها أو للتوصل إلى إشارات بديلة وصحية.

الجزء 3: إشارات النشاط (15 دقيقة)

تمامًا كما تقودنا إشارات الطعام السلبية إلى الإفراط في تناول الطعام ، فإن إشارات النشاط السلبية تقودنا إلى عدم النشاط. بالنسبة لبعض الناس ، بعد العشاء ، يشاهدون التلفاز. سيبحث المشاركون عن إشارات نشاطهم السلبي الشخصي ويعملون معًا للتوصل إلى بدائل إيجابية.

الجزء الرابع: ختام وقائمة المهام (10 دقائق)

**الرسائل الرئيسية**

من المهم أن ندرك العوامل العديدة التي تؤثر على سلوكنا المتعلق بالأكل والنشاط ، بعضها بطرق إيجابية والبعض الآخر بطرق سلبية.

الخبر السار هو أنه يمكننا أن نجعل إشارات الطعام والنشاط تعمل لصالحنا وتساعدنا بدلاً من ذلك أو تعمل ضدنا.

ليست كل إشارات الطعام والنشاط سيئة. إذا كانت الإشارات تعوق جهودنا لتناول كميات أقل من الدهون والسعرات الحرارية الأقل أو أن تكون أكثر نشاطًا ، فهذا يعني أنها مشكلة. ومع ذلك ، يمكننا أن نبدأ عادات جديدة وأكثر صحة عن طريق إضافة إشارات إيجابية والقضاء على الإشارات السلبية.

من المهم بناء إشارات طعام ونشاط إيجابية في روتيننا اليومي.

**الجزء الأول: التقدم والمراجعة الأسبوعية (10 دقائق)**

**وزع:** نشرات الجلسة 8 و متتبعات الطعام والنشاط للجلسة 6 مع ملاحظاتك.

**اجمع** متتبعات الطعام والنشاط الجلسة 7.

**ناقش** نجاحات وتحديات المشاركين في الأسبوع الماضي.

**حاضر:** تحدثنا الأسبوع الماضي عن موازنة السعرات الحرارية التي تتناولها من خلال تناول الطعام والشراب مع السعرات الحرارية التي تستخدمها أثناء النشاط البدني. يعمل الطعام والنشاط البدني معًا للتحكم في وزنك. لفقدان الوزن ، تناول سعرات حرارية أقل وكن أكثر نشاطًا.

**اسأل:** كم شخص منكم وصل إلى هدفه من النشاط البدني الأسبوع الماضي؟

**افتح المجال للرد**

**اسأل:** هل كنت قادرًا على اتخاذ خيارات نمط الحياة النشطة التي خططت لها؟ لما و لما لا؟

**افتح المجال للرد**

**اسأل:** إذا قررت خفض هدف السعرات الحرارية أو هدف غرام الدهون ، هل كنت قادرًا على القيام بذلك؟

**افتح المجال للرد**

**عالج** أي مشاكل حول ما طُلب من المشاركين القيام به خلال الأسبوع الماضي.

**حاضر**: هذا الأسبوع سوف:

- نرجع إلى الوراء نلقي نظرة على الأسباب التي تجعلنا نرغب في تناول الطعام وتجنب النشاط البدني. تسمى هذه المحفزات "الإشارات".
- نبحث عن طرق لتغيير المشكلة الغذائية وإشارات النشاط.
- نبحث عن طرق لإضافة إشارات إيجابية للنشاط والتخلص من إشارات عدم النشاط.

الجزء الثاني: إشارات الطعام (25 دقيقة)

تحديد الإشارات

**حاضر**: في هذه المرحلة من البرنامج ، نريد مساعدتك على فهم بعض الأفكار الأساسية حول السلوك. سنتحدث عن العوامل التي لها تأثير قوي على مقدار ما نأكله ، ونوعية ما نأكله ، والمدة التي نتحرك فيها.

يطلق عليها العلماء الذين يدرسون هذه العوامل "الإشارات". يمكن أن تكون الإشارات إيجابية أو سلبية. تعد الإشارات مشكلة عندما تجعلنا نتخذ خيارات غير صحية مثل الإفراط في تناول الطعام أو عدم النشاط البدني.

سنتحدث عن إشارات الطعام: العوامل التي تؤثر على مقدار ما نأكله وما نأكله ، وإشارات النشاط: العوامل التي تؤثر على مدى نشاطنا.

لن نتحدث فقط عن كيفية تأثير الإشارات علينا ، ولكن أيضًا ، والأهم من ذلك ، كيف يمكن لكل واحد منا التحكم في تلك الإشارات لتساعدنا على تحقيق أهدافنا بدلاً من منعناا.

الإشارات التي تجعلنا نأكل

**حاضر:** لنبدأ بإشارات الطعام.

**اسأل:** ما هي الإشارات التي تجعلك تأكل؟

**افتح المجال للرد**

**اطلب** من المشاركينالرجوع إلى منشور "ما الذي يجعلنا نأكل؟".

**قدم** هذه الاقتراحات:

- الجوع: بالطبع ، أحد أسباب تناولنا للطعام هو أننا جائعون.
- التفكير أو الشعور: قد تأكل لأنك تشعر بالوحدة أو الملل أو السعادة أو الحزن.
- بسبب أشخاص آخرون: قد تأكل شيئًا لأن شخصًا ما يعرض عليك أو يأكله أي شخص آخر. مثال: زيارة أشخاص آخرين ، حفلات الزفاف.
- رؤية أو رائحة الطعام: أحد أقوى الإشارات. مثال: ترى الآيس كريم على سناب شات وتريد بعضًا منه على الفور.
- بعض الأنشطة مثل مشاهدة التلفزيون أو الذهاب إلى المزرعة قد تجعلك تفكر في تناول الطعام.

**اسأل:** هل هناك من يرغب في تقديم مثال على اشارة طعام شخصي؟

**افتح المجال للرد**

**حاضر:** على سبيل المثال ، لماذا نأكل عندما نذهب إلى المركز التجاري؟

**افتح المجال للرد**

**حاضر:** هل تعتقد لأننا جائعون بالفعل؟ معظم الوقت ، لأنه عادة. عندما تستجيب لإشارة طعام بالطريقة نفسها مرارًا وتكرارًا ، فإنك تبني عادة. بمجرد أن تكون هذه العادة ، نستجيب لإشارة الطعام بطريقة تصبح أكثر تلقائية.

عندما يصبح تناول الطعام في المركز التجاري عادة ، من الصعب الذهاب إلى المركز التجاري وعدم تناول أي شيء.

**اسأل:** هل لدى أي شخص مثال لعادات الأكل التي قمت بتشكيلها و يصعب كسرها أو التخلي عنها الان ؟

**افتح المجال للرد**

اشارات المشاكل الشائعة في المنزل.

**حاضر**: تلميحاتأو اشارات الطعام ليست سيئة دائمًا. على سبيل المثال ، الجوع هو إشارة إيجابية. إذا لم يكن لدينا جوع ، فربما مات أسلافنا بسبب عدم الأكل. ومع ذلك ، فإن العديد من إشارات الطعام سلبية لأننا الآن في العصر الحديث نحتاج إلى تناول كمية أقل من الدهون والسعرات الحرارية أقل مما فعله أسلافنا.

**اسأل**: كيف تعتقد أنه يمكنك تغيير الإشارات والعادات الغذائيةالمكتسبة؟

**افتح المجال للرد**

**قم بإحالة** المشاركين إلى نشرة "تغيير عادات الطعام ".

**حاضر:** يمكنك تجربة هذه الأفكار:

- واحدة من أفضل الطرق هي الابتعاد عن مؤثرات الطعام أو إبقائه بعيدًا عن الأنظار.
- ابتعد عن صالة الطعام في المركز التجاري.
- أو يمكنك بناء عادة جديدة وأكثر صحة.
- يمكنك اختيار المطعم الصحي أو اختيار الطعام قبل الذهاب إلى المركز التجاري.

**حاضر:** تذكر أن التخلص من عادة قديمة أو بناء عادة جديدة يستغرق وقتًا. التغيير لا يحدث في يوم واحد.

من المهم أن تكون مدركًا للعديد من الأحداث والمواقف التي لها تأثير قوي على سلوكنا في الأكل والنشاط بطرق إيجابية وسلبية. فكر لمدة دقيقة حول العديد من مؤثرات الطعام الموجودة حولنا. في كثير من الأحيان لا ندرك مدى قوتها.

**اسأل:** ما هي بعض الأمثلة على إشارات الطعام من حولنا التي هي أقوى مما نعتقد؟

**افتح المجال للرد**

قدم هذه الاقتراحات:

- شاحنات الغذاء
- مواقع طلبيات الطعام كطلبات وجبلي
- البقالة
- تضع محلات السوبر ماركت منتجات جديدة على الرفوف هي الأسهل للرؤية وسهل الوصول إليها. إنهم يعرفون أنه كلما كان من الأسهل رؤية المنتج والتقاطه ، زاد احتمال شرائه.

كسر إشاراتك أو التخلي عنها

**حاضر:** دعنا نتحدث عن بعض إشارات مشكلتك ونناقش بعض الطرق التي يمكنك تغييرها.

سنبدأ من حيث تعيش. تصوروا أننا وصلنا للتو إلى منازلكم. دعونا نتصور ما هو موجود في أول غرفة ندخلها ، وبعد ذلك سنتحدث عما نراه. في اي غرفة انت الان؟ هل يوجد طعام في الغرفة؟ هل ترى أي شيء آخر قد يجعلك تفكر في تناول الطعام ، مثل كرسي مريح ، أو ذكرى حفلة؟

انتقل من غرفة إلى غرفة. هل توجد غرف أخرى بها إشارات تجعلك ترغب في تناول الطعام؟

**اسأل:** هل سيشارك شخص ما المشاكل التي واجهته أثناء رحلته حول المنزل؟ ما هو التغيير الذي يمكنك إجراؤه للابتعاد عن هذه الاشارات أو لبناء عادة جديدة وأكثر صحة؟

**افتح المجال للرد**

**ملاحظة:** اكتب كل مشكلة على السبورة واطلب من المجموعة إيجاد حلول.

إذا لم يستجب المشاركون ، اطلب منهم مراجعة "متعقب الطعام والنشاط" واسألهم عما إذا كان ذلك يساعدهم على التفكير في أي مشكلة.

**اعرض** هذه الاقتراحات إذا لم تكن مقترحة بالفعل.

- تلفزيون / كمبيوتر / جوال
- اجعلها قاعدة لا تأكلها أبدًا على الجهاز ؛ هذا يمكن أن يؤدي إلى الأكل والأكل بدون تفكير حتى عندما نكون ممتلئين.
- احتفظ بآلة تمرين بالقرب من التلفاز.
- اسمح لنفسك بتناول العلكة فقط أثناء وجودك على الجهاز.
- الحلويات
- لا تدخل الحلوى والحلويات للمنزل.
- إذا أحضرتهم إلى منزلك ، احتفظ بهم بعيدًا عن الأنظار.
- قم بشراء الكمية التي ستأكلها فقط في ذلك الوقت.
- اشترِ أحجامًا أصغر من الحلوى.
- الأطعمة عالية الدهون والسعرات الحرارية العالية في المطبخ
- توقف عن شراء هذه الأطعمة تمامًا.
- قم بتخزينها بعيدًا عن الأنظار ، في حاوية غير جذابة مثل حقيبة بنية أو صندوق غير مميز.
- اجعلهم يصعب الوصول إليهم.
- حافظ على سهولة الوصول إلى الطعام قليل الدسم وقليل السعرات الحرارية في الأفق وجاهز للأكل. (على سبيل المثال ، قطع الخضار في الثلاجة.)
- الأطعمة التي تطبخها أو تتناولها كبقايا الطعام
- اجعلها قاعدة أو عادة عدم تناول الطعام أثناء الطهي.
- عند الطهي ، تذوق الطعام مرة واحدة فقط للنكهة. ثم اغسل فمك بالماء.
- اطلب من شخص آخر تذوق الطعام.

* طاولة العشاء

- استخدم أطباق أو أوعية صغيرة.
- قدّم أجزاء صغيرة.
- تناول الطعام ببطء وامضغ جيدًا.

الإشارات الشائعة في العمل

**حاضر**: لقد نظرت إلى منزلك ووجدت بعض المشاكل الغذائية.

**اسأل:** لمن يعمل ، وماذا عن مكان عملك؟ هل هناك أي شيء في طريقك إلى العمل ، أو في العمل ، أو في طريقك إلى المنزل من العمل الذي يمكن أن يكون مشكلة غذائية؟

**افتح المجال للرد**

**قدم** هذه الاقتراحات إذا لم تكن قد تم اقتراحها بالفعل:

- مطعم وجبات سريعة ، شاحنة طعام
- خذ طريقًا مختلفًا للعمل.
- اجعلها قاعدة عدم تناول الطعام في السيارة.
- قم بإعداد غداء صحي أو وجبة فطور جاهزة للذهاب قبل مغادرة المنزل وعند عودتك.
- الأطعمة الغنية بالدهون والسعرات الحرارية العالية في الأماكن العامة (أخرج ، حلوى على مكتب زميل في العمل)
- تجنب تلك المناطق
- حاول إبقاء هذه الأطعمة بعيدة عن الأنظار.
- أحضر وجبات خفيفة قليلة الدسم وقليلة السعرات الحرارية لمشاركتها مع زملائك في العمل بدلاً من ذلك.
- التفاح والجزر النيء والبسكويت المملح والفشار قليل الدسم.
- آلات البيع
- ابتعد عن آلات البيع.
- إحضار وجبات خفيفة قليلة الدسم ومنخفضة السعرات الحرارية من المنزل.
- إذا كان يجب عليك الشراء ، فاختر خيارًا منخفض الدهون ومنخفض السعرات الحرارية.

**حاضر**: الأشياء المهمة التي يجب تذكرها هي:

1. احتفظ بالأطعمة التي تحتوي على نسبة عالية من الدهون والسعرات الحرارية خارج منزلك ومكان عملك ، أو ابقائها بعيدًا عن الأنظار.
2. حافظ على سهولة الوصول إلى الأطعمة قليلة الدسم وقليلة السعرات الحرارية وجاهزة للأكل.
   - فواكه طازجة ، خضار نيئة ، مغسولة ومقطعة ، فشار قليل الدسم ، ماء.
3. قللي من تناول الطعام في مكان واحد.
   - لا تأكل أمام التلفاز.
4. عندما تأكل ، قم بالحد من الأنشطة الأخرى.
   - تناول وجبتك دون أن تكون على الجهاز والقيادة.
   - ركز على الاستمتاع بالوجبة.

مشاكل التسوق الشائعة

**حاضر:** أخيرًا ، دعنا نلقي نظرة على المكان الذي تتسوق فيه عادة.

**اسأل:** من المسؤول في الأسرة عن التسوق للطعام؟

**حاضر**: تصور المشي حول المتجر أو السوق كما تفعل عادة.

**اسأل**: ما الذي تراه يمثل مشكلة لك أو لعائلتك؟

**افتح المجال للرد**

**قم بإحالة** المشاركين إلى نشرة "نصائح التسوق".

**حاضر:** ليس لديك القدرة على التحكم في الأطعمة الموجودة في محل البقالة كما تفعل في منزلك ، ولكن هناك بعض الأشياء التي يمكنك القيام بها.

هذه بعض الاقتراحات:

- اكتب قائم التسوق في وقت مبكر. اجعلها قاعدة لا تشتري أي شيء غير موجود في القائمة.
- لا تذهب للتسوق عندما تشعر بالجوع. تناول وجبة خفيفة قليلة الدسم أو منخفضة السعرات الحرارية أولاً.
- تجنب أقسام المتجر التي تجذبك إن أمكن. تجنب أقسام المخبوزات أو الآيس كريم / الحلوى ، على سبيل المثال.

**اسأل**: هل هناك أي مشاكل غذائية أخرى تود مناقشتها؟

**افتح المجال للرد**

تقديم: الآن ، لنلق نظرة على أنواع الإشارات التي يمكن أن تجعلنا غير نشطين.

الجزء 3: إشارات النشاط (15 دقيقة)

حاضر: العديد من الإشارات في بيئتنا يمكن أن تؤدي إلى عدم النشاط.

على سبيل المثال ، بعد العشاء يمكنك الجلوس تلقائيًا أمام التلفاز. هذا لأنك ربطت مشاهدة التلفزيون بعد تناول العشاء عدة مرات في الماضي. تذكر ، مع ذلك ، أنه لديك خيار. يمكنك تغيير هذا النمط واختيار المشي بعد العشاء بدلاً من مشاهدة التلفزيون أو مشاهدة مقاطع الفيديو على هاتفك المحمول.

إذا كنت غير نشط لفترة من الوقت ، فمن المحتمل أن يكون لديك العديد من الإشارات التي تؤدي إلى عدم النشاط وقليل من الإشارات التي تؤدي إلى النشاط.

لكي تصبح نشطًا بانتظام ، يجب عليك إضافة إشارات نشاط إيجابية إلى حياتك. بمرور الوقت ، ستؤدي الإشارات إلى عادات جديدة والمزيد من النشاط. سوف يصبح الأمر أسهل بمرور الوقت حيث تصبح عاداتك أكثر تلقائية.

**اسأل:** ما هي بعض إشارات النشاط الإيجابي التي يمكنك الحصول عليها؟

لنبدأ بتصور المكان الذي تعيش فيه مرة أخرى.

**اسأل**: ما الذي يمكن أن تضيفه إلى غرفة المعيشة التي ستحثك على أن تكون أكثر نشاطًا؟ ماذا عن غرفة النوم؟ في المطبخ؟

**قم بإحالة** المشاركين إلى نشرة "إشارات النشاط".

**قدم** هذه الأمثلة من الإشارات الإيجابية:

- في غرفة المعيشة أو غرفة النوم:
- ضع أحذية ومعدات التمرين في مكان واضح (ليس في الخزانة).
- قم بتعليق تقويم نشاط ولاحظ عندما كنت نشطًا في ذلك اليوم.
- احتفظ بدراجة ثابتة أو بساط تمرين أمام التلفاز.
- يمكنك تعليق صورة لمشهد خارجي أو الأشخاص النشطين.
- ضع ملاحظة على التليفزيون لتذكيرك بأن نصف ساعة من وقت مشاهدة التلفاز يمكن استخدامها لممارسة الرياضة بدلاً من ذلك.
- في المطبخ:
- علق الملاحظات لتكون نشطا.
- استخدم هاتفك المحمول لجدولة الوقت لتكون نشطًا.

إزالة إشارات الخمول

**اسأل:** ما هي بعض الإشارات الإيجابية التي يمكنك إضافتها إلى مكان عملك؟

افتح المجال للرد

- بدلًا من أن تطلب من مساعدك أن يحصل على شيء لك ، اذهب بنفسك.
- ضع ملاحظة على باب مكتبك أو جهاز الكمبيوتر الخاص بك للتنزه.
- اضبط منبهًا على هاتفك أو جهاز الكمبيوتر لتذكيرك بالتنقل كل ساعة.

**حاضر:** هناك إشارات لمساعدتك على أن تكون نشطًا ولا تعتمد على محيطك.

يمكنك تحديد موعد منتظم للنشاط مع أحد أفراد العائلة. يمكن أن يساعدك الالتزام مع الآخرين في أن تكون نشطًا حتى عندما لا تشعر بالدوافع أو الطاقة. يمكنك تذكير بعضكما البعض "مرحبًا ، حان وقت المشي!"

**اسأل:** هل يمكنك التفكير في أي إشارات نشاط أخرى لم نناقشها؟

**افتح المجال للرد**

باختصار ، يستغرق الأمر وقتًا لكسر العادات القديمة وبناء عادات جديدة وأكثر صحة. ولكن يمكن القيام بذلك ، ويمكنك القيام بذلك! أهم الخطوات هي العثور على إشارات مشكلتك والقضاء عليها ، أو حتى الأفضل ، استبدالها بأخرى جديدة وأكثر صحة.

يمكنك جعل إشارات الطعام والنشاط مناسبة لك ، وليس ضدك.

الجزء الرابع: ختام وقائمة المهام (10 دقائق)

**اسأل** عما إذا كانت هناك أي أسئلة حول ما تمت تغطيته خلال هذه الجلسة.

**حاضر: إ**ليك ما أريدك أن تفعله في الأسبوع المقبل: فلنضع خطة لكل واحد منا لإزالة تلميح أو اشارة سلبية واحدة للطعام من حياتنا.

**قم بإحالة** المشاركين إلى نشرة "المهام الأسبوع المقبل".

فكر في مشكلة واحدة للتخلص منها قبل الأسبوع المقبل. فكر في أي مشاكل يمكنك رؤيتها بالتخلص منها ، وكيف ستتغلب عليها.

اكتب إشارة المشكلة وماذا ستفعل لإزالتها.

**اضافي:** فكر في إشارة إيجابية واحدة وهي كونك أكثر نشاطًا جسديًا التي يمكنك إضافته. تذكر توازن السعرات الحرارية. إذا تمكنا من تحسين الداخل من السعرات الحرارية والخارج منها ، فسوف نرى نتائج أفضل. اكتب تلميحك الإيجابي لكونك أكثر نشاطًا ، ومرة ​​أخرى ، فكر في المشكلات التي قد تواجهها في إضافة هذا ، وكيف ستحلها.

**للأسبوع القادم:**

- استمر في البحث عن طرق للنشاط البدني. معظمكم قام ببناء ما يصل إلى 150 دقيقة أسبوعيًا للنشاط البدني.
- تذكر ، يمكن تقسيم الوقت إلى أجزاء أصغر مثل 20 أو حتى 10 دقائق في المرة الواحدة.
- تتبع وزنك وتناول الطعام والنشاط. ابذل قصارى جهدك للوصول إلى أهدافك!

**تلخيص النقاط الرئيسية:**

- **نظرنا في أنواع الطعام والنشاط التي تجعلنا نتصرف بطرق غير صحية.**
- **تحدثنا عن كيفية تغيير تلك الإشارات ، من خلال تجنب الاشارات التي تقف في طريق أهدافنا واستبدالها بإشارات إيجابية.**
- **عملنا على خطة لكيفية البدء في إزالة إشارات الطعام والنشاط السلبية.**

**النهاية:** لقد نجحت في النصف الأول من هذا البرنامج! ثابر على العمل الجيد!

تسمى جلستنا التالية "حل المشكلات". سنتحدث عن خمس خطوات يمكننا استخدامها لحل المشاكل التي نواجهها.

**اسأل** المشاركين عما إذا كان لديهم أي أسئلة قبل إغلاق الجلسة.

**بعد الجلسة:**

قم بمراجعة وكتابة ملاحظات حول النجاحات والتوصيات الخاصة بتتبع الطعام والأنشطة من الجلسة 7.
