# Supplementary material for: Emirates Heart Health Project (EHHP): A protocol for a stepped-wedge family-cluster randomized-controlled trial of a health-coach guided diet and exercise intervention to reduce weight and cardiovascular risk in overweight and obese UAE nationals
Source: PLoS One. 2023 Apr 10;18(4):e0282502. doi: 10.1371/journal.pone.0282502 (PMC10085020; doi:10.1371/journal.pone.0282502)
Supplement: S22 Appendix — (DOCX) [file pone.0282502.s022.docx]

**الجلسة 9: حل المشكلات**

**أهداف التعلم**

في ختام هذه الجلسة سيتمكن المشاركون من:

- سرد ووصف 5 خطوات لحل المشكلة
- تطبيق الخطوات الخمس لحل المشكلات التي يعاني منها أي من المشتركين كتناول كمية أقل من الدهون والسعرات الحرارية أو زيادة النشاط.

**المواد:**

- نشرات المشاركين للجلسة 9:
  - نظرة عامة
  - الخطوة 1: وصف المشكلة بالتفصيل
  - الخطوة 2: قم بعصف ذهني لخياراتك
  - الخطوة 3: حدد خيارًا واحدًا للمحاولة
  - الخطوة 4: وضع "خطة عمل إيجابية"
  - الخطوة 5: جربها!
  - التدريب عل حل المشكلات
  - مهام الأسبوع المقبل
- "متتبعو الطعام والنشاط" للجلسة 9
- علامات الأسماء
- لوحة بيضاء وأقلام

**نظرة عامة**

تواصل الجلسة 9 السعي لفهم كيفية إنشاء العادات الصحية والحفاظ عليها. ـتمنح المشاركين أداة يحتاجون إليها لفهم ومعالجة العوائق التي يواجهونها أثناء عملهم نحو أهدافهم المتمثلة في الأكل الصحي والنشاط البدني المنتظم.

تنقسم الجلسة 9 إلى 3 أجزاء:

الجزء الأول: التقدم والمراجعة الأسبوعية (10 دقائق)

الجزء 2: 5 خطوات لحل المشكلات (40 دقيقة)

نعلم خطوات حل المشكلات باستخدام مثال "يوم في حياة سارة". تنتهي سارة ، بسبب العديد من العوامل والأحداث ، تناول جزء كبير من الكوكيز. سنستخدم هذا المثال للتعلم ومناقشة وتطبيق 5 خطوات لحل المشكلات:

1. صف المشكلة بالتفصيل.

2. خيارات العصف الذهني.

3. حدد خيارًا واحدًا للمحاولة.

4. ضع خطة عمل إيجابية للخيار المختار.

5. جرب الخطة.

بعد ذلك ، ستتحول المناقشة إلى مشاكل المتطوعين ، وسيتم استخدام الخطوات الخمس للتدرب على حل المشكلة. سيختار كل مشارك بعد ذلك مشكلة واحدة مع الأكل الصحي أو النشاط البدني ويطبق الخطوات الخمس على مشكلته في الأسبوع المقبل.

.

**الرسائل الرئيسية**

حل المشكلات هو عملية . لا تستسلم عندما لا يمكنك التوصل إلى حل على الفور. ولا تستسلم عندما لا تنجح خطتك الأولى لحل المشكلة. غالبًا ما تحتاج الى العديد من المحاولات لإيجاد حل.

المشاكل حتمية ، ولكن يمكن حل معظم المشاكل المتعلقة بتناول القليل من الطعام وزيادة النشاط.

إن اتباع إجراء لحل المشكلات يساعدنا على رؤية أننا لسنا عاجزين عن تغيير وضعنا.

**الجزء الأول: التقدم والمراجعة الأسبوعية (10 دقائق)**

**وزع** نشرات:

- نشرات الجلسة 9.
- الجلسة 9 "متتبعو الطعام والنشاط".
- الجلسة 7 "متتبعو الطعام والنشاط" مع ملاحظاتك.
- اجمع الجلسة 8 "متتبعو الطعام والنشاط".

**اسأل**: هل يحتاج أي شخص للمساعدة في تسجيل نشاطه على الرسم البياني؟

ساعد المشاركين عند الحاجة.

**حاضر:** درسنا في الأسبوع الماضي الإشارات التي تجعلنا نأكل ونكون غير نشطين. بحثنا عن طرق لاستبدال الإشارات السلبية بإشارات إيجابية. لقد توصلت إلى خطة لاستبدال الإشارات السلبية الشخصية بأخرى إيجابية.

**اسأل:** كيف كان النشاط؟ ماذا عن إشارات النشاط البدني؟ هل كانت هناك مشاكل؟ هل يريد أي متطوعين مشاركة تجاربهم الأسبوع الماضي؟

**ردود مفتوحة**

**حاضر**: ما الذي يمكنك فعله بشكل مختلف الأسبوع المقبل؟

**ردود مفتوحة**

**عالج** أي مشاكل أو ارتباك حول ما كان من المفترض أن يفعلوه الأسبوع الماضي.

**حاضر:** هذا الأسبوع سوف:

نتحدث عن 5 خطوات لحل المشكلات لمساعدتك في الوصول إلى أهداف الأكل الصحي والنشاط البدني.

نمنحك الفرصة لتطبيق هذه الخطوات الخمس على المشاكل التي تعرقل تحقيق أهدافك.

الجزء 2: 5 خطوات لحل المشكلات (40 دقيقة)

**حاضر**: في الجلسات الثماني الماضية ، تعلمت الكثير عن كيفية تناول الطعام الصحي والنشاط البدني. إذا تم ذلك باستمرار ، ستساعدك هذه الطرق على منع أو تأخير أمراض القلب ، أو إذا كنت تعاني بالفعل من مشاكل في القلب ، تقلل من فرصة الإصابة بمزيد من مشاكل القلب. قد تساعدك أيضًا على إنقاص الوزن والحفاظ على الصحة بشكل عام.

قد يختلف إجراء هذه التغييرات خلال فترة زمنية قصيرة ، مع الكثير من الدعم الأسري ، عن جعل هذه التغييرات دائمة. يمكن أن تقف العديد من العوامل في طريق التغيير طويل الأمد.

لذلك ، في الجلسات العديدة القادمة ، سنتحدث عن كيفية جعل التغييرات الإيجابية جزءًا دائمًا من حياتك. سنناقش:

- الأفكار السلبية وكيفية التغلب عليها.
- الزلات أو الاخطاء (عندما لا تتبع خطة الأكل الصحي / النشاط البدني بشكل مؤقت) ورد فعلك تجاه الزلات.
- الضغط العصبي\ النفسيى.
- إشارات اجتماعية (ما يقوله الآخرون ويفعلونه).
- كل هذا يمكن أن يؤثر على تناول الطعام الصحي والنشاط البدني.

**اسأل:** ما هي بعض العوامل التي تقف في طريق تغيير العادات غير الصحية؟

**ردود مفتوحة**

**ملاحظة:** اذكر بعض المشكلات التي تمت مناقشتها في جلسات سابقة. فيما يلي بعض الأمثلة المحتملة:

- أردت الذهاب في نزهة على الأقدام ، لكن الجو كان ساخن / بارد جدًا.
- أردت تناول كمية أقل من الدهون ، لكن عائلتك أرادت شراء البطاطس المقلية.
- أنت متردد في المشي عندما يحل الظلام.

**حاضر**: لا مفر من حدوث المشاكل ، ولكن عادة ما يمكن حلها. سنتحدث اليوم عن عملية لحل المشكلات ، وسنستمر في العمل على مهارات حل المشكلات لبقية البرنامج.

الخطوة 1: وصف المشكلة.

**حاضر**: عملية حل المشكلة هذه لها 5 خطوات:

1. وصف المشكلة.

2. عصف ذهني الخيارات المتاحة أمامك لحل المشكلة.

3. حدد خيارًا واحدًا للمحاولة.

4. ضع خطة عمل إيجابية لتطبيق الخيار الذي تم اختياره.

5. جربها!

**ارجع** إلى نشرة "الخطوة 1: وصف المشكلة بالتفصيل".

**حاضر**: سيكون لديك نشرة منفصلة اليوم لكل خطوة من خطوات العملية.

الخطوة 1: وصف المشكلة.

أولاً ، صف المشكلة بالتفصيل. كن دقيقا. على سبيل المثال ، إذا كنت تتناول الأطعمة التي تحتوي على نسبة عالية من الدهون والسعرات الحرارية ، صفها

- أنواع الأطعمة التي تتناولها والتي تحتوي على نسبة عالية من الدهون والسعرات الحرارية. (معجنات ، لحم أحمر)
- الحالات التي تتناول فيها تلك الأطعمة. (في حفلات الزفاف والأطراف الأخرى)
- الظروف المرتبطة بتناول تلك الأطعمة ، بما في ذلك الأفكار والمشاعر والسلوكيات. (أنا أيضا أكل هذه الأطعمة عندما أشعر بالحزن أو الوحدة)

مثال آخر هو ، بدلاً من القول ، "أنا آكل الكثير من الدهون" ، قل ، "أنا آكل الحلويات الغنية بالدهون عندما أذهب إلى منزل حماتي. تتأذى مشاعرها إذا لم أتناول الكوكيز( بسكويت) الشهيرة التي صنعتها ، ثم ينزعج زوجي لأنني أزعج والدته.

تسمى هذه السلسلة من الأحداث والأفكار "سلسلة العمل" أو "سلسلة السلوك". دعونا نلقي نظرة على كيفية ارتباط العوامل. في هذا المثال ، يتضمن عوامل مثل:

- إشارات الطعام أو النشاط (كما ناقشنا في الجلسة الأخيرة): المواقف التي تجعلنا نأكل طعامًا غير صحي أو الكثير من الطعام أو نكون غير نشطين. في هذه الحالة ، أحد الإشارات هو زيارة منزل حماة. بمرور الوقت ، شكلت الاستجابات لهذا التلميح عادة.
- الأصدقاء والأقارب الذين لا يدعمون جهودنا لفقدان الوزن وأن يكونوا أكثر نشاطًا.
- حماة غير داعمة.
- زوج غير داعم.
- الأفكار أو المشاعر التي تقف في طريق الهدف.

"لا أستطيع أن أدافع عن نفسي ، لذلك سوف أتناول الحلويات فقط."

ربما تؤدي الزيارات إلى شعور هذا الشخص بالملل أو الغضب أو الوحدة أو التوتر.

**حاضر:** قد يبدو الأمر معقدًا للنظر في مشكلة بهذه الطريقة. ومع ذلك ، فإن تقسيم سلسلة الإجراءات إلى الأحداث الصغيرة التي أدت إلى هذا السلوك يجعل المشكلة التي نحاول حلها أصغر وأسهل لحلها. كما هو الحال مع الطرق الأخرى ، تتطلب هذه الطريقة ممارسة.

اسمحوا لي ان اقول لكم قصة. سارة امرأة مشغولة تعمل وتعتني بأسرتها. بالأمس ، كانت مشغولة للغاية في العمل ولم تتناول الإفطار أو الغداء لأنها لم يكن لديها الوقت. في فترة ما بعد الظهر ، كان رئيسها ينتقد ويطالب بمشروع كبير في العمل ، وشعرت سارة بالتوتر والقلق.

في نهاية اليوم ، عادت سارة إلى المنزل وهي متعبة ومنزعجة وجائعة.

**اسأل:** ماذا تعتقد أن سارة فعلت؟

**ردود مفتوحة**

**استمر:** دخلت المطبخ. لم يكن الطعام جاهزًا بعد ، لكنها رأت على الفور كيسًا من الكوكيز على المنضدة. قبل أن تعرف ذلك ، كانت قد تناولت نصف الكيس.

دعونا نلقي نظرة على يوم سارة وما أدى إلى سلوك تناول الكثير من الكوكيز - سلسلة الحركة. عندما ننظر إلى هذه السلسلة ، سنرى أن مشكلة تناول الكثير من الكوكيز كان لها أكثر من سبب واحد وكانت أكثر تعقيدًا من مجرد "الافتقار إلى قوة الإرادة أو ضبط النفس".

نعم. أكلت سارة نصف كيس الكوكيز . هذا ما نسميه "زلة". عندما يحدث هذا ، لا يمكننا التراجع عنه ، ولكن يمكننا التعامل مع سلسلة الحركة لمنعها من الإفراط في تناول الطعام مرة أخرى.

سيساعدنا النظر في سلسلة الإجراءات في العثور على "الروابط الضعيفة" حيث يمكننا كسر السلسلة حتى لا تؤدي العوامل إلى السلوك الذي نحاول منعه.

الخطوة 2: العصف الذهني خياراتك.

**حاضر:** كانت الخطوة 1 لوصف المشكلة وسلسلة الإجراءات التي أدت إلى ذلك بالتفصيل. في قصتنا ، تخطيت سارة الإفطار والغداء ، وضغطت في العمل ، وعادت إلى المنزل جائعة وأكلت نصف كيس من الكوكيز. دعنا ننتقل إلى الخطوة 2.

**قم** بإحالة المشاركين إلى نشرة "الخطوة 2: قم بعصف ذهني لخياراتك".

**اسأل:** ما هي الأشياء التي يمكن أن تفعلها سارة بشكل مختلف لتجنب تناول الكوكيز في اللحظات التي تصل فيها إلى المنزل؟

**ردود مفتوحة**. دع الأفكار تتدفق. كلما زادت الأفكار ، مهما كانت شديدة أو سخيفة ، كان ذلك أفضل.

قدم هذه الأفكار (إذا لم تعرضها المجموعة):

- يمكنها اختيار عدم شراء الكوكيز في المقام الأول.
- يمكنها التحدث مع زميلة في العمل أو زميل لها حول ضغوط وظيفتها.
- يمكنها إحضار وجبة خفيفة صحية للعمل ، حتى تتمكن من تناول شيء ما قبل المغادرة.
- يمكنها الذهاب في نزهة للاسترخاء.
- يمكنها القيام ببعض الأنشطة التي تستمتع بها لتقليل التوتر.
- يمكنها تغيير الوظائف.
- يمكنها أن تبدأ مشروعها الخاص ، حيث تكون هي الرئيسة!

**حاضر:** من خلال طرح الكثير من الأفكار ، يمكنك أن ترى أننا لسنا عاجزين عن تغيير مواقفنا. انظر إلى عدد الخيارات التي توصلنا إليها.

الخطوة 3: حدد خيارًا واحدًا للمحاولة

**تقديم**: بعد أن توصلنا إلى جميع الخيارات الممكنة ، حان الوقت للخطوة 3: حدد خيارًا لتجربته.

**قم** بإحالة المشاركين إلى نشرة "الخطوة 3: حدد خيارًا واحدًا للمحاولة".

**قم** بموازنة الفوائد والمشكلات مع كل خيار واختر واحدًا أو مجموعة من عدة خيارات للتجربة.

**تأكد** من اختيار الشخص الذي تعتقد أنه لديه أفضل فرصة للعمل ، وأنك قادر على القيام بذلك ، وأنك على استعداد للقيام بذلك. وبعبارة أخرى ، كن واقعياً. يجب أن تكون واثقًا بشكل معقول من أنك ستنجح.

من المفيد أيضًا كسر أكبر عدد ممكن من الروابط في سلسلة الإجراءات في أقرب وقت ممكن.

على سبيل المثال ، لنفترض أن سارة اختارت خيار إحضار وجبة خفيفة صحية للعمل حتى تتمكن من الحصول عليها إذا لم يكن لديها فرصة لتناول وجبة الإفطار. بهذه الطريقة لا تصل إلى المنزل جائعة للغاية. قد ينجح هذا حتى لو اشترت أختها الكوكيز وتركتها في المنزل.

أو قد تختار سارة عدم شراء الكوكيز ، لذلك عندما تصل إلى المنزل جائعة ، عليها أن تبحث عن شيء آخر ، نأمل أن يكون أكثر صحة من الكوكيز.

الخيار الثالث ، إذا استطاعت ، هو عدم شراء الكوكيز وإحضار وجبة خفيفة للعمل في الأيام التي لا تحصل فيها على فرصة لتناول الإفطار. باختيار الخيارين ، تزيد من فرص نجاحها. حتى لو كانت جائعة عندما تعود إلى المنزل ، فإن هذه الوجبة الخفيفة قد ساعدت ، لذلك لن تكون جائعة تمامًا ومن المحتمل أن تأكل. ثم عندما تعود إلى المنزل ، لا توجد الكوكيز في المنزل ، لذلك تضطر إلى تناول شيء آخر. نأمل أن يكون هناك بعض الفاكهة أو خيار صحي آخر لتناول الطعام.

الخطوة 4: ضع خطة عمل إيجابية

**قم** بإحالة المشاركين إلى نشرة "الخطوة 4: وضع خطة عمل إيجابية".

**حاضر**: الخطوة الرابعة هي وضع خطة عمل مفصلة للخيار أو الخيارات التي حددتها. اكتب بالضبط:

- ماذا ستفعل.
- متى ستفعلها
- ما عليك القيام به أولا.
- كيف ستتعامل مع أي تحديات تظهر.
- ضع في اعتبارك ما يلي لزيادة احتمالية النجاح:
- أشرك شخصًا آخر.
- اجعل خطتك ممتعة وممتعة.
- اكتب خطتك وضعها في مكان ما حيث ستراها.

**حاضر:** لنفترض أن سارة اختارت خيار إحضار وجبة خفيفة صحية للعمل. في خطة عملها ، قررت سارة شراء بعض المكسرات و الجرانولا والاحتفاظ بها في مكتبها في حال شعرت بالجوع.

وهي تدرك أن زميلتها في المكتب حمدة لديها نفس المشكلة وقد "تشارك" بعض وجباتها الخفيفة إذا تركتها في درج مكتبها.

في حالة حدوث ذلك ، واختفت المكسرات وجرانولا وسارة جائعة للغاية ، توصلت إلى خطة بديلة: ستتوقف عند لولو في طريقها إلى المنزل (وليس هارديز) وستشتري شطيرة جاهزة أكثر صحة من الكوكيز .

لذا ، اكتشفت سارة طريقة لكسر حلقة ضعيفة في سلسلة العمل ، ولديها خطة أخرى في حالة ظهور بعض التحديات.

الخطوة 5: جربها!

**حاضر**: ما هي الخطوة الخامسة والأخيرة؟

**قم** بإحالة المشاركين إلى منشور "الخطوة 5: جربها!"

**حاضر: ب**مجرد وصف المشكلة وسلسلة الإجراءات الخاصة بها ، وخيارات العصف الذهني ، واختيار الخيار والخروج بخطة عمل إيجابية ، حان الوقت لتجربتها. نرى كيف ستسير الامور. إذا كان يعمل ، عظيم! إذا لم يكن الأمر كذلك ، فيمكننا استخدام ما تعلمناه من تجربتنا لوضع خطة عمل جديدة.

تذكر أن حل المشكلات هو عملية. لا تستسلم. غالبًا ما يتطلب النجاح عدة محاولات.

**حاضر**: فلنراجع. هل يمكنك إخباري بالخطوات الخمس لحل المشكلات؟

ردود مفتوحة

مراجعة:

1. صف المشكلة.

2. عصف ذهني الخيارات المتاحة أمامك لحل المشكلة.

3. حدد خيارًا واحدًا للمحاولة.

4. ضع خطة عمل إيجابية لوضع الخيار الذي تم اختياره موضع التنفيذ.

5. جربها.

ممارسة حل المشكلات

**حاضر: ا**لآن ، دعنا نطبق هذه العملية على حياتك الخاصة. أريد أن يفكر كل منكما في مشكلة تتعلق بالأكل الصحي أو النشاط البدني يمكنك حلها باستخدام هذه الخطوات الخمس.

**إحالة** المشاركين إلى نشرة "ممارسة حل المشكلات".

**حاضر:** نفذ الخطوات من 1 إلى 4 باستخدام نشرة "ممارسة حل المشكلات".

1. صف المشكلة بالتفصيل. قسّمها إلى أقسام أو أجزاء. كيف ترتبط هذه الأجزاء؟

2. العصف الذهني خياراتك.

3. حدد خيارًا. اسأل نفسك ، "هل من المحتمل أن تعمل؟" و "هل يمكنني القيام بذلك؟"

4. ضع خطة عمل إيجابية للخيار الذي اخترته.

أ. ماذا ستفعل؟

ب. متى وكيف ستبدأ؟

ج. ما هي حواجز الطرق التي قد تظهر ، وكيف ستتعامل معها؟

د. ماذا ستفعل لجعل النجاح محتمل؟

5. ثم ، ستحاول!

اطلب المساعدة أيضًا إذا كنت بحاجة إليها. أنا هنا لمساعدتك ، كما هو الحال مع بقية المجموعة.

**اسأل**: هل سيشارك شخص ما مشكلته وكيف عمل في الخطوات الخمس؟

**ردود مفتوحة.** العمل من خلال مشكلة واحدة على الأقل كمجموعة.

الجزء 3: ختام وقائمة المهام (10 دقائق)

**اسأل**: هل لدى أي شخص أي أسئلة حول ما تناولناه في هذه الجلسة؟

**حاضر**: في الأسبوع المقبل ، تدرب على استخدام الخطوات الخمس لحل مشكلة لديك تتعلق بالوصول إلى أهدافك الغذائية ونشاطك.

تتبع وزنك وتناول الطعام والنشاط البدني. ابذل قصارى جهدك للوصول إلى أهدافك.

جربها! نفذ الخطوة 5 من خطة العمل التي أنشأتها لنفسك في النشرة ، وفي الأسبوع المقبل أخبرنا كيف سارت الأمور.

قم بتجميع القوائم من المطاعم المفضلة لديك ، وأحضرها إلى الجلسة التالية. سنستخدمها خلال المناقشات المستقبلية.

**اسأل:** ما المطاعم التي تفضلها؟ (اكتب هذه الأشياء وحاول الحصول على القوائم بنفسك.)

**اسأل** عما إذا كان لدى المشاركين أي أسئلة حول ما تمت تغطيته في هذه الجلسة.

تلخيص النقاط الرئيسية:

سوف تواجه دائما مشاكل. المشاكل جزء طبيعي من الحياة.

سيساعدك استخدام عملية حل المشكلات التي تعلمناها اليوم في العثور على أفضل حل.

تذكر الخطوات الخمس عندما تواجه مشكلة:

1. صف المشكلة.

2. العصف الذهني خياراتك للحلول.

3. حدد خيارًا واحدًا للمحاولة. تأكد من أنها واقعية.

4. ضع خطة عمل لتطبيق الخيار الذي تم اختياره.

5. جربها.

قد لا تجد حلاً في محاولتك الأولى ، ولكن لا تستسلم. غالبًا ما يستغرق الأمر عدة محاولات للعثور على حل ناجح.

إغلاق: لقد أعطتك هذه الجلسة أداة للتعامل مع المشاكل التي ستواجهها أثناء عملك نحو هدفك. سوف نركز في الجلسة القادمة على تحدٍ حقيقي عندما نحاول تناول طعام صحي: تناول الطعام في الخارج.

تذكر إحضار بعض القوائم حتى نتمكن من العمل في الأماكن التي ترغب في تناول الطعام فيها.

تغطي الجلسة 10 طرقًا للتأكد من استمرار الأكل الصحي حتى عندما لا تكون في المنزل.

**اسأل** عما إذا كانت هناك أي أسئلة أو استفسارات.

**عالج** أي أسئلة أو استفسارات.

بعد الجلسة:

قم بتدوين ملاحظاتك وتوصياتك المعتادة في "متتبعي الطعام والنشاط" من الجلسة 8.
