# Supplementary material for: Emirates Heart Health Project (EHHP): A protocol for a stepped-wedge family-cluster randomized-controlled trial of a health-coach guided diet and exercise intervention to reduce weight and cardiovascular risk in overweight and obese UAE nationals
Source: PLoS One. 2023 Apr 10;18(4):e0282502. doi: 10.1371/journal.pone.0282502 (PMC10085020; doi:10.1371/journal.pone.0282502)
Supplement: S23 Appendix — (DOCX) [file pone.0282502.s023.docx]

**Session 10: 4 keys to healthy eating out**

**Learning objectives**

At the close of this session, the participants will be able to:

- List and describe the 4 keys for healthy eating out.
- Give examples of how to apply these keys at the restaurants that the participants go to regularly.
- Make an appropriate meal selection from a restaurant menu.
- Demonstrate how to ask for a substitute item using assertive language and a polite tone of voice.

**Materials**

- Participant handouts for session 10:
  - Session 10 overview
  - 4 keys to healthy eating out
  - Plan ahead
  - Ask for what you want
  - Take charge of what’s around you
  - Choose your food carefully
  - What’s on the menu?
  - Fast food can be low in fat
  - A positive action plan
  - To do next week
- Food and Activity Tracker for Session 10
- Name tags
- White board and markers
- Sample menus from favorite restaurants.
  - One for discussion, with enough copies for each participant.
  - One for the practice activity, with enough copies for each participant.

**Session overview**

Session 10 continues the process of helping participants to take control of their surroundings by giving them tools to make healthy choices when eating out. Eating out in a healthy way requires participants to plan ahead, stay focused and be assertive in asking for what they want.

Session 10 has 4 parts:

*Part 1: Weekly progress and review (5 minutes)*

*Part 2: Keys to healthy eating out (25 minutes)*

You will talk about eating out in different situations (visits to family, restaurants, parties) and use 4 keys to healthy eating out:

1. Plan ahead.
2. Ask for what you want.
3. Take charge of what’s around you.
4. Choose foods carefully.

*Part 3: Practice getting what you want (25 minutes)*

Participants practice the 4 keys by role playing with you as the server, using real menus and the handouts.

*Part 4: Wrap up and to-do list (5 minutes)*

**Key messages:**

- **Eating out** (whether at someone’s home, at a restaurant, at a party, in an airplane) **is a common experience for many people, but doing so can pose many challenges to healthy eating.**
- **Eating out and eating healthily CAN BE DONE. However, doing so requires planning, effective communication, and careful food selection.**

*Part 1: Weekly progress and review (5 minutes)*

**Distribute:**

- Session 10 handouts
- Session 10 “Food and Activity Trackers”
- Session 8 “Food and Activity Trackers” with your notes and recommendations.

**Collect** Session 9 “Food and Activity Trackers”.

**Discuss** participants’ success and difficulties in meeting their weight loss goals.

**Ask:** What went well and not so well in keeping track of your food and physical activity last week? Were you able to stay within your fat gram and calorie budget? Were you able to reach physical activity goal?

**Open responses.**

**Present:** Last week, we learned about 5 steps to problem solving and practiced using them to solve problems that get in the way of reaching our goals.

**Ask:** Did you try your action plan? What did you learn from the problem solving process?

**Open responses.**

**Address** any issues or confusion about what they were asked to do last week. Remember to praise all progress, no matter how small. Discuss barriers, and problem solve with participants to overcome the barriers.

**Present:** This week we will:

- Discuss the 4 keys to healthy eating out: 1) Plan ahead, 2) Ask for what you want, 3) Take charge of what’s around you, and 4) choose healthy foods.
- See examples of how to use these 4 keys at the places where you eat out (restaurants, parties, other people’s houses.
- Talk about how to make the healthiest meal selections from a restaurant menu.
- Learn how to ask confidently for an item substitution, using appropriate language and tone of voice.

*Part 2: Keys to healthy eating out (25 minutes)*

**Present:** Today we will talk about eating outside of your house. IT is something we all do that is especially challenging when we are trying to make healthy lifestyle changes.

**Ask:** Where do you usually eat when you eat outside of the house? Any favorite restaurants? Did you bring menus to share?

Note: If participants brought menus, choose 1 or 2 of them to use as examples for the rest of the session. If they did not bring menus, ask them for a few examples: fast food restaurants, friends’ or family members’ houses…

**Open responses.**

**Ask:** What problems do you have when you eat out? Is it difficult to stay within your fat gram and calorie goals when you eat at these places? What specifically is difficult for you?

**Open responses.**

**Refer** to the “4 keys to healthy eating out” handout.

**Present:** There are several ways to stick to healthy eating patterns when eating out.

1. Plan ahead. If you have a plan, you can think about possible problems you might have and handle them more easily.
2. Ask for what you want. Be friendly but firm. We will talk about this in a moment. By now you know what you need and want, so we want you to be able to get that as smoothly and respectfully as possible.
3. Take charge of what’s around you. Make positive use of what is around you, such as encouraging each other to make healthy choices. If you can, get rid of the items (for example, appetizers) that stand in the way of you achieving your goal.
4. Choose foods carefully.

Using these 4 keys is easy in some situations, and more difficult in others. Let’s practice how to use them in different places where we eat outside of our own houses.

We will start with restaurants where a server takes our order at the table.

Restaurants with servers

**Present:** Let’s say we are going to (choose one of their favorite non-fast food restaurants; if they brought a menu that is better.)

**Ask:** What are some ways that you can plan ahead for eating out at __________ restaurant?

**Open responses.**

**Refer** participants to the “Plan Ahead” handout.

This handout gives you several ways to plan ahead.

**Suggest** these tips for planning ahead when eating at a restaurant, if they were not given by the participants. You do not need to use all of them; use your judgment to see which ones might be the most helpful.

- Pick the restaurant carefully. It is better if you pick one with low-fat or low-calorie choices. Stay away from buffets, brunches and other all-you-can-eat meals.
- Eat fewer calories and less fat during meals a day or two before you plan to eat out. In other words, save some calories for a few days, and use them when you eat out.
- Eat a small, healthy snack before you go to the restaurant so that you are not very hungry when you get there. Examples are: fruit, low fat crackers and water.
- Plan what to order without looking at the menu. Looking at the menu can tempt you to order something that is not as healthy.

**Present:** We have talked about planning ahead. Now, once you get to ______ restaurant, how would you make sure you get what you want?

**Open responses.**

**Refer** participants to the “Ask for what you want” handout.

**Present:** Some people may find it hard at first to ask a server for something special. With practice, asking becomes easier. This handout gives some tips for how to ask for what you want.

**Ask:** What would you say to the server if you want to make sure your food is served without sauce?

**Open responses.**

**Ask:** What would you say to the server if the food comes to you with sauce that you did not want?

**Open responses.**

**Present:** In a moment, you will have the chance to practice ordering food and asking for exactly what you want. With time and practice, you will not feel uncomfortable in asking.

**Suggest** (if not already suggested by the group):

- Be firm and friendly. Remember you are paying for the meal. Most restaurants want to make you happy.
- Ask for food substitutions. For example, ask for salad instead of French fries.
- Ask whether foods can be prepared in a different way. For example, ask that the fish be grilled and seasoned with lemon juice instead of fried. Ask for vegetables without sauce or butter.
- Do not be afraid to ask for foods that are not on the menu.
- Ask the server about the size of each food. You can say, “Can you please tell me how many grams of meat in the hamburger?”
- Ask for salad dressing, sauce, butter on the side. Then you can control the amount. If you dip your fork into the sauce or dressing, you will use less, and reduce your fat and calorie intake.
- Ask for less cheese or no cheese.
- Share a main dish with someone.
- Order a smaller size (children’s, appetizer, half-portion).
- Before the meal, have the amount you do not want to eat set aside for take-away.

**Present:** in an earlier session we discussed the importance of taking charge of what’s around you when trying to make healthy choices. Do you remember what this means? What are some ways that you can take charge?

**Open responses.**

**Refer** to the “Take charge of what’s around you” handout.

**Present:** This handout offers a few ways to take control and stay focused on your goals. With all the possible problems of eating at a restaurant, it is important to remember your goals and what your plan is to achieve them.

**Suggest** these tips, if not already suggested.

- When appropriate, be the first to order. Then you will not be tempted by what others order, and they may even follow your healthy example.
- Keep foods off the table that you do not want to eat.
- If the server brings bread, chips or other foods that you do not want, say, “No, thank you,” or put it in a place where you can’t reach it if others at your table want it.
- When you order something, ask that half of it be put aside for take-away before they bring it to the table. Then have them bring it to you at the end of the meal.
- Remove from the table any advertisements for high-fat or high-calorie foods (desserts, appetizers, special drinks).

**Present:** Unhealthy choices are almost always available at restaurants, so it is important that you make good choices. Even if you plan ahead, ask for what you want, and take control, it is still up to you to choose healthy foods.

**Ask:** What do you remember about healthy choices from earlier sessions? What are some of the main ways we can stay focused?

**Open responses.**

**Refer** to the “Choose your food carefully” handout.

**Present:** This handout lists healthy and unhealthy food choices that you already know. You can tell a lot from the words on a menu. One thing to look for are words that indicate whether foods are high-fat or low-fat. This handout lists words that describe foods that are healthier, and words that describe foods you should avoid or limit.

**Suggest:** If not already mentioned by the group, you can mention these:

- Be cautious of sauces on meats and vegetables. Ask that these foods be without sauce or with the sauce on the side.
- Think about how much food you really need. Make some compromises: “I would rather have a small dessert, so I won’t have any rice.”
- Trim visible fat off meat and remove the skin from chicken.

**Present:** Now, let’s look at a local menu.

**Distribute** copies of one of the menus you brought to the class.

**Ask** each participant to circle a healthy menu item they would choose to include in a meal.

**Discuss** their choices as a group.

**Ask:** Was it difficult to find an item that worked for you?

**Open responses.**

**Refer** participants to the “What’s on the menu?” hand out.

**Present:** Take a look at this list and try to find low-fat and low-calorie items that appeal to you.

Even when we know which items are healthy, we do not always choose them. It takes practice to know which choices are the best choices, but it also takes commitment to decide to order them.

Now let’s look at how we can use the keys to healthy eating with fast food.

Fast food restaurants

**Ask:** How many of you have ever eaten fast food?

**Present:** Almost all of us have eaten fast food at one time or another, and some of us eat it frequently because it is so easy to get. And some of us really like the way it tastes! Although fast food is usually not the best option for healthy eating, sometimes we cannot avoid it. Some fast food restaurants are starting to offer healthier, lower-fat and lower-calorie items.

**Refer** participants to the “Fast food *can* be lower in fat and calories” handout.

**Present:** Take a moment to look over these lower-fat, lower-calorie options.

**Ask:** Does anything surprise you?

**Open responses.**

**Present:** Here are keys to healthy eating out at fast food restaurants.

Plan ahead.

- Pick a restaurant with care. Some fast food restaurants have low-fat, low-calorie foods, such as salad with low-fat dressing, grilled chicken, etc. Pick those whenever possible.
- Plan what you will order without looking at the menu. Menus can cause you to order foods that are less healthy.

Ask for what you want. Be firm and friendly.

- For example, “May I have my coffee with a little low-fat milk instead of Rainbow?” or “Please, no mayonnaise on the burger.”

Take charge of what’s around you.

- Be the first in your group to order. You will not be tempted by what or how much others order, and you may set a good example for them.

Choose foods carefully.

- Try grilled chicken sandwiches instead of fried, try salads with low-calorie dressing, choose items without sauce.
- Stay away from French fries. If you must have them, order a small size and try not to finish them.
- If you must have a hamburger, order a smaller size, without cheese.

Extended family gatherings

**Present:** In many ways, this one is the most difficult. Our social lives include eating together: dinner with cousins, time at the mall, weddings and parties, and holidays. Yet even at these occasions, we can use the 4 keys to eating healthier and meeting our goals.

**Ask:** What are some ways you can eat healthily at dinner at a cousin’s house or at a party?

**Open responses.**

**Suggest** these ideas, if not already suggested:

Plan ahead.

- For meals where everyone brings something, bring something healthy as your contribution: fruit salad, salad with low-calorie dressing, etc.
- Talk to the host or hostess before you go, if it is appropriate, especially if you eat at their home frequently. Ask them for their support and help in reaching your goal of losing weight.
- Eat a small, healthy snack before you go, so it is easier to control how much and what you eat when you get there.

Ask for what you want. Be firm and friendly.

- When offered a food that is not healthy, say, “It looks really good, but no thank you.”

Take charge of what’s around you.

- At buffets or parties, stay away from the food table. Choose a small plate, and after serving yourself, sit at a table farther from the food.

Choose foods carefully.

- Take only a small amount of foods that are high-fat and high-calorie foods, just enough to enjoy the taste.
- Look at everything on the buffet before taking food for yourself. Then choose three or four of the ones that look the best, instead of having everything.

Airplanes and airports

**Present:** On airplanes and in airports, we have similar challenges, but fewer choices.

**Ask:** How many of you have traveled by airplane recently? What were your healthy food options the last time you traveled by airplane?

**Open responses.**

**Present:** We can again use the same 4 keys to try to avoid unhealthy foods and physical inactivity when we travel.

Plan ahead.

- Plan the meals and snacks you will have while traveling. Remember to include your time waiting in the airport and during your flight.
- Bring healthy snacks with you, and consider whether you can bring a healthy meal with you if the flight is going to be long.

Ask for what you want. Be firm and friendly./Choose foods carefully.

- Ask for fruit or other healthy snacks on longer flights.

Take charge of what’s around you.

- Say, “No, thank you,” when offered unhealthy snacks on the airplane.

*Part 3: Practice getting what you want (25 minutes)*

**Present:** Before we begin practicing with each other, let’s review one more time the 4 steps:

1. Take the time to plan ahead. Know where you are going. Make adjustments to your food plan for that day when you are not at home so you can eat healthfully.
2. Be firm and friendly in asking for what you want.
3. Take charge of what’s around you- try to eliminate things that make healthy eating a challenge.
4. Choose the healthiest foods possible. If there are no healthy foods, do the best you can and adjust your other meals that day.

**Ask** whether there are any questions.

**Present:** Using the handouts for this session, let’s practice ordering from a menu. I know this may seem uncomfortable and strange, but it is important to practice out loud so that you can choose words that work for you. I will play the part of the server and take your order.

Let’s look at the “What’s on the menu?” and the “Fast food can be lower in fat and calories” handouts.

**Distribute** a copy of the menu to each participant of the second menu you brought for the practice activity.

**Ask:** Please read all the menu options and consider which items are low-fat or low-calorie items you would eat instead of the high-fat and high-calorie choices.

**Ask** each participant, one after the other, for their order. They can use their handouts if needed. Remind them to use a firm but friendly tone and words.

**Discuss** their choices and why they chose that option. Offer feedback for how to order, and how to ask for substitutions. Be positive and encouraging.

**Present:** This activity gives you a start on using the 4 steps to get what you want. Now let’s make a plan for next week.

*Part 4: Wrap up and to do list (5 minutes)*

**Ask** whether participants have any question about what was covered during the session.

**Refer** participants to the “A positive action plan” handout.

**Present:** Think of a problem you have when you eat outside the house.

1. Write the problem on the handout.
2. Choose one of the 4 keys we practiced today. Choose one that is likely to help you solve the problem and one you can do without much difficulty.
3. Fill in the rest of the handout to complete your positive action plan.

For next week:

**Keep track** of your weight, eating and activity.

**Try your action plan.** Next week, we will talk about the answer to two questions: “Did your plan work?” and “If not, what went wrong?” There is space at the bottom of your to do list to write down the answers so you are ready to discuss them next time.

**Ask** if there are any questions or concerns.

**Summarize the key points.**

- **You were introduced to 4 basic keys for healthy eating out:**
  - Plan ahead.
  - Be firm and friendly in asking for what you want.
  - Take charge of what’s around you.
  - Choose healthy foods.
- **You were given examples of how to use these keys when you eat outside of your house.**
- **We practiced making healthy meal selections from a restaurant menu.**
- **We practiced making healthy substitutions and asking for them from a server.**

**Close:** It is often a challenge to stay focused on a goal in places where you feel you have less or even no control. But you can gain control over your situation by using the four key steps we practiced for making healthy food choices when you are not at home. Be confident and ask for what you want.

Next week we will look at ways to overcome negative thoughts that get in the way of our goals.

**Ask** whether participants have any questions or concerns.

**After the session:**

Review an make notes on their “Food and Activity Trackers”.
