# Supplementary material for: Emirates Heart Health Project (EHHP): A protocol for a stepped-wedge family-cluster randomized-controlled trial of a health-coach guided diet and exercise intervention to reduce weight and cardiovascular risk in overweight and obese UAE nationals
Source: PLoS One. 2023 Apr 10;18(4):e0282502. doi: 10.1371/journal.pone.0282502 (PMC10085020; doi:10.1371/journal.pone.0282502)
Supplement: S24 Appendix — (DOCX) [file pone.0282502.s024.docx]

الجلسة 10: 4 مفاتيح لتناول الطعام الصحي بالخارج

**أهداف التعلم**

في ختام هذه الجلسة سيتمكن المشاركون من:

- وصف قائمة المفاتيح الأربعة لتناول الطعام الصحي في الخارج.
- أعطاء أمثلة على كيفية تطبيق هذه المفاتيح في المطاعم التي يذهب إليها المشاركون بانتظام.
- القيام باختيار وجبة مناسبة من قائمة المطعم.
- توضّيح كيفية طلب عنصر بديل باستخدام لغة حازمة ونبرة صوت مهذبة.

**المواد**

- نشرات المشاركين للدورة 10:
- نظرة عامة على الجلسة 10
- 4 مفاتيح للأكل الصحي بالخارج
- خطط مسبقا
- أسأل عن ما تريد
- تولي مسؤولية ما حولك
- اختر طعامك بعناية
- ما ذا يوجد بالقائمة؟
- يمكن أن تكون الوجبات السريعة منخفضة الدهون
- خطة عمل إيجابية
- مهام الأسبوع المقبل
- متتبع الغذاء والنشاط للجلسة 10
- علامات الأسماء
- لوحة بيضاء واقلام
- تذوق القوائم من المطاعم المفضلة.
- واحد للمناقشة ، مع نسخ كافية لكل مشارك.
- واحد للنشاط التدريبي ، مع نسخ كافية لكل مشارك.

**نظرة عامة على الجلسة**

تواصل الجلسة 10 عملية مساعدة المشاركين على التحكم في محيطهم من خلال منحهم أدوات لاتخاذ خيارات صحية عند تناول الطعام في الخارج. يتطلب تناول الطعام بطريقة صحية من المشاركين التخطيط للمستقبل ، والبقاء مركزًا وحزمًا في السؤال عما يريدون.

تتكون الجلسة 10 من 4 أجزاء:

الجزء الأول: التقدم والمراجعة الأسبوعية (5 دقائق)

الجزء الثاني: مفاتيح تناول الطعام الصحي (25 دقيقة)

سوف نتحدث عن تناول الطعام بالخارج في مواقف مختلفة (زيارات للعائلة والمطاعم والحفلات) واستخدام 4 مفاتيح لتناول الطعام الصحي في الخارج:

1. التخطيط للمستقبل.

2. اسأل عما تريد.

3. تولي مسؤولية ما حولك.

1. اختر الأطعمة بعناية.

الجزء 3: تدرب على الحصول على ما تريد (25 دقيقة)

يمارس المشاركون المفاتيح الأربعة عن طريق لعب الأدوار معك كخادم ، باستخدام القوائم الحقيقية والنشرات.

الجزء الرابع: ختام وقائمة المهام (5 دقائق)

**الرسائل الرئيسية:**

يعد تناول الطعام بالخارج (سواء في منزل شخص ما أو في مطعم أو حفلة أو في طائرة) تجربة شائعة للكثير من الناس ، ولكن القيام بذلك يمكن أن يفرض العديد من التحديات للأكل الصحي.

يمكن تناول الطعام خارج المنزل والأكل الصحي. ومع ذلك ، فإن القيام بذلك يتطلب التخطيط والاتصال الفعال واختيار الطعام بعناية.

الجزء الأول: التقدم والمراجعة الأسبوعية (5 دقائق)

**وزع** عليهم :

نشرات الجلسة 10

- "متتبعو الطعام والنشاط" للجلسة 10
- "متتبعو الطعام والنشاط" الجلسة 8 مع ملاحظاتك وتوصياتك.
- اجمع "متتبعو الطعام والنشاط"للجلسة 9.

**ناقش** نجاح المشاركين والصعوبات في تحقيق أهدافهم في إنقاص الوزن.

**اسأل:** ما الذي سار بشكل جيد وغير جيد في تتبع طعامك ونشاطك البدني الأسبوع الماضي؟ هل كنت قادرًا على البقاء في حدود غرام الدهون والسعرات الحرارية؟ هل تمكنت من الوصول إلى هدف النشاط البدني؟

**استجابات مفتوحة.**

**حاضر:** تعلمنا الأسبوع الماضي حوالي 5 خطوات لحل المشكلات وتمرننا على استخدامها لحل المشكلات التي تعترض طريق تحقيق أهدافنا.

**اسأل:** هل جربت خطة العمل الخاصة بك؟ ماذا تعلمت من عملية حل المشكلات؟

**استجابات مفتوحة.**

**عالج** أي مشاكل حول ما طُلب منه القيام به الأسبوع الماضي. تذكر أن تمدح كل تقدم ، مهما كان صغيرًا. ناقش الحواجز وحل المشكلات مع المشاركين للتغلب على الحواجز.

**حاضر**: هذا الأسبوع سوف:

**نناقش** المفاتيح الأربعة لتناول الطعام الصحي في الخارج: 1) خطط للمستقبل ، 2) اسأل عما تريد ، 3) تولي مسؤولية ما حولك ، و 4) اختر الأطعمة الصحية.

اذكر أمثلة على كيفية استخدام هذه المفاتيح الأربعة في الأماكن التي تتناول فيها الطعام (المطاعم والحفلات ومنازل الأشخاص الآخرين.

تحدّث عن كيفية إجراء اختيارات الوجبات الصحية من قائمة المطاعم.

تعرف على كيفية السؤال بثقة عن استبدال عنصر باستخدام اللغة المناسبة ونبرة الصوت المناسبة.

الجزء الثاني: مفاتيح تناول الطعام الصحي (25 دقيقة)

**حاضر:** سنتحدث اليوم عن تناول الطعام خارج منزلك. إنه شيء نقوم به جميعًا والذي يمثل تحديًا خاصًا عندما نحاول إجراء تغييرات صحية في نمط الحياة.

**اسأل:** أين تأكل عادة عندما تأكل خارج المنزل؟ أي مطاعم مفضلة؟ هل أحضرت قوائم المطاعم المفضلة لمشاركتها معنا؟

**ملاحظة:** إذا أحضر المشاركون قوائم ، فاختر 1 أو 2 منها لاستخدامها كأمثلة لبقية الجلسة. إذا لم يأتوا بالقوائم ، فاطلب منهم بعض الأمثلة: مطاعم الوجبات السريعة ، أو منازل الأصدقاء أو أفراد الأسرة ...

**استجابات مفتوحة.**

**اسأل:** ما هي المشاكل التي لديك عند تناول الطعام في الخارج؟ هل من الصعب أن تظل ضمن أهدافك من الدهون والسعرات الحرارية عند تناول الطعام في هذه الأماكن؟ ما هو الصعب على وجه التحديد بالنسبة لك؟

**استجابات مفتوحة.**

**ارجع** إلى نشرة "4 مفاتيح لتناول الطعام الصحي".

**حاضر**: هناك عدة طرق للالتزام بأنماط الأكل الصحي عند تناول الطعام في الخارج.

1. التخطيط للمستقبل. إذا كان لديك خطة ، يمكنك التفكير في المشاكل المحتملة التي قد تواجهها والتعامل معها بسهولة أكبر.

2. اسأل عما تريد. كن ودودًا ولكن حازمًا. سنتحدث عن هذا في لحظة. الآن أنت تعرف ما تحتاجه وتريده ، لذلك نريدك أن تكون قادرًا على الحصول على ذلك بسلاسة واحترام قدر الإمكان.

3. تولي مسؤولية ما حولك. استخدم ما هو إيجابي حولك بشكل إيجابي ، مثل تشجيع بعضهم البعض على اتخاذ خيارات صحية. إذا استطعت ، تخلص من العناصر (على سبيل المثال ، المقبلات) التي تقف في طريق تحقيق هدفك.

4. اختر الأطعمة بعناية.

يعد استخدام هذه المفاتيح الأربعة أمرًا سهلاً في بعض المواقف وأكثر صعوبة في حالات أخرى. دعونا نتدرب على كيفية استخدامها في أماكن مختلفة حيث نتناول الطعام خارج منازلنا.

سنبدأ مع المطاعم حيث يأخذ النادل طلبنا على الطاولة.

مطاعم مع خوادم او نادل

**حاضر**: لنفترض أننا سنختار (اختر أحد مطاعمهم المفضلة غير السريعة ، إذا أحضروا قائمة طعام أفضل.)

**اسأل**: ما هي بعض الطرق التي يمكنك التخطيط بها مسبقًا لتناول الطعام في مطعم __________؟

**استجابات مفتوحة.**

**قم بإحالة** المشاركين إلى نشرة "الخطة المستقبلية".

تمنحك هذه النشرة عدة طرق للتخطيط للمستقبل.

**اقترح** هذه النصائح للتخطيط المسبق عند تناول الطعام في مطعم ، إذا لم يعطها المشاركون. لا تحتاج لاستخدام كل منهم. استخدم حكمك لمعرفة أيها قد يكون الأكثر فائدة.

- اختر المطعم بعناية. من الأفضل أن تختار واحدًا بخيارات قليلة الدهون أو منخفضة السعرات الحرارية. ابتعد عن البوفيهات والغداء والوجبات الأخرى التي يمكنك تناولها.
- تناول سعرات حرارية أقل ودهونًا أقل أثناء الوجبات يوميًا أو يومين قبل أن تخطط لتناول الطعام بالخارج. وبعبارة أخرى ، احفظ بعض السعرات الحرارية لبضعة أيام ، واستخدمها عند تناول الطعام بالخارج.
- تناول وجبة خفيفة صغيرة وصحية قبل الذهاب إلى المطعم حتى لا تشعر بالجوع الشديد عندما تصل إلى هناك. ومن الأمثلة على ذلك: الفاكهة والمقرمشات قليلة الدسم والماء.
- خطط لما تطلبه دون النظر إلى القائمة. يمكن أن يجذبك النظر إلى القائمة لطلب شيء غير صحي.

**حاضر:** لقد تحدثنا عن التخطيط للمستقبل. الآن ، بمجرد الوصول إلى مطعم ______ ، كيف تتأكد من حصولك على ما تريد؟

**استجابات مفتوحة.**

**قم بإحالة** المشاركين إلى نشرة "اسأل عما تريد".

**حاضر**: قد يجد بعض الأشخاص صعوبة في البداية في سؤال النادل عن شيء خاص. مع الممارسة يصبح السؤال أسهل. توفر هذه النشرة بعض النصائح حول كيفية السؤال عما تريد.

**اسأل:** ماذا تقول للنادل إذا كنت تريد التأكد من تقديم طعامك بدون صلصة؟

**استجابات مفتوحة.**

اسأل: ماذا تقول للنادل إذا جاء الطعام إليك بالصوص الذي لا تريده؟

**استجابات مفتوحة.**

**حاضر:** ستتاح لك الفرصة للحظة لممارسة طلب الطعام وطلب ما تريده بالضبط. مع الوقت والممارسة ، لن تشعر بعدم الارتياح في السؤال.

**اقتراح** (إذا لم تقترحه المجموعة بالفعل):

- كن حازمًا ووديًا. تذكر أنك تدفع للوجبة. تريد معظم المطاعم أن تجعلك سعيدًا.
- اطلب بدائل الغذاء. على سبيل المثال ، اطلب سلطة بدلاً من البطاطس المقلية.
- اسأل عما إذا كان يمكن تحضير الأطعمة بطريقة مختلفة. على سبيل المثال ، اطلب شوي السمك وتتبيله بعصير الليمون بدلاً من قليه. اطلب الخضار بدون صوص أو زبدة.
- لا تخف من طلب الأطعمة غير الموجودة في القائمة.
- اسأل النادل عن حجم كل طعام. يمكنك أن تقول ، "هل يمكنك أن تخبرني كم عدد جرامات اللحم في الهامبرغر؟"
- اطلب صلصة السلطة والصلصة والزبدة على الجانب. ثم يمكنك التحكم في الكمية. إذا غطست شوكة في الصلصة ، فستستخدم كمية أقل ، وتقلل من استهلاك الدهون والسعرات الحرارية.
- اطلب جبن أقل أو بدون جبن.
- شارك الطبق الرئيسي مع شخص ما.
- اطلب حجمًا أصغر (حجم الأطفال ، المقبلات ، نصف وجبة).
- قبل تناول الوجبة ، خذ الكمية التي لا تريد تناولها جانبًا لتناولها.

**حاضر:** ناقشنا في جلسة سابقة أهمية تولي مسؤولية ما حولك عند محاولة اتخاذ خيارات صحية. هل تتذكر ماذا يعني هذا؟ ما هي بعض الطرق التي يمكنك من خلالها تولي المسؤولية؟

**استجابات مفتوحة.**

**ارجع إلى نشرة** "تولي مسؤولية ما حولك".

**حاضر:** تقدم هذه النشرة بضع طرق للتحكم ومواصلة التركيز على أهدافك. مع كل المشاكل المحتملة لتناول الطعام في المطعم ، من المهم أن تتذكر أهدافك وما هي خطتك لتحقيقها.

**اقترح** هذه النصائح ، إذا لم تكن مقترحة بالفعل.

- عند الطلب, كن أول من يطلب. ثم لن يجذبك ما يطلبه الآخرون ، وقد يتبعون مثالك الصحي.
- أبعد الأطعمة التي لا ترغب في تناولها عن المائدة.
- إذا كان النادل يجلب الخبز أو الرقائق أو الأطعمة الأخرى التي لا تريدها ، قل "لا ، شكرًا لك" ، أو ضعها في مكان لا يمكنك الوصول إليه إذا أراده الآخرون على طاولتك.
- عندما تطلب شيئًا ، اطلب وضع نصفه جانبًا قبل أن يحضره إلى الطاولة. ثم اطلب منهم إحضارها لك في نهاية الوجبة.
- أزل من الجدول أي إعلانات للأطعمة الغنية بالدهون أو ذات السعرات الحرارية العالية (الحلويات والمقبلات والمشروبات الخاصة).

**حاضر**: تتوفر الخيارات غير الصحية دائمًا في المطاعم ، لذلك من المهم أن تتخذ خيارات جيدة. حتى إذا كنت تخطط مسبقًا ، اسأل عما تريد ، وتحكم ، فلا يزال بإمكانك اختيار الأطعمة الصحية.

**اسأل**: ماذا تتذكر عن الاختيارات الصحية من الجلسات السابقة؟ ما هي بعض الطرق الرئيسية التي يمكننا من خلالها التركيز؟

**استجابات مفتوحة.**

**ارجع** الى منشور "اختر طعامك بعناية".

**حاضر:** يسرد هذا المنشور خيارات الطعام الصحي وغير الصحي التي تعرفها بالفعل. يمكنك معرفة الكثير من الكلمات الموجودة في القائمة. شيء واحد يجب البحث عنه هو الكلمات التي تشير إلى ما إذا كانت الأطعمة عالية الدهون أم قليلة الدهون. تسرد هذه النشرة الكلمات التي تصف الأطعمة الأكثر صحة ، والكلمات التي تصف الأطعمة التي يجب تجنبها أو الحد منها.

**اقتراح**: إذا لم تكن المجموعة قد ذكرت بالفعل ، فيمكنك ذكر ما يلي:

- احذر من الصلصات على اللحوم والخضروات. اطلب أن تكون هذه الأطعمة خالية من الصلصة أو مع الصلصة على الجانب.
- فكر في كمية الطعام التي تحتاجها حقًا. تقديم بعض التنازلات: "أفضل تناول حلوى صغيرة ، لذا لن يكون لدي أي أرز".
- ازالة الدهون المرئية من اللحوم وإزالة الجلد من الدجاج.

**حاضر**: الآن ، دعنا ننظر إلى قائمة محلية.

**وزع** نسخ من القوائم التي أحضرتها إلى الفصل.

**اطلب** من كل مشارك وضع دائرة حول قائمة طعام صحية يختارون اضافتها في الوجبة.

**ناقش** خياراتهم كمجموعة.

**اسأل:** هل كان من الصعب العثور على عنصر يناسبك؟

**استجابات مفتوحة.**

**إحالة** المشاركين إلى مذكرة "ماذا يوجد في القائمة؟".

**حاضر:** ألق نظرة على هذه القائمة وحاول العثور على العناصر قليلة الدهون والسعرات الحرارية التي تعجبك.

حتى عندما نعرف العناصر الصحية ، فإننا لا نختارها دائمًا. يتطلب الأمر معرفة أي الخيارات هي أفضل الخيارات ، ولكن الأمر يتطلب أيضًا الالتزام بترتيبها.

فلنلقِ نظرة الآن على كيفية استخدام مفاتيح الأكل الصحي مع الوجبات السريعة.

مطاعم الوجبات السريعة

**اسأل:** كم منكم تناول طعامًا سريعًا؟

**حاضر**: لقد تناولنا جميعًا تقريبًا الوجبات السريعة في وقت أو آخر ، والبعض منا يأكلها كثيرًا لأنه من السهل الحصول عليها. والبعض منا حقا يحب طعمها! على الرغم من أن الوجبات السريعة ليست عادةً الخيار الأفضل لتناول الطعام الصحي ، إلا أنه في بعض الأحيان لا يمكننا تجنبها. بدأت بعض مطاعم الوجبات السريعة في تقديم أصناف صحية وقليلة الدهون والسعرات حرارية.

**إحالة** المشاركين إلى نشرة "الوجبات السريعة يمكن أن تكون أقل في الدهون والسعرات الحرارية".

**حاضر**: خذ لحظة لإلقاء نظرة على هذه الخيارات قليلة الدسم والسعرات الحرارية .

**اسأل:** هل يفاجئك أي شيء؟

**استجابات مفتوحة.**

**حاضر**: فيما يلي مفاتيح تناول الطعام الصحي في مطاعم الوجبات السريعة.

خطط مسبقا و اختر مطعمًا بعناية. تحتوي بعض مطاعم الوجبات السريعة على أطعمة قليلة الدسم وقليلة السعرات الحرارية ، مثل السلطة مع تتبيلة قليلة الدسم والدجاج المشوي وما إلى ذلك.

- خطط لما ستطلبه دون النظر إلى القائمة. قد تتسبب القوائم في طلب الأطعمة الأقل صحة.

أسأل عن ما تريد. كن حازمًا ووديًا.

- على سبيل المثال ، "هل يمكنني تناول قهوتي مع القليل من الحليب قليل الدسم بدلاً من حليب قوس قزح؟" أو "من فضلك ، لاأريد مايونيز على البرجر".

تولي مسؤولية ما حولك.

- كن الأول في مجموعتك للطلب. لن يتم إغراءك بما يطلبه الآخرون أو بمقدار ذلك ، ويمكنك أن تكون مثالًا جيدًا لهم.

اختر الأطعمة بعناية.

- جرب شطائر الدجاج المشوي بدلاً من المقلي ، جرب السلطات مع صلصة منخفضة السعرات الحرارية ، اختر العناصر بدون صلصة.
- ابتعد عن البطاطس المقلية. إذا كان يجب أن يكون لديك ، اطلب حجمًا صغيرًا وحاول ألا تنهيها.
- إذا كان يجب أن يكون لديك همبرغر ، اطلب حجمًا أصغر بدون جبن.

التجمعات العائلية الممتدة

**حاضر: يعتبر** الأصعب من عدة نواحي. تشمل حياتنا الاجتماعية تناول الطعام معًا: العشاء مع أبناء العم ، او في المركز التجاري ، وحفلات الزفاف والحفلات الاخرى ، والعطلات. ولكن حتى في هذه المناسبات ، يمكننا استخدام المفاتيح الأربعة لتناول طعام صحي وتحقيق أهدافنا.

**اسأل:** ما هي بعض الطرق التي يمكنك من خلالها تناول الطعام الصحي في العشاء في منزل ابن عمك أو في حفلة؟

**استجابات مفتوحة.**

اقترح هذه الأفكار ، إذا لم تكن مقترحة بالفعل:

خطط مسبقا.

- بالنسبة للوجبات التي يحضر فيها كل شخص شيئًا ما ، احضر شيئًا صحيًا كمساهمة منك: سلطة فواكه ، سلطة بتتبيلة منخفضة السعرات الحرارية ، إلخ.
- تحدث إلى المضيف أو المضيفة قبل أن تذهب ، إذا كان ذلك مناسبًا ، خاصة إذا كنت تأكل في المنزل بشكل متكرر. اطلب منهم دعمهم ومساعدتهم لك في الوصول إلى هدفك المتمثل في فقدان الوزن.
- تناول وجبة خفيفة صغيرة وصحية قبل أن تذهب ، ليكون من السهل عليك التحكم في كمية ما تأكله عندما تصل إلى هناك.

أسأل عن ما تريد. كن حازمًا ووديًا.

- عندما يتم تقديم طعام غير صحي ، قل: "يبدو جيدًا حقًا ، ولكن لا أريد شكرًا لك".

تولي مسؤولية ما حولك.

- في البوفيهات أو الحفلات ، ابتعد عن طاولة الطعام. اختر طبقًا صغيرًا ، وبعد تقديم نفسك ، اجلس على طاولة أبعد من الطعام.

اختر الأطعمة بعناية.

- خذ فقط كمية صغيرة من الأطعمة التي تحتوي على نسبة عالية من الدهون وذات السعرات الحرارية ، ما يكفي للاستمتاع بالطعم.
- انظر إلى كل شيء في البوفيه قبل تناول الطعام بنفسك. ثم اختر ثلاثة أو أربعة من تلك التي تبدو الأفضل ، بدلاً من الحصول على كل شيء.

الطائرات والمطارات

**حاضر:** في الطائرات وفي المطارات ، لدينا تحديات مماثلة ، ولكن خيارات أقل.

**اسأل**: كم منكم سافر بالطائرة مؤخرًا؟ ما هي خيارات طعامك الصحي في آخر مرة سافرت فيها بالطائرة؟

**استجابات مفتوحة.**

**حاضر:** يمكننا مرة أخرى استخدام المفاتيح الأربعة نفسها لمحاولة تجنب الأطعمة غير الصحية والخمول البدني عند السفر.

خطط مسبقا.

- خطط الوجبات والوجبات الخفيفة التي ستحصل عليها أثناء السفر. تذكر تضمين وقتك في المطار وأثناء رحلتك.
- اصطحب معك وجبات خفيفة صحية ، وفكر فيما إذا كان بإمكانك إحضار وجبة صحية معك إذا كانت الرحلة ستكون طويلة.

أسأل عن ما تريد. كن حازمًا ووديًا. / اختر الأطعمة بعناية.

- اطلب الفاكهة أو الوجبات الخفيفة الصحية الأخرى على الرحلات الطويلة.

تولي مسؤولية ما حولك.

- قُل "لا ، شكرًا" ، اذا عُرضت وجبات خفيفة غير صحية على متن الطائرة.

الجزء 3: تدرب على الحصول على ما تريد (25 دقيقة)

**حاضر:** قبل أن نبدأ في التدرب مع بعضنا البعض ، فلنراجع مرة أخرى الخطوات الأربع التالية:

1. خذ الوقت الكافي للتخطيط للمستقبل. اعرف إلى أين أنت ذاهب. قم بإجراء تعديلات على خطة طعامك لذلك اليوم عندما لا تكون في المنزل حتى تتمكن من تناول الطعام الصحي.

2. كن حازمًا ووديًا في طلب ما تريد.

3. تولي مسؤولية ما حولك - حاول التخلص من الأشياء التي تجعل الأكل الصحي تحديًا.

4. اختر أصح الأطعمة الممكنة. إذا لم تكن هناك أطعمة صحية ، افعل أفضل ما يمكنك وعدل وجباتك الأخرى في ذلك اليوم.

**اسأل** عما إذا كانت هناك أي أسئلة.

**حاضر:** باستخدام النشرات لهذه الجلسة ، دعنا نمارس الطلب من القائمة. أعلم أن هذا قد يبدو غير مريح وغريب ، ولكن من المهم التمرن بصوت عالٍ حتى تتمكن من اختيار الكلمات التي تناسبك. سوف ألعب دور النادل وأخذ طلبك.

فلنلق نظرة على "ماذا يوجد في القائمة؟" و "الوجبات السريعة يمكن أن تكون أقلمن حيث الدهون والسعرات الحرارية".

**وزع** نسخة من القائمة الثانية التي أحضرتها لنشاط التدريب على كل مشارك

**اسأل:** يرجى قراءة جميع خيارات القائمة والنظر في العناصر قليلة السعرات أو منخفضة السعرات الحرارية التي ستأكلها بدلاً من الخيارات عالية الدهون والسعرات الحرارية.

**اطلب** من كل مشارك ، واحدًا تلو الآخر ، عن طلباتهم. بامكانهم الرجوع للمنشورات التي لديهم. ذكرهم باستخدام نبرة صوت وكلمات حازمة ولكن ودودة.

**ناقش** خياراتهم ولماذا اختاروا هذه الخيارات. **قدّم** تعليقات حول كيفية الطلب وكيفية طلب الاستبدال. كن إيجابيًا ومشجعًا.

**حاضر:** يعطيكم هذا النشاط البداية للحصول على ما تريد باستخدام الخطوات الأربع. الآن دعنا نضع خطة للأسبوع المقبل.

الجزء 4: ختام وقائمة المهام (5 دقائق)

**اسأل** عما إذا كان لدى المشاركين أي سؤال حول ما تمت تغطيته خلال الجلسة.

**قم بإحالة** المشاركين إلى نشرة "خطة عمل إيجابية".

**حاضر:** فكر في مشكلة لديك عندما تأكل خارج المنزل.

1. اكتب المشكلة على النشرة.

2. اختر أحد المفاتيح الأربعة التي مارسناها اليوم. اختر واحدًا من المحتمل أن يساعدك في حل المشكلة وآخر يمكنك القيام به دون صعوبة كبيرة.

3. املأ باقي النشرة لإكمال خطة العمل الإيجابية الخاصة بك.

للأسبوع القادم:

**تتبع** وزنك وتناول الطعام والنشاط.

**جرب** خطة العمل الخاصة بك. في الأسبوع المقبل ، سنتحدث عن إجابة سؤالين: "هل نجحت خطتك؟" و "إذا لم يكن الأمر كذلك ، فما الخطأ الذي حدث؟" توجد مساحة أسفل قائمة المهام لكتابة الإجابات حتى تكون مستعدًا لمناقشتها في المرة القادمة.

**اسأل** عما إذا كانت هناك أي أسئلة أو استفسارات.

**لخص** النقاط الرئيسية.

* تعرفت على 4 مفاتيح أساسية لتناول الطعام الصحي بالخارج:

- خطط مسبقا.
- كن حازمًا ووديًا في طلب ما تريد.
- تولي مسؤولية ما حولك.
- اختر الأطعمة الصحية.

* لقد تم إعطاؤك أمثلة حول كيفية استخدام هذه المفاتيح عندما تأكل خارج المنزل.

* تدربنا على اختيار وجبات صحية من قائمة المطاعم.

* تدربنا على إجراء بدائل صحية وطلبها من النادل.

**اختتام:** غالبًا ما يكون من الصعب الاستمرار في التركيز على هدف ما في الأماكن التي تشعر فيها أن لديك سيطرة أقل أو حتى لا تتحكم فيها. ولكن يمكنك التحكم في وضعك باستخدام الخطوات الرئيسية الأربع التي مارسناها من أجل اتخاذ خيارات طعام صحي عندما لا تكون في المنزل. كن واثقًا واطلب ما تريد.

في الأسبوع المقبل ، سننظر في طرق للتغلب على الأفكار السلبية التي تعترض أهدافنا.

**اسأل** عما إذا كان لدى المشاركين أي أسئلة أو مخاوف.

**بعد الجلسة:**

راجع وضع ملاحظات حول "متتبعو الطعام والنشاط".
