# Supplementary material for: Emirates Heart Health Project (EHHP): A protocol for a stepped-wedge family-cluster randomized-controlled trial of a health-coach guided diet and exercise intervention to reduce weight and cardiovascular risk in overweight and obese UAE nationals
Source: PLoS One. 2023 Apr 10;18(4):e0282502. doi: 10.1371/journal.pone.0282502 (PMC10085020; doi:10.1371/journal.pone.0282502)
Supplement: S25 Appendix — (DOCX) [file pone.0282502.s025.docx]

الجلسة 11: رد على الأفكار السلبية

أهداف التعلم

في نهاية هذه الجلسة ، سيتمكن المشاركون من:

- أعطاء أمثلة على الأفكار السلبية التي يمكن أن تمنعهم من تحقيق أهدافهم المتمثلة في فقدان الوزن وزيادة النشاط البدني.
- وصف كيف يمكن أن تتوقف عن الفكر السلبي وتتبدل معهم بأفكار إيجابية.
- ممارسة

1. وقف الأفكار السلبية

2. تبديل الأفكار السلبية بأفكار إيجابية.

**المواد**

المنشورات او المذكرات للمشاركين

- نظرة عامة على الجلسة 11
- الأفكار السلبية
- الرجوع عن الأفكار السلبية
- تدرب على الرد
- مهام الأسبوع المقبل

متتبعي الطعام والنشاط للجلسة 11

السبورة والاقلام

نظرة عامة على الجلسة

نواصل النقاش حول السيطرة على وضعنا والتعامل مع الأشياء التي تحدث كل يوم والتي تخلق تحديات ونحن نعمل نحو أهدافنا. نحن ننظر إلى طرق للسيطرة على الأفكار السلبية التي يمكن أن تعرقل تقدمنا. نحن ننظر عن كثب إلى كيفية التعرف على هذه الأفكار والتحدث معهم بأفكار إيجابية.

تنقسم الجلسة 11 إلى 4 أجزاء.

الجزء الأول: التقدم والمراجعة الأسبوعية (5 دقائق)

الجزء الثاني: ما هي الأفكار السلبية؟ (30 دقيقة)

حدد الأفكار السلبية ، و صف الأنواع المختلفة من الأفكار السلبية ، واعطي أمثلة لكل منها. على الرغم من أن بعض المشاركين قد يكونون غير مرتاحين ، حاول أن تشجعهم على النظر إلى أفكارهم السلبية الخاصة ، واطلب منهم مشاركة بعضها مع المجموعة.

الجزء الثالث: الرد على الأفكار السلبية (20 دقيقة)

اشرح للمشاركين كيفية الرد على الأفكار السلبية. باستخدام صورة علامة التوقف ، اطلب من المشاركين التمرن على التقاط الافكار السلبية. ووضح لهم كيفية الرد على الأفكار السلبية بأفكار إيجابية.

الجزء الرابع: ختام وقائمة المهام (5 دقائق)

الرسائل الرئيسية

- ي**مكن أن تكون الأفكار السلبية معتادة ومتأصلة لدرجة أننا لا ندركها أو ندرك مدى تأثيرها على سلوكنا.**
- **أقوى علاج للأفكار السلبية هو منعها في منتصف الجملة واستبدالها بالأفكار الإيجابية.**
- **غالبًا ما تكون الأفكار السلبية عادات تتشكل بمرور الوقت. بغض النظر عن مدى فعاليتنا في إيقافها ، فمن المحتمل أن يعودوا في مواقف مشابهة حتى نتعلم كيفية مطابقتها مع الأفكار الإيجابية.**
- **كل منا لديه أنواع مختلفة من الأفكار السلبية . عندما تؤدي هذه الأفكار إلى الأكل أو الخمول غير الصحي ، استخدم عملية حل المشكلات لإيجاد استراتيجيات تعمل بشكل أفضل في التغلب على أفكار هزيمة الذات.**

الجزء الأول: التقدم والمراجعة الأسبوعية (5 دقائق)

**توزيع:** نشرات الجلسة 11 ، متتبعو الطعام والنشاط للجلسة 9 ، و وزع متتبعو الطعام والنشاط للجلسة 10 مع ملاحظاتك و اي توصيات مخصصة.

**اجمع**  متتبعات الطعام والنشاط **ا**لجلسة 9 .

**ناقش** نجاحات وتحديات المشاركين في الأسبوع الماضي.

**اسأل**: هل واجهت أي صعوبة في تتبع تناول الطعام والنشاط البدني الأسبوع الماضي؟ هل كنت قادرًا على البقاء تحت ميزانية غرام الدهون والسعرات الحرارية؟ هل وصلت إلى هدفك للنشاط البدني؟

**استجابات مفتوحة**. امتدح اي تقدم مهما كان صغيراً.

**حاضر**: الجلسة الأخيرة :

- تعلمنا 4 مفاتيح للأكل الصحي بالخارج.
- تحدثنا عن كيفية تطبيق المفاتيح في المواقف المختلفة.
- تدربنا على طلب ما نريده واختيار خيارات وجبات صحية واستبدال القائمة.
- وضعنا خطة عمل للأسبوع.

**اسأل:** هل جربت خطة العمل الخاصة بك لتناول الطعام في الخارج؟ أي متطوعين يريدون تبادل خبراتهم؟

**استجابات مفتوحة.**

**اسأل:** كيف تشعر هذا الأسبوع بشأن أهدافك وما نحن هنا لتحقيقه؟

**استجابات مفتوحة.**

**ناقش** أي عوائق ذكرها المشاركون وساعدهم في حل المشكلة.

**حاضر:** هذا الأسبوع سوف:

- نعرف كيف يمكن للأفكار السلبية أن تمنعنا من تحقيق أهدافنا المتمثلة في فقدان الوزن وزيادة النشاط البدني.
- نناقش بعض الطرق التي يمكننا من خلالها إيقاف الأفكار السلبية.
- نتدرب على وقف الأفكار السلبية والتحدث معها بأفكار إيجابية.

الجزء الثاني: ما هي الأفكار السلبية؟ (30 دقيقة)

**حاضر:** سنتحدث اليوم عن الأفكار السلبية.

**اسأل**: هل يستطيع أحد أن يشرح ما هي الأفكار السلبية؟

**استجابات مفتوحة.**

**حاضر:** الأفكار السلبية هي أفكار تجعلنا نفقد الأمل أو تمنعنا من المضي قدمًا. عادة ما يكون التفكير السلبي نقدًا لأنفسنا.

كل شخص لديه أفكار سلبية في بعض الأحيان. يمكن أن تقودنا الأفكار السلبية إلى الإفراط في تناول الطعام ، وتناول الأطعمة غير الصحية أو الى عدم النشاط. بعد أن نقوم بخيارات غير صحية ، نشعر بالإحباط عند الوصول إلى أهدافنا ، وبالتالي تتولد لدينا أفكار سلبية أكثر. عندما يؤدي التفكير السلبي إلى تصرفنا بطريقة غير صحية ويؤدي هذا السلوك بعد ذلك إلى فكرة سلبية أخرى ، ندخل في دائرة من هزيمة الذات.

على سبيل المثال ، افترض أنك عدت إلى المنزل بعد يوم عمل شاق أو لديك خلاف مع أحد أفراد عائلتك. أنت تفكر في نفسك ، "لقد سئمت من مشاهدة ما آكله طوال الوقت. ما الفرق الذي يحدثه؟ سأقوم فقط بتناول ما أريد ".

لذا تأكل كيسًا كاملاً من رقائق البطاطس. ثم تفكر ، "لقد فعلت ذلك مرة أخرى. لن أفقد الوزن أبدًا ". أنت الآن محبط وتناول كيسًا آخر من رقائق البطاطس.

**اسأل:** هل يمكنك التفكير في أي أمثلة أخرى لأفكار سلبية أو مهزومة؟

**استجابات مفتوحة.**

(قد يشارك بعض المشاركين أشياء خارج قدرتك على المساعدة. شجعهم على رؤية طبيبهم وإحالتهم إلى العيادة المناسبة.)

**حاضر**: في بعض الأحيان لا ندرك أن لدينا أفكارًا سلبية. يصبح التفكير السلبي عادة بالنسبة لمعظمنا بحيث نؤمن بأفكارنا السلبية ونتصرف بناءً عليها دون التفكير في طرق لتحديها.

الهدف من هذه الجلسة هو مساعدتك على أن تكون على دراية بأفكارك السلبية ، لتظهر لك كيف تقلل من فرصك في الوصول إلى أهدافك ، وتعليمك كيفية الرد على الأفكار السلبية بأفكار إيجابية.

**قم بإحالة** المشاركين إلى نشرة "الأفكار السلبية".

**حاضر:** يوضح المثال الأول في هذه النشرة حلقة من الأفكار السلبية المهزومة ذاتيًا. تحتوي النشرة أيضًا على بعض الأمثلة على أنواع مختلفة من الأفكار السلبية

فكر في وقت لم تحقق فيه شيئًا كنت تريد تحقيقه ووضعت نفسك في وضع صعب بسببه . فكر فيما إذا كانت أفكارك جعلت الوضع أفضل أم أسوأ.

**اسأل:** هل يرغب أي شخص في مشاركة تجربة عندما تعمل الأفكار السلبية ضد جهودك لتناول الطعام الصحي والنشاط؟

(كن مستعدًا لإعطاء مثال لكيفية عمل الأفكار السلبية ضدنا ، إذا لم يتحدث أحد.)

**ناقش** كل فئة من فئات التفكير السلبي.

أفكار جيدة أو سيئة

**حاضر:** النوع الأول من التفكير يسمى أحيانًا "التفكير الأسود أو الأبيض". يقسم هذا النوع من التفكير كل شيء إلى فئتين: جيد تمامًا وسيئًا تمامًا.

- الأطعمة الجيدة والسيئة.
- نجاح أو فشل.
- في البرنامج أو خارج البرنامج.

مثال: "انظر إلى ما فعلته. أكلت الكعكة. لن أنجح أبدًا في هذا البرنامج ".

**اسأل**:

هل تعتبر بعض الأطعمة "جيدة" والبعض الآخر "سيئة"؟

ماذا يحدث عندما تأكل قليلا مما تعتبره طعاما "سيئا"؟

هل يمكنك التفكير في سبب عدم اعتبار الطعام "سيئًا" تمامًا؟

**استجابات مفتوحة.**

(تأكد من عدم حظر الأطعمة في هذا البرنامج. يحتاج المشاركون إلى فهم أنه عندما لا توجد وجبة غير صحية واحدة أو عندما يمريوم واحد بدون نشاط بدني يعتبر كارثة .)

الأعذار

**حاضر**: مع الأعذار ، نلوم شيئا أو شخص آخر على مشاكلنا. نتصرف كما لو لم يكن لدينا خيار سوى الإفراط في تناول الطعام أو عدم النشاط بسبب هذا الشيء أو هذا الشخص. "لم أستطع المساعدة بسبب ..."

مثال: "يجب أن أشتري الكوكيز هذه في حالة حضور شخص ما".

**اسأل:**

هل يمكنك التفكير في وقت قمت فيه بشراء بعض الأطعمة عالية الدهون والسعرات الحرارية "لشخص آخر"؟

هل يحتاج هذا الشخص حقًا إلى الطعام ، أم أنك استخدمته كحجة لشراء الطعام لنفسك؟

**استجابات مفتوحة.**

(ساعدهم على معرفة كيف يمكن أن تؤدي الأعذار إلى هزيمة الذات).

أفكار "يجب"

**حاضر:** أفكار "ينبغي" تتوقع الكمال. ومع ذلك ، لا يوجد أحد مثالي. لذا ، فإن أفكار "ينبغي او يجب" يمكن أن تثير خيبة الأمل لدينا. يمكن أن تؤدي أيضًا إلى الغضب والإحباط ، لأننا نشعر أن شخصًا ما يحكم علينا ، مما يجبرنا على فعل ما لا نريد القيام به.

مثال: "كان يجب أن أتخطى الحلوى".

**اسأل:**

ما الذي يجب عليك فعله لفقدان الوزن وزيادة النشاط؟ ما الذي يجب ألا تفعله؟

ماذا يحدث عندما تتوقع أن تكون مثاليًا؟ ما هو شعورك؟ كيف تؤثر على القرارات والخيارات التي تتخذها؟

**استجابات مفتوحة.**

(الرسالة هي أننا لا نستطيع أن نتوقع أن نكون مثاليين. لذلك نحتاج إلى طرق للتغلب على الإحباط عندما نرتكب الأخطاء. يمكننا دائمًا اختيار المضي قدمًا والعمل بشكل أفضل.)

أفكار "ليست جيدة"

**حاضر:** بهذه الأفكار ، نقارن أنفسنا مع شخص آخر ، ثم نلوم أنفسنا إذا لم نفعل ذلك كما يفعلون.

مثال: "فقدت مريم كيلوغرامين هذا الأسبوع ، وفقدت كيلوغرام واحد فقط".

اسأل:

هل تقارن نفسك مع شخص آخر؟ مع من؟

كيف تؤثر على نفسك بهذا الشخص؟

كيف تجعلك المقارنة تشعر؟ كيف تؤثر على قراراتك واختياراتك حول الأكل والنشاط؟

(ربما يكون هذا أكثر حساسية للمناقشة. إذا كنت تشعر بالراحة ، شارك تجربتك الشخصية الخاصة بك مع المقارنة مع الآخرين. إذا أمكنك ، فقم بالإشارة أيضًا إلى أننا نحقق تقدمًا نحو الاختيارات الصحية ، مقارنة بيننا الآن شخصيًا بما نحن ما كنت تفعله من قبل جيد لأنه يشجعنا على الاستمرار.)

أفكار "التخلي"

**حاضر**: أفكار "الاستسلام" تهزم. غالبًا ما يتبعون الأنواع الأخرى من الأفكار السلبية.

مثال: "هذا البرنامج صعب للغاية. أنا لا أفقد الكثير من الوزن مثل مريم ، لذا قد أستسلم أيضًا ".

**اسأل:** هل تريد شيئًا يحتوي على نسبة عالية من الدهون أو السعرات الحرارية ، وفكر ، "لقد سئمت من هذا البرنامج"؟

**استجابات مفتوحة.**

**ملحوظة**: الرسالة الرئيسية هي أننا نعمل جميعًا في رحلتنا الخاصة طوال الحياة إلى السلوك الصحي. هذا البرنامج هو مجرد بداية تلك الرحلة. يذهب الجميع في هذه الرحلة بسرعات مختلفة. قد ترغب في الحصول على كيسين سعة 2.5 كيلوغرام من الطحين حتى يتمكن المشاركون من تحمل مقدار الوزن الذي فقدوه في أيديهم ويشعروا بثقله.

الجزء الثالث: الرد على الأفكار السلبية (20 دقيقة)

**حاضر**: لقد تحدثنا عن أنواع الأفكار السلبية التي يمكن أن تجعل من الصعب الاستمرار في التركيز على أهدافنا. لذا ، كيف يمكننا منع هذه الأفكار ، أو التخلص منها عند حدوثها؟

بمجرد أن تدرك فكرة سلبية ، يمكنك "الرد" عليها.

**اسأل:** ما رأيك ماذا يعني "الرد على" مرة أخرى إلى فكرة سلبية؟

**استجابات مفتوحة.**

**ارجع** إلى نشرة "الرجوع إلى الأفكار السلبية".

إليك كيفية الرد على الأفكار السلبية:

1. امسك نفسك بفكر سلبي. اسأل نفسك ، "هل هذه الفكرة تدفعني إلى الأمام نحو هدفي ، أم أنها تحبطني؟" بمجرد أن تدرك فكرة سلبية ، قل لنفسك ، "أنا سلبية تجاه نفسي."

2. تصوير يقول لنفسك ، "توقف!" تصور علامة توقف حمراء كبيرة تملأ عقلك. يجب أن يحل محل كل شيء آخر يحدث حتى تتمكن من التخلص من الفكر السلبي.

3. الآن وقد اختفى الفكر السلبي ، استبدلها بالعودة إلى التفكير الإيجابي. ما لم تملأ المكان الذي كان فيه التفكير السلبي في عقلك ، فمن المحتمل أن يعود مرة أخرى في موقف مماثل لأنه مع مرور الوقت أصبح عادة. هذا هو السبب في أهمية بناء عادة جديدة. قم ببناء عادة جديدة بأفكار إيجابية ، وليس أفكارًا سلبية.

**ملاحظة:** قد يكون من المفيد أن يكون لديك علامة STOP عند مناقشة الخطوة 2.

**استعرض** بإيجاز الطرق المختلفة للتحدث مرة أخرى الموجودة في النشرة.

- جيد أو سيئ: تحدث مجددًا عن هذه الأفكار السلبية باستخدام "العمل من أجل التوازن: لا تتوقع الكمال من نفسك ، ولكن ابق متحكمًا. اعمل على تحقيق توازن شامل.
- الأعذار: رد على الأعذار بعبارة "الأمر يستحق المحاولة". بدلًا من البحث عن شيء أو شخص آخر لإلقاء اللوم ، امنح نفسك فرصة - فقد تنجح.
- ينبغي: التحدث إلى عبارات "يجب" بعبارة "إنه خياري". أنت مسؤول عن الأكل والنشاط. لا أحد غيره. لا أحد يقف عليك بتوقعات غير واقعية.
- ليست جيدة مثل: تحدث مرة أخرى مع أفكار المقارنة هذه مع "الجميع مختلفون. سأفعل ما هو جيد بالنسبة لي ".
- استسلم: رد على الأفكار المهزومة ذاتيًا بـ "خطوة واحدة في كل مرة". يستغرق الأمر بعض الوقت لإجراء تغييرات مدى الحياة ، وقد يستغرق عدة محاولات. إذا لم ينجح شيء ما ، فجرّب طريقة أخرى. دائمًا ما يكون تعلم أي شيء ناجحًا ، حتى إذا كان كل ما نتعلمه هو شيء لا يعمل.

**حاضر**: دعونا نتدرب الآن على وقف الأفكار السلبية واستبدالها بالتحدث إليهم بأفكار إيجابية. خذ لحظة وانظر إلى أنواع الأفكار السلبية التي ناقشناها.

**ارجع** إلى نشرة "تدرب على التحدث مرة أخرى".

**اسأل:** هل تعرف بعض طرق التفكير هذه؟ ما أنواع هذه الأفكار الأكثر شيوعًا بالنسبة لك؟ هل أنت أكثر من مفكر "عذر" ، أو "لست جيدة مثل ____"؟

(إذا كانت المجموعة كبيرة ، يمكنك تقسيمها إلى مجموعات أصغر وجعل كل مشارك يأخذ دوره في القيام بالنشاط. من المهم أن يقوم كل مشارك في الواقع بالتمرين. "

**تعليمات للمشاركين:**

1. **اكتب** مثالاً لكل نوع من أنواع التفكير السلبي من حياتك الخاصة. إذا لم تتمكن من التفكير في أي مشاركة أو لم تكن مرتاحًا ، فاستخدم أحد الأمثلة في النشرات.

2. **قل** أول فكرة سلبية في قائمتك بصوت عال.

3. **قل** "توقف!"

4. **تحدث** مرة أخرى إلى التفكير السلبي بصوت عالٍ مع التفكير الإيجابي.

5. ا**كتب** الفكرة الإيجابية في مذكرتك.

(قد يكون من المفيد معرفة ما إذا كان المشاركون يتذكرون ما كتبوه في نشرة "تذكر هدفك" في الجلسة الأولى حيث كتبوا أسباب الانضمام إلى البرنامج. وقد يساعدهم ذلك على تكوين أفكار إيجابية.)

الجزء الرابع: ختام وقائمة المهام (5 دقائق)

**قم بإحالة** المشاركين إلى نشرة "المهام الأسبوع المقبل".

**حاضر:** في الأسبوع القادم ، أكمل العناصر الموجودة في هذه النشرة:

- تتبع الأكل والنشاط.
- سيطر على نفسك بخصوص الأفكار السلبية. قم بتدوينهم هذا الأسبوع ، بالإضافة إلى كيفية "الرد" عليهم. تدرب على وقف الأفكار السلبية عندما تصبح على دراية بها ، وتحدث معها لاستبدالها بأفكار إيجابية.

**لخص** هذه النقاط الرئيسية:

- غالبًا ما تجعل الأفكار السلبية من الصعب الاستمرار في التركيز على الأهداف الصحية.
- يمكنك إيقاف الأفكار السلبية عن طريق التحدث مرة أخرى باستخدام الأفكار الإيجابية.
- تدرب على الرد على الأفكار السلبية باستخدام الأفكار والصور التي تناسبك.

**حاضر:**

كانت هذه جلسة مهمة وشخصية طُلب منك فيها العثور على الأفكار السلبية التي تتحدىك عندما تعمل على إجراء تغييرات صحية والتحدث عنها. في الأسبوع المقبل ، سنلقي نظرة على كيفية حدوث الزلات ، والأهم من ذلك ، كيف نعود إلى طريقنا إلى هدفنا عند حدوث الزلات او الاخطاء.

**اسأل** المشاركين إذا كان لديهم أي أسئلة.

**بعد الجلسة:**

**راجِع** متتبعي الطعام والنشاط ودوِّن ملاحظات.
