# Supplementary material for: Emirates Heart Health Project (EHHP): A protocol for a stepped-wedge family-cluster randomized-controlled trial of a health-coach guided diet and exercise intervention to reduce weight and cardiovascular risk in overweight and obese UAE nationals
Source: PLoS One. 2023 Apr 10;18(4):e0282502. doi: 10.1371/journal.pone.0282502 (PMC10085020; doi:10.1371/journal.pone.0282502)
Supplement: S27 Appendix — (DOCX) [file pone.0282502.s027.docx]

**Session 12: The slippery slope of lifestyle change**

**Learning objectives**

At the close of this session, the participants will be able to:

- Describe their personal current progress toward defined goals.
- Describe common causes for slipping from healthy eating or being active.
- Explain what to do to get back on their feet after a slip.

**Materials**

- Before the session, prepare the graphs of how each participant’s weight and physical activity have changed over time.
- Participant handouts
  - Session 12 overview
  - Progress review
  - The slippery slope of lifestyle change
  - After a slip
  - Slips from healthy eating: Action plan
  - Slips from being active: Action plan
  - To do next week
- Food and Activity Trackers for Session 12
- Whiteboard and markers

**Session 12 overview**

We move from recognizing and talking back to negative thoughts to the bigger problem of coping with slips from healthy eating and physical activity. This session helps participants 1) to keep a positive perspective on their efforts to make long-term lifestyle changes and 2) to understand that setbacks are common.

Session 12 is divided into four parts:

*Part 1: Weekly progress and review (5 minutes)*

Discuss how well participants did in achieving their goals for the past week. Ask participants to share their experiences in replacing negative thoughts with positive thoughts.

*Part 2: Checking your progress (20 minutes)*

Today is the first time the participants analyze their progress since the early sessions. Ask participants to share their individual progress. How are they doing? Are they progressing? Individual experiences often are also experienced by others in the group. The realization that others have similar experiences and challenges usually leads to valuable group discussion.

*Part 3: Slips in progress (30 minutes)*

Discuss why we slip and go over what to do after a slip to get back on track.

*Part 4: Wrap up and to do list (5 minutes)*

**Key messages**

- **Slipping off the path of healthy eating and adequate physical activity is natural, normal and expected. You can learn from slips.**
- **When you slip, don’t give up. Keep a positive attitude, and regain control as soon as possible. Remember slipping is not the end of your hopes for a healthy lifestyle.**
- **Slips are opportunities to learn about cues that lead to slips and to understand the negative or self-defeating thoughts we have after a slip.**
- **When you slip, talk back to negative thoughts with positive thoughts.**

*Part 1: Weekly progress and review (5 minutes)*

**Distribute**

- Session 12 handouts
- Session 12 “Food and Activity Trackers”
- Session 10 “Food and Activity Trackers” with your notes

**Collect** Session 11 “Food and Activity Trackers”

**Ask:** Did you have any trouble keeping track last week? Were you able to stay within your fat gram and calorie budgets? Did you reach your goal for physical activity?

**Open responses.**

**Praise all progress**, no matter how small.

**Present:** This week we will:

- Take a look at your progress so far and make adjustments if needed.
- Talk about some common causes for slipping off the path of healthy eating or being active.
- Discuss how to get back on your feet after a slip.

*Part 2: Checking your progress (20 minutes)*

**Present:** Today we are going to talk about slips: times when you do not follow your plan for healthy eating or being active. However, before we talk about that, let’s review your progress since Session 7, which was the last time we checked on your profess. We will look at how each of you are progressing toward your personal goals, and I’ll help you improve your progress if needed.

**Ask** for volunteers to share personal challenges and successes during the past 4-5 weeks.

**Encourage** group discussion.

**Present:** What steps did you take to overcome the challenges? What were the reasons for your successes?

**Open responses.**

**Refer** to the “Progress review” handout, and the “How am I doing?” charts (that you prepared in advance for each participant) for weight and physical activity.

**Distribute** each participants’ progress graph.

**Present:** Let’s take a look at how you are doing. Remember that you are on your own personal journey. Don’t let the negative thought of “Not as good as” come into your mind.

**Ask** the participants the following questions about progress:

Activity: What major changes did you make to be more active? Include both your activities like walking for 10-30 minutes as well as lifestyle changes that you did not record, like going to get something yourself instead of asking your maid, parking farther and walking, etc.

Food: What changes did you make to eat less fat and fewer calories?

Look at your “How am I doing?” chart. Have you reached your goal weight? Your activity goal?

(Congratulate those who are on track or at goal, and praise those who are making progress. Encourage those who are having difficulty to develop a plan to make better progress, and to write this plan down.)

*Part 3: Slips in progress (30 minutes)*

**Present:** Now let’s move on to our topic for today: slips.

Slips are times when we do not follow our plans for healthy eating or physical activity. Slips are:

- **A normal part of lifestyle change.** When we are learning to walk, we fall. That is normal. Even as adults now, from time to time we may slip and fall or trip and fall. And everyone who sets out to lose weight, eat in a healthy way, and be more physically active will slip.
- **To be expected.** Because it is a normal part of change, we should expect slips. If you have not already had some slips, you will most likely have them in the future.

Does this sound discouraging? Well, it does not have to be, because slips do not have to hurt your progress. What can hurt your progress is the way you respond to a slip, so today, we will talk about the best way to respond to slips so they do not hurt your progress.

Let’s use walking as an example.

Everyone learning to walk will slip and fall. It is a natural part of learning to walk. Parents, uncles, cousins of babies know the baby will fall and show them how to get up and try again. That’s what we will do today, talk about when you slip from your healthy eating and physical activity plans and how you can get back up again.

**Present:** Everyone has different reasons for slipping. Earlier, we talked about many problem cues for eating unhealthy foods and being inactive. Moods or feelings may cause us to slip. Some of us eat too much when we are happy.

Imagine that your family is celebrating a holiday, a birthday, or a vacation. There is plenty of everyone’s favorite food, from appetizers to desserts. For years, it is your family habit to take it east, have fin and relax during these times.

**Ask:** What would this situation be like for you? Would you tend to slip off the path to healthy eating and activity in this kind of situation?

**Open responses.**

**Present:** Some of us are more likely to slip when we are bored.

Imagine you are at home alone, watching television. You are feeling okay, pretty relaxed, but nothing you want to watch is on television. No one is posting on Snapchat or Instagram. What do you do?

**Ask:** Do you find yourself in the kitchen, looking for a snack?

**Open responses.**

**Present:** Some people overeat when they are upset.

Imagine you are having a relaxing evening at home. A family member brings up a topic that is upsetting to both of you. You both get angry, and they leave the room angrily and slams their door. What do you do?

**Open responses.**

**Present:** You’re behind on a project at work. The manager looks in on you every 10 minutes, and they are clearly upset. You feel pressured and tense. Someone has brought in some delicious cookies and cake this morning.

**Ask:** What would this situation be like for you?

**Open responses.**

**Ask:** Which one of these examples is the most difficult for you regarding your ability to stay on the path of healthy eating: When you are happy, bored, upset or worried?

**Open responses.**

**Refer** to “The slippery slope of lifestyle change” handout.

**Present:** On your handout, write down the things and situations that cause you to slip from healthy eating and physical activity.

**Ask** for volunteers to share what they wrote.

(If no one wants to share, ask whether anyone is more likely to slip at certain times: traveling, at parties, in certain moods, with cold weather.)

**Present:** Slips are learned habits that come from responding to a certain cue in the same way over and over in our past. For example, at a party, one person may be so busy talking and laughing that she forgets to eat, but someone else is focused on eating the foods that are available.

After you slip, here are 5 things to do:

1. **Talk back to negative thoughts with positive thoughts.**
2. **Ask yourself what happened.**
3. **Regain control as soon as you can.**
4. **Talk to someone supportive.**
5. **Focus on all the positive changes you made, and realize that you can get back on track.**
6. **Talk back to negative thoughts with positive thoughts.** Negative thoughts after a slip are your worst enemy. They can leave you feeling discouraged, guilty and angry. They can make you think that you cannot handle the slip. Remember how we replace negative thoughts with positive thoughts: “I am not a failure because I slipped. Everyone slips. I will get back on my feet again.”
7. **Ask yourself what happened.** Look closely at the situation and ask yourself what happened. Remember the chain of events we talked about in an earlier session. Was it is special occasion, or something that happens repeatedly? Did you feel lonely, bored, happy, worried? Was it social pressure? Did you skip physical activity because of work or family pressures?
8. **Regain control as soon as you can.** Don’t wait for the next day. Make your next meal a healthy one. Get back to being active right away. You will not have lost much progress if you follow this suggestion.
9. **Talk to someone supportive.** Talk to someone in your family, or to me. Discuss your new strategy for handling slips. Commit yourself to getting up and continuing to your goal.
10. **Focus on all the positive changes you made, and realize that you can get back on track.** If you are person who slipped today, you are the same person who was successful in the past few weeks. Slips are not who you are, they are simply a behavior that can be changed.

**Present:** Here are some helpful ideas about slips.

- **You can learn from a slip.** You have the opportunity to learn and then plan how to handle a similar situation in a better way next time.
  - **If you slip, take a few moments to think about how you can avoid similar situations.**
  - **If you cannot avoid a situation, think about how you can manage it in a better way.**
- **Remember you are making life-long changes. Slips are just a normal part of the process.**

**Present:** Earlier, we said that what causes us to slip is a habit. And the way we react to slips is also a habit. You can learn a new way and then create new habits that will get you back on your feet again and moving toward your goal.

Remember two things:

1. **Slips are normal and to be expected.** Everyone who is on the way to losing weight and being more active has slips. But just because you have slips doesn’t mean you give up completely. Slips teach us how we can do better.
2. **No one time of overeating or being inactive, no matter how severe, will ruin everything**. You will not gain more than a few kilos of weight even after the biggest slip possible- unless you choose to stay off the path and keep on overeating. The slip is not the problem. The only problem is if you do not get up to your feet again and keep moving toward your goals.

*Part 4: Wrap up and to do list (5 minutes)*

**Present:** Now let’s create an action plan for 1) how you will recover from slips toward your goals and 2) how to avoid having slips in the first place.

**Refer** to the “Slips from healthy eating: Action plan” and “Slips from being active: Action plan” handouts.

**Complete the handouts.**

1. Describe one thing that caused you to slip from healthy eating. Can you avoid it in the future? If so, how? If you cannot avoid it, how will you get back to your feet after?
2. Describe one thing that caused you to slip from being active. Can you avoid it in the future? If so, how? If you cannot avoid it, how will you get back to your feet after?

**Refer** to the “To do next week” handout.

**Present:** For next week:

Keep track of your eating and activity.

Try your two action plans; avoid the situation that caused you to slip before, and if you slip, use your plan to get back on your feet.

**Summarize key points:**

- **Slips are a normal part of changing behavior.**
- **When you slip, it is important to change something. Avoid situations that cause you to slip, and if you can’t avoid it, change your response to the situation, and if you slip, get up and keep moving toward your goal.**
- **Slipping is not failure. Learn from your slip. Figure out what you need to do to get back on track and keep moving forward.**

**Close:** As you begin your week, consider the strategies we discussed about slipping. Do not worry about slips; slips happen. What’s important is to regain your focus- remember what you’re trying to do and why. Get back on your feet. Do your best.

**Ask** if there are any questions.

**After the session:** Make your usual notes and recommendations for each participant.
