# Supplementary material for: Emirates Heart Health Project (EHHP): A protocol for a stepped-wedge family-cluster randomized-controlled trial of a health-coach guided diet and exercise intervention to reduce weight and cardiovascular risk in overweight and obese UAE nationals
Source: PLoS One. 2023 Apr 10;18(4):e0282502. doi: 10.1371/journal.pone.0282502 (PMC10085020; doi:10.1371/journal.pone.0282502)
Supplement: S28 Appendix — (DOCX) [file pone.0282502.s028.docx]

**الجلسة 12: المنحدر الزلق لتغيير نمط الحياة**

**أهداف التعلم**

في ختام هذه الجلسة سيتمكن المشاركون من:

- وصف تقدمهم الشخصي الحالي نحو أهداف محددة.
- وصف الأسباب الشائعة للانزلاق من الأكل الصحي أو النشاط.
- شرح ما يجب القيام به للوقوف على أقدامهم بعد الانزلاق.

**المواد**

- قبل الجلسة ، قم بإعداد الرسوم البيانية لكيفية تغير وزن كل مشارك ونشاطه البدني بمرور الوقت.
- منشورات المشاركين
  - نظرة عامة على الجلسة 12
  - مراجعة التقدم
  - المنحدر الزلق لتغيير نمط الحياة
  - بعد الانزلاق
  - زلات من الأكل الصحي: خطة العمل
  - زلات من كونك نشطا: خطة العمل
  - مهام الأسبوع المقبل
- متتبعي الطعام والنشاط للجلسة 12
- السبورة والأقلام

**نظرة عامة للجلسة 12**

ننتقل من التعرف على الأفكار السلبية والتحدث عنها إلى المشكلة الأكبر في التعامل مع الانزلاقات أو الزلات من الأكل الصحي والنشاط البدني. تساعد هذه الجلسة المشاركين 1) على الحفاظ على منظور إيجابي حول جهودهم لإجراء تغييرات طويلة المدى في نمط الحياة و 2) لفهم أن الانتكاسات شائعة.

تنقسم الجلسة 12 إلى أربعة أجزاء:

الجزء الأول: التقدم والمراجعة الأسبوعية (5 دقائق)

ناقش مدى نجاح المشاركين في تحقيق أهدافهم في الأسبوع الماضي. اطلب من المشاركين تبادل تجاربهم في استبدال الأفكار السلبية بأفكار إيجابية.

الجزء الثاني: التحقق من تقدمك (20 دقيقة)

اليوم هي المرة الأولى التي يحلل فيها المشاركون تقدمهم منذ الجلسات الأولى. اطلب من المشاركين مشاركة تقدمهم الفردي. كيف أداءهم ؟ هل هنالك تقدم؟ غالبًا ما مر الاخرون في المجموعة بنفس التجارب . عادة ما يؤدي إدراك أن لدى الآخرين تجارب وتحديات مماثلة إلى مناقشة جماعية قيمة.

الجزء 3: الزلات قيد التقدم او التنفيذ (30 دقيقة)

ناقش لماذا ننزلق واستعرض ما يجب فعله بعد الانزلاق للعودة إلى المسار الصحيح.

الجزء 4: الختام وقائمة المهام (5 دقائق)

**الرسائل الرئيسية**

- إن الانزلاق عن مسار الأكل الصحي الصحيح والنشاط البدني الكافي أمر طبيعي ومتوقع. يمكنك التعلم من الزلات.
- عندما تنزلق ، لا تستسلم. حافظ على الموقف الإيجابي واستعد السيطرة في أقرب وقت ممكن. تذكر أن الانزلاق ليس نهاية آمالك في أسلوب حياة صحي.
- الزلات هي فرص للتعرف على الإشارات التي تؤدي إلى الزلات وفهم الأفكار السلبية أو المهزومة ذاتيا التي لدينا بعد الانزلاق.
- عندما تنزلق ، بدل الأفكار السلبية بأفكار إيجابية.

**الجزء الأول: التقدم والمراجعة الأسبوعية (5 دقائق)**

**وزع**

- نشرات الجلسة 12
- "متتبعو الطعام والنشاط"للجلسة 12
- "متتبعو الطعام والنشاط"الجلسة 10 مع ملاحظاتك عليها
- اجمع"متتبعو الطعام والنشاط" للجلسة 11

**اسأل:** هل واجهتك أي مشكلة في متابعة مسارك الأسبوع الماضي؟ هل كنت قادرًا على البقاء ضمن حدك من الدهون والسعرات الحرارية؟ هل وصلت إلى هدفك للنشاط البدني؟

**ردود مفتوحة**

**امدح** كل تقدم مهما كان صغيرًا.

**حاضر:** هذا الأسبوع سوف:

- نلق نظرة على تقدمك حتى الآن ونقم بإجراء تعديلات إذا لزم الأمر.
- نتحدث عن بعض الأسباب الشائعة للانزلاق عن مسار الأكل الصحي أو النشاط.
- نناقش كيفيةا لرجوع بعد الانزلاق.

**الجزء الثاني: التحقق من تقدمك (20 دقيقة)**

**حاضر**: سنتحدث اليوم عن الزلات: الأوقات التي لا تتبع فيها خطتك لتناول طعام صحي أو تكون نشطًا. ومع ذلك ، قبل أن نتحدث عن ذلك ، دعنا نراجع تقدمك منذ الجلسة 7 ، وهي المرة الأخيرة التي قمنا فيها بالتحقق من تقدمك. سنلقي نظرة على كيفية تقدم كل واحد منكم نحو أهدافه الشخصية ، وسأساعدكم على تحسين تقدمكم إذا لزم الأمر.

**اطلب** متطوعين لمشاركة التحديات والنجاحات الشخصية خلال الأسابيع 4-5 الماضية.

**شجع** المناقشة الجماعية.

**حاضر:** ما هي الخطوات التي اتخذتها للتغلب على التحديات؟ ما هي أسباب نجاحك؟

**ردود مفتوحة**

**ارجع** إلى نشرة "مراجعة التقدم" و "كيف حالى؟" الرسوم البيانية (التي قمت بإعدادها مسبقًا لكل مشارك) للوزن والنشاط البدني.

**وزع** الرسم البياني للتقدم لكل مشارك.

**تقديم**: دعنا نلقي نظرة على حالتك. تذكر أنك في رحلتك الشخصية. لا تدع الفكر السلبي لـ " لست جيدا " يخطر على بالك.

**اطرح** على المشاركين الأسئلة التالية حول التقدم:

**النشاط**: ما التغييرات الرئيسية التي قمت بها لتكون أكثر نشاطًا؟ قم بتضمين كل من أنشطتك مثل المشي لمدة 10-30 دقيقة بالإضافة إلى التغييرات في نمط الحياة التي لم تسجلها ، مثل الذهاب لاحضار شيء بنفسك بدلاً من طلب الخادمة ، والوقوف في مكان أبعد والمشي ، إلخ.

**الطعام:** ما هي التغييرات التي أجريتها على تناول كمية أقل من الدهون والسعرات الحرارية؟

انظر إلى مخطط "كيف حالي؟". هل وصلت الوزن المطلوب؟ هل وصلت الى هدفك في النشاط البدني؟

(قم بتهنئة أولئك الذين يسيرون على الطريق الصحيح أو في الهدف ، والثناء على أولئك الذين يحرزون التقدم. شجع أولئك الذين يجدون صعوبة في تطوير خطة لتحقيق تقدم أفضل ، وكتابة هذه الخطة.)

**الجزء 3: الانزلاق قيد التنفيذ (30 دقيقة)**

**حاضر**: دعنا ننتقل الآن إلى موضوعنا اليوم: الانزلاقات.

الزلات هي أوقات لا نتبع فيها خططنا لتناول الطعام الصحي أو النشاط البدني. الزلات هي:

- **جزء طبيعي من تغيير نمط الحياة**. عندما نتعلم المشي ، نسقط. هذا طبيعي. حتى كبالغين الآن ، من وقت لآخر قد ننزلق ونهبط أو نسقط. وكل شخص يخطط لإنقاص الوزن ، ويأكل بطريقة صحية ، وأن يكون أكثر نشاطًا بدنيًا يمكن أن ينزلق.
- **من المتوقع**. لأنه جزء طبيعي من التغيير ، يجب أن نتوقع زلات. إذا لم تكن قد حصلت بالفعل ، فمن المرجح أن تحصل في المستقبل.

هل هذا الصوت محبط؟ حسنًا ، لا يجب أن يكون الأمر كذلك ، لأن الزلات لا يجب أن تؤذي تقدمك. ما يمكن أن يضر تقدمك هو الطريقة التي تستجيب بها لها ، لذا سنتحدث اليوم عن أفضل طريقة للتخلص من الزلات حتى لا تضر تقدمك.

دعنا نستخدم المشي كمثال.

كل شخص يتعلم المشي سوف ينزلق ويسقط. إنه جزء طبيعي من تعلم المشي. الآباء والأعمام وأبناء العمومة يعرفون أن الطفل سيسقط ويروهم كيفية النهوض والمحاولة مرة أخرى. هذا ما سنفعله اليوم و نتحدث عنه ، عندما تنزلق من خطط الأكل الصحي والنشاط البدني وكيف يمكنك العودة مرة أخرى.

**حاضر:** لكل شخص أسباب مختلفة للانزلاق. في وقت سابق ، تحدثنا عن العديد من الإشارات حول تناول الأطعمة غير الصحية وعدم النشاط. المزاج أو المشاعر قد تجعلنا ننزلق. البعض منا يأكل كثيرا عندما يكون سعيدا.

تخيل أن عائلتك تحتفل بعطلة أو عيد ميلاد. هناك الكثير من الأطعمة المفضلة للجميع ، من المقبلات إلى الحلويات. سنوات ، من عادة عائلتك أن تأخذ الأمر بسهولة ، وأن تستمتع بالراحة والاسترخاء خلال هذه الأوقات.

**اسأل:** كيف سيكون هذا الموقف بالنسبة لك؟ هل تميل إلى الانزلاق عن طريق الأكل الصحي والنشاط في هذا النوع من الحالات؟

**حاضر:** من المرجح أن ينزلق البعض منا عندما يشعر بالملل.

تخيل أنك في المنزل وحدك ، تشاهد التلفاز. تشعر أنك بخير ، مرتاح جدًا ، ولكن لا شيء تريد مشاهدته على التلفزيون. لا أحد ينشر على سناب شات أو انستقرام ماذا تفعل؟

**اسأل:** هل تجد نفسك في المطبخ وتبحث عن وجبة خفيفة؟

**حاضر**: بعض الناس يفرطون في تناول الطعام عندما يكونوا منزعجين.

تخيل أنك تقضي أمسية مريحة في المنزل. يطرح أحد أفراد الأسرة موضوعًا مزعجًا لكليكما. كلاكما تغضبان ، وتغادرا الغرفة بغضب . ماذا تفعل؟

**ردود مفتوحة**

**حاضر**: أنت متأخر في تسليم مشروع في العمل. ينظر المدير إليك كل 10 دقائق ، ومن الواضح أنه مستاء. تشعر بالضغط والتوتر. أحضر شخص ما بعض الكعك والكوكيز اللذيذ هذا الصباح.

**اسأل:** كيف سيكون هذا الموقف بالنسبة لك؟

**ردود مفتوحة**

**اسأل**: أي من هذه الأمثلة هو الأصعب بالنسبة لك فيما يتعلق بقدرتك على البقاء على طريق الأكل الصحي: عندما تكون سعيدًا أو تشعر بالملل أو الانزعاج أو القلق؟

**ردود مفتوحة**

**ارجع** إلى نشرة "المنحدر الزلق لتغيير نمط الحياة".

**حاضر:**  اكتب الأشياء والمواقف التي تجعلك تنزلق من الأكل الصحي والنشاط البدني.

**اطلب** من المتطوعين مشاركة ما كتبوه.

(إذا لم يرغب أحد في المشاركة ، اسأل ما إذا كان أي شخص أكثر عرضة للانزلاق في أوقات معينة: السفر ، في الحفلات ، في بعض الحالات ، مع الطقس البارد.)

**حاضر:** الزلات هي عادات متعلمة تأتي من الاستجابة لإشارة معينة بنفس الطريقة مرارًا وتكرارًا في ماضينا. على سبيل المثال ، في إحدى الحفلات ، قد يكون أحد الأشخاص مشغولاً للغاية في التحدث والضحك لدرجة أنه ينسى تناول الطعام ، لكن شخصًا آخر يركز على تناول الأطعمة المتوفرة.

**بعد الانزلاق ، إليك 5 أشياء للقيام بها:**

1. تحويل الأفكار السلبية بأفكار إيجابية.
2. اسأل نفسك عما حدث.
3. استعادة السيطرة بأسرع ما يمكن.
4. تحدث إلى شخص داعم.
5. ركز على كل التغييرات الإيجابية التي قمت بها ، وادرك أنه يمكنك العودة إلى المسار الصحيح.

1) رد على الأفكار السلبية بأفكار إيجابية. الأفكار السلبية بعد الانزلاق هي أسوأ عدو لك. يمكنها تركك تشعر بالإحباط ، بالذنب والغضب. يمكنها جعلك تعتقد أنه لا يمكنك التعامل مع الانزلاق. تذكر كيف نستبدل الأفكار السلبية بأفكار إيجابية: "أنا لست فاشلاً لأنني انزلقت. الجميع ينزلق. سأعود على قدمي مرة أخرى ".

2) اسأل نفسك عما حدث. انظر عن كثب إلى الموقف واسأل نفسك عما حدث. تذكر سلسلة الأحداث التي تحدثنا عنها في جلسة سابقة. هل كانت مناسبة خاصة ، أم حدث شيء ما بشكل متكرر؟ هل شعرت بالوحدة والملل والسعادة والقلق؟ هل كان الضغط الاجتماعي؟ هل لم تمارس النشاط البدني بسبب ضغوط العمل أو الأسرة؟

3) استعادة السيطرة بأسرع ما يمكن. لا تنتظر اليوم التالي. اجعل وجبتك التالية صحية. عُد إلى نشاطك على الفور. لن تفقد الكثير من التقدم إذا اتبعت هذا الاقتراح.

4) تحدث إلى شخص داعم. تحدث مع شخص في عائلتك أو معي. ناقش استراتيجيتك الجديدة للتعامل مع الزلات ألزم نفسك بالاستيقاظ والاستمرار في تحقيق هدفك.

5) ركز على جميع التغييرات الإيجابية التي أجريتها ، وندرك أنه يمكنك العودة إلى المسار الصحيح. إذا كنت الشخص الذي تراجع اليوم ، فأنت الشخص نفسه الذي نجح في الأسابيع القليلة الماضية. الزلاتهي ليست ما أنت عليه ، إنها ببساطة سلوك يمكن تغييره.

**حاضر:** إليك بعض الأفكار المفيدة حول الزلات.

- **يمكنك التعلم من الخطأ. لديك الفرصة للتعلم** ثم تخطط لكيفية التعامل مع موقف مماثل بطريقة أفضل في المرة القادمة.
  - **إذا انزلقت ، خذ بضع دقائق للتفكير في كيفية تجنب المواقف المماثلة.**
  - **إذا لم تتمكن من تجنب الموقف ، فكر في كيفية إدارته بطريقة أفضل.**
- **تذكر أنك تقوم بإجراء تغييرات مدى الحياة. الزلات ليست سوى جزء طبيعي من العملية.**

**حاضر**: قلنا في وقت سابق أن ما يجعلنا ننزلق هو عادة. والطريقة التي نتفاعل بها مع الزلات هي أيضًا عادة. يمكنك تعلم طريقة جديدة ثم إنشاء عادات جديدة تعيدك إلى قدميك مرة أخرى وتتجه نحو هدفك.

تذكر شيئين:

1. **الزلات أمر طبيعي ومتوقع**. كل شخص في طريقه إلى فقدان الوزن وزيادة النشاط لديه زلات. ولكن لمجرد أن لديك زلات لا يعني أنك تستسلم تمامًا. تعلمنا أخطائنا كيف يمكننا أن نفعل الأفضل.
2. **مرة واحدة من الإفراط في تناول الطعام أو عدم النشاط ، مهما كانت شديدة ، لم تدمر كل شيء**. لن تكسب أكثر من بضعة كيلوغرامات من الوزن حتى بعد أكبر زلة ممكنة - ما لم تختر البقاء بعيدًا عن المسار والاستمرار في الإفراط في تناول الطعام. الزلة ليست المشكلة. المشكلة الوحيدة هي أنك إذا لم تقف على قدميك مرة أخرى وواصلت التقدم نحو أهدافك.

الجزء 4: الختام وقائمة المهام (5 دقائق)

قدم: الآن دعنا ننشئ خطة عمل لـ 1) كيف ستتعافى من الانزلاق نحو أهدافك و 2) كيفية تجنب وجود الانزلاق في المقام الأول.

ارجع إلى نشرات "الزلات من الأكل الصحي: خطة العمل" و "الزلات من النشاط: خطة العمل".

**أكمل النشرات.**

1. صف شيئًا واحدًا جعلك تنزلق من الأكل الصحي. هل يمكنك تجنب ذلك في المستقبل؟ إذا كان الأمر كذلك ، فكيف؟ إذا كنت لا تستطيع تجنب ذلك ، كيف ستعود على قدميك بعد ذلك؟

2. صف شيئًا واحدًا جعلك تنزلق من النشاط. هل يمكنك تجنب ذلك في المستقبل؟ إذا كان الأمر كذلك ، فكيف؟ إذا كنت لا تستطيع تجنب ذلك ، كيف ستعود إلى قدميك بعد ذلك؟

**راجع** نشرة "المهام للأسبوع المقبل".

**حاضر**: للأسبوع القادم:

- تتبع الأكل والنشاط الخاص بك.
- جرب خطة العمل الخاصة بك ؛ تجنب الموقف الذي تسبب في انزلاقك من قبل ، وإذا انزلقت ، استخدم خطتك للوقوف على قدميك.

**تلخيص النقاط الرئيسية:**

- الزلات جزء طبيعي من تغيير السلوك.
- عندما تنزلق ، من المهم تغيير شيء ما. تجنب المواقف التي تجعلك تنزلق ، وإذا لم تتمكن من تجنبها ، فغير ردك على الموقف ، وإذا انزلق ، قم واستمر في التحرك نحو هدفك.
- الانزلاق ليس الفشل. تعلم من قسيمة الخاص بك. اكتشف ما عليك القيام به للعودة إلى المسار الصحيح ومواصلة المضي قدمًا.

**الخاتمة:** عندما تبدأ أسبوعك ، فكر في الاستراتيجيات التي ناقشناها حول الانزلاق. لا تقلق بشأن الزلات. تحدث زلات. المهم هو استعادة تركيزك - تذكر ما تحاول القيام به ولماذا. ارجع اقف على قدميك. ابذل قصارى جهدك.

أسأل ما إذا كان هناك أي أسئلة.

**بعد الجلسة:** اكتب ملاحظاتك وتوصياتك المعتادة لكل مشارك.
