# Supplementary material for: Emirates Heart Health Project (EHHP): A protocol for a stepped-wedge family-cluster randomized-controlled trial of a health-coach guided diet and exercise intervention to reduce weight and cardiovascular risk in overweight and obese UAE nationals
Source: PLoS One. 2023 Apr 10;18(4):e0282502. doi: 10.1371/journal.pone.0282502 (PMC10085020; doi:10.1371/journal.pone.0282502)
Supplement: S29 Appendix — (DOCX) [file pone.0282502.s029.docx]

**Session 13: Jump start your activity plan**


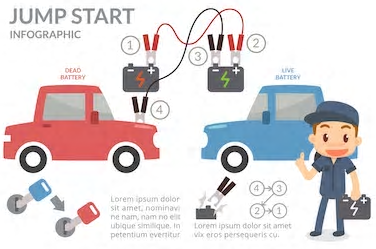


**Learning objectives**

- **Describe ways to add interest and variety to their activity plans.**
- **Define “aerobic fitness.”**
- **Explain the four F.I.T.T principles (frequency, intensity, time, and type of activity) and how they relate to aerobic fitness.**

**Materials**

- Handouts for Session 13:
  - Session 13: Overview
  - Ways to prevent boredom
  - Improving your aerobic fitness
  - FITT principles
  - How hard are you working?
  - To do next week
- Food and Activity Trackers for Session 13
- Whiteboard and markers

**Overview**

Boredom is an important reason people do not continue in a physical activity routine. Research by Dr. Juma Al Kaabi at UAEU showed that more than 20% of Emiratis said this was the main reason they did not exercise more. When an activity plan becomes routine, one may lose interest and fall back to old habits of being inactive. To make physical activity more enjoyable, participants will generate ideas for how to add interest and variety to their activity routine. You will also suggest ways to eliminate boredom and increase aerobic fitness.

Session 13 is divided into 4 parts:

*Part 1: Weekly progress and review (5 minutes)*

*Part 2: Adding interest and variety (20 minutes)*

Guidance on how to add variety to an activity plan is covered. You lead a discussion about how to overcome boredom with a physical activity routine and how to jump an activity plan to make it more enjoyable.

*Part 3: Improving your aerobic fitness (30 minutes)*

You will teach the meaning and importance of aerobic activity. You will explain that increasing aerobic fitness and monitoring how hard our body is working during physical activity helps us reach new and more enjoyable levels of fitness. The FITT principles are discussed and you will explain how to use them to get the most benefit from physical activity.

*Part 4: Wrap up and to-do list (5 minutes)*

**Key messages**

- **Becoming bored with a routine is normal. Add variety to your physical activity to prevent loss of interest or enjoyment.**
- **Avoiding injury is important. When making any changes to your physical activity routine, make sure that the new activity will not increase your risk for injury or take too heavy a toll on your heart.**
- **Increasing activity levels may help to increase your capacity to work hard, which makes it easier to reach and maintain your goal weight.**
- **Pay attention to your body so that you recognize its capabilities and limits while you make any changes to a physical activity plan.**

*Part 1: Weekly progress and review (5 minutes)*

**Distribute:**

- Session 13 handouts
- Session 13 Food and Activity Trackers
- Session 11 Food and Activity Trackers with your feedback.

**Collect** Session 12 Food and Activity Trackers

**Ask:** Did you have any trouble keeping track last week? Were you able to stay under your fat gram and calorie budgets and reach your physical activity goal?

**Open responses.**

**Ask:** How are you feeling this week about your goals and your progress in general?

**Present:** Last week, we talked about the reality of slips in our progress toward goals. We all slip, but slips do not mean failure. We discussed what you can do after you slip and ways to get back on your feet and keep moving toward your goal.

**Ask:** Did you have any slips last week? Did you try your either of your two action plans to get back on the path? (One was for slips in eating, one was for slips in physical activity.)

**Open responses.**

**Present:** This week we will:

- Look at ways to add interest and variety to your activity plans so that you stay motivated.
- Talk about the importance of aerobic fitness and how to make aerobic activity part of your routine.
- Discuss the 4 ideas of F.I.T.T: frequency, intensity, time and type of activity.

*Part 2: Adding interest and variety (20 minutes)*

**Preventing boredom**

**Present:** So far, our physical activity focus is on increasing the amount of time you are active. Remember, we moved gradually from 30-60 minutes per week to 150 minutes per week of physical activity. We suggested and emphasized walking because it is easy to do and does not require special equipment.

By now, some of you may find that your activity routine has become a little stale and boring.

Boredom is a problem because it may cause you to slip back into old habits of physical inactivity. Recognize when you’re feeling bored with your physical activity plan, and do something to keep it fresh and interesting.

Today we will talk about ways to give your activity routine new energy when it begins to feel boring.

**Ways to add variety**

**Ask:** What can you do keep activity from feeling routine or boring?

**Open responses.**

**Ask:** Does anyone have an experience where they became bored with their physical activity and changed something to make it more interesting and less boring?

**Present:** One thing you can do is to add variety. Do something new or different now and then. You cannot expect to do the same activity, day after day, every season of the year, without getting bored. Imagine eating the same food, day after day, month after month, year after year. Remember that you are making lifelong changes, and being active is something we want you to do for the rest of your life. So build in some variety.

**Refer** to the “Ways to prevent boredom” handout.

**Present:** Take a moment to complete this handout.

**Ask:** What are some ways you could build variety into your activity routines?

**Open responses.**

**Praise** all ideas.

**Offer** these suggestions:

- Youtube videos
- Strength training with bodyweight exercises
- Walking in the sand

**Ask:** How about changing where you do your activity?

**Open responses.**

**Offer** these suggestions:

- Walk in a park.
- Walk at the mall.
- Walk at the zoo.
- Walk at the oasis.
- Walk at the farm.

**Ask:** What about being active together; as a way to be social with a family member or friends?

**Open responses.**

**Offer** these suggestions:

- Instead of going for a cup of coffee, go for a walk and talk with a friend or family member.
- Plan a weekend hike with a group of friends.
- Play football.

**Present:** It also helps if you make being active fun.

**Ask:** What ideas do you have for having fun while being physically active?

**Open responses.**

**Offer** these suggestions:

- Some people listen to music or a podcast while they walk or jog.
- When you travel, walk around the cities you visit.

**Present:** Finally, what can you do to motivate yourself to maintain or increase your activity?

**Open responses.**

**Offer** these suggestions:

- Sign up for the Al Ain zoo run (you can walk part or all of it).
- Set up a friendly competition with a friend or family member. For example, whoever walks the fewest miles before a certain date has to buy a healthy lunch.

**Present:** Have you ever been bored with being physically active? What was helpful for you?

**Open responses.**

**Present:** Even if you are not bored now, we can help each other with ideas if you ever get bored.

*Part 3: Improving your aerobic fitness (30 minutes)*

**Present:** One way to add something new to your activity routine is to begin improving your aerobic fitness.

**Ask:** What is “aerobic fitness”?

**Open responses.**

**Present:** Aerobic fitness refers to how well your heart can deliver oxygen through your blood to your muscles, especially the muscles in your arms and legs.

Your heart is a muscle, too. If you exercise your heart, like any other muscle, it will become stronger over time. As your heart becomes stronger, you will notice that it is easier for you to do activities such as walk up stairs and carry groceries. Over time, as your regular physical activity increases, your heart will not need to beat as fast or as hard to do the same level of activity.

For example, if you are more fit than you were, you will notice that walking up a flight of stairs will feel easier; you will not be as out of breath, and your heart will not beat as fast. These changes mean that your heart and lungs are doing the same amount of work with less effort.

**Present:** Not all forms of activity help to strengthen your heart.

**Refer** to the “F.I.T.T principles” handout.

**Present:** We organized the kinds of physical activities that help strengthen the heart to match the abbreviation F.I.T.T.

The F stands for Frequency. This is how often you are active.

Aerobic fitness levels decrease within 48 hours of no activity, so it’s important to be active often.

- Try to be active on most days of the week. At least 3 days a week is recommended, because then less than 48 hours of being inactive happens. But 5-7 days a week of activity is much better.
- Remember that to prevent injury, we increase physical activity slowly. Also, increase the frequency slowly.

The I stands for Intensity. Intensity is how hard you work while you are being active.

Intensity is usually measured by how fast your heart beats. We want you to challenge your heart to beat faster than it normally does so that it will become stronger, but not so hard that you injure yourself.

Other than your heart rate, we can also estimate your intensity by seeing if you can sing. If you are working hard enough while being active, you can have a conversation, but you shouldn’t be able to sing. If you can sing, speed it up!

On the other hand, if you are having trouble breathing and talking while you are being active, slow it down.

With time, you will gradually need to do more to get the same benefit. You may have to walk faster than you used to. This is good! You’re getting stronger and more fit.

The T stands for Time. This is how long you are active.

To improve your aerobic fitness, you have to stay active continuously for 10 minutes or more. That’s why we ask you not to record any activity of less than 10 minutes.

Again, start small and slowly increase the time that you are active to between 20-60 minutes per session. The eventual goal number of minutes of activity per week is 150 minutes or more.

The final T stands for Type. This is the kind of activity you do.

To improve your fitness, you should do aerobic activities, which are activities that challenge your heart. Examples are brisk walking, jogging, swimming, riding a bicycle. These activities use large muscle groups in your arms or legs and last 10 minutes or longer.

Shorter activities that do not require your heart to work harder like walking a short distance in the house or washing a window will not improve your aerobic fitness.

**Present:** Paying attention to our bodies is an important way to keep track of your intensity- how hard you’re working- when you are being active.

**Refer** to the “How hard are you working?” handout.

**Present:** The next time you are active, rate yourself using this scale. Ask yourself, “ How hard am I working?” We want you working hard enough, but not too hard.

*Part 4: Wrap up and to-do list (5 minutes)*

**Present:** For next week, I want each of you to measure your heart rate while you are being active, and try to stay within your target heart rate range.

**Refer** to the “To do next week” handout.

For next week:

1. Keep track of your weight, eating and activity.
2. Do your best to reach your activity goal for the week.
3. Try using one of the FITT principles to jump start your physical activity plan.

**Ask** if there are any question.

**Summarize these key points:**

- **Your activity routine can become boring over time. Add variety so you don’t become less active.**
- **To improve your aerobic fitness, you must increase your activity level to challenge your heart.**
- **Use the FITT principles to get the most out of your active time.**
- **Pay attention to how hard your body is working. Work hard enough but not too hard.**

**Close:** Begin varying your activity routine. Trying something new will make your physical activity more enjoyable. Increase your aerobic fitness while paying attention to your body. Come ready to share your experience next time!

**Ask** participants if there are any questions or concerns.

**After the session:**

Make your notes on participants’ progress and recommend changes.
