# Supplementary material for: Emirates Heart Health Project (EHHP): A protocol for a stepped-wedge family-cluster randomized-controlled trial of a health-coach guided diet and exercise intervention to reduce weight and cardiovascular risk in overweight and obese UAE nationals
Source: PLoS One. 2023 Apr 10;18(4):e0282502. doi: 10.1371/journal.pone.0282502 (PMC10085020; doi:10.1371/journal.pone.0282502)
Supplement: S30 Appendix — (DOCX) [file pone.0282502.s030.docx]

**الجلسة 13: ابدأ خطة نشاطك**


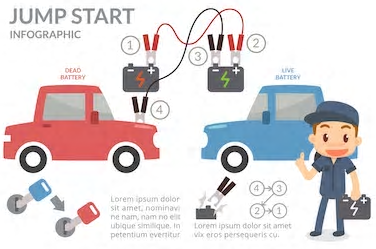


**أهداف التعلم**

- وصف طرق لإضافة الاهتمام والتنوع إلى خطط أنشطتهم.
- تحديد "اللياقة الهوائية".
- شرح مبادئ اللياقة الأربعة (التردد ، الشدة ، الوقت ، ونوع النشاط) وكيفية ارتباطها باللياقة الهوائية.

**المواد**

نشرات الجلسة 13:

- نظرة عامة
- طرق منع الملل
- تحسين لياقتك الهوائية
- المبادئ الاربعة للياقة
- كيف تعمل بجد؟

مهام الأسبوع المقبل

متتبعي الطعام والنشاط للجلسة 13

السبورة والأقلام

**نظرة عامة**

الملل هو سبب مهم لعدم استمرار الناس في ممارسة النشاط البدني. أظهر البحث الذي أجرته الدكتورة جمعة الكعبي في جامعة الإمارات العربية المتحدة أن أكثر من 20٪ من الإماراتيين قالوا إن هذا هو السبب الرئيسي في أنهم لم يستمروا . عندما تصبح خطة النشاط روتينية ، قد يفقد المرء الاهتمام ويعود إلى العادات القديمةكعدم النشاط. لجعل النشاط البدني أكثر متعة ، سيقوم المشاركون بتكوين أفكار حول كيفية إضافة التنوع إلى نشاطهم البدني. سوف نقترح أيضًا طرقًا للقضاء على الملل وزيادة اللياقة البدنية.

تنقسم الجلسة 13 إلى 4 أجزاء:

الجزء الأول: التقدم والمراجعة الأسبوعية (5 دقائق)

الجزء الثاني: إضافة الاهتمام والتنوع (20 دقيقة)

يتم تناول إرشادات حول كيفية إضافة مجموعة متنوعة إلى خطة النشاط. و مناقشة حول كيفية التغلب على الملل في روتين النشاط البدني وكيفية جعله أكثر متعة.

الجزء 3: تحسين لياقتك الهوائية (30 دقيقة)

سوف تعلم معنى وأهمية النشاط الهوائي. سوف نشرح أن زيادة اللياقة الهوائية ومراقبة مدى صعوبة عمل الجسم أثناء النشاط البدني يساعدنا على الوصول إلى مستويات جديدة وأكثر متعة من اللياقة البدنية. تتم مناقشة مبادئ اللياقة وسوف نشرح كيفية استخدامها للحصول على أكبر فائدة من النشاط البدني.

الجزء 4: الختام وقائمة المهام (5 دقائق)

**الرسائل الرئيسية**

- **الشعور بالملل من الروتين أمر طبيعي. أضف مجموعة متنوعة من الأنشطة إلى نشاطك البدني لمنع فقدان الاهتمام أو الاستمتاع.**
- **تجنب الإصابة أمر مهم. عند إجراء أي تغييرات على روتين نشاطك البدني ، تأكد من أن النشاط الجديد لن يزيد من خطر الإصابة أو سيؤثر بشدة على قلبك.**
- **قد تساعد زيادة مستويات النشاط على زيادة قدرتك على العمل بجد ، مما يجعل الوصول إلى الوزن المستهدف والحفاظ عليه أسهل.**
- **انتبه لجسدك حتى تتعرف على قدراته وحدوده أثناء إجراء أي تغييرات على خطة النشاط البدني.**

الجزء الأول: التقدم والمراجعة الأسبوعية (5 دقائق)

**وزع** على المشاركين:

نشرات الجلسة 13

متتبعو الطعام والنشاط الجلسة 13

متتبعو الطعام والنشاط مع تعليقاتكم للجلسة 11.

اجمع متتبعي الطعام والنشاط الجلسة 12

**اسأل:** هل واجهتك أي مشكلة في متابعة المسار الأسبوع الماضي؟ هل كنت قادرًا علىالحفاظ على كمية غرام الدهون والسعرات الحرارية الخاصة بك والوصول إلى هدف نشاطك البدني؟

**استجابات مفتوحة.**

**اسأل:** كيف تشعر هذا الأسبوع بشأن أهدافك وتقدمك بشكل عام؟

الحاضر: تحدثنا الأسبوع الماضي عن واقع الزلات في تقدمنا ​​نحو الأهداف. كلنا ننزلق ، لكن الزلات لا تعني الفشل. ناقشنا ما يمكنك فعله بعد الانزلاق وطرق العودة إلى قدميك ومواصلة التحرك نحو هدفك.

اسأل: هل كان لديك أي قسائم الأسبوع الماضي؟ هل جربت أيًا من خطتي العمل لديك للعودة إلى المسار؟ (واحد كان للزلات في الأكل ، والآخر للزلات في النشاط البدني.)

**استجابات مفتوحة.**

الجزء الأول: التقدم والمراجعة الأسبوعية (5 دقائق)

**وزع** على المشتركين :

نشرات الجلسة 13

- متتبعو الطعام والنشاط للجلسة 13
- متتبعو الطعام والنشاط الجلسة 11 مع تعليقاتك .
- اجمع متتبعي الطعام والنشاط للجلسة 12

**اسأل:** هل واجهتك أي مشكلة في متابعة المسار الأسبوع الماضي؟ هل كنت قادرًا على الحفاظ على كمية الدهون والسعرات الحرارية الخاصة بك والوصول إلى هدف نشاطك البدني؟

**استجابات مفتوحة.**

**اسأل:** بشكل عام كيف تشعرون هذا الأسبوع بشأن أهدافكم وتقدمكم؟

**حاضر**: تحدثنا الأسبوع الماضي عن تأثير الزلات في تقدمنا ​​نحو الأهداف. يمكن ان تحذث للجميع ، لكن الزلات لا تعني الفشل. ناقشنا ما يمكنك فعله وطرق العودة ومواصلة التحرك نحو هدفك.

**اسأل:** هل كان لديك أي زلات أو اخطاء الأسبوع الماضي؟ هل جربت أيًا من خطتي العمل لديك للعودة إلى المسار؟ (واحد كان للزلات في الأكل ، والآخر للزلات في النشاط البدني.)

**استجابات مفتوحة.**

**حاضر**: هذا الأسبوع سوف:

- نبحث عن طرق لإضافة الاهتمام والتنوع إلى خطط أنشطتك حتى تظل متحفزًا.
- نتحدث عن أهمية اللياقة الهوائية وكيفية جعل الأنشطة الهوائية جزءًا من روتينك.
- نناقش الأفكار الأربعة لللياقة:- التكرار والقوة والوقت ونوع النشاط.

**الجزء الثاني: إضافة الاهتمام والتنوع (20 دقيقة)**

**منع الملل**

**حاضر**: حتى الآن ، ينصب تركيز نشاطنا البدني على زيادة الوقت الذينقوم فيه بأداء النشاط الرياضي. تذكر ، لقد انتقلنا تدريجيًا من 30-60 دقيقة أسبوعيًا إلى 150 دقيقة أسبوعيًا من النشاط البدني. كنا قد اقترحنا وشددنا على المشي لأنه من السهل القيام به ولا يتطلب معدات خاصة.

حتى الآن ، قد يجد بعضكم أن روتين النشاط البدني قد أصبح قديمًا ومملًا.

الملل مشكلة لأنها قد تتسبب في العودة إلى العادات القديمة كعدم النشاط البدني. تعرف على شعورك بالملل، وافعل شيئًا لإبقاء خطتك نشاطك البدني جديدة ومثيرة للاهتمام.

سنتحدث اليوم عن طرق لإعطاء نشاطك طاقة جديدة عندما تبدأ في الشعور بالملل.

**طرق لإضافة التنوع في النشاط الرياضي**

**اسأل:** ما الذي يمكنك فعله لمنع النشاط الروتيني أو الممل؟

**استجابات مفتوحة.**

**اسأل**: هل لدى أي شخص خبرة عندما يشعر بالملل من نشاطه البدني و يقوم بتغيير شيء ما لجعله أكثر إثارة للاهتمام وأقل ممل؟

**حاضر:** شيء واحد يمكنك القيام به هو التنوع. افعل شيئًا جديدًا أو مختلفًا بين الحين والآخر. لا يمكنك أن تتوقع القيام بنفس النشاط ، يومًا بعد يوم ، في كل موسم من العام ، دون الشعور بالملل. تخيل أنك تأكل نفس الطعام ، يومًيا أو شهريا أو سنويا. تذكر أنك تقوم بإجراء تغييرات مدى الحياة ، وأن تكون نشطًا هو شيء نريدك أن تفعله لبقية حياتك. لذا قم باضافة بعض التنوع.

**ارجع** إلى نشرة "طرق منع الملل".

**حاضر**: خذو لحظة لإكمال هذه النشرة.

**اسأل:** ما هي بعض الطرق التي يمكنك من خلالها تنويع أنشطتك؟

**استجابات مفتوحة.**

**امدح** كل الأفكار.

**قدم** هذه الاقتراحات:

- فيديو في يوتيوب
- تمارين القوة مع تمارين وزن الجسم
- المشي في الرمال

**اسأل**: ماذا عن تغيير مكان نشاطك؟

**استجابات مفتوحة.**

**قدم** هذه الاقتراحات:

- المشي في حديقة.
- المشي في المركز التجاري.
- المشي في حديقة الحيوان.
- المشي في الواحة.
- المشي في المزرعة.

**اسأل:** ماذا عن النشاط مع مجموعة؟ كطريقة للتواصل الاجتماعي مع أحد أفراد الأسرة أو الأصدقاء؟

**استجابات مفتوحة.**

**قدم** هذه الاقتراحات:

- بدلًا من الذهاب لتناول فنجان من القهوة ، اذهب في نزهة على الأقدام وتحدث مع صديق أو فرد من العائلة.
- خطط لممارسة رياضة المشي لمسافات طويلة مع مجموعة من الأصدقاء
- العب كرة القدم.

**قدم:** يمكن ان تجعل نشاطك الرياضي مرحا.

**اسأل:** ما الأفكار التي لديك للاستمتاع أثناء نشاطك البدني؟

**استجابات مفتوحة.**

**قدم** هذه الاقتراحات:

- يستمع بعض الناس إلى الموسيقى أثناء المشي أو الركض.
- عند السفر ، تجول حول المدن التي تزورها.

**قدم:** أخيرًا ، ما الذي يمكنك فعله لتحفيز نفسك على الحفاظ على نشاطك أو زيادته؟

**استجابات مفتوحة.**

**قدم** هذه الاقتراحات:

- قم بالتسجيل في حديقة حيوانات العين (يمكنك المشي جزئيًا أو كليًا).
- قم بإعداد مسابقة ودية مع صديق أو فرد من العائلة. على سبيل المثال ، من يمشي أقل عدد من الأميال قبل موعد معين يجب عليه شراء غداء صحي.

**حاضر**: هل شعرت بالملل من أي نشاط بدني؟ ما الذي كان مفيدًا لك؟

**استجابات مفتوحة.**

**حاضر:** حتى لو لم تشعر بالملل الآن ، يمكننا مساعدة بعضنا البعض في الأفكار إذا شعرت بالملل.

**الجزء 3: تحسين لياقتك الهوائية (30 دقيقة)**

**حاضر:** إحدى الطرق لإضافة شيء جديد إلى روتين نشاطك هي البدء في تحسين لياقتك الهوائية.

**اسأل**: ما هي "اللياقة الهوائية"؟

**استجابات مفتوحة.**

**حاضر**: تشير اللياقة الهوائية إلى مدى قدرة القلب على توصيل الأكسجين من خلال الدم إلى عضلاتك ، وخاصة عضلات الذراعين والساقين.

قلبك عضلة أيضا. إذا مارست الرياضة ، قلبك مثل أي عضلة أخرى ، سيصبح أقوى بمرور الوقت. عندما يصبح قلبك أقوى ، ستلاحظ أنه من الأسهل عليك القيام بأنشطة مثل صعود الدرج وحمل الأغراض. بمرور الوقت مع زيادة نشاطك البدني المنتظم ، لن تشعر ان قلبك إلى يخفق بسرعةو لن تكون هناك صعوبة في القيام بنفس مستوى النشاط الرياضي.

على سبيل المثال ، إذا كنت لائقًا أكثر مما كنت عليه ، فستلاحظ أن صعود الدرج سوف يكون أسهل. لن تتنفس بشكل سريع ، ولن ينبض قلبك بسرعة. تعني هذه التغييرات أن قلبك ورئتيك يقومان بنفس القدر من العمل بجهد أقل.

**حاضر:** لا تساعد جميع أشكال النشاط على تقوية قلبك.

ارجع إلى نشرة "مبادئ اللياقة "

**حاضر**: قمنا بتنظيم أنواع الأنشطة البدنية التي تساعد على تقوية القلب لتتناسب مع الحروف F.I.T.T.( تعني لائق باللغة الانجليزية )

يشير الحرف F إلى التردد. هذا هو عدد المرات التي تنشط فيها.

تنخفض مستويات اللياقة البدنية خلال 48 ساعة من عدم وجود نشاط ، لذا من المهم أن تكون نشطًا في كثير من الأحيان.

حاول أن تكون نشطًا في معظم أيام الأسبوع. يوصى ب 3 أيام في الأسبوع على الأقل ، لأنه بعد ذلك يحدث أقل من 48 ساعة من عدم النشاط. لكن 5-7 أيام في الأسبوع من النشاط أفضل بكثير.

**تذكر** أنه لمنع الإصابة ، نزيد من النشاط البدني ببطء. أيضا ، زيادة التردد ببطء.

( I ) يعني كثافة. الشدة هي مدى صعوبة عملك أثناء نشاطك.

يتم قياس الشدة عادةً من خلال سرعة دقات القلب.

بخلاف معدل ضربات القلب ، يمكننا أيضًا تقدير شدتك من خلال معرفة ما إذا كان بإمكانك الغناء. إذا كنت تعمل بجد كافٍ أثناء نشاطك ، فيمكنك إجراء محادثة ، ولكن لا يجب أن تكون قادرًا على الغناء. إذا كنت تستطيع الغناء ، قم بتسريعها!

من ناحية أخرى ، إذا كنت تواجه صعوبة في التنفس والتحدث أثناء نشاطك ، فقم بإبطائه.

بمرور الوقت ، ستحتاج تدريجيًا إلى القيام بالمزيد للحصول على نفس الفائدة. قد تضطر إلى المشي بشكل أسرع مما اعتدت عليه. هذا جيد! أصبحت أقوى وأكثر لياقة.

يشير حرف T إلى الوقت. هذه هي مدة نشاطك.

لتحسين لياقتك الهوائية ، يجب أن تظل نشطًا باستمرار لمدة 10 دقائق أو أكثر. لهذا السبب نطلب منك عدم تسجيل أي نشاط أقل من 10 دقائق.

مرة أخرى ، ابدأ قليلًا وزد ببطء وقت نشاطك إلى ما بين 20-60 دقيقة لكل جلسة. العدد النهائي لدقائق النشاط في الأسبوع 150 دقيقة أو أكثر.

يشير الحرف T النهائي إلى النوع. هذا هو نوع النشاط الذي تقوم به.

لتحسين لياقتك ، يجب عليك القيام بأنشطة هوائية ، وهي أنشطة تتحدى قلبك. ومن الأمثلة على ذلك المشي السريع والركض والسباحة وركوب الدراجة. تستخدم هذه الأنشطة مجموعات عضلية كبيرة في ذراعيك أو ساقيك وتستمر لمدة 10 دقائق أو أكثر.

لن تؤدي الأنشطة الأقصر التي لا تتطلب من قلبك أن يعمل بجد أكبر مثل المشي لمسافة قصيرة في المنزل أو غسل نافذة إلى تحسين لياقتك.

**حاضر**: الانتباه إلى أجسادنا هو طريقة مهمة، لتتبع شدتك - مدى صعوبة عملك - عندما تكون نشطًا.

**راجع** "ما مدى صعوبة عملك؟" مذكرة.

**حاضر:** في المرة التالية التي تنشط فيها ، قم بتقييم نفسك باستخدام هذا المقياس. اسأل نفسك ، "ما مدى صعوبة عملي؟" نريدك أن تعمل بجد كافٍ

الجزء 4: ختام وقائمة المهام (5 دقائق)

**حاضر:** في الأسبوع القادم ، أريد منكم قياس معدل ضربات القلب أثناء النشاط ، ومحاولة البقاء ضمن نطاق معدل ضربات القلب المستهدف.

**ارجع** لنشرة "المهام الأسبوع المقبل".

للأسبوع القادم:

1. تتبع وزنك وتناول الطعام والنشاط.

2. ابذل قصارى جهدك للوصول إلى هدف نشاطك للأسبوع.

3. حاول استخدام أحد مبادئ FITT لبدء خطة نشاطك البدني.

**اسأل** عما إذا كان هناك أي سؤال.

**لخص** هذه النقاط الرئيسية:

- يمكن أن يصبح روتين نشاطك مملًا بمرور الوقت. أضف مجموعة متنوعة حتى لا تصبح أقل نشاطًا.
- لتحسين لياقتك الهوائية ، يجب عليك زيادة مستوى نشاطك لتحدي قلبك.
- استخدم مبادئ FITT لتحقيق أقصى استفادة من وقتك النشط.
- انتبه إلى مدى صعوبة عمل جسمك. اعمل بجد كاف ولكن ليس بجهد.

النهاية: ابدأ في تغيير روتين نشاطك. تجربة شيء جديد سيجعل نشاطك البدني أكثر متعة. قم بزيادة لياقتك الهوائية مع الانتباه لجسدك. كن على استعداد لمشاركة تجربتك في المرة القادمة!

**اسأل** المشاركين إذا كانت هناك أي أسئلة أو مخاوف.

بعد الجلسة:

**قم** بتدوين ملاحظاتك حول تقدم المشاركين والتوصية بالتغييرات.
