# Supplementary material for: Emirates Heart Health Project (EHHP): A protocol for a stepped-wedge family-cluster randomized-controlled trial of a health-coach guided diet and exercise intervention to reduce weight and cardiovascular risk in overweight and obese UAE nationals
Source: PLoS One. 2023 Apr 10;18(4):e0282502. doi: 10.1371/journal.pone.0282502 (PMC10085020; doi:10.1371/journal.pone.0282502)
Supplement: S31 Appendix — (DOCX) [file pone.0282502.s031.docx]

**Session 14: Make social cues work for you**

**Learning objectives**

By the end of this session, participants will be able to:

- **Give examples of problem social cues and helpful social cues.**
- **Explain how to remove problem social cues and add helpful social cues.**
- **Describe ways of coping with vacations and social events such as parties, holidays, and visits from relative and friends.**
- **Create an action pain to change a problem social cue and add a helpful social cue.**

**Materials**

- Participant handouts for Session 14
  - Session 14 overview
  - Examples of social cues
  - Dealing with problem social cues
  - Social cues at special events
  - Adding helpful social cues
  - Getting support from others
  - My problem social cues
  - My helpful social cues
  - My action plan for special events
  - To do next week
- Food and Activity Trackers for Session 14
- Whiteboard with markers

**Session 14 overview**

The focus changes to how to stay committed to goals in the face of problem social cues. This builds on what we discussed in Session 8.

Session 14 is divided into 4 parts.

*Part 1: Weekly progress and review (5 minutes)*

*Part 2: The power of social cues (25 minutes)*

You will introduce the idea of social cues and discuss the control they can have over us. You will give examples of problem social cues and helpful social cues. The participants will have the chance to share their own examples. They will learn that habits are formed on the basis of the way we respond to social cues over time.

*Part 3: Changing social cues (25 minutes)*

This section will work on how to come up with plans for dealing with problem social cues by avoiding them or by replacing them with new and healthier helpful cues.

*Part 4: Wrap up and to-do list (5 minutes)*

**Key messages**

- **Social cues- what other people say or do- have a powerful effect on our eating and activity.**
- **Changing the effects of social cues requires that we reduce problem cues and add helpful cues.**
- **Our responses to social cues are often habits that formed over time. To overcome problems, we must change our own habits and learn to respond differently to problem social cues.**

*Part 1: Weekly progress and review (5 minutes)*

**Distribute:**

- Session 14 handouts
- Session 14 “Food and Activity Trackers”
- Session 12 “Food and Activity Trackers” with your notes and feedback

**Collect** Session 13 Food and Activity Trackers.

**Ask:** Did you have any trouble keeping track last week? Were you able to stay within your fat gram and calorie budgets? Did you reach your physical activity goal?

**Open responses.**

**Present:** Last week, we talked about ways to stay motivated as you continue working toward lifestyle goals. We looked at how to add variety to your physical activity plans to avoid boredom. We also discussed adding activities that improve your aerobic fitness. Doing both (adding variety and new activities) will make it easier to stay on your physical activity plan and improve your health.

**Ask:** How many of you worked on changing your routine to prevent becoming bored? What adjustments did you make?

**Open responses.**

**Present:** This week we will cover:

- Social cues: what they are and how they affect us.
- Problem cues and helpful cues.
- How to remove problem cues and add helpful cues.
- How to deal with vacations and social events such as parties and holidays.
- How to create an action plan to change a problem social cue and add a helpful social cue.

*Part 2: The power of social cues (25 minutes)*

**Present:** In Session 8 we talked about how to take charge of what is around you. We took an imaginary tour of your home, and for those of you who work, your work place. We were looking for problem food or activity cues that prompt you to eat unhealthy food or overeat, or might cause you to be inactive.

We planned some ways to remove problem cues and add positive cues (keep your sport/walking shoes where you can see them).

Today we will talk about social cues- what other people say or do that affect our eating and activity patterns. There are again two types of social cues: Problem (or negative) and Helpful (or positive) cues.

Our goal is to replace problem social cues with helpful ones.

**Ask:** Can anyone describe a powerful problem social cue?

**Open responses.**

**Present:** One of the most powerful problem social cues is the sight of other people eating problem foods or being inactive. For example, imagine a wedding or a buffet at a hotel. There is no limit to the amount of food you can eat, and watching the people in line ahead of you putting lots of food on their plate can make you feel like you want to do the same.

**Ask:** Can you think of an example from your own experience? Is it difficult for you when you see a friend or family member eating certain foods?

**Offer** additional examples, if needed.

**Refer** to the “Examples of social cues” handout.

**Present:** Let’s go over together some examples of Problem and Helpful social cues. Let’s start with Problem social cues.

**Offer** these examples: Then have the participants write their own problem social cues on the handout.

- Being offered (or pressured to eat) problem foods or being invited to do something inactive. Example: On a visit to your uncle’s house, they offer you sweets after sweets after sweets. “Why aren’t you eating?”
- Being told not to eat something. Example: “You shouldn’t be eating those French Fries. They are full of fat!” Some people think they are helping, but it can cause us to do the problem behavior more, not less.
- Hearing complaints. Example: Your sister or brother says, “You’re always eating healthy, I miss eating burgers with you.”

**Present:** Now let’s go over some examples of Helpful social cues.

**Offer** as Helpful social cues these examples, and have participants write their own Helpful social cues on their handout.

- The sight of other people eating healthy foods. Example: You go to a restaurant with your brother or sister who is also trying to follow a healthy diet. They suggest a restaurant where healthy options are available. They order first, and they order something healthy.
- Someone is going on a walk, and they invite you to go with them.

**Ask:** Can you think of any other people who are positive influences as you work toward your goals?

Does anyone support you and praise you for your efforts and successes? How does that make you feel?

**Open responses.**

**Present:** Remember, we form habits by responding to a social cue in the same way over and over again. The cue then becomes paired with the way you respond, and your response becomes more and more automatic; you don’t think about it as much any more. Social cues work in the same way.

Let’s say that since childhood, every Friday your mother makes a food that is unhealthy, and after you finish, more of this food is given to you. You developed a habit of accepting the offer. Now when you return home as an adult, and more of this food is given to you after you finish how much you wanted to eat. You find it difficult to refuse.

**Ask:** Do you have any childhood habits that are now hard to break?

**Present:** Understand that with social cues, the other person also learned a habit. So, in the example we just used, your mother learned to offer you more food and expects that you will accept. The involvement of another person, especially a family member, makes social cues even harder to change than other cues.

*Part 3: Changing social cues (25 minutes)*

**Present:** As we discussed during the session on food and activity cues, Problem social cues can be replaced with Helpful cues.

**Ask:** How can you change Problem social cues?

**Open responses.**

**Refer** to the “Dealing with problem social cues” handout.

**Present:** We can get around problem social cues by:

- Staying away from them.
- Changing the cue when possible.
- Responding differently to the cue.

Let’s take a closer look.

- Stay away from the cue.
  - As with problem food cues, staying away from the cue is best. Examples include:
    - Move to a different room when a family member eats problem foods in front of you.
    - Limit eating out as a way to socialize.
    - Suggest other activities when someone suggests watching TV.
    - Change the topic when someone talks about problem foods.
- Change the cue, if you can.
  - Changing the cue requires influencing another person’s part of the habit. When someone pressures you to eat or presents another problem cue, try the following:
    - Discuss the problem with the person directly, and come up with ideas together to solve the problem. “I am trying to eat healthier. It is hard for me when you eat ice cream in front of me. I like spending time with you. Is there a way we can spend time together and enjoy it without eating unhealthy foods?”
    - Be willing to compromise to find a solution that will work for everyone.
    - Tell people about this program, and your effort to reach your goal of losing weight and being healthier. Tell them why this is important to you. Many people would be willing to help if they understand that you are making a change and why.
    - Ask others to encourage you for your efforts and ignoring your slips. Be sure to thank them when they support you.

(Note: you can consider role playing a few examples if you think it would help the group understand.)

- Practice responding in healthier ways.
  - If you cannot stay away from or change the problem social cue, practice responding in a healthier way. Over time you will build a new, healthier habit to the same cue.
    - Say, “No, thank you,” to food offers. If you are consistent and continue saying “No,” others will eventually stop offering.
    - Show others you know they mean well, and suggest how they can help you. Be specific. Most people are trying to show you love and respect when they criticize, offer food, or pressure someone to eat. Many people think they are required as a host to insist that you eat more. If you recognize this, you can offer a specific, positive alternative. Doing this allows the host to feel helpful, and at the same time, assist you in moving toward your goal. For example, instead of cake, say, “Thank you for offering. You know, I would rather have some (fruit, coffee, tea without sugar).”

**Ask:** What are some ways people can help you?

(You can consider role playing exercises here.)

**Present:** Remember that it takes time to change an old habit and build a new one. Change does not happen in one day. With social cues, there are at least two people involved in making a change: you and someone else. Do not expect other people to adjust or change instantly to a new way of relating to you, and don’t expect yourself to change instantly.

**Present:** Social cues are especially powerful at social events such as parties, weddings, Eid meals in your home or someone else’s.

**Refer** participants to the “Social cues at special events” handout.

These events tend to:

- Upset our routine. You usually walk after dinner, so how will you fit in a walk if you are going to a party or wedding?
- Challenge us with special food and social cues. You go on vacation to a place where you don’t know the restaurants. How will you find one that has healthy choices?
- Involve habits that developed over many years and can be powerful.

**Ask:** What are some social events that are difficult for you?

**Present:** To handle social events, try to think about what problems might occur. What might be hard? How will you solve these?

**Review** the handout:

- Plan ahead.
- Stay away from problem cues when you can.
- Change problem cues.
- Respond to problem cues in a healthier way.
- Add helpful social cues.

**Present:** Stay positive. Think of every social event as an opportunity to learn what works well for you and what does not. Remember, this is a process where you are building healthy habits for the rest of your life.

**Present:** Not all social cues are problems. You can use helpful social cues to help you eat and be active in healthier patterns.

**Refer** to the “Adding helpful social cues” handout.

- Spend time with people who are active.
- Spend time with people who make healthy food choices. For example, spend time at weddings with people who are talking or dancing instead of eating.
- Join an exercise club.
- Set up a regular time with others to be active. You are more likely to continue in this because you will not want to disappoint others by canceling.
- Ask your family to remind you to be active.
- Be the first to order when you eat outside of the house, and order something healthy. By setting an example, you may also help others to make healthy choices.
- Be social while being active. Go for a walk and talk with a friend or family member.

**Ask:** Are there people in your life who want to support you or who are already supporting you?

**Open responses.**

**Ask:** What could they do, or what are they already doing to help you?

**Open responses.**

**Refer** to the “Getting support from others” handout.

**Present:** This handout has some ideas. Are any of them helpful? Fill in the blanks by writing in the names of the people who can help you and how they can help you. You can also add other helpful cues at the bottom of the paper.

**Present:** Before we close, let’s work on putting together an action plan for eliminating problem social cues and creating helpful social cues.

**Refer** to the “My problem social cues”, “My helpful social cues”, and “My action plan for special events” handouts.

**Review** the handouts with participants and have them:

- Create a plan to change a problem social cue.
- Create a plan to add a helpful social cue.
- Create a plan for an upcoming social event or holiday.

*Part 4: Wrap up and to-do list*

**Present:** For next week, keep track of your weight, eating and activity, as usual. In addition, using the action plans you just created, work on changing a problem social cue and adding a helpful social cue.

**Refer** to the “To do next week” handout.

**Present:** During the week, put these plans into practice and record on your handout what the plans were, how they went, and what you could have done differently in each case.

**Summarize the key points:**

- **Social cues are powerful and can be difficult to change because other people are involved.**
- **Problem cues are those that can cause us to lose focus on our weight and activity goals.**
- **Your goal is to replace problem social cues with helpful ones.**
- **For difficult social situations, try to think about what problems you might have and plan ahead to overcome them.**
- **Breaking a habit or building a new habit takes time. Change does not happen in one day. We cannot expect ourselves to change in one day, and we cannot expect other people to instantly adjust to who we are becoming.**

**Close:** From this point forward, pay attention to social cues and their effect on your life. These cues are powerful and take persistence and time to change. Do not give up! Stay positive. Removing problem cues creates opportunities for adding helpful ones that can make you healthier and enjoy life more.

**Ask** whether there are any questions.

**After the session:**

Review the Food and Activity Trackers from Session 13 and comment on participants’ successes and challenges.
