# Supplementary material for: Emirates Heart Health Project (EHHP): A protocol for a stepped-wedge family-cluster randomized-controlled trial of a health-coach guided diet and exercise intervention to reduce weight and cardiovascular risk in overweight and obese UAE nationals
Source: PLoS One. 2023 Apr 10;18(4):e0282502. doi: 10.1371/journal.pone.0282502 (PMC10085020; doi:10.1371/journal.pone.0282502)
Supplement: S32 Appendix — (DOCX) [file pone.0282502.s032.docx]

**Session 15: You can manage stress**

**Learning objectives**

At the end of this session, the participants will be able to:

- Explain how to prevent avoidable stress and cope with unavoidable stress.
- Describe how this program can be a source of stress.
- Explain how to manage stressful situations.
- Create and follow an action plan for either preventing or coping with a stressful situation.

**Materials**

- Session 15 handouts
  - Session 15 overview
  - How do you feel stress?
  - Ways to prevent stress
  - When you cannot avoid stress
  - Stress related to this program
  - Your plan for stress
  - To do next week
- Food and Activity Trackers for Session 15
- Whiteboard and markers

**Session 15 overview**

This session discusses ways to avoid and reduce stress in situations that might cause participants to lose focus on (or lose motivation for) achieving their goals. Together, the group will look for ways to avoid or reduce the damaging effects of stress.

Session 15 is divided into 4 parts:

*Part 1: Weekly progress and review (10 minutes)*

*Part 2: Preventing stress (20 minutes)*

You will present how stress affects people and invite participants to share their own experiences with stress and how they cope with it. You will then give some tips for other ways to avoid stress that is avoidable.

*Part 3: Coping with unavoidable stress (20 minutes)*

You will move the discussion to ways to cope with stress that we cannot avoid. Techniques include relaxation, physical activity, and enjoyable activities. Participants will be asked to give examples of what would help them personally and create an action plan for the coming week.

*Part 4: Wrap up and to do list (10 minutes)*

**Key messages**

- **Everyone has pressures that influence the way they feel, think and make decisions. Stress is the tension that develops in response to these pressures.**
- **Any change, good or bad, big or small, can cause stress; stress is a natural part of life.**
- **Stress can be good, but it can also work against you and your goals toward a healthier lifestyle.**
- **Many people react to stress by changing their eating and activity habits.**
- **The best approach to avoiding stress that works against you is to avoid it wherever and whenever possible.**

*Part 1: Weekly progress and review (10 minutes)*

**Distribute:**

- Session 15 handouts
- Session 15 “Food and Activity Trackers”
- Session 13 “Food and Activity Trackers” with your notes.

**Collect** Session 14 “Food and Activity Trackers”.

**Ask:** Were you able to stay within your fat gram and calorie budgets this week? Were you able to reach your activity goal for the week?

**Open responses.**

**Present:** Last week, we looked at problem social cues and helpful social cues. You created action plans for 1) changing a problem social cue, 2) adding a helpful social cue, and 3) planning ahead for a social event.

**Ask:** Were you able to follow your action plans? Would someone volunteer to share their experience?

**Open responses.**

**Praise** any successes. **Address** any questions or difficulties.

**Present:** In this session, we will change our focus to how stresses in life can work against us and our goals that we have for a healthier life. This week we will:

- Talk about how to prevent stress and to cope with stress that we cannot avoid.
- Look at the ways this program might be contributing to your stress and how to cope with that.
- Come up with an action plan for either preventing a stressful situation or coping with it.

*Part 2: Preventing stress (20 minutes)*

**Present:** Everyone has pressures, whether from other people or ourselves, that influence the way we feel, think, and make decisions. The tension that we feel in response to these pressures is called stress. Any change, good or bad, big or small, can cause stress, making stress a natural part of life.

Big changes or events in our lives- getting married, having a serious illness, changing jobs- can cause stress. Even small events- losing our keys, having car trouble, needing to get things done before an appointment- can also cause stress.

**Ask:** What makes you feel stressed?

**Open responses.**

**Refer** to the “How do you feel stress?” handout.

**Present:** Take a moment to write down the kinds of situations that make you feel stressed. Describe how they affect your feeling and your behavior.

**Ask:** Would anyone like to volunteer to share what they wrote?

(Note: If no one shares, mention one or two situations that are stressful for most people: a sick child, pressure at work, car trouble. Ask how the group would feel or react to those situations.)

**Ask:**

- Do you get any physical symptoms such as headache, stomachache, muscle tension?
- Do you change your behavior when you feel stressed?
- Do you eat more when you are stressed?
- Do you eat different kinds of food when you feel stressed?
- Do you change how active you are or the kind of physical activity you do when you are stressed?

**Present:** Why are we talking about stress? With this program, we are interested in stress because many people change how much and what they eat and change how active they are in response to stress. Some people eat and drink too much, or eat and drink unhealthy options as a way to cope with stress. Others may stop eating. Some people may withdraw and become physically inactive.

**Present:** Preventing a problem is better than solving it once it happens. This is also true of negative stress. The best approach to avoiding the problem effects of stress is to prevent negative stress whenever you can.

**Refer** to the “Ways to prevent negative stress” handout.

**Review** the strategies in the handout.

- Practice saying no when it is appropriate. If someone asks you to do something you do not want to do, when possible, say “No.” You should say this politely, and it is often helpful to follow it with a different choice that you would rather do. Example, “I can’t help you with your homework right now, but I will be free later. Can I help you with […}?” Saying “No” can be hard, and it creates its own stress, but that stress may not last as long as if you say “Yes” to something that will cause you negative stress when you do something you do not want to do.
- Share some of your work with others, both at home and at work. For example, a co-worker might be able to help you when you are overwhelmed with something at work. Sharing your work does not mean you are not being responsible. It gives others a chance to learn, participate and gain experience. However, you must then be willing to share their work when they are feeling overwhelmed with their work. Also, avoid criticizing people who are trying to help. Instead, thank them for their help, and be patient with them.
- Set goals you can reach. Sometimes we create our own stress by trying to be perfect, or by having goals that are unreasonable. Remember, we talked about this in the session on negative thoughts: if we try to be perfect, we probably will not succeed. Once in a while, take a good look at the demands you are putting on yourself. Ask yourself, “Am I expecting myself to do more than anyone could possibly do?”
- Take charge of your time. Make schedules that are realistic. Do not try to do in 30 minutes what needs an hour. Take a good look at the things you need to do and remove what is not needed. Give yourself a realistic amount of time to do the rest. Get organized. Use some time every day to get more organized, and this will save you time and negative stress in the future.
- Use the steps for solving problems we discussed in Session 9.
  1. Describe the problem in detail.
  2. Brainstorm your options.
  3. Pick one option that is likely to work and that you can realistically do.
  4. Make an action plan.
  5. Try the action plan.
  6. Continue the process until you find a solution that works.
  7. Move on.
- Plan ahead. Think about the kind of situations that are stressful for you. Those situations put you at risk for slipping- unhealthy eating and being inactive. So plan ahead for what you will do. For example, if you are going to a wedding of your cousin, and you know you will have to eat food that is less healthy, you could change your diet that day and the day before to compensate for it.
- Remember your goal. Maintain a positive attitude. Remember why you are making these changes- to be healthier and enjoy life more!
- Reach out to people. Find people you can ask for support and help. We talked about this last week.
- Be physically active. It may be hard to get started, but most people find that being physically active helps them feel more relaxed and manage stressful situations more smoothly.

*Part 3: Coping with unavoidable stress (20 minutes)*

**Present:** What about the times when we cannot avoid negative stress?

**Ask** participants for examples of negative stress they cannot avoid and how they deal with it.

**Refer** to the “When you cannot avoid negative stress” handout.

**Present:** The first thing we need to do is to *notice that we feel stressed as early as we can.*

We talked in an earlier session about action or behavior chains, and the importance of breaking chains that lead to unhealthy behavior as early as possible. The same is true of stress. If you recognize the signs of negative stress early, you may have more control over how you respond, and avoid some of the harm such as overeating or being inactive.

**Ask:** Do you know of any signs that indicate you are getting stressed or are already stressed?

**Open responses.**

**Present:** As soon as you notice these signs, develop a habit of taking 10 minutes as a break. Stop what you are doing, and take a few minutes for yourself. Do something that you find helpful that does not involve eating.

Here are some possibilities:

- Move your muscles. Research shows that being physically active relieves tension, reduces anxiety and helps depression. So when you notice the signs of negative stress, go for a 10-15 minute brisk walk. The distraction and breathing can help.
- Treat yourself nicely. Read. Watch a funny video. But do not eat.
- Breathe. Most of us hold our breath when we are stressed, which creates more tension.
  - Take a full, deep breath.
  - Count to five.
  - Let your breath out slowly.
  - Let the muscles in your face, arms, legs, and body go completely loose.

**Present:** We understand that this program and the lifestyle changes we are working towards together causes you stress.

**Refer** to the “Stress related to this program” handout.

**Present:** This handout lists some ways that this program causes stress, and gives you some suggestions for how to respond to this stress.

**Review the handout.**

(Some sources of stress in the handout may not apply to every family. For example, some families may already eat low-feat foods. The purpose of this handout is not to create a negative feeling toward this program but to help participants if they encounter the stress that is explained in the handout.)

**Refer** to the “Your plan for stress” handout.

**Ask**: What are your major sources of stress? (This is the first question of the handout.)

**Guide** the group to complete the rest of the handout, which helps them to make an action plan for either preventing or reducing the negative stress that comes from one of their major sources of stress.


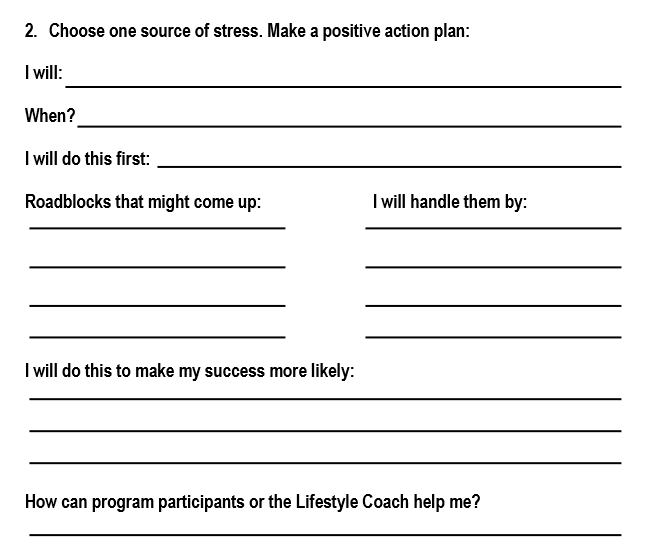


Part 4: Wrap up and to do list (10 minutes)

**Present:** For next week, work on your plan for preventing or coping with the stressful situations you described on the last handout.

**Refer** to the “To do next week” handout.

During the next week, do the following:

- Keep track of your weight, eating and activity.
- Follow your action plan to reduce negative stress. During the week, answer the questions: Did your plan work? If not, what went wrong?

**Summarize the key points:**

- **Stress is the result of various pressures in our lives, and it affects everyone.**
- **Negative stress can make it harder to achieve your goals in this program.**
- **When possible, work to prevent or avoid negative stress instead of coping with it when it comes.**
- **Practice preventing and reducing negative stress using the tools we gave you.**
- **This program can cause stress. Use your action plan to practice coping with program-related stress.**
- **You can manage negative stress instead of having it control you!**

**Close:** Next week is the last session of the program. Do your best to avoid stressful situations. When you cannot avoid negative stress, use the tools we discussed to manage it.

**Ask** participants whether they have any questions before closing the session.

**After the session:**

**Review and make notes on participants’ progress.**
