# Supplementary material for: Emirates Heart Health Project (EHHP): A protocol for a stepped-wedge family-cluster randomized-controlled trial of a health-coach guided diet and exercise intervention to reduce weight and cardiovascular risk in overweight and obese UAE nationals
Source: PLoS One. 2023 Apr 10;18(4):e0282502. doi: 10.1371/journal.pone.0282502 (PMC10085020; doi:10.1371/journal.pone.0282502)
Supplement: S33 Appendix — (DOC) [file pone.0282502.s033.doc]

**الجلسة 15: يمكنك إدارة التوتر**

**أهداف التعلم**

**في نهاية هذه الجلسة ، سيتمكن المشاركون من:**

- شرح كيفية منع الإجهاد الذي يمكن تجنبه والتعامل مع الإجهاد الذي لا مفر منه.
- وصف كيف يمكن أن يكون هذا البرنامج مصدرًا للتوتر.
- شرح كيفية إدارة المواقف العصيبة.
- وضع خطة عمل واتباعها إما لمنع المواقف العصيبة أو التعامل معها.

**المواد**

- منشورات الجلسة 15
- نظرة عامة على الجلسة 15
- كيف تشعر بالتوتر؟
- طرق منع التوتر
- ماذا تفعل عندما لا تستطيع تجنب التوتر
- الإجهاد المرتبط بهذا البرنامج
- خطتك للتوتر
- مهام الأسبوع المقبل
- أدوات تعقب الطعام والنشاط للجلسة 15
- السبورة والأقلام

**نظرة عامة على الجلسة 15**

تناقش هذه الجلسة طرق تجنب وتقليل التوتر في المواقف التي قد تجعل المشاركين يفقدون التركيز (أو يفقدون الدافع) لتحقيق أهدافهم. ستبحث المجموعة معًا عن طرق لتجنب أو تقليل الآثار الضارة للتوتر.

**الجلسة 15 مقسمة إلى 4 أجزاء:**

الجزء 1: التقدم الأسبوعي والمراجعة (10 دقائق)

الجزء 2: منع التوتر (20 دقيقة)

سوف تشرح كيف يؤثر الضغط على الناس وتدعو المشاركين لمشاركة تجاربهم الخاصة مع التوتر وكيف يتعاملون معه. ستقدم بعد ذلك بعض النصائح لطرق أخرى لتجنب التوتر الذي يمكن تجنبه.

الجزء 3: التعامل مع الإجهاد الذي لا مفر منه (20 دقيقة)

ستنقل المناقشة إلى طرق للتعامل مع التوتر لا يمكننا تجنبه. تشمل التقنيات الاسترخاء والنشاط البدني والأنشطة الممتعة. سيُطلب من المشاركين تقديم أمثلة لما يمكن أساعدهم شخصيًا ووضع خطة عمل للأسبوع القادم.

الجزء 4: اختتام قائمة المهام (10 دقائق)

**الرسائل الرئيسية**

- كل شخص لديه ضغوط تؤثر على الطريقة التي يشعر بها ويفكر ويتخذ قراراته. الإجهاد هو التوتر الذي يتطور استجابة لهذه الضغوط.
- أي تغيير ، جيد أو سيئ ، كبير أو صغير ، يمكن أن يسبب التوتر ؛ التوتر هو جزء طبيعي من الحياة.
- يمكن أن يكون التوتر أمرًا جيدًا ، ولكنه قد يعمل أيضًا ضدك وضد أهدافك نحو أسلوب حياة أكثر صحة.
- يتفاعل العديد من الأشخاص مع الإجهاد من خلال تغيير عاداتهم الغذائية والنشاطية.
- أفضل طريقة لتجنب الإجهاد هو تجنبه حيثما وكلما أمكن ذلك.

**الجزء 1: التقدم الأسبوعي والمراجعة (10 دقائق)**

**وزع:**

- توزيعات الجلسة 15
- "أدوات تتبع الطعام والنشاط" للجلسة 15
- "تعقب الطعام والنشاط" مع ملاحظاتك للجلسة 13
- اجمع "أدوات تتبع الطعام والنشاط" للجلسة 14

**اسأل:** هل كنت قادرًا على البقاء في حدود ميزانيات الدهون والسعرات الحرارية هذا الأسبوع؟ هل تمكنت من الوصول إلى هدف نشاطك لهذا الأسبوع؟

**افتح المجال للرد**

**حاضر:** في الأسبوع الماضي ، نظرنا في الإشارات الاجتماعية السلبية والإشارات الاجتماعية الايجابية. لقد أنشأت خطط عمل من أجل 1) تغييرالاشارة اجتماعية السلبية، 2) إضافة اشارة اجتماعية مفيدة ، و 3) التخطيط المسبق لحدث اجتماعي.

**اسأل:** هل كنت قادرًا على متابعة خطط عملك؟ هل يتطوع شخص ما لمشاركة تجربته؟

**افتح المجال للرد**

**امدح** أي نجاحات. **عالج** أي أسئلة أو صعوبات.

**حاضر**: في هذه الجلسة ، سنغير تركيزنا إلى كيف يمكن للضغوط في الحياة أن تعمل ضدنا وضد أهدافنا المتمثلة في حياة أكثر صحة. هذا الأسبوع سوف:

- نتحدث عن كيفية منع التوتر والتعامل مع التوتر الذي لا يمكننا تجنبه.
- ننظر إلى الطرق التي قد يساهم بها هذا البرنامج في كيفية التعامل مع توترك.
- نضع خطة عمل إما لمنع المواقف العصيبة أو التعامل معها.

**الجزء 2: منع التوتر (20 دقيقة)**

**حاضر**: كل شخص لديه ضغوط ، سواء من الآخرين أو من أنفسنا ، والتي تؤثر على الطريقة التي نشعر بها ونفكر بها ونتخذها. يسمى التوتر الذي نشعر به استجابة لهذه الضغوط بالتوتر. أي تغيير ، سواء كان جيدًا أو سيئًا ، كبيرًا كان أم صغيرًا ، يمكن أن يسبب التوتر ، مما يجعل التوتر جزءًا طبيعيًا من الحياة.

التغييرات أو الأحداث الكبيرة في حياتنا - الزواج ، الإصابة بمرض خطير ، تغيير الوظائف - يمكن أن تسبب التوتر. حتى الأحداث الصغيرة - فقدان مفاتيحنا ، أو وجود مشكلة في السيارة ، أو الحاجة إلى إنجاز الأشياء قبل الموعد - يمكن أن تسبب التوتر أيضًا.

**اسأل:** ما الذي يجعلك تشعر بالتوتر؟

**افتح المجال للرد**

**ارجع** الى مذكرة "كيف تشعر بالتوتر؟" .

**حاضر:** خذ لحظة لكتابة أنواع المواقف التي تجعلك تشعر بالتوتر. صف كيف تؤثر على مشاعرك وسلوكك.

**اسأل**: هل يرغب أي شخص في التطوع لمشاركة ما كتبه؟

(ملاحظة: إذا لم يشارك أحد ، اذكر موقفًا أو حالتين مرهقتين لمعظم الناس: طفل مريض ، ضغط في العمل ، مشكلة في السيارة. اسأل عن شعور المجموعة أو رد فعلها تجاه هذه المواقف.)

**اسأل:**

هل تعاني من أي أعراض جسدية مثل الصداع وآلام المعدة وتوتر العضلات؟

هل تغير سلوكك عندما تشعر بالتوتر؟

هل تأكل أكثر عندما تكون متوترًا؟

هل تأكل أنواعًا مختلفة من الطعام عندما تشعر بالتوتر؟

هل تغير مدى نشاطك أو نوع النشاط البدني الذي تقوم به عندما تكون متوترًا؟

**حاضر**: لماذا نتحدث عن التوتر؟ مع هذا البرنامج ، نحن مهتمون بالتوتر لأن العديد من الأشخاص يغيرون مقدار ما يأكلونه وما يأكلونه ويغيرون مدى نشاطهم في الاستجابة للتوتر. بعض الناس يأكلون ويشربون الكثير ، أو يأكلون ويشربون خيارات غير صحية كوسيلة للتغلب على التوتر. قد يتوقف الآخرون عن الأكل. قد ينسحب بعض الأشخاص ويصبحون غير نشيطين بدنيًا.

**حاضر:** منع المشكلة أفضل من حلها بمجرد حدوثها. هذا ينطبق أيضًا على الإجهاد السلبي. أفضل طريقة لتجنب آثار مشكلة التوتر هو منع الإجهاد السلبي كلما أمكن ذلك.

**ارجع** إلى نشرة "طرق لمنع الإجهاد السلبي".

**راجع** الاستراتيجيات الموجودة في النشرة.

- **تدرب على قول لا عندما يكون ذلك مناسبًا.** إذا طلب منك شخص ما أن تفعل شيئًا لا تريد فعله ، فقل "لا" إن أمكن. يجب أن تقول هذا بأدب ، وغالبًا ما يكون من المفيد اتباعه بخيار مختلف تفضل القيام به. على سبيل المثال ، "لا يمكنني مساعدتك في أداء واجبك المنزلي الآن ، ولكن سأكون متفرغًا لاحقًا. هل يمكنني مساعدتك في [...}؟ " قد يكون قول "لا" أمرًا صعبًا ، ويخلق ضغوطًا خاصة به ، ولكن هذا التوتر قد لا يستمر طالما قلت "نعم" لشيء يسبب لك ضغوطًا سلبية عندما تفعل شيئًا لا تريد فعله.
- **شارك بعض أعمالك مع الآخرين** ، سواء في المنزل أو في العمل. على سبيل المثال ، قد يكون زميل العمل قادرًا على مساعدتك عندما تكون غارقًا في شيء ما في العمل. لا تعني مشاركة عملك أنك لست مسؤولاً. يمنح الآخرين فرصة للتعلم والمشاركة واكتساب الخبرة. ومع ذلك ، يجب أن تكون على استعداد لمشاركتهم عملهم عندما يشعرون بالإرهاق. أيضًا ، تجنب انتقاد الأشخاص الذين يحاولون المساعدة. بدلاً من ذلك ، اشكرهم على مساعدتهم ، وكن صبورًا معهم.
- **ضع أهدافًا يمكنك الوصول إليها.** أحيانًا نخلق ضغوطًا خاصة بنا من خلال محاولة أن نكون مثاليين ، أو من خلال وضع أهداف غير معقولة. تذكر ، تحدثنا عن هذا في الجلسة حول الأفكار السلبية: إذا حاولنا أن نكون مثاليين ، فربما لن ننجح. من حين لآخر ، ألق نظرة فاحصة على المطالب التي تضعها على نفسك. اسأل نفسك ، "هل أتوقع من نفسي أن أفعل أكثر مما يمكن لأي شخص فعله؟"
- **تولي مسؤولية وقتك.** ضع جداول زمنية واقعية. لا تحاول أن تفعل في 30 دقيقة ما يحتاج إلى ساعة. ألق نظرة فاحصة على الأشياء التي تحتاج إلى القيام بها وقم بإزالة ما هو غير ضروري. امنح نفسك قدرًا واقعيًا من الوقت للقيام بالباقي. كن منظمًا. استغل بعض الوقت كل يوم لتصبح أكثر تنظيمًا ، وهذا سيوفر لك الوقت والضغط السلبي في المستقبل.

**استخدم** خطوات حل المشكلات التي ناقشناها في الجلسة 9.

1. **وصف المشكلة بالتفصيل.**

**2. قم بالعصف الذهني لخياراتك.**

**3. اختر خيارًا واحدًا من المحتمل أن ينجح ويمكنك القيام به بشكل واقعي.**

**4. ضع خطة عمل.**

**5. جرب خطة العمل.**

**6. استمر في العملية حتى تجد الحل المناسب.**

**7. المضي قدما.**

- **خطط مسبقا**. فكر في نوع المواقف التي تسبب لك التوتر. تعرضك هذه المواقف لخطر عدم الالتزام - الأكل غير الصحي وعدم النشاط. لذا خطط مسبقًا لما ستفعله. على سبيل المثال ، إذا كنت ستذهب إلى حفل زفاف لابن عمك ، وتعلم أنه سيتعين عليك تناول طعام أقل صحة ، يمكنك تغيير نظامك الغذائي في ذلك اليوم واليوم السابق للتعويض عن ذلك.
- **تذكر هدفك.** حافظ على موقف إيجابي. تذكر سبب إجراء هذه التغييرات - لتكون أكثر صحة والاستمتاع بالحياة أكثر!
- **تواصل مع الناس**. ابحث عن أشخاص يمكنك طلب الدعم والمساعدة. تحدثنا عن هذا الأسبوع الماضي.
- **كن نشيطًا بدنيًا.** قد يكون من الصعب البدء ، ولكن يجد معظم الناس أن النشاط البدني يساعدهم على الشعور بمزيد من الاسترخاء وإدارة المواقف العصيبة بسلاسة أكبر.

**الجزء 3: التعامل مع الإجهاد الذي لا مفر منه (20 دقيقة)**

**حاضر:** ماذا عن الأوقات التي لا يمكننا فيها تجنب الإجهاد السلبي؟

**اسأل** المشاركين عن أمثلة للتوتر السلبي الذي لا يمكنهم تجنبه وكيف يتعاملون معه.

**ارجع** إلى نشرة "عندما لا يمكنك تجنب الإجهاد السلبي".

**حاضر:** أول شيء علينا القيام به هو أن نلاحظ أننا نشعر بالتوتر في أقرب وقت ممكن.

تحدثنا في جلسة سابقة عن قيود الإجراءات أو السلوك ، وأهمية كسر القيود التي تؤدي إلى سلوك غير صحي في أقرب وقت ممكن. نفس الشيء ينطبق على التوتر. إذا كنت تتعرف على

علامات الإجهاد السلبي في وقت مبكر ، قد يكون لديك المزيد من التحكم في كيفية استجابتك ، وتجنب بعض الضرر مثل الإفراط في تناول الطعام أو عدم النشاط.

**اسأل:** هل تعرف أي علامات تشير إلى أنك تعاني من التوتر أو أنك بالفعل مضغوط؟

**افتح المجال للرد**

**حاضر**: بمجرد ملاحظة هذه العلامات ، قم بتطوير عادة أخذ 10 دقائق راحة. توقف عما تفعله وخذ بضع دقائق لنفسك. افعل شيئًا تجده مفيدًا لا يتضمن تناول الطعام.

**فيما يلي بعض الحلول:**

- **حرك عضلاتك.** تظهر الأبحاث أن النشاط البدني يخفف التوتر ويقلل من القلق ويساعد على الاكتئاب. لذلك عندما تلاحظ علامات الإجهاد السلبي ، قم بالمشي السريع لمدة 10-15 دقيقة. يمكن أن يساعد الإلهاء والتنفس.
- **عامل نفسك بلطف.** اقرأ. شاهد فيديو مضحك. لكن لا تأكل.
- **تنفس**. يحبس معظمنا أنفاسه عندما نكون مرهقين ، مما يخلق المزيد من التوتر.
  - خذ نفسا كاملا وعميقا.
  - عد إلى خمسة.
  - دع أنفاسك ببطء.
- **ارخي عضلات** وجهك وذراعيك وساقيك وجسمك.

**حاضر**: نتفهم أن هذا البرنامج وتغييرات نمط الحياة التي نعمل من أجلها معًا تسبب لك التوتر.

ارجع الى نشرة "الإجهاد المتعلق بهذا البرنامج".

**حاضر:** تسرد هذه النشرة بعض الطرق التي يسبب بها هذا البرنامج التوتر ، وتعطيك بعض الاقتراحات حول كيفية الاستجابة لهذا الضغط.

راجع النشرة.

(قد لا تنطبق بعض مصادر التوتر في النشرة على كل أسرة. على سبيل المثال ، قد تتناول بعض العائلات بالفعل أطعمة منخفضة القيمة. والغرض من هذه النشرة ليس خلق شعور سلبي تجاه هذا البرنامج ولكن لمساعدة المشاركين إذا واجهوا الضغط الموضح في النشرة.)

**ارجع** إلى نشرة "خطتك للتوتر".

**اسأل:** ما هي المصادر الرئيسية للتوتر؟ (هذا هو السؤال الأول في النشرة.)

**قم** بتوجيه المجموعة لإكمال بقية النشرة ، مما يساعدهم على وضع خطة عمل إما لمنع أو تقليل الضغط السلبي الذي يأتي من أحد مصادر التوتر الرئيسية لديهم.


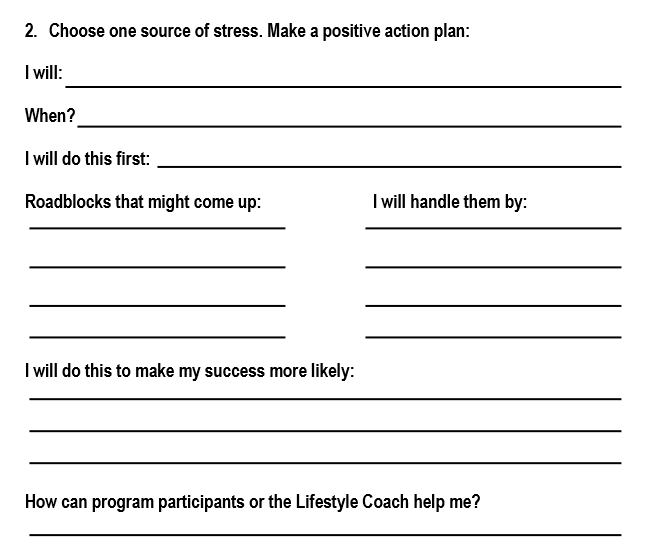


**الجزء 4: اختتام قائمة المهام (10 دقائق)**

**حاضر**: للأسبوع المقبل ، اعمل على خطتك لمنع المواقف العصيبة التي وصفتها في النشرة الأخيرة أو التعامل معها.

**ارجع** إلى نشرة "المهام الأسبوع المقبل".

**خلال الأسبوع القادم ، سنقوم بما يلي:**

- تتبع وزنك وتناول الطعام والنشاط.
- تتبع خطة عملك لتقليل التوتر السلبي. خلال الأسبوع ، أجب عن الأسئلة: هل نجحت خطتك؟ إذا لم يكن كذلك ، فما الخطأ الذي حدث؟

**لخص النقاط الرئيسية:**

- الإجهاد هو نتيجة الضغوط المختلفة في حياتنا ، ويؤثر على الجميع.
- يمكن أن يجعل الإجهاد السلبي من الصعب تحقيق أهدافك في هذا البرنامج.
- عندما يكون ذلك ممكنًا ، اعمل على منع أو تجنب الإجهاد السلبي بدلاً من التعامل معه عندما يأتي.
- تدرب على منع وتقليل الإجهاد السلبي باستخدام الأدوات التي قدمناها لك.

هذا البرنامج يمكن أن يسبب الإجهاد. استخدم خطة العمل الخاصة بك لممارسة التكيف مع الضغط المرتبط بالبرنامج.

**يمكنك إدارة التوتر السلبي بدلاً من السيطرة عليك!**

**خاتمة** : الأسبوع القادم هو آخر جلسة للبرنامج. ابذل قصارى جهدك لتجنب المواقف العصيبة. عندما لا تستطيع تجنب الإجهاد السلبي ، استخدم الأدوات التي ناقشناها لإدارتها.

**اسأل** المشاركين عما إذا كانت لديهم أية أسئلة قبل انتهاء الجلسة.

**بعد الجلسة:**

**قم بمراجعة وتدوين ملاحظات حول تقدم المشاركين.**
