# Supplementary material for: Emirates Heart Health Project (EHHP): A protocol for a stepped-wedge family-cluster randomized-controlled trial of a health-coach guided diet and exercise intervention to reduce weight and cardiovascular risk in overweight and obese UAE nationals
Source: PLoS One. 2023 Apr 10;18(4):e0282502. doi: 10.1371/journal.pone.0282502 (PMC10085020; doi:10.1371/journal.pone.0282502)
Supplement: S34 Appendix — (DOCX) [file pone.0282502.s034.docx]

**Session 16: Ways to stay motivated**

**Learning objectives**

At the close of this session, the participants will be able to:

- Measure their progress toward their weight and physical activity goals since Session 1.
- Develop a plan for improving progress, if their goals have not yet been attained.
- Describe ways to stay motivated long-term.

**Materials**

- Participant handouts for Session 16:
  - Session 16 overview
  - Progress review
  - Ways to stay motivated
  - A plan for staying motivated
  - To do next week
- Food and Activity Trackers for Session 16
- Whiteboard with markers

**Session 16 overview**

Conclude the program by evaluating each participant’s progress and helping each of them to come up with ways to stay motivated after the program.

Session 16 is divided into 4 parts:

*Part 1: Weekly progress and review (10 minutes)*

Review the information covered in Session 15. Discuss participants’ successes, challenges, and questions since the last time the group met.

*Part 2: Review of goals and accomplishments (20 minutes)*

Ask participants to consider their accomplishments during the last 16 sessions. Review each person’s progress toward their goals since the start of the program, and for those who have not yet met their goals, recommend ways to continue to work toward achieving them.

*Part 3: Staying motivated (20 minutes)*

Focus on helping participants find ways to stay motivated. Give them a list of suggestions for staying motivated, and ask them to choose the strategies that work best for them.

*Part 4: Wrap up and to do list (10 minutes)*

Using the tools and information learned over the 16 sessions, help participants build a long-term plan for staying motivated as they move into the post-core phase of the program.

**Key messages**

- **You accomplished great things and took important steps toward a healthier way of life and preventing heart disease.**
- **Although you finished 16 sessions, the weight loss and physical activity goals remain in place for the rest of your life.**
- **Staying motivated is crucial to maintaining healthy eating and physical activity for the long term, but staying motivated is one of the biggest problems people face.**
- **Keep sight of your successes and progress so far. Knowing what you CAN do helps keep you motivated to continue in your new, healthier lifestyle.**

*Part 1: Weekly progress and review (10 minutes)*

**Distribute:**

- Session 16 handouts
- Session 14 Food and Activity Trackers with your notes.

**Collect:** Session 15 Food and Activity Trackers

**Ask:** Did you have any trouble keeping track last week? Were you ale to stay within your fat gram and calorie budgets? Did you reach your goal for physical activity?

**Open responses.**

**Present:** Last week, we talked about ways to prevent or cope with negative stress, including stress related to your efforts in making healthy lifestyle changes. We looked at ways to manage stressful situations and came up with a plan to work on preventing or coping with stressful situations during last week.

**Ask:** Were you able to follow your action plan to prevent or cope with one source of stress?

**Open responses.**

**Praise all progress.**

**Present:** You have reached Session 16 of the Emirates Heart Health Program! You accomplished great things since week 1: you took important steps toward reducing your risk of heart disease and toward living a healthier life.

**CONGRATULATIONS!**

This week we will:

- Discuss your many accomplishments since Session 1 and your progress toward your goals.
- Talk about the ways you can stay motivated so you can have these benefits for the rest of your life.

*Part 2: Review of goals and accomplishments (20 minutes)*

(Note: as you congratulate participants for completing the program, briefly mention a personal accomplishment of each one; a particular contribution to the group, persevering toward their goal, etc.)

**Present:** Keep in mind that the weight loss and physical activity goals remain in place for the rest of your life.

(Note: If some participants have not reached their goals, emphasize the importance of continuing to work toward their goals. To participants who achieved their goals, emphasize the importance of maintaining their weight and level pf activity and setting new goals.)

**Present:** Today we are going to talk about how to stay motivated for the long term, and how to make your commitment to healthy eating and physical activity last for the rest of your life. But first, let’s review your progress since the beginning of the program.

**Refer** to the “Progress review” handout.

**Ask:** What are some of the major changes you made to be more active?

**Open responses.**

**Present:** Take a minute to write down these changes on the handout. Include both what activities you do that you have been recording as well as the changes you made to be more active in general (the activity you do not record such as taking the stairs instead of the elevator).

**Ask:** What changes did you make o eat less fat and fewer calories?

**Open responses.**

**Present:** Write the changes in your eating habits on the handout.

(Note: Praise participants for the changes, and encourage them to keep them up.)

**Ask:** Have you reached your weight goal? What about your activity goal?

**Refer** to the “How am I doing?” charts for weight and physical activity.

**Present:** Check the boxes on the “Progress review” handout to record whether or not you achieved your goals set at the beginning of the program.

If you have not met your weight or activity goals, take a few minutes to think about, and write down, what you can do to improve your progress.

If you have met your goals, choose new goals for the next 6 months. For example, do you want to continue losing weight, or do you want to maintain your current weight? Do you want to increase your physical activity further? Or do you want to maintain the current level?

**Praise all progress.**

*Part 3: Staying motivated (20 minutes)*

**Present:** Motivation is crucial to maintaining healthy eating and physical activity for the long term, but staying motivated to do this is one of the biggest problems people face.

Sometimes staying motivated is difficult just because we are doing well. This is strange- our progress itself makes it hard to maintain that progress. But think back to when you first started this program.

(Note: adjust the following examples to fit experiences that have been shared by participants so far.)

You felt tired when you walked, motivating you to become more active. Now that you are more active, walking is easy. This means that your source of motivation- feeling short of breath when you walked- is gone.

Or maybe you did not like the way you looked. If you like the way you look now better, it is not as strong of a motivation for you.

**Present:** However, it is possible to stay motivated for the long term, and as we discussed, staying motivated is important to maintaining healthy eating and regular activity.

**Refer** to the “Ways to stay motivated” handout.

**Present:** Here are 9 strategies that other people have found helpful for staying motivated.

1. Stay aware of the goals you’ve already reached and the goals you plan to reach.
2. Recognize your successes.
3. Keep visible signs of your progress.
4. Keep track of your weight, eating, and activity.
5. Add variety to your routine.
6. Identify additional strategies.
7. Create some friendly competition.
8. Remember, slips are normal.
9. Look to others to stay motivated.

Let’s look at each strategy in closer detail.

Strategy #1: Stay aware of the goals you’ve already reached and the goals you plan to reach.

Again, remember when you first started the program. What goals did you set?

Now think about where you are today. You came a long way.

(Note: Acknowledge any positive or negative consequences in the process)

**Ask:** Did you reach the goals you set out to achieve? Did you receive any benefits that you did not expect? Write those benefits on your handout.

**Ask:** What would you like to achieve in the next 6 months? Write these down on your handout.

**Ask:** for volunteers to share what they want to achieve.

Strategy #2: Recognize your successes.

**Ask:** What changes in your eating and physical activity habits are you most proud of?

What was easier to change than you thought it would be?

What was harder to change than you thought it would be?

**Present:** When your motivation is running low (and it will), think about these positive changes, and give yourself credit for them. Ask yourself if you want to give those up. Try not to lose momentum.

Strategy #3: Keep visible signs of your progress.

**Ask:** What are some ways you might do this? Any ideas you would like to share?

**Open responses.**

**Present:** Here are some suggestions that others have used:

- Measure yourself monthly.
- Print out a map of your neighborhood. Mark how far you can walk. Find a place past that. Keep marking your progress until you can get all the way there. Reward yourself.

Strategy #4: Keep track of your weight, eating, and activity.

**Present:** It is common for us to slowly move from new habits to older ones. You may gradually slip and make small changes in your eating and physical activity over a long time and not even be aware that you are slowly going back to your old habits. The best way to prevent this and stay in control is to continue keeping track of your weight, eating, and activity. This will help you to catch changes earlier.

Please write down on the handout how you plan to keep tracking these.

The more you track, the better. Try to keep track of how you are eating at least one week out of the month. If you notice you are gaining weight, you should go back to tracking what you eat every day.

Strategy #5: Add variety to your routine.

**Present:** We’ve talked about this in our session on jump starting your activity plan. Were you able to variety to keep yourself from becoming bored with physical activity? Did you notice a difference in how you felt about physical activity?

The same is true with eating. You don’t have to eat the same thing every night. Experiment with different low-fat, low-calorie foods. Try new foods, visit new restaurants.

**Ask:** What meals, snacks, or particular foods have become boring for you? Can you think of some ways to vary your eating? Does anyone have suggestions about healthy foods that they enjoy?

**Open responses.**

**Present:** (If not already shared, you can use these.) Here are some suggestions for adding variety to food:

- Use different seasonings or spices to add flavor to low-fat dishes.
- Eat a wide range of different fruits, vegetables, and grains.
- Have a variety of colors, textures, and tastes on your plate.
- Make one night a week a night where you serve a new food.
- If you eat out often, plan to have more meals at home.
- If you eat at home, often, find some restaurants that serve healthy food that you want to try. Or adjust your food intake and physical activity to allow you eat food that you really enjoy once in a while.

**Present:** Take a short time and write your ideas on the handout.

Strategy #6: Identify new ways to challenge yourself. Reward yourself when you reach your goal.

**Present:** New challenges should be specific and short-term (“I will not drink any cola this week.”) It should be something that is not too easy or not too hard. It should be something you can do, but also hard enough that you will feel you really did something if you succeed.

The reward should be something you will do or buy if, AND ONLY IF, you reach your goal. It does not have to be expensive.

For example, “After I walk ten more minutes, I will watch some funny videos on my mobile.” Then, if you need a boost to keep going, you can think about how much you will laugh after your walk.

**Ask:** What are some ways you can reward yourself without food?

**Open responses.**

**Offer** these ideas:

- Fresh flowers
- Manicure
- Set aside a small amount of money for something you want to do or buy.

**Ask** participants to write their reward ideas on the handout.

Strategy #7: Create some friendly competition.

**Present:** Have a friendly competition with a friend or someone in your family. The prize should be something you both win.

Here are some suggestions:

- If you and your friend are both active every day for a month, at the end of the month you will both ______________.
- If you are active every day and your cousin does her homework every day for a month, you will both treat yourselves to _____________.
- See how many days in a row you can be active for at least 30 minutes. Try to beat your best. For example, if you were active for three days in a row last month, see if you can do four this month.

Strategy #8: Remember, slips are normal.

**Present:** If you do slip, as soon as possible, begin focusing on how you can get back on track. Remembering that slips are a normal part of the process will help you to keep looking forward to your goal, instead of focusing on the slip itself.

Strategy #9: Look to others for help in staying motivated.

**Present:** Now you have a group of people that understand what you are trying to do. Help each other stay motivated. Everyone has trouble staying focused sometimes, but we can encourage and support each other through those times.

*Part 4: Wrap up and to do list (10 minutes)*

**Present:** We have reached the end of this program. Continue to use the tools you learned during these 16 sessions. Continue self-monitoring, setting goals, staying motivated, and overcoming challenges.

Now let’s make a plan for staying motivated as you progress.

**Refer** to the “A plan for staying motivated” handout.

**Present:** Create a plan that will keep you motivated. Pick what you think works best for you. Choose something that is likely to work and that you can do. Be realistic and specific. Write down what you choose to do.

**Summarize these key points:**

- **You completed the Emirates Heart Health Program. But we want you to continue and improve in your diet and physical activity.**
- **Some of you achieved your goal, and some of you are still on the way. Do not give up. What matters most is your determination to continue working in a healthy direction.**
- **There are many ways to stay motivated. Pick the way or ways that work best for you. If it doesn’t work, pick another one and try again.**

**Close:** Remember the distance you have come. You now have tools and commitment to make and maintain positive lifestyle changes. You can succeed in achieving the goals you have set for yourself.

You have the power to choose what you eat and how physically active you are. You have the power to control your surroundings, the power to say no in a friendly but firm way, the power to make time to take care of yourself, the power to prevent negative thoughts, and the power to change.

**Ask** if there are any questions or concerns.

**Remind** participants that they will need to check their labs, blood pressure and weight at the clinic.

**Remind** participants that dietician support is available through Ambulatory Health Services.

**Collect** equipment for return to UAEU.
