# Supplementary material for: Emirates Heart Health Project (EHHP): A protocol for a stepped-wedge family-cluster randomized-controlled trial of a health-coach guided diet and exercise intervention to reduce weight and cardiovascular risk in overweight and obese UAE nationals
Source: PLoS One. 2023 Apr 10;18(4):e0282502. doi: 10.1371/journal.pone.0282502 (PMC10085020; doi:10.1371/journal.pone.0282502)
Supplement: S35 Appendix — (DOC) [file pone.0282502.s035.doc]

**الجلسة 16: طرق للبقاء متحفزًا**

**أهداف التعلم**

في ختام هذه الجلسة ، سيتمكن المشاركون من:

- قياس تقدمهم نحو أهداف الوزن والنشاط البدني منذ الجلسة الأولى.
- وضع خطة لتحسين التقدم ، إذا لم تتحقق أهدافهم بعد.
- وصف طرقًا للبقاء متحفزًا أو متحمسا على المدى الطويل.

**المواد**

- نشرات المشاركين للجلسة 16
- نظرة عامة على الجلسة 16
- مراجعة التقدم
- طرق للحفاظ على الحماس
- خطة للبقاء متحمسًا
- مهام الأسبوع المقبل
- متتبعات الطعام والنشاط للجلسة 16
- السبورة مع الأقلام
- نظرة عامة على الجلسة 16

اختتم البرنامج من خلال تقييم تقدم كل مشارك ومساعدة كل منهم على إيجاد طرق للبقاء متحفزًا بعد البرنامج.

**تنقسم الجلسة 16 إلى 4 أجزاء:**

**الجزء 1: التقدم الأسبوعي والمراجعة (10 دقائق)**

راجع المعلومات التي تم تناولها في الجلسة 15. ناقش نجاحات المشاركين والتحديات والأسئلة منذ آخر مرة اجتمعت فيها المجموعة.

**الجزء الثاني: مراجعة الأهداف والإنجازات (20 دقيقة)**

اطلب من المشاركين التفكير في إنجازاتهم خلال آخر 16 جلسة. راجع تقدم كل شخص نحو أهدافه منذ بداية البرنامج ، وبالنسبة لأولئك الذين لم يحققوا أهدافهم بعد ، أوصي بطرق لمواصلة العمل نحو تحقيقها.

**الجزء 3: البقاء متحفزًا (20 دقيقة)**

ركز على مساعدة المشاركين في إيجاد طرق للبقاء متحمسين. قدم لهم قائمة من الاقتراحات للبقاء متحمسًا ، واطلب منهم اختيار الاستراتيجيات التي تناسبهم بشكل أفضل.

**الجزء 4: الخاتمة و قائمة المهام (10 دقائق)**

باستخدام الأدوات والمعلومات التي تم تعلمها خلال الجلسات الـ 16 ، ساعد المشاركين على بناء خطة طويلة الأجل للبقاء متحمسين أثناء انتقالهم إلى مرحلة ما بعد الأساسية للبرنامج

**الرسائل الرئيسية**

- **لقد أنجزتم أشياء عظيمة واتخذتم خطوات مهمة نحو أسلوب حياة أكثر صحة والوقاية من أمراض القلب.**
- **على الرغم من أننا انهينا 16 جلسة ، إلا أن أهداف فقدان الوزن والنشاط البدني تظل سارية لبقية حياتك.**
- **يعد البقاء متحمسًا أمرًا ضروريًا للحفاظ على الأكل الصحي والنشاط البدني على المدى الطويل ، لكن البقاء متحمسًا هو أحد أكبر المشاكل التي يواجهها الناس.**
- **حافظ على نجاحاتك وتقدمك حتى الآن. إن معرفة ما يمكنك فعله يساعد في تحفيزك للاستمرار في نمط حياتك الجديد الأكثر صحة.**

**الجزء 1: التقدم الأسبوعي والمراجعة (10 دقائق)**

**وزع:**

- توزيعات او منشورات الجلسة 16
- تتبع الطعام والنشاط مع ملاحظاتك للجلسة 14.
- اجمع: متتبعي الطعام والنشاط للجلسة 15

**اسأل**: هل واجهتك أي مشكلة في تتبع المسار الأسبوع الماضي؟ هل كنت قادرًا على البقاء في حدود ميزانيات الدهون والسعرات الحرارية؟ هل وصلت إلى هدفك من النشاط البدني؟

**افتح المجال للرد**

**حاضر:** تحدثنا الأسبوع الماضي عن طرق الوقاية من الإجهاد السلبي أو التعامل معه بما في ذلك التوتر المرتبط بجهودك في إجراء تغييرات صحية في نمط الحياة. نظرنا في طرق لإدارة المواقف العصيبة وتوصلنا إلى خطة للعمل على منع المواقف العصيبة أو التعامل معها خلال الأسبوع الماضي.

**اسأل:** هل كنت قادرًا على اتباع خطة العمل الخاصة بك لمنع أو التعامل مع مصدر واحد للتوتر؟

**افتح المجال للرد**

**امدح** كل تقدم.

**حاضر:** لقد وصلت إلى الجلسة 16 من برنامج صحة القلب الإماراتي! لقد أنجزت أشياء عظيمة منذ الأسبوع الأول: لقد اتخذت خطوات مهمة نحو تقليل مخاطر الإصابة بأمراض القلب ونحو عيش حياة أكثر صحة.

**تهانينا!**

**هذا الأسبوع سوف:**

- نناقش إنجازاتكم الكثيرة منذ الجلسة الأولى وتقدمكم نحو أهدافكم.
- نتحدث عن الطرق التي يمكنك من خلالها البقاء متحمسين حتى تتمكنوا من الحصول على هذه الفوائد لبقية حياتك.

**الجزء الثاني: مراجعة الأهداف والإنجازات (20 دقيقة)**

(ملاحظة: أثناء تهنئة المشاركين على إكمال البرنامج ، اذكر بإيجاز إنجازًا شخصيًا لكل منهم ؛ مساهمة خاصة للمجموعة ، والمثابرة على تحقيق هدفهم ، وما إلى ذلك)

**حاضر**: ضع في اعتبارك أن أهداف فقدان الوزن والنشاط البدني تظل سارية لبقية حياتك.

(ملاحظة: إذا لم يصل بعض المشاركين إلى أهدافهم ، فقم بالتأكيد على أهمية الاستمرار في العمل نحو أهدافهم. بالنسبة للمشاركين الذين حققوا أهدافهم ، أكدوا على أهمية الحفاظ على أوزانهم ومستوى نشاطهم ووضع أهداف جديدة.)

**حاضر**: سنتحدث اليوم عن كيفية البقاء متحمسًا ومتحفزا على المدى الطويل ، وكيفية جعل التزامك بالأكل الصحي والنشاط البدني يدومان لبقية حياتك. لكن أولاً ، دعنا نراجع تقدمك منذ بداية البرنامج.

**ارجع** إلى نشرة "مراجعة التقدم".

**اسأل:** ما هي بعض التغييرات الرئيسية التي أجريتها لتكون أكثر نشاطًا؟

**افتح المجال للرد**

**حاضر:** خذ دقيقة لكتابة هذه التغييرات في النشرة. قم بتضمين كل من الأنشطة التي تقوم بها والتي قمت بتسجيلها بالإضافة إلى التغييرات التي أجريتها لتكون أكثر نشاطًا بشكل عام ( مثل صعود الدرج بدلاً من المصعد).

**اسأل:** ما هي التغييرات التي أجريتها - لتناول كميات أقل من الدهون والسعرات حرارية؟

**افتح المجال للرد**

**حاضر**: اكتب التغييرات في عاداتك الغذائية في النشرة.

(ملاحظة: امدح المشاركين على التغييرات ، وشجعهم على الاستمرار).

**اسأل:** هل وصلت إلى الوزن المطلوب؟ ماذا عن هدف نشاطك؟

**ارجع** إلى "كيف أفعل؟ " مخططات الوزن والنشاط البدني.

**حاضر:** حدد المربعات الموجودة في نشرة "مراجعة التقدم" لتسجيل ما إذا كنت قد حققت أهدافك المحددة في بداية البرنامج أم لا.

إذا لم تصل الى هدف وزنك أو أهداف نشاطك ، فخذ بضع دقائق للتفكير فيه واكتب ما يمكنك فعله لتحسين تقدمك.

إذا حققت أهدافك ، فاختر أهدافًا جديدة للأشهر الستة القادمة. على سبيل المثال ، هل تريد الاستمرار في فقدان الوزن ، أم تريد الحفاظ على وزنك الحالي؟ هل تريد زيادة نشاطك البدني أكثر؟ أم تريد المحافظة على المستوى الحالي؟

**امدح** كل التقدم.

**الجزء 3: البقاء متحمسا او متحفزا (20 دقيقة)**

**حاضر**: التحفيز أمر بالغ الأهمية للحفاظ على الأكل الصحي والنشاط البدني على المدى الطويل ، ولكن البقاء متحمسًا للقيام بذلك هو أحد أكبر المشاكل التي يواجهها الناس.

أحيانًا يكون البقاء متحفزًا أمرًا صعبًا لمجرد أننا نعمل بشكل جيد. هذا غريب - تقدمنا ​​نفسه يجعل من الصعب الحفاظ على هذا التقدم. لكن فكر في الوقت الذي بدأت فيه هذا البرنامج لأول مرة.

(ملاحظة: اضبط الأمثلة التالية لتناسب الخبرات التي شاركها المشاركون حتى الآن.)

شعرت بالتعب عند المشي ، مما دفعك إلى أن تصبح أكثر نشاطًا. الآن بعد أن أصبحت أكثر نشاطًا ، أصبح المشي سهلًا. هذا يعني أن مصدر تحفيزك - الشعور بضيق في التنفس عند المشي - قد انتهى.

أو ربما لم تعجبك الطريقة التي كنت بها. إذا كنت تحب الطريقة التي تبدو بها الآن أفضل ، فهي ليست دافعًا قويًا لك.

**حاضر:** ومع ذلك ، من الممكن أن تظل متحمسًا على المدى الطويل ، وكما ناقشنا ، فإن البقاء متحمسًا أمر مهم للحفاظ على الأكل الصحي والنشاط المنتظم.

**ارجع** لنشرة "طرق البقاء متحفزًا".

**حاضر:** فيما يلي 9 استراتيجيات وجدها الآخرون مفيدة للبقاء متحفزين

1. كن على دراية بالأهداف التي حققتها بالفعل والأهداف التي تخطط لبلوغها.
2. اعترف بنجاحاتك.
3. احتفظ بعلامات واضحة على تقدمك.
4. تتبع وزنك وتناول الطعام والنشاط.
5. إضافة التنوع إلى روتينك.
6. تحديد استراتيجيات إضافية.
7. خلق بعض المنافسة الودية.
8. تذكر ، الزلات او الاخطاء أمر طبيعي.
9. انظر للآخرين لتظل متحفزًا.

دعونا نلقي نظرة على كل استراتيجية بمزيد من التفصيل.

**الإستراتيجية رقم 1: كن على دراية بالأهداف التي حققتها بالفعل والأهداف التي تخطط للوصول إليها.**

مرة أخرى ، تذكر متى بدأت البرنامج لأول مرة. ما هي الأهداف التي حددتها؟

الآن فكر في مكانك اليوم. لقد قطعت شوطا طويلا.

(ملاحظة: الاعتراف بأي نتائج إيجابية أو سلبية في العملية)

**اسأل:** هل وصلت إلى الأهداف التي حددتها لكي تحققها؟ هل تلقيت أي مزايا لم تكن تتوقعها؟ اكتب تلك الفوائد على نشرة الخاص بك.

**اسأل:** ما الذي ترغب في تحقيقه في الأشهر الستة المقبلة؟ اكتب هذه على نشرة الخاص بك.

**اطلب من :**متطوعين لمشاركة ما يريدون تحقيقه.

**الإستراتيجية رقم 2: اعترف بنجاحاتك.**

**اسأل:** ما هي التغييرات في عادات الأكل والنشاط البدني التي تفتخر بها؟

ما الذي كان أسهل في التغيير مما كنت تعتقد أنه سيكون؟

ما الذي كان من الصعب تغييره أكثر مما كنت تعتقد أنه سيكون؟

الحاضر: عندما ينخفض ​​الدافع لديك (وسوف يحدث) ، فكر في هذه التغييرات الإيجابية ، وامنح نفسك الفضل لها.

**الإستراتيجية رقم 3: احتفظ بإشارات واضحة لتقدمك.**

**اسأل:** ما هي بعض الطرق التي يمكنك القيام بها؟ أي أفكار تود مشاركتها؟

**افتح المجال للرد**

**حاضر**: فيما يلي بعض الاقتراحات التي استخدمها الآخرون:

- قم بقياس نفسك شهريًا.
- اطبع خريطة حيك. حدد المسافة التي يمكنك المشي فيها. ابحث عن مكان يتجاوز ذلك. استمر في تحديد تقدمك حتى تتمكن من الوصول إلى هناك. كافئ نفسك

**الاستراتيجية رقم 4: تتبع وزنك وطعامك ونشاطك البدني.**

**حاضر:** من الشائع بالنسبة لنا الانتقال ببطء من العادات الجديدة إلى العادات القديمة. يمكنك فقدان السيطرة تدريجيًا وإجراء تغييرات طفيفة في طعامك ونشاطك البدني على مدى فترة طويلة ولا تدرك حتى أنك تعود ببطء إلى عاداتك القديمة. أفضل طريقة لمنع ذلك والبقاء مسيطرًا هي الاستمرار في تتبع وزنك وتناول الطعام والنشاط. سيساعدك هذا على اكتشاف التغييرات في وقت مبكر.

يرجى تدوين على النشرة كيف تخطط لمتابعة تتبع هذه الأشياء.

كلما تتبعت ، كان ذلك أفضل. حاول أن تتابع كيف تأكل أسبوعًا واحدًا على الأقل من الشهر. إذا لاحظت أنك تكتسب وزناً ، فعليك العودة لمتابعة ما تأكله كل يوم.

**الإستراتيجية رقم 5: أضف مجموعة متنوعة إلى روتينك.**

**حاضر:** لقد تحدثنا عن هذا سابقا. هل كنت قادرًا على التنويع في الانشطة الرياضية للحفاظ على نفسك من الملل من النشاط البدني؟ هل لاحظت اختلافًا في شعورك تجاه النشاط البدني؟

الشيء نفسه ينطبق على الأكل. ليس عليك أن تأكل نفس الشيء كل ليلة. جرب أطعمة مختلفة قليلة الدسم ومنخفضة السعرات الحرارية. جرب أطعمة جديدة ، قم بزيارة مطاعم جديدة.

**اسأل:** ما هي الوجبات أو الوجبات الخفيفة أو الأطعمة المحددة التي أصبحت مملة بالنسبة لك؟ هل يمكنك التفكير في بعض الطرق لتنويع طعامك؟ هل لدى أي شخص اقتراحات بشأن الأطعمة الصحية التي يستمتع بها؟

**افتح المجال للرد**

**حاضر**: (إذا لم يتم مشاركته بالفعل ، يمكنك استخدام هذه.) فيما يلي بعض الاقتراحات لإضافة مجموعة متنوعة إلى الطعام:

- استخدم توابل أو بهارات مختلفة لإضافة نكهة للأطباق قليلة الدسم.
- تناول مجموعة كبيرة من الفواكه والخضروات والحبوب المختلفة.
- احصل على مجموعة متنوعة من الألوان والقوام والنكهات على طبقك.
- اجعل ليلة واحدة في الأسبوع تتناول فيها طعامًا جديدًا.
- إذا كنت تأكل بالخارج كثيرًا ، فخطط لتناول المزيد من الوجبات في المنزل.
- إذا كنت تأكل في المنزل ، فغالبًا ما ابحث عن بعض المطاعم التي تقدم طعامًا صحيًا تريد تجربته. أو اضبط كمية الطعام التي تتناولها والنشاط البدني للسماح لك بتناول الطعام الذي تستمتع به حقًا من حين لآخر.

حاضر: ارجوا منكم كتابة افكاركم على النشرة.

**الاستراتيجية رقم 6: حدد طرقًا جديدة لتحدي نفسك. كافئ نفسك عندما تصل إلى هدفك.**

**حاضر:** يجب أن تكون التحديات الجديدة محددة وقصيرة المدى ("لن أشرب أي كولا هذا الأسبوع.") يجب أن يكون شيئًا ليس سهلاً للغاية أو ليس صعبًا للغاية. يجب أن يكون شيئًا يمكنك القيام به ، ولكن أيضًا صعبًا بما يكفي لتشعر أنك فعلت شيئًا حقًا إذا نجحت.

يجب أن تكون المكافأة شيئًا ستفعله أو تشتريه إذا وصلت إلى هدفك فقط. لا يجب أن تكون باهظة الثمن.

على سبيل المثال ، "بعد أن أمشي عشر دقائق أخرى ، سأشاهد بعض مقاطع الفيديو المضحكة على هاتفي المحمول." بعد ذلك ، إذا كنت بحاجة إلى دفعة للاستمرار ، فيمكنك التفكير في مقدار الضحك بعد المشي.

**اسأل:** ما هي بعض الطرق التي يمكنك بها مكافأة نفسك بدون طعام؟

**افتح المجال للرد**

**قدم** هذه الأفكار:

- الورود
- طلاء الاظافر
- خصص مبلغًا صغيرًا من المال لشيء تريد القيام به أو شرائه.

**اطلب** من المشاركين كتابة أفكار المكافآت الخاصة بهم على النشرة.

**الاستراتيجية رقم 7: إنشاء بعض المنافسة الودية.**

**حاضر**: خوض منافسة ودية مع صديق أو أحد أفراد عائلتك. يجب أن تكون الجائزة شيئًا تفوز بهما كلاكما.

هذه بعض الاقتراحات:

- إذا كنت أنت وصديقك نشيطين كل يوم لمدة شهر ، فستكون ______________ في نهاية الشهر.
- إذا كنت نشيطًا كل يوم وقريبتك تؤدي واجباتها المدرسية كل يوم لمدة شهر ، فستتعاملان كلاكما مع _____________.
- تعرف على عدد الأيام المتتالية التي يمكنك أن تكون فيها نشطًا لمدة 30 دقيقة على الأقل. حاول التغلب على أفضل ما لديك. على سبيل المثال ، إذا كنت نشطًا لمدة ثلاثة أيام متتالية الشهر الماضي ، فتأكد مما إذا كان يمكنك القيام بأربعة أيام هذا الشهر.

**الإستراتيجية رقم 8: تذكر أن الزلات أمر طبيعي.**

**حاضر:** إذا انحرفت عن المسار. ابدأ في التركيز على كيفية العودة إلى المسار الصحيح.في اقرب وقت ممكن - تذكر أن الزلات جزء طبيعي من العملية ستيساعدك على الاستمرار في التطلع إلى هدفك .

**الإستراتيجية رقم 9: ابحث عن الآخرين للمساعدة في البقاء متحمسًا.**

**حاضر:** لديك الآن مجموعة من الأشخاص يفهمون ما تحاول القيام به. ساعدوا بعضكم البعض على البقاء متحمسًا. يواجه الجميع صعوبة في التركيز أحيانًا ، ولكن يمكننا تشجيع ودعم بعضنا البعض خلال تلك الأوقات.

**الجزء 4: خاتمة وقائمة المهام (10 دقائق)**

**حاضر**: وصلنا إلى نهاية هذا البرنامج. استمر في استخدام الأدوات التي تعلمتها خلال هذه الجلسات الـ 16. استمر في المراقبة الذاتية ، وحدد الأهداف ، وابقَ متحمسًا ، وتغلب على التحديات .

دعنا الآن نضع خطة للبقاء متحفزًا أثناء تقدمك.

**ارجع** نشرة "خطة للبقاء متحفزًا".

**حاضر**: ضع خطة تحفزك. اختر ما تعتقد أنه الأفضل بالنسبة لك. اختر شيئًا من المحتمل أن ينجح ويمكنك القيام به. كن واقعيًا ومحددًا. اكتب ما تختار القيام به.

**لخص هذه النقاط الرئيسية:**

لقد أكملت برنامج صحة القلب الإماراتي. لكننا نريدك أن تواصل وتحسن نظامك الغذائي ونشاطك البدني.

حقق بعضكم هدفك ، وما زال البعض منكم على الطريق. لا تستسلم. ما يهم أكثر هو تصميمك على مواصلة العمل في اتجاه صحي.

هناك العديد من الطرق للبقاء متحمسًا. اختر الطريقة أو الطرق التي تناسبك بشكل أفضل. إذا لم ينجح الأمر ، فاختر واحدًا آخر وحاول مرة أخرى.

**الخاتمة:** تذكر المسافة التي قطعتها. لديك الآن الأدوات والالتزام لإجراء تغييرات إيجابية في نمط الحياة والحفاظ عليها. يمكنك النجاح في تحقيق الأهداف التي حددتها لنفسك.

لديك القدرة على اختيار ما تأكله ومدى نشاطك البدني. لديك القدرة على التحكم في محيطك ، والقدرة على قول لا بطريقة ودية ولكن حازمة ، والقدرة على تخصيص الوقت للاعتناء بنفسك ، والقدرة على منع الأفكار السلبية ، والقدرة على التغيير.

**اسأل** إذا كان هناك أي أسئلة أو مخاوف.

**ذكر** المشاركين سيحتاجون إلى فحص مختبراتهم وضغط الدم والوزن في العيادة.

**ذكّر** المشاركين بأن دعم أخصائي التغذية متاح من خلال الخدمات الصحية المتنقلة

**اجمع** المعدات لإعادتها إلى جامعة الإمارات العربية المتحدة.
