# Supplementary material for: Emirates Heart Health Project (EHHP): A protocol for a stepped-wedge family-cluster randomized-controlled trial of a health-coach guided diet and exercise intervention to reduce weight and cardiovascular risk in overweight and obese UAE nationals
Source: PLoS One. 2023 Apr 10;18(4):e0282502. doi: 10.1371/journal.pone.0282502 (PMC10085020; doi:10.1371/journal.pone.0282502)
Supplement: S36 Appendix — (DOCX) [file pone.0282502.s036.docx]

Appendix 38

**Table 2: Assessments involving participants in the EHHP**

| Activity/assessment | Staff member | Approximate time to complete (minutes) | Baseline | Post intervention |
| --- | --- | --- | --- | --- |
| Prescreening consent/inclusion and exclusion screen | Investigators in the family medicine clinic (AK, KC) | 15 | X |  |
| Consent form | Investigators in the family medicine clinic (AK, KC) | 15 | X |  |
| Biometric measurements (blood pressure, weight, height, BMI, waist circumference) | Clinic nurse | 5 | X | X |
| Blood draw | Hospital phlebotomist | 15 | X | X |
| Termination form | Investigators in the family medicine clinic (AK, KC) | 5 |  | X |
| Serious adverse event form | Investigators in the family medicine clinic (AK, KC) | 5 | As needed during throughout study period |  |
| Progress notes | Health coach | 5 | Weekly |  |
| Communication log | All team members | 5 | Every phone or email contact outside of a regular visit |  |
