# Supplementary material for: Emirates Heart Health Project (EHHP): A protocol for a stepped-wedge family-cluster randomized-controlled trial of a health-coach guided diet and exercise intervention to reduce weight and cardiovascular risk in overweight and obese UAE nationals
Source: PLoS One. 2023 Apr 10;18(4):e0282502. doi: 10.1371/journal.pone.0282502 (PMC10085020; doi:10.1371/journal.pone.0282502)
Supplement: S5 Appendix — (PDF) [file pone.0282502.s005.pdf]

مستشفى كند
KANAD HOSPITAL
إيمان أمل سلام محبة
FAITH HOPE PEACE LOVE

مهد لقادة الوطن منذ عام
Birthplace of Leaders for the Nation
Since 1960

Dr Jeffrey K King
Department of Family Medicine, College of Medicine and Health Sciences, United Arab Emirates University
jking@uaeu.ac.ae

16th March 2022

Study Title: The Emirates Heart Health Project
Short Title: EHHP
Sponsor: United Arab Emirates University, Grant number 31M450
Principal Investigator: Dr. Jeffrey K King
Site Investigator: Dr. Alexander Kieu and Dr. Kristoffer Crawford

Dear Dr. King,

Thank you for submitting the above referenced study to Kanad Hospital Research Ethics Committee which is registered with the Department of Health (Research Authorisation #2012.15).

The research proposal was discussed, assessed and registered during a meeting held on 16th March 2022 and attended by a quorum of the Research Ethics Committee. I am pleased to confirm that approval has been given for the amendment, on the basis described in the application, protocol, and supporting documentation

Study commencement and continuation is conditional on the following conditions:

Investigators must:
1. Follow the regulatory requirements described in DOH Standard on Human Subjects Research 2020 (DOH/ QD/SD/HSR/0.9) and Healthcare Regulator Policy Manual, The Health Authority Abu Dhabi 2012.
2. Notify the Research Ethics Committee if there is any reason to review the ethical approval including any serious or unexpected adverse events, changes to the study protocol, study team or to the conduct of research, or new information that may adversely affect the safety of subjects.
3. Submit an Annual Report about the progress of the study
4. Submit a Final Report on completion of the study

The approval for the study expires on 16th March 2023 and continuing approval of the research will be subject to submitting a request for renewal to the Research Ethics Committee.

We wish you every success with your research study.

Yours sincerely,

Dr Nicholas Hoque
Chairperson, Kanad Hospital Research Ethics Committee
